# Bias-corrected maximum-likelihood estimation of multiplicity of infection and lineage frequencies

**DOI:** 10.1371/journal.pone.0261889

**Published:** 2021-12-29

**Authors:** Meraj Hashemi, Kristan A. Schneider

**Affiliations:** Department of Applied Computer- and Biosciences, University of Applied Sciences Mittweida, Mittweida, Germany; Arizona State University, UNITED STATES

## Abstract

**Background:**

The UN’s Sustainable Development Goals are devoted to eradicate a range of infectious diseases to achieve global well-being. These efforts require monitoring disease transmission at a level that differentiates between pathogen variants at the genetic/molecular level. In fact, the advantages of genetic (molecular) measures like multiplicity of infection (MOI) over traditional metrics, e.g., *R*_0_, are being increasingly recognized. MOI refers to the presence of multiple pathogen variants within an infection due to multiple infective contacts. Maximum-likelihood (ML) methods have been proposed to derive MOI and pathogen-lineage frequencies from molecular data. However, these methods are biased.

**Methods and findings:**

Based on a single molecular marker, we derive a bias-corrected ML estimator for MOI and pathogen-lineage frequencies. We further improve these estimators by heuristical adjustments that compensate shortcomings in the derivation of the bias correction, which implicitly assumes that data lies in the interior of the observational space. The finite sample properties of the different variants of the bias-corrected estimators are investigated by a systematic simulation study. In particular, we investigate the performance of the estimator in terms of bias, variance, and robustness against model violations. The corrections successfully remove bias except for extreme parameters that likely yield uninformative data, which cannot sustain accurate parameter estimation. Heuristic adjustments further improve the bias correction, particularly for small sample sizes. The bias corrections also reduce the estimators’ variances, which coincide with the Cramér-Rao lower bound. The estimators are reasonably robust against model violations.

**Conclusions:**

Applying bias corrections can substantially improve the quality of MOI estimates, particularly in areas of low as well as areas of high transmission—in both cases estimates tend to be biased. The bias-corrected estimators are (almost) unbiased and their variance coincides with the Cramér-Rao lower bound, suggesting that no further improvements are possible unless additional information is provided. Additional information can be obtained by combining data from several molecular markers, or by including information that allows stratifying the data into heterogeneous groups.

## Introduction

The UN’s Sustainable Development Goals (SDGs; see [1]) are devoted to eradicate a range of infectious diseases to achieve global well-being. These efforts require monitoring disease transmission at a resolution that differentiates between pathogen variants at the genetic/molecular level. This is because, when switching the focus from disease control toward elimination, routes of transmission need to be identified (cf. [2]), which requires to distinguish between pathogen variants that are circulating within an endemic population and those that are imported. The gold standards to measure transmission are still the entomological inoculation rate (EIR) and the basic reproduction number *R*_0_ [3, 4]. However, molecular metrics, e.g., multiplicity of infection (MOI) and molecular force of infection (mFOI), are recognized as being more appropriate [4]. Moreover, genetic (molecular) measures like multiplicity of infection (MOI) have advantages over traditional metrics, e.g., *R*_0_, which rely on incidence data and healthcare records that are notoriously difficult to maintain. Although technically more challenging, the former can be appropriately estimated from selected study sites with an appropriate sample designs. The importance of MOI is well established in malaria and increasingly becoming recognized in other infectious diseases [5].

The SDG particularly aim to end the malaria epidemic by the year 2030. Malaria is caused by several species of unicellular eukaryotic parasites of genus *Plasmodium*. It is a vector-borne disease transmitted by several species of anopheles mosquitoes. With half of the world’s population living at the risk of infection, malaria is considered a major obstacle to global development. While programs such as the President’s Malaria Initiative (PMI) substantially contributed in lowering transmission, by introducing long-lasting insecticide-treated bed nets, rapid diagnostic tests (RDTs) and artemisinin-based combination therapies (ACTs) since the early 2000s, in 2018 the number of malaria cases increased after several years of steady decrease (WHO 2018 [6]). Nevertheless, plans to eradicate malaria in several endemic regions remain ambitious, e.g., in India malaria elimination is targeted by 2030 [7]. Recently, successful malaria-control interventions are challenged by the spread of (i) insecticide resistance [8, 9], (ii) HRP2/3 deletions in the parasite’s genome [10, 11], which cause false-negative RDT results, and (iii) drug resistance. Particularly, the spread of artimisinin resistance (especially mutations in the propeller region of the K13 gene of *P.falciparum*) in the greater MeKong subregion is a source of concern [12]. This entails putting forward efficient tools for monitoring malaria epidemiology and reliably measure the impact of new and existing control interventions aiming to reduce malaria transmission.

MOI’s incidence (or superparasitism per se) is epidemiologically an important metric of exposure in infectious diseases (cf. [13]). However, the definitions of MOI, or complexity of infection (COI) [14, 15], are ambiguous in the literature (cf. Model background, Alternative definitions of MOI). Here, MOI refers to the number of super-infections due to multiple infectious contacts, which often (but not always) lead to multiclonal infections [16, 17]. Apart from their association to transmission, multiclonal infections are believed to affect intra-host dynamics, characterized by complex interactions between genetically distinct parasite lineages [18]. In malaria, the epidemiological importance of MOI in relation to disease severity (and its implications on identifying mutations associated with drug resistance) are well recognized [19–21]. The concept of MOI applies to other infectious diseases likewise, although it has not been recognized as much as in malaria.

In many studies, ad-hoc methods are used to provide estimates for MOI and lineage frequencies from molecular/genetic data. Although these methods are intuitive, they usually yield biased results. In several studies, an estimation of MOI is derived as the total number of distinct genotypes detected at a marker divided by the number of disease-positive samples [22]. Regularly, this approach is applied to multiple markers to derive one estimate of MOI per marker [23, 24]. Some authors calculate MOI for each sample in a dataset as the maximum number of alleles observed across several marker loci (typically STR markers, e.g., [18, 25]), or the mean number of alleles across all loci [26]. For SNP data, in [27] counts of the number of heterozygous SNPs are used in each sample to define multiclonal infections relating to MOI. Because haplotypes super- or co-infecting a host can carry the same allele at one or many loci, these approaches might substantially underestimate MOI. On the contrary, if a large number of markers is considered, MOI might be overestimated as sequencing or allele-calling errors accumulate. (See Model background, Alternative definitions of MOI for a formal discussion.)

A formal approach is to build a uniform statistical framework providing adequate estimations of MOI while accounting for confounding factors. In the context of malaria, such a framework was introduced by [28] and further developed by [16, 29]. This approach employs molecular data from a collection of blood samples of disease-positive patients to obtain maximum-likelihood estimates (MLEs) for MOI and the frequency spectrum of pathogen lineages. A comprehensive investigation on the MLEs’ performance showed that the method has the typical desirable asymptotic properties of an estimator, i.e., asymptotic unbiasedness, strong consistency, and efficiency [17]. In spite of these large-sample properties, the method yields biased results in finite-sample settings. More precisely, while lineage-frequency (allele-frequency) estimates are unbiased, the MOI estimate is typically biased. The bias of the MOI estimator was assessed in detail in [17]: the estimator is most biased in either low- or high-transmission setting for the following reasons. While in low-transmission setting, the estimates will typically slightly underestimate the true parameter, occasionally in the data samples with several lineages present are over-represented, which results in substantial overestimates of the true parameter. Overall this leads to positive bias, which is substantial in relative terms but not in absolute terms. In high-transmission settings, it is likely that samples with multiple lineages are over-represented, which lead to substantial overestimates due to the Poisson model (cf. [17]). In mathematical terms, the bias of the MLE is of order $O(N^{-1})$ [30]. In areas of high transmission, in future studies the problem of bias can be solved by aiming for larger sample sizes. However, in low-transmission settings, bias is appreciable even for moderate sample sizes. This is a considerable shortcoming, because collecting a large number of clinical samples in low-transmission settings is challenging—transmission intensities correlate with disease prevalence through MOI. Therefore, applying a bias correction becomes essential—particularly in areas of low and high transmission, when the collection of large sample sizes is not feasible. Moreover, bias depends on the skewness of the lineage-frequency distribution, with markers with more balanced distributions—typically reflecting neutral markers—leading to less biased results (cf. [17]). Molecular markers which are under selection will tend to have more skewed lineage-frequency distributions, e.g., markers in the vicinity of drug resistance associated genes in malaria or PCR-RFLP data on msp genes. In such situations, it is also recommendable to apply bias corrections.

Here, we present an analytical bias adjustment to the MLE, which reduces bias to the order $O(N^{-2})$. We adopt the method for bias correction outlined in [31], which requires the likelihood function to be “well-behaved’’, i.e., fulfills the standard regularity conditions. This is the case here. The method derives the second-order biases of the MLEs for MOI and lineage frequencies. We provide explicit formulas for bias in terms of the true and estimated parameters. Moreover, we consider a heuristic approach to introduce several adjustments to the bias corrections. To investigate the improvement by bias correction, we conduct a systematic numerical study. Namely, performance of the MLE is compared to its bias-corrected counterparts by quantifying the estimator’s empirical bias (accuracy) and variance (precision). Moreover, the robustness of the estimators is investigated under model violations assuming that MOI follows a negative binomial distribution rather than a Poisson distribution as assumed by the method introduced here. MOI estimates tend to be most biased in areas of either low or high transmission. The bias correction substantially reduces bias of MOI estimates in such situations.

Readers with a more applied focus, shall feel free to skip the more technical parts of the Model Background, and the Analytical Results and move directly to Finite Sample Properties.

## Model background

**Fig 1:**
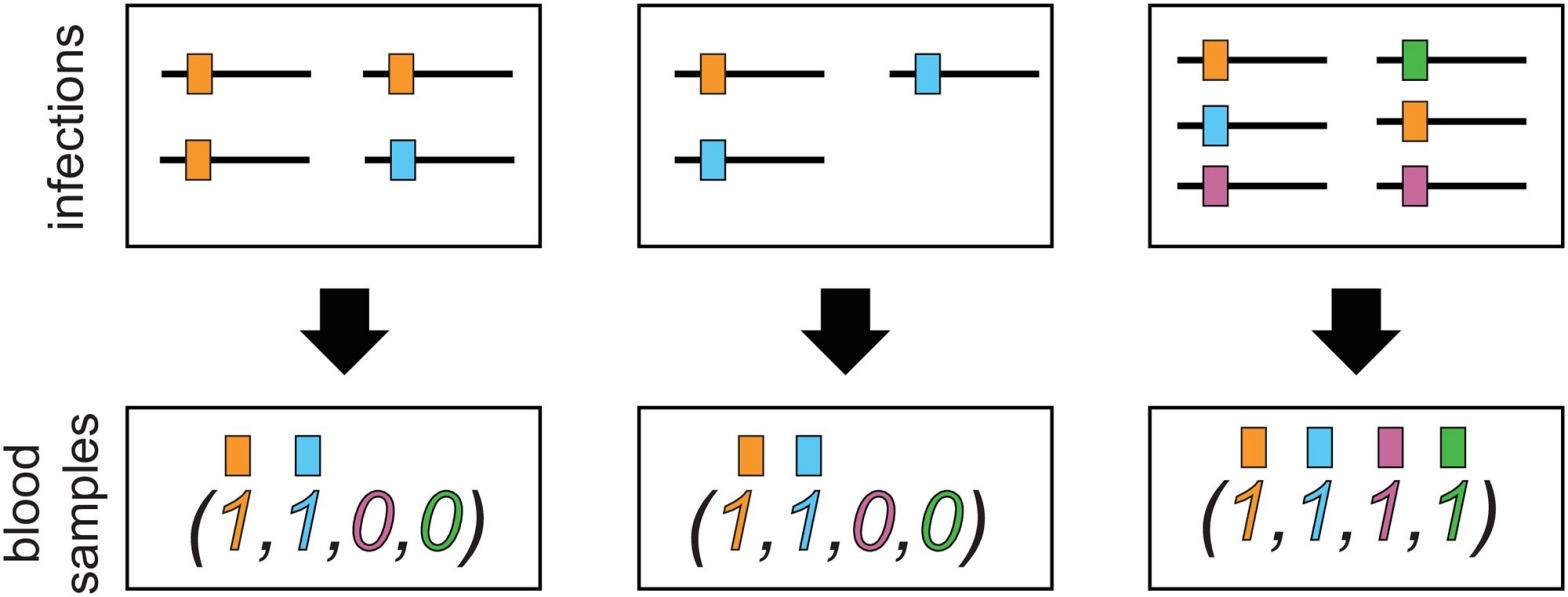
Illustration of observable and unobservable information. Illustration of information contained in blood samples. The top row illustrated three (super-)infections. The bottom row illustrates the respective information about the infection that can be reconstructed from a blood sample. The first individual was infected by *m* = 4 lineages, three times with the orange and once with the blue lineage. Hence, the orange and blue lineages are observed in the blood sample, while the pink and green lineages were not observed. In the middle, a super-infection with *m* = 3 lineages is illustrated which differs from the first infection but results in the same observation. All four lineages were infecting in the third example, however, *m* = 6 super-infections occurred.

To estimate the distribution of MOI, we adapt the model of [28], which is also described in [16]. We refer to “lineages” as pathogen variants, identified by allelic variants at a single locus and synonymously use the terms “lineage” and “allele”. Lineages can also be interpreted as haplotypes in a non-recombining region. Now, let us consider *n* different lineages circulating in a given pathogen population. Let us denote the lineages by *A*_1_, …, *A*_*n*_ and their frequencies by *p*_1_, …, *p*_*n*_, respectively. The frequencies can be subsumed by the vector ***p*** = (*p*_1_, …, *p*_*n*_). At each infective event, exactly one lineage is transmitted to a host. However, hosts can be super-infected multiple times with the same or different lineages. Let *m*_*k*_ be the number of times a host is (super-)infected by lineage *A*_*k*_. Therefore, *m* = *m*_1_ + … + *m*_*n*_ is the number of times a host is (super-)infected during the course of its infection (see Fig 1). Note that the model can be reinterpreted to also cover co-infections, i.e., the transmission of several lineages at one infectious bite (see [5] Section 2.1.1).

We assume infective events to be independent. Hence, conditioned on being super-infected *m* times, the probability that the host is infected *m*_*k*_ times with lineage *A*_*k*_ (*k* = 1, …, *n*) follows a multinomial distribution, i.e., P(m|m)=(mm)pm≔m!m1!…mn!p1m1…pnmn. The quantity *m* is called multiplicity of infection (MOI) or complexity of infection (COI) [14]. Note that this definition is not uniformly used in the literature, especially not in empirical studies (cf. Introduction). With no underlying statistical framework, MOI is often referred to as the number of different lineage variants found in a clinical specimen, e.g., blood sample, taken from an infected host (e.g. [17]).

If infections with the disease are rare and independent, the natural assumption is that MOI is Poisson distributed, or more precisely follows a conditional Poisson distribution (CPD), i.e.,

$$\begin{array}{c} \kappa_{m}=\frac{1}{e^{\lambda }-1}\frac{\lambda^{m}}{m!},\;\;\;\;m\geq 1, \end{array}$$ (1)

as only disease-positive hosts are considered. Under this assumption, the distribution of MOI is identified by the Poisson parameter λ. The average MOI is

$$\begin{array}{c} \psi =\frac{\lambda }{1-e^{-\lambda }} \end{array}$$ (2)

(cf. [29]). In practice, for a given infection, *m* is unknown (see Fig 1) and it is impossible to reconstruct ***m*** = (*m*_1_, …, *m*_*n*_). However, it is possible to detect the absence/presence of lineages within an infection. Let *x*_*k*_ ∈ {0, 1} denote the absence/presence of lineage *A*_*k*_ in a blood sample. Therefore, the observed data obtained from a blood sample is represented by the configuration ***x*** = (*x*_1_, …, *x*_*n*_) ∈ {0, 1}^*n*^\{**0**} and relates to the true infection via ***x*** = sign(***m***). Notably, the excluded configuration **0** represents an uninfected host (*m* = 0). The probability of a clinical sample having configuration ***x*** is given by

$$\begin{array}{c} Q_{x}=\frac{1}{e^{\lambda }-1}\prod_{k=1}^{n}{\left(e^{\lambda p_{k}}-1\right)}^{x_{k}}, \end{array}$$ (3)

according to [16]. This model is identifiable, i.e., different sets of parameters lead to different distributions of ***x*** (cf. [17]).

The model parameters λ and ***p*** can be jointly estimated by maximum-likelihood method from *N* disease-positive clinical samples, i.e., from *N* configurations ***x***^(1)^, …, ***x***^(*N*)^. Collectively we denote this dataset by X. Let *N*_*k*_ be the number of clinical samples in which lineage *A*_*k*_ is observed, i.e.,

$$\begin{array}{c} N_{k}=\sum_{j=1}^{N}x_{k}^{\left(j\right)}, \end{array}$$ (4)

where $x_{k}^{\left(j\right)}$ indicates the absence/presence of lineage *A*_*k*_ in the *j*-th sample. The log-likelihood function is given by

$$\begin{array}{c} \ell \left(\lambda ,p\right)=-N\;\text{log}\left(e^{\lambda }-1\right)+\sum_{k=1}^{n}N_{k}\;\text{log}\left(e^{\lambda p_{k}}-1\right), \end{array}$$ (5)

cf. [16]. The MLE is found by maximizing the Lagrange function defined by

$$\begin{array}{c} \Lambda \left(\lambda ,\beta ,p\right)=\ell \left(\lambda ,p\right)+\beta (1-\sum_{k=1}^{n}p_{k}), \end{array}$$ (6)

where *β* is the Lagrange multiplier. Under the Poisson assumption (1), the values *N* and *N*_1_, …, *N*_*n*_ form a sufficient statistic (cf. [16]). Clearly, the values $\frac{N_{1}}{N},\ldots ,\frac{N_{n}}{N}$ are the estimated prevalences of the lineages in the pathogen population.

### Alternative definitions of MOI

A downside in the definition of MOI or COI is that these terms are ambiguously defined in the literature. Typically in the theoretical literature MOI is defined as the number of super-infections or co-infections in the same way as here, e.g., [32–36]. It was also defined in the same way in [28, 37] without explicitly referring to the term MOI, but rather to the “number of malaria clones in blood samples” or “multiple infections”, respectively. This is similar for [38]. In [39], MOI was defined as the Poisson parameter λ rather than to the realizations of a Poisson distribution. In [14, 15, 34] the terms MOI or COI were defined somehow ambiguously, by referring verbally to the number of distinct haplotypes within an infection, but formally to the same quantity as here. In empirical studies, MOI is often referred to the number of distinct pathogen haplotypes within an infection. This quantity is then typically estimated from unphased data, e.g., as the maximum (e.g. [40]) or average (e.g., [41]) of the number of alleles across several markers. Note however, that a number of empirical studies would use MOI exactly as defined here, e.g. [2, 13]. The same is true in [42], where the estimates of haplotype frequencies are based on the same definition of MOI as the one used here.

In any case, defining MOI as here is convenient, because the alternative definitions derive from it. For instance, if MOI was defined as the Poisson parameter as in [39], the connection is obvious. If MOI was defined as the number of the number of distinct lineages within an infection as in [40], the distribution of MOI could be easily derived from Eq (3). For instance the probability of an infection with one detectable lineage would be

$$\begin{array}{c} \sum_{\begin{array}{c} x:\\ |x|=1 \end{array}}Q_{x}=\frac{1}{e^{\lambda }-1}\sum_{k=1}^{n}\left(e^{\lambda p_{k}}-1\right). \end{array}$$ (7)

In general, the probability to observe *m* = 1, …, *n* different lineages in a sample is given by

$$\begin{array}{c} \sum_{\begin{array}{c} x:\\ |x|=m \end{array}}Q_{x}, \end{array}$$ (8)

which is combinatorically more involved, but straightforward to calculate numerically.

### Maximum-likelihood estimate

The maximum-likelihood estimate (MLE) for the model parameters ***θ*** = (λ, ***p***) exists, is unique, and lies in the interior of the parameter space except in two pathological situations [16, 29]. In the first, only one lineage is found in each blood sample ($\sum_{k=1}^{n}N_{k}=N$), i.e., there is no sign of super-infections. In the second, at least one lineage is found in every blood sample, i.e., *N*_*k*_ = *N* for at least one *k*. For regular data (non-pathologic data), the MLE of the model parameters is given by

$$\begin{array}{c} {\hat{p}}_{k}=-\frac{1}{\hat{\lambda }}\;\text{log}(1-\frac{N_{k}}{N}\left(1-e^{-\hat{\lambda }}\right)), \end{array}$$ (9a)

where $\hat{\lambda }$ is derived by iterating

$$\begin{array}{c} \lambda_{t+1}=\lambda_{t}-\frac{\lambda_{t}+\sum_{k=1}^{n}\text{log}(1-\frac{N_{k}}{N}\left(1-e^{-\lambda_{t}}\right))}{1-\sum_{k=1}^{n}\frac{N_{k}}{Ne^{\lambda_{t}}-N_{k}\left(e^{\lambda_{t}}-1\right)}}, \end{array}$$ (9b)

(cf. [16]). The sequence (9b) converges monotonically, at a quadratic rate from any initial value $\lambda_{1}\geq \hat{\lambda }$. Hence, by choosing λ_1_ sufficiently large, the iteration is guaranteed to converge.

In [17] the large and finite sample properties of the MLEs (9) were studied in detail. In particular, it was proven that the MLE has the typical desirable asymptotic properties, i.e., it is asymptotically unbiased ($\underset{N\to \infty }{\text{lim}}\;E\left(\hat{\theta }\right)=\theta$), strongly consistent ($\hat{\theta }\overset{\text{a.s.}}{\to }\theta$), and asymptotically efficient ($\underset{N\to \infty }{\text{lim}}\;I_{N}\;\text{Var}\left(\hat{\theta }\right)=I_{n+1}$, where $I_{N}$ denotes the Fisher information and *I*_*n*+1_ is the (*n* + 1)-dimensional identity matrix, cf. [17]).

Under the standard regularity conditions, the MLE is only asymptotically unbiased. In particular, it carries a bias of order $O(N^{-1})$. Additionally, the MOI parameter has no upper bound, which leads to disproportionately large estimates of λ in cases that super-infections (i.e. samples containing many different lineages) are over-represented in the data. Hence, the estimator’s performance can suffer in terms of precision if sample size is small, i.e., the estimator is biased. However, the estimator can be bias-corrected, which is the aim of this work.

### Cramér-Rao lower bound

The estimator’s covariance matrix is well approximated by the Cramér-Rao lower bound (inverse Fisher information). For the model, the Cramér-Rao lower bound can be derived explicitly as follows (cf. [17]).

**Remark 1**. *The entries of the inverse Fisher information matrix* $I_{N}^{\left(-1\right)}=\left(\tau^{ij}\right)$ *are derived as*

$$\tau^{\left(11\right)}=\frac{1}{N}\frac{{\left(e^{\lambda }-1\right)}^{2}}{e^{\lambda }}\;\;\frac{\gamma }{e^{\lambda }-1-\gamma },$$ (10a)

$$\tau^{\left(1j\right)}=\frac{1}{N}\frac{{\left(e^{\lambda }-1\right)}^{2}}{\lambda e^{\lambda }}\;\;\frac{e^{\lambda p_{j}}-1-p_{j}\gamma }{e^{\lambda }-1-\gamma },$$ (10b)

$$\tau^{\left(ii\right)}=\frac{1}{N}\frac{{\left(e^{\lambda }-1\right)}^{2}}{\lambda^{2}e^{\lambda }}(\frac{e^{\lambda p_{i}}-1}{e^{\lambda }-1}+\frac{p_{i}^{2}\gamma -2p_{i}\left(e^{\lambda p_{i}}-1\right)+\frac{{\left(e^{\lambda p_{i}}-1\right)}^{2}}{e^{\lambda }-1}}{e^{\lambda }-1-\gamma }),$$ (10c)

$$\tau^{\left(ij\right)}=\frac{1}{N}\frac{{\left(e^{\lambda }-1\right)}^{2}}{\lambda^{2}e^{\lambda }}\;\;\frac{p_{i}p_{j}\gamma -p_{i}\left(e^{\lambda p_{j}}-1\right)-p_{j}\left(e^{\lambda p_{i}}-1\right)+\frac{\left(e^{\lambda p_{i}}-1\right)\left(e^{\lambda p_{j}}-1\right)}{e^{\lambda }-1}}{e^{\lambda }-1-\gamma },$$ (10d)

*where*

$$\gamma =\sum_{k=1}^{n}\left(e^{\lambda p_{k}}-1\right)$$ (10e)

*for i, j* = 2, …*n* + 1, *i* ≠ *j*.

This was proven in [17]. For a simplified alternative proof see S1 Appendix. For practical purposes, reporting the average MOI (*ψ*) is more appropriate compared with the Poisson parameter (λ). Since MLEs are transformation respecting, $\hat{\psi }=\frac{\hat{\lambda }}{1-e^{-\hat{\lambda }}}$ holds. The Cramér-Rao lower bound for $\hat{\psi }$ can be calculated as shown in the next remark (cf. [17]).

**Remark 2**. *Let* (λ, *p*_1_, …, *p*_*n*_) *denote the true but unknown parameters, and let τ*^(*ii*)^, *τ*^(*ij*)^ *and γ be given by* (10c), (10d) *and* (10e), *respectively. The Cramér-Rao bound of the MLE* $\left(\hat{\psi },{\hat{p}}_{1},\ldots ,{\hat{p}}_{n}\right)$, *is given by*

$${\tilde{\tau }}^{\left(11\right)}=\frac{e^{\lambda }{\left(e^{\lambda }-1-\lambda \right)}^{2}}{N{\left(e^{\lambda }-1\right)}^{2}}\;\;\frac{\gamma }{e^{\lambda }-1-\gamma }\;$$ (11a)

$${\tilde{\tau }}^{\left(1j\right)}=\frac{e^{\lambda }-\lambda -1}{\lambda N}\;\;\frac{e^{\lambda p_{j}}-1-p_{j}\gamma }{e^{\lambda }-1-\gamma }\;,$$ (11b)

$${\tilde{\tau }}^{\left(ii\right)}=\tau^{\left(ii\right)}\;,$$ (11c)

$${\tilde{\tau }}^{\left(ij\right)}=\tau^{\left(ij\right)}\;,$$ (11d)

*where i*, *j* = 2, …*n* + 1 *and* $\hat{\psi }=\frac{\hat{\lambda }}{1-e^{-\hat{\lambda }}}$ *with* $\hat{\lambda }$ *being the MLE of* λ. *Note that* (11) *cannot be calculated explicitly as the true parameters are unknown. However, it is estimated by substituting the true parameters with the MLE*.

## Analytical results

The approach applied here is “corrective”, i.e., the bias-corrected MLE is constructed by subtracting the bias (estimated at the MLE) from the original MLE. This method requires the log-likelihood function (5) to be regular with respect to all derivatives up to (and including) the third order [31, 43]. We employ the general formula provided in [31] to derive the second-order bias of the MLE.

### Bias correction

The MLE’s bias is of order $O(N^{-1})$. Indeed, bias might be quite large for samples of size *N* < 100, especially if the true λ is small, which translates to fewer super-infections (cf. [17]). Since transmission intensity correlates not only with MOI but also with disease prevalence, it will be difficult to collect a large number of samples in low-transmission settings, rendering *N* ≈ 80 − 100 a realistic sample size [44–47]. On the contrary, in high-transmission regions, super-infections with high MOI are common. However, if the lineage-frequency distribution is skewed, individuals are frequently infected by identical lineages, and hence samples do not provide good evidence for the true MOI—MOI will be underestimated (cf. Fig 1). Therefore, it is important to apply a bias correction to (9). We employ a correction that reduces the bias to the order $O(N^{-2})$ (cf. [30]).

Let

$$\begin{array}{c} \Theta ≔\{\left(\lambda ,\beta ,p\right)\;|\;\lambda \in R^{+},\;\beta \in R\;\text{and}\;p\in \text{int}\;S_{n}\} \end{array}$$ (12)

denote the parameter space of the model, where $\text{int}\;S_{n}=\{\left(x_{1},\ldots ,x_{n}\right)\in R^{n}\;|\;\sum_{k=1}^{n}x_{k}=1\;\text{and}\;0<x_{k}<1\;\forall k\}$ is the interior of the (*n* − 1)–dimensional simplex. For convenience of notation we write ***θ*** = (*θ*_1_, …, *θ*_*n*+2_) for parameter vectors (λ, *β*, *p*_1_, …, *p*_*n*_) ∈ Θ wherever appropriate. Using this notation, the moments of the log-likelihood derivatives are

$$k_{ij}≔E(\frac{\partial^{2}\Lambda }{\partial \theta_{i}\partial \theta_{j}}),$$ (13a)

$$k_{ijl}≔E(\frac{\partial^{3}\Lambda }{\partial \theta_{i}\partial \theta_{j}\partial \theta_{l}}),$$ (13b)

and

$$k_{ij,l}≔E((\frac{\partial^{2}\Lambda }{\partial \theta_{i}\partial \theta_{j}})(\frac{\partial \Lambda }{\partial \theta_{l}})).$$ (13c)

Note that the above moments of the log-likelihood derivatives equal the joint cumulants of the log-likelihood derivatives (cf. [31], Chapter 2, p.16), hence we refer to them as such. The following derivatives of the cumulants are also needed:

$$\begin{array}{c} k_{ij}^{\left(l\right)}≔\frac{\partial k_{ij}}{\partial \theta_{l}}. \end{array}$$ (14)

The second-order bias is calculated to be

$$\begin{array}{c} B_{\theta }\left({\hat{\theta }}_{s}\right)≔E\left({\hat{\theta }}_{s}-\theta_{s}\right)=\sum_{i,j,l=1}^{n+2}\tau^{\left(si\right)}\tau^{\left(jl\right)}(\frac{1}{2}k_{ijl}+k_{ij,l})+O\left(N^{-2}\right), \end{array}$$

(cf. [31]). Using the Bartlett identity $k_{ijl}+k_{ij,l}-k_{ij}^{\left(l\right)}=0$ (see [31]), this can be rewritten as

$$\begin{array}{c} B_{\theta }\left({\hat{\theta }}_{s}\right)=E\left({\hat{\theta }}_{s}-\theta_{s}\right)=\sum_{i,j,l=1}^{n+2}\tau^{\left(si\right)}\tau^{\left(jl\right)}(k_{ij}^{\left(l\right)}-\frac{1}{2}k_{ijl})+O\left(N^{-2}\right). \end{array}$$ (15)

In matrix form, (15) becomes

$$\begin{array}{c} B_{\theta }\left(\hat{\theta }\right)=E\left(\hat{\theta }-\theta \right)=I_{N}^{-1}A\;\text{vec}\left(I_{N}^{-1}\right)+O\left(N^{-2}\right), \end{array}$$ (16)

where $\text{vec}(I_{N}^{-1})$ is the (*n* + 2)^2^-dimensional vector obtained by stacking the columns of the inverse Fisher information, i.e.,

$$\begin{array}{c} \begin{array}{cc} \text{vec}(I_{N}^{-1}) & =(\tau^{\left(11\right)},\ldots ,\tau^{\left((n+2)1\right)},\ldots ,\tau^{\left(1(n+2)\right)},\ldots ,\tau^{\left((n+2)(n+2)\right)}{)}^{T}\\ & =(\tau^{\left(11\right)},\ldots ,\tau^{\left(1(n+2)\right)},\ldots ,\tau^{\left((n+2)1\right)},\ldots ,\tau^{\left((n+2)(n+2)\right)}{)}^{T}. \end{array} \end{array}$$ (17)

The latter equality holds because $I_{N}^{-1}$ is symmetric. Furthermore, the (*n* + 2)^2^ × (*n* + 2)-matrix *A* is constructed as

$$\begin{array}{c} A=(\begin{array}{c} A^{\left(\theta_{1}\right)},A^{\left(\theta_{2}\right)},\ldots ,A^{\left(\theta_{n+2}\right)} \end{array}), \end{array}$$ (18a)

i.e., as the concatenation of the (*n* + 2) × (*n* + 2)-matrices $A^{\left(\theta_{l}\right)}$ having elements

$$\begin{array}{c} a_{ij}^{\left(l\right)}=k_{ij}^{\left(l\right)}-\frac{1}{2}k_{ijl},\;i,j,l=1,\ldots ,\left(n+2\right). \end{array}$$ (18b)

In terms of the model parameters λ and ***p*** this is summarized in the following result.

**Result 1**. *The MLE* $\hat{\lambda }$ *of the MOI parameter* λ *has second-order bias*

$$\begin{array}{c} B_{\theta }\left(\hat{\lambda }\right)=\frac{1-e^{-\lambda }}{N(e^{\lambda }-1-\gamma )}(\frac{\gamma }{2}(e^{\lambda }+1)-\frac{e^{\lambda }-1}{e^{\lambda }-1-\gamma }\sum_{1\leq i<j\leq n}\;\;\;\;\;(e^{\lambda p_{i}}-1)(e^{\lambda p_{j}}-1)\;\;)\\ +O(N^{-2}), \end{array}$$ (19a)

*where*

$$\begin{array}{c} \gamma =\sum_{k=1}^{n}e^{\lambda p_{k}}-1. \end{array}$$ (19b)

*The second-order bias for the MLE* $\hat{p}$ *of lineage frequency p_k_ is*

$$\begin{array}{c} \begin{array}{cc} B_{\theta }\left({\hat{p}}_{k}\right)= & \frac{1\;-\;e^{-\lambda }}{2\lambda N(e^{\lambda }\;-\;1\;-\;\gamma )}(\;\frac{1}{\lambda }(e^{\lambda p_{k}}\;\;-\;1\;-\;p_{k}\gamma )(\lambda \left(e^{\lambda }\;+\;1\right)\;-\;2\left(e^{\lambda }\;-\;1\right))+(e^{\lambda p_{k}}\;\;-\;1{)}^{2}\\ & +\frac{e^{\lambda }\;-1\;}{e^{\lambda }\;-\;1\;-\;\gamma }(\;\gamma (p_{k}\gamma -e^{\lambda p_{k}}+1)+(\frac{e^{\lambda p_{k}}-1}{e^{\lambda }-1}-p_{k})\sum_{j=1}^{n}(e^{\lambda p_{j}}-1{)}^{2}\;)\;)\\ & +O(N^{-2}). \end{array} \end{array}$$ (19c)

The proof of the result is presented in S1 Appendix. As the true parameters are unknown, estimates for the second-order bias are obtained by substituting the MLE for the true parameters and neglecting all terms of order $O(N^{-2})$. This yields the bias-corrected estimates as follows:

**Result 2**. *The bias-corrected MLE (BCMLE) of the MOI parameter* λ *is*

$$\begin{array}{c} {\hat{\lambda }}^{\left(bc\right)}=\hat{\lambda }-B_{\hat{\theta }}\left(\hat{\lambda }\right), \end{array}$$ (20a)

*where*

$$\begin{array}{c} B_{\hat{\theta }}\left(\hat{\lambda }\right)=\frac{1\;-\;e^{-\hat{\lambda }}}{N(e^{\hat{\lambda }}\;-\;1\;-\;\hat{\gamma })}(\frac{\hat{\gamma }}{2}(e^{\hat{\lambda }}\;+\;1)-\frac{e^{\hat{\lambda }}\;-\;1}{e^{\hat{\lambda }}\;-\;1\;-\;\hat{\gamma }}\sum_{1\leq i<j\leq n}\;(e^{\hat{\lambda }{\hat{p}}_{i}}\;-\;1)(e^{\hat{\lambda }{\hat{p}}_{j}}\;-\;1)\;\;). \end{array}$$ (20b)

*The bias-corrected estimate of the lineage frequency p_k_ is*

$$\begin{array}{c} {\hat{p}}_{k}^{\left(bc\right)}={\hat{p}}_{k}-B_{\hat{\theta }}\left({\hat{p}}_{k}\right), \end{array}$$ (20c)

*where*

$$\begin{array}{c} \begin{array}{cc} B_{\hat{\theta }}\left({\hat{p}}_{k}\right)= & \frac{1\;-\;e^{-\hat{\lambda }}}{2\hat{\lambda }N(e^{\hat{\lambda }}\;-\;1\;-\;\hat{\gamma })}(\;\frac{1}{\hat{\lambda }}(e^{\hat{\lambda }{\hat{p}}_{k}}\;-\;1\;-{\hat{p}}_{k}\hat{\gamma })(\hat{\lambda }\left(e^{\hat{\lambda }}\;+\;1\right)\;-\;2\left(e^{\hat{\lambda }}\;-\;1\right))\;+\;(e^{\hat{\lambda }{\hat{p}}_{k}}\;-\;1{)}^{2}\\ & +\frac{e^{\hat{\lambda }}\;-\;1}{e^{\hat{\lambda }}\;-\;1\;-\;\hat{\gamma }}(\hat{\gamma }({\hat{p}}_{k}\hat{\gamma }\;-\;e^{\hat{\lambda }{\hat{p}}_{k}}\;+\;1)+(\frac{e^{\hat{\lambda }{\hat{p}}_{k}}\;-\;1}{e^{\hat{\lambda }}\;-\;1}\;-\;{\hat{p}}_{k})\sum_{j=1}^{n}(e^{\hat{\lambda }{\hat{p}}_{j}}\;-\;1{)}^{2}\;)\;\;). \end{array} \end{array}$$ (20d)

*In the above* $\hat{\gamma }=\sum_{k=1}^{n}e^{\hat{\lambda }{\hat{p}}_{k}}-1$, *and* $\hat{\theta }=\left(\hat{\lambda },{\hat{p}}_{1},\ldots ,{\hat{p}}_{n}\right)$ *is given by* (9).

We explore the properties of the bias-corrected estimate in a systematic simulation study below. Before we do so, we make some heuristic changes to the BCMLE in the next section.

### Heuristically improved bias corrections

The MLE (9) is only meaningful for regular data. (Remember for pathological data the MLE does not exist or lies on the boundary of the admissible parameter space, where the asymptotic properties of the MLE do not hold.) Therefore, also the bias correction is only meaningful for regular data. Since the general derivation of the second-order bias correction (given in [31]) is not conditioned on regular data, it corrects the actual bias by too much. We therefore heuristically adjust the bias correction by multiplying it with the probability of observing regular data. This applies only to the MOI estimate. The lineage frequencies are a probability distribution normalized by 1 and cannot be multiplied by a constant.

In [17] the probability of observing pathological data was derived. It is given by

$$\begin{array}{c} q≔\frac{1}{{\left(1\;-\;e^{-\lambda }\right)}^{N}}(\;1\;-\;\prod_{j=1}^{n}\;(1\;-{\left(1\;-\;e^{-\lambda p_{j}}\right)}^{N}\;)\;\;)\;+\;(\sum_{j=1}^{n}\frac{e^{\lambda p_{j}}\;-\;1}{e^{\lambda }\;-\;1}{)}^{N}\;\;-\;\sum_{j=1}^{n}\;\;(\frac{e^{\lambda p_{j}}\;-\;1}{e^{\lambda }\;-\;1}{)}^{N}. \end{array}$$ (21)

The probability of observing regular data is then given by 1 − *q*. This involves the true unknown parameters. However, we can use the MLE or even the BCMLE as a plug-in estimate to adjust the BCMLE as follows:

**Remark 3**. *Assuming regular data, a heuristically adjusted BCMLE of the MOI parameter is*

$$\begin{array}{c} {\hat{\lambda }}^{\left(hbc1\right)}=\left(1-\hat{q}\right){\hat{\lambda }}^{\left(bc\right)}, \end{array}$$ (22a)

*where*

$$\begin{array}{c} \hat{q}≔\;\frac{1}{{\left(1\;-\;e^{-\hat{\lambda }}\right)}^{N}}(\;1\;-\;\prod_{j=1}^{n}\;(\;1\;-\;{\left(1\;-\;e^{-\hat{\lambda }{\hat{p}}_{j}}\right)}^{N}\;)\;\;)\;+\;(\sum_{j=1}^{n}\frac{e^{\hat{\lambda }{\hat{p}}_{j}}-1}{e^{\hat{\lambda }}\;-\;1}{)}^{N}\;\;\;-\;\sum_{j=1}^{n}\;\;(\frac{e^{\hat{\lambda }{\hat{p}}_{j}}\;-\;1}{e^{\hat{\lambda }}\;-\;1}{)}^{N}. \end{array}$$ (22b)

*The lineage frequency estimates are not adjusted, i.e*.,

$$\begin{array}{c} {\hat{p}}_{k}^{\left(hbc1\right)}={\hat{p}}_{k}^{\left(bc\right)}, \end{array}$$ (22c)

*for k* = 1, …, *n*. *We refer to* (22) *as the first version of a heuristically adjusted BCMLE (HBCMLE1)*.

The heuristic adjustment can also be done in a two-step procedure, i.e., first calculating the BCMLE according to Result 2, and then use this estimate as a plug-in for the heuristic adjustment. We obtain the following.

**Remark 4**. *As an alternative to the HBCMLE1 given in Remark 3 we can define*

$${\hat{\lambda }}^{\left(hbc2\right)}=\left(1-{\hat{q}}^{\left(bc\right)}\right)\left(\hat{\lambda }-B_{{\hat{\theta }}^{\left(bc\right)}}\left(\hat{\lambda }\right)\right),$$ (23a)

*and*

$${\hat{p}}_{k}^{\left(hbc2\right)}={\hat{p}}_{k}-B_{{\hat{\theta }}^{\left(bc\right)}}\left({\hat{p}}_{k}\right),$$ (23b)

*where* ${\hat{q}}^{\left(bc\right)}$ *is given by* (22b) *with* $\hat{\lambda }$ *and* ${\hat{p}}_{j}$ *replaced by* ${\hat{\lambda }}^{\left(bc\right)}$ *and* ${\hat{p}}_{j}^{\left(bc\right)}$, *respectively*. *Similarly*, $B_{{\hat{\theta }}^{\left(bc\right)}}\left(\hat{\lambda }\right)$ *and* $B_{{\hat{\theta }}^{\left(bc\right)}}\left({\hat{p}}_{k}\right)$ *are given by* (20b) *and* (20d) *with* $\hat{\lambda }$ *and* ${\hat{p}}_{j}$ *replaced by* ${\hat{\lambda }}^{\left(bc\right)}$ *and* ${\hat{p}}_{j}^{\left(bc\right)}$, *respectively*. *This estimator is referred to as HBCMLE2*.

Many other heuristic adjustments are possible, i.e., corrected estimates do not have to be adjusted by multiplying with a constant as in Remarks 3 and 4. We present only one possibility, whose properties will be numerically investigated in the following.

**Remark 5**. *Assuming regular data, a heuristically adjusted BCMLE of the MOI parameter is*

$${\hat{\lambda }}^{\left(hbc3\right)}=\left(1-{\hat{q}}^{\left(bc\right)}\right)\hat{\lambda }-B_{\hat{\theta }}\left(\hat{\lambda }\right),$$ (24a)

*where* ${\hat{q}}^{\left(bc\right)}$ *is calculated as in Remark 4*. *The lineage-frequency estimates are not adjusted, i.e*.,

$${\hat{p}}_{k}^{\left(hbc3\right)}={\hat{p}}_{k}^{\left(bc\right)},$$ (24b)

*for k* = 1, …, *n*. *We refer to* (24) *as the third version of a heuristically adjusted BCMLE (HBCMLE3)*.

Next, we compare the performance of the MLE and bias-corrected MLEs in a systematic numerical study. The simulations are described in S1 Appendix (Simulation study) with the MLE.

## Finite sample properties

For large samples no bias correction is necessary, because the maximum-likelihood estimate has the usual desirable asymptotic properties (asymptotically unbiased, efficient, strongly consistent) as shown in [17]. However, for small samples it can be substantially biased. In such case a bias correction can substantially improve he estimator.

### Bias of the original estimator—Demand for bias correction

The bias of the original estimator was studied in detail in [17]. On average, the estimator tends to overestimate the true parameter. In relative terms, bias is highest in areas of either low- or high transmission, i.e., for either λ < 0.2 (or *ψ* < 1.1) or λ > 1.5 (or *ψ* > 1.9). The relative bias as a function of *ψ* has a bathtub shape, with a strong decline for small values. The reasons are as follows. The Poisson parameter λ is positive. Hence, it has a lower but no upper bound. If the true parameter λ is small, in a dataset samples with several lineages present will be occasionally over-represented. This leads to substantial overestimates—ML estimators are known to be sensitive to outliers (cf. [48] Section 5.5). (Note, when defining the bias by the median rather than by the mean, the estimator would be substantially less biased, see [17]). In areas of high transmission (large true λ), the number of samples containing several lineages varies substantially in datasets. The reason is that the Poisson distribution has mean and variance λ, i.e., for larger λ, the variance of MOI is larger. Thus, frequently samples harboring several lineages are either over- or under-represented in datasets. In the former case the Poisson parameter λ is substantially overestimated, while in the latter case it is underestimated—however these estimates have a lower bound. In principle sample size can be increased to avoid bias. This is easier in high-transmission areas. However, in absolute terms bias will be substantial compared with low-transmission areas. Importantly, bias increases substantially if the lineage frequency distributions are skewed. The reason is that super-infections with the predominant lineages are common, which give no (or little) evidence of multiple infections. Thus, the number of distinct observable lineages in a sample does not adequately reflect the actual (unobservable) number of super-infections. Since is it often impossible to increase the sample size sufficiently, bias corrections should be applied to the estimator.

### Assessing the estimator’s bias

Since there is no closed solution for the MLE, it is impossible to assess its bias analytically for a given sample size. The estimator’s bias for a given true parameter ***θ*** needs to be numerically estimated by, e.g., Monte Carlo simulations. In [17] the finite sample properties of the MLE in terms of bias and variance were investigated by a comprehensive simulation study.

Also the finite sample properties of the bias corrected versions of the estimator need to be studied numerically. Importantly, the bias correction applied here was derived from a general asymptotic approximation, because the MLE’s true bias cannot be derived analytically. Hence, also the bias correction is not perfect for small sample sizes. Therefore, it is important to study the properties of the bias correction for ‘small’ sample sizes and its improvements compared to the original estimator.

Here, we compare the bias-corrected estimator and the original one. Using numerical simulations, we systematically estimate the bias and variance of the BCMLE and its heuristically adjusted variants for representative sets of model parameters under different sample sizes. Furthermore, we study the estimates’ robustness to model violations, by assuming that MOI follows a negative binomial distribution (corresponding to an over-dispersed Poisson distribution). The numerical investigations were implemented in R [49]. The heart of the code (the code to generate simulated data, and the implementation of the estimators and all functions) is available at GitHub (https://github.com/Maths-against-Malaria/MOI-Bias-correction.git, https://doi.org/10.5281/zenodo.5119425). A detailed description of the simulation study is given in S1 Appendix (Simulation study).

### Estimates of the MOI parameter

In this section, we investigate the performance of the BCMLE and the heuristically adjusted estimates of average MOI *ψ* in terms of accuracy (relative bias) and precision (coefficient of variation). The Poisson parameter λ ranges from 0.1 to 0.5 (*ψ* ranges approximately between 1.05 and 1.27) in low-transmission areas, from 0.5 to 1.5 (*ψ* between 1.27 and 1.9) in intermediate-transmission areas, and 1.5 to 2 (*ψ* between 1.9 and 2.4) in high-transmission areas. We refer to *ψ* in this ranges as small, intermediate, and large.

#### The bias corrected maximum likelihood estimate—BCMLE

**Fig 2:**
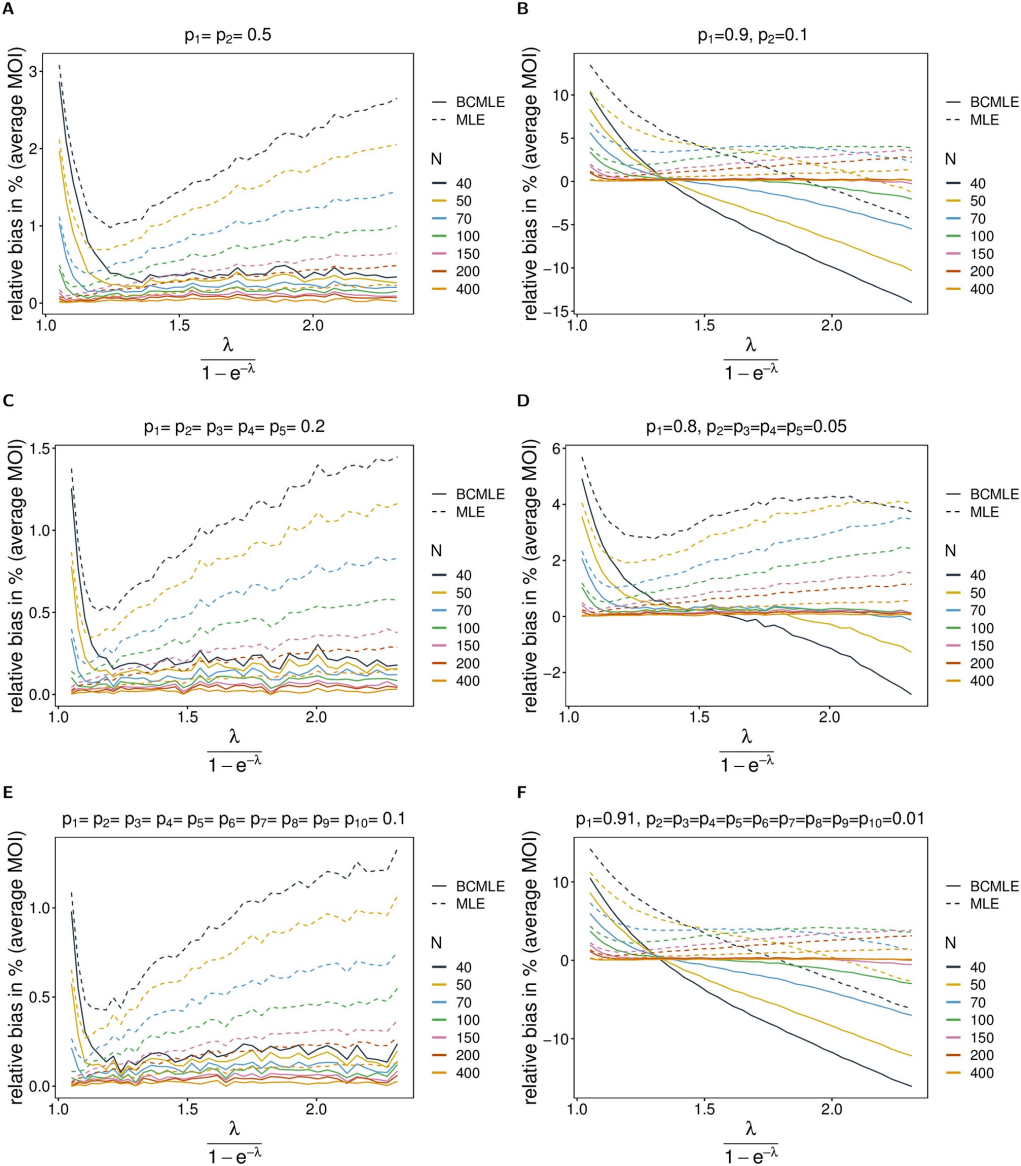
Bias of MOI estimates. The figure shows the relative bias in % of the BCMLE ${\hat{\psi }}^{\text{(bc)}}$ (solid lines) and MLE $\hat{\psi }$ (dashed lines) as a function of the true parameter *ψ* based on simulated data created by the conditional Poisson model. Each panel assumes a different lineage-frequency distribution ***p*** shown at the top of each panel. Each colored line corresponds to a different sample size *N*.

*Bias*. The bias correction noticeably improves the MLE. For any sample size, the BCMLE of the average MOI, i.e., ${\hat{\psi }}^{\text{(bc)}}=\frac{{\hat{\lambda }}^{\text{(bc)}}}{1-e^{-{\hat{\lambda }}^{\text{(bc)}}}}$, has bias less than 0.5% for intermediate and large *ψ*, and balanced or slightly-skewed lineage-frequency distributions (with frequency of dominant lineage < 0.8; see Fig 2). In this case, the bias correction removes bias efficiently for intermediate and large *ψ*. Although, the MLE is slightly more biased for small sample sizes *N*, the bias correction results in low bias almost independent of sample size *N* (Fig 2, see also Section Relative bias in %, S2 Appendix for a comprehensive range of parameters). The reason is that $B_{\theta }\left(\hat{\lambda }\right)$ (see Eq 19a) is of order *N*^−1^ and the estimator is corrected more for smaller sample sizes. Concluding, for intermediate and high average MOI, the BCMLE is accurate independently of sample size. However, sample size needs to be adjusted to meet a desired precision goal, i.e., increasing sample size lowers the estimator’s variance to the desired level.

The bias is not noticeably corrected for very small *ψ*. In this case, the MLE and the BCMLE carry almost a similar amount of bias. The reason is, the bias is small due to the scale of the parameter and large only in relative terms. Furthermore, the relative bias is derived conditioned on regular data, whereas the bias correction does not condition on such data. This fact motivated us to consider heuristically adjusted estimators (see Analytical results, Heuristically improved bias corrections). The occurrence of pathological data is substantial for small average MOI (cf. Section Probability of regular data, S2 Appendix).

For highly-skewed lineage-frequency distributions (characterized by only one predominant lineage at high frequency, while all other lineages have low frequency), the bias correction does not improve the estimates (see Fig 2B, 2D and 2F). In this case, the BCMLE is noticeably biased if the sample size is small (*N* < 100) except for intermediate *ψ* (but notably depends on a combination of the number of lineages and the skewness—cf. Fig 2D with Fig 2B and 2F).

Because $B_{\theta }\left(\hat{\lambda }\right)$ increases with increasing skewness of the lineage-frequency distribution, the bias of the MLE is corrected more strongly as skewness increases. This is more evident if *N* < 100. In fact, the bias correction may result in negative bias (see Fig 2F). This is the case for highly-skewed lineage-frequency distributions (if the dominant lineage has frequency > 0.8). The BCMLE tends to be underestimated for large *ψ* (*ψ* > 1.5). In this parameter range observations are not very informative, as multiple infections, which contain lineages other than the predominant one, are rare. However, if the average MOI is small to intermediate (*ψ* < 1.5) or sample size sufficiently large (*N* > 100), the BCMLE performs reasonably better than the MLE for these lineage-frequency distributions (see Fig 2). Notably, if average MOI is large, transmission is high, and it is realistic to collect sufficiently large datasets (*N* > 100).

**Fig 3:**
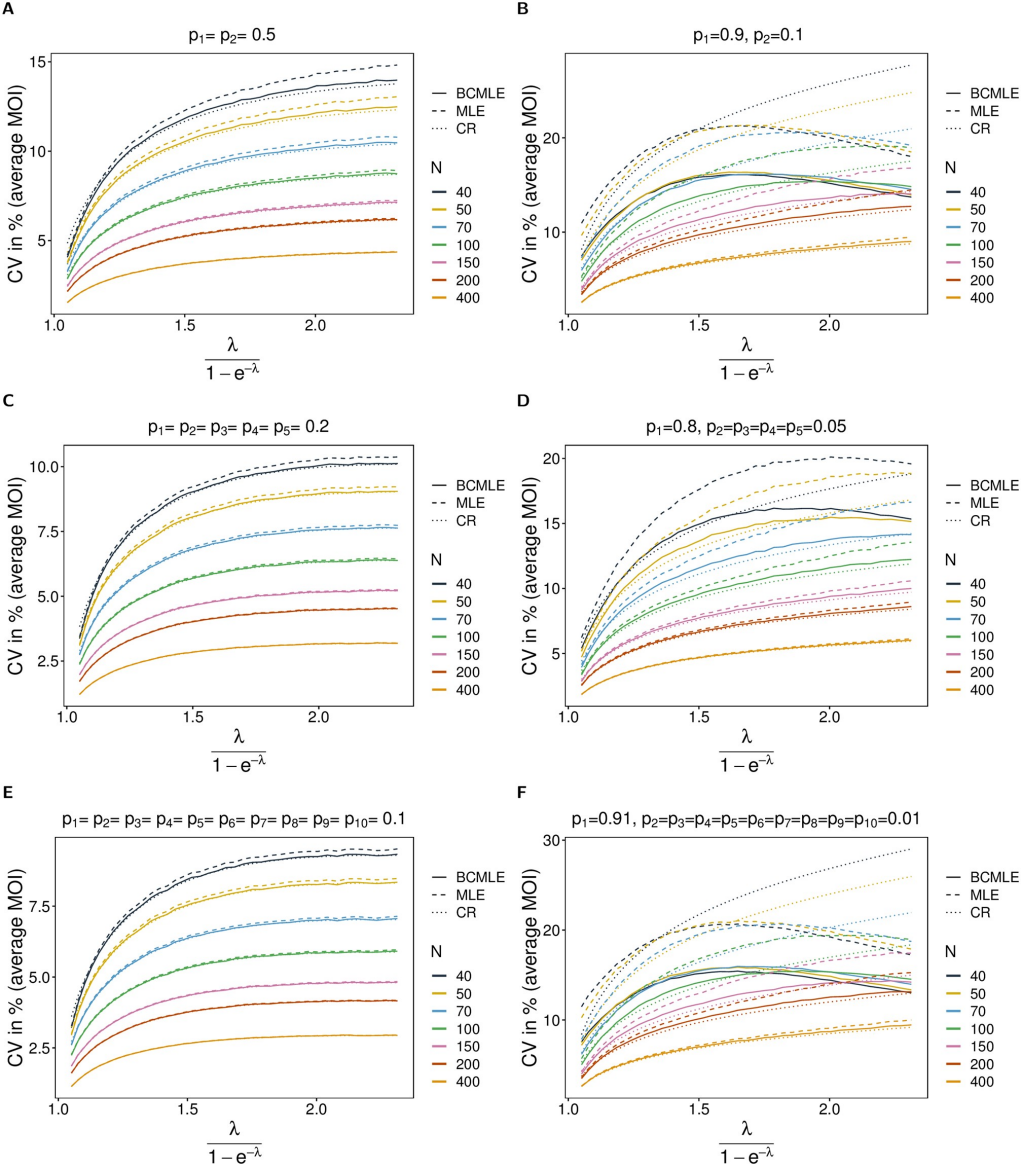
Variance of MOI estimates. Similar to Fig 2 but for the coefficient of variation in %. The dotted lines are the respective predictions based on the Cramér-Rao lower bounds.

*Coefficient of variation*. For all combinations of model parameters, the BCMLE has a smaller coefficient of variation (CV) than the MLE (Fig 3, see also Section Relative bias in %, S2 Appendix for a comprehensive range of parameters). Not surprisingly, for large sample size the CVs of both estimators approximate the theoretical prediction (the square root of the Cramér-Rao lower bound divided by the true value of *ψ*) very well. For smaller sample size (*N* < 200) the CV of the BCMLE is better approximated by its theoretical prediction than the MLE. For small *ψ* the CV of the BCMLE is always smaller than its theoretical prediction, while that of the MLE can get larger for skewed lineage-frequency distributions (frequency of the predominant lineage >0.8; Fig 3B, 3D and 3F). For intermediate and large *ψ* and skewed distributions the CVs of the BCMLE and MLE are smaller than their theoretical predictions. Particularly, the CV of the BCMLE can be substantially smaller. The reason is that most samples in this parameter range do not adequately reflect the true MOI because super-infections with the predominant lineage are likely. This results in frequent underestimates (and rare overestimates), and hence reduced variation in the estimates. For highly-skewed lineage-frequency distributions (predominant frequency >0.9) the BCMLE has substantially reduced variance (3 to 5 percentage points) compared with the MLE and the theoretical prediction for intermediate and large *ψ* (see Fig 3B and 3F).

Therefore, the BCMLE is an (almost) efficient estimator for *ψ* in a finite sample sense—except for very skewed lineage frequencies, large average MOI and small sample size. In this parameter range, the BCMLE has noticeably lower variance than the MLE. Although this is desirable, this will not per se result in a better performance of the BCMLE in terms of the mean squared error. Remember, for large *ψ* the BCMLE has a substantial negative bias. Anyhow, the BCMLE overall outperforms the MLE.

**Fig 4:**
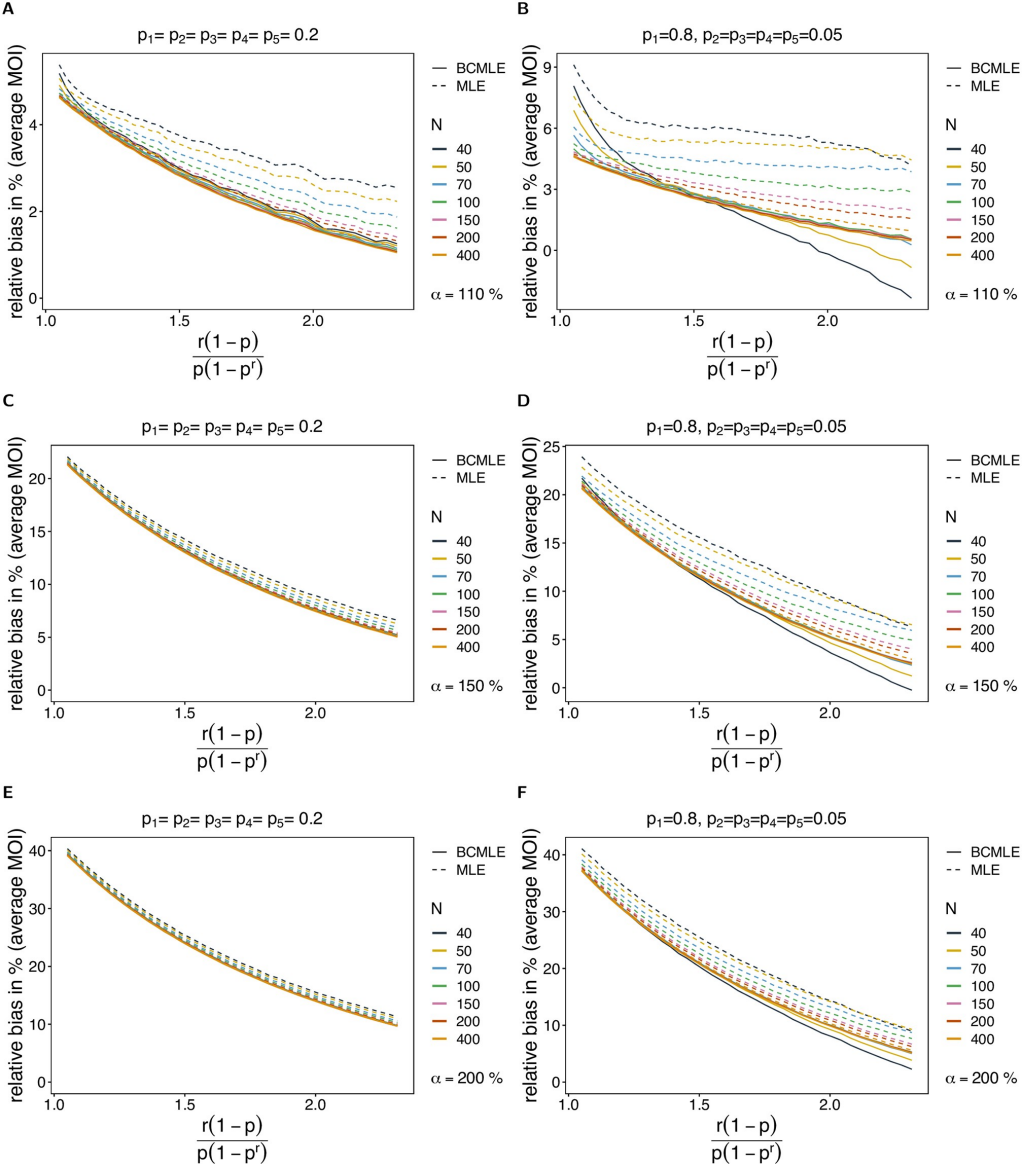
Robustness of MOI estimates against model violations. The figure shows the bias of the BCMLE ${\hat{\psi }}^{\text{(bc)}}$ (solid lines) and MLE $\hat{\psi }$ (dashed lines) in % as a function of the true parameter $\psi =\frac{r(1-p)}{p(1-p^{r})}$. The datasets are generated from the conditional negative binomial model whereas the estimates are derived from the conditional Poisson model. The panels in different rows correspond to different levels of overdispersion indicated by *α*. Panels on the left and right assume a different lineage-frequency distributions ***p*** shown at the top of each panel. Line colors correspond to a different sample sizes (*N*).

*Model violations*. The model violation has a noticeable effect on the MLE’s performance (see Section 5, S2 Appendix for results for a comprehensive range of parameters). The MLE largely overestimates the true *ψ* if overdispersion is added to the data. While bias is still small for low amounts of overdispersion (10% overdispersion compared to the mean; Fig 4A and 4B) and acceptable for overdispersion as much as 50% (Fig 4C and 4D), it gets substantial for high overdispersion (> 100%; Fig 4E and 4F).

Bias increases mainly for small true *ψ*. With overdispersion, it is more likely to observe datasets that contain samples with multiple lineages. For such datasets MOI will be substantially overestimated.

Under the alternative model, the bias correction is not as effective as it is under the original model, and the BCMLE also carries a relatively large bias if overdispersion is high. However, the bias correction performs independently of the amount of overdispersion. (This is reasonable since the second-order bias is calculated under the original model which assumes that the true mean and variance are equal). If MOI follows a negative binomial distribution, the BCMLE performs better than the MLE by having typically 1 to 2 percent points less bias, and smaller CV than the MLE for balanced or slightly-skewed lineage-frequency distributions.

**Fig 5:**
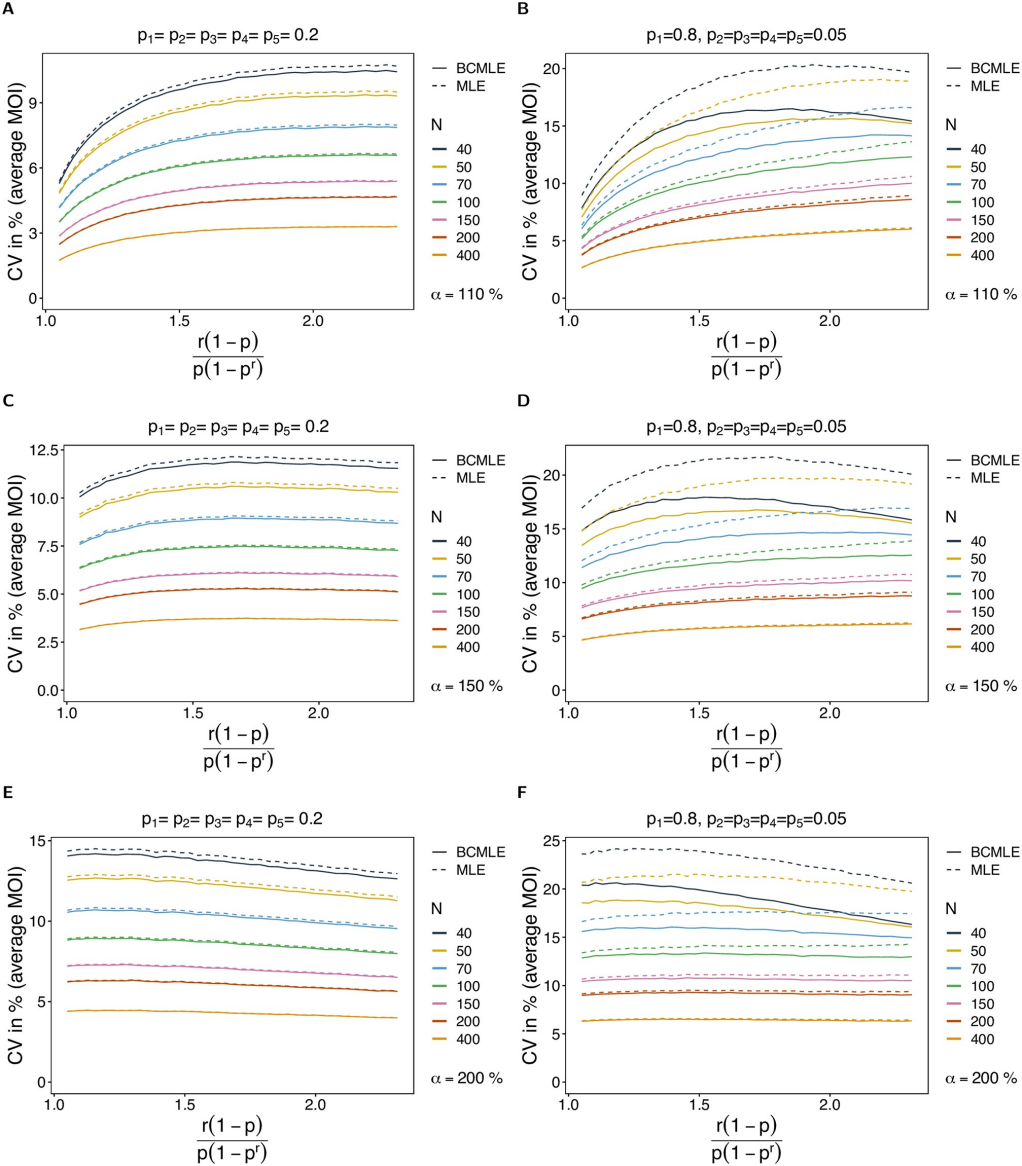
Variance if MOI estimates under model violations. Similar to Fig 4 but for the coefficient of variation in %.

The model violation also affects the CV noticeably. The CV increases mainly for small and intermediate *ψ*, whereas the increase is moderate for large *ψ*. This is intuitive, because for small *ψ*, the true parameter is sometimes substantially overestimated due to the overrepresentation of samples with multiple lineages in some datasets. This increases the variance of the estimator substantially. The higher overdispersion, the larger the increase in the CV for small and intermediate *ψ* (compare Fig 5A with Fig 5C and 5E, and Fig 5B with Fig 5D and 5F). The BCMLE has a slightly lower CV than the original estimator (compare Fig 3C and 3D with Fig 5).

#### Heuristically adjusted estimates

**Fig 6:**
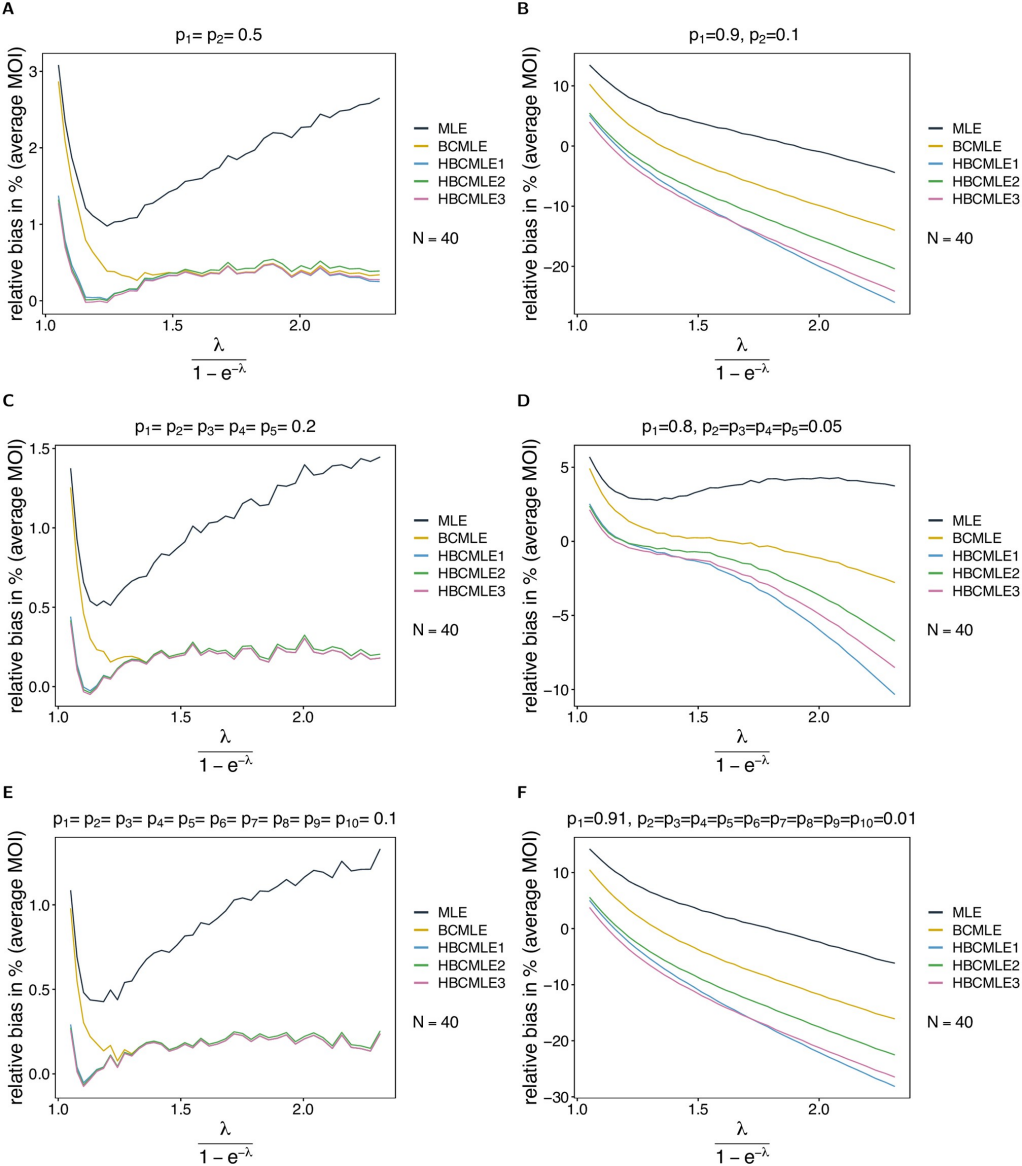
Bias of heuristically adjusted MOI estimators. Shown is the relative bias in % of the heuristically adjusted estimators (HBCMLE1—${\hat{\psi }}^{\left(hbc1\right)}$, HBCMLE2—${\hat{\psi }}^{\left(hbc2\right)}$, HBCMLE3—${\hat{\psi }}^{\left(hbc3\right)}$) along with the relative bias in % of the MLE $\hat{\psi }$ and BCMLE ${\hat{\psi }}^{\text{(bc)}}$ as a function of the true parameter *ψ* based on simulated data created by the conditional Poisson model. Each panel assumes a different lineage-frequency distribution ***p*** shown at the top of each panel. Colors correspond to different estimators. The relative bias in each panel is derived from *S* = 100, 000 randomly generated datasets of sample size 40.

The occurrence of pathological data, for which the MLE does not exist (or is degenerate), is not properly incorporated in the general derivation of the bias correction. To resolve this approximately, we proposed several heuristic adjustments to the BCMLE, which essentially conditions the correction on the occurrence of regular data. In fact, bias can change substantially (see Fig 6). The amount of adjustment depends on the likelihood of observing pathological data (*q*; cf. also Section 3 in S2 Appendix). For large sample size pathological data is unlikely and all estimates are similar, which is not surprising because they are asymptotically equivalent. For small sample size pathological data can be common, depending on the true parameters.

For small *ψ*, small datasets are often pathological. The heuristic adjustments properly down-correct bias of the estimators. The heuristic adjustments are stronger for more skewed lineage-frequency distributions because pathological data is more likely. This is a desirable property of the heuristic adjustments. Hence, they are preferable compared to the BCMLE. All heuristic adjustments are similar (see Fig 6).

For intermediate *ψ*, degenerate data becomes less likely and the heuristic adjustments are less relevant. This is particularly true for balanced frequency distributions. In this case the heuristic adjustments do not change the BCMLE noticeably (see Fig 6A, 6C and 6E). For skewed distributions, pathological data is still likely and the heuristic adjustments tend to improve the BCMLE (Fig 6D). However, the adjustments are too strong for extremely skewed distributions (Fig 6B and 6F), and the estimators substantially underestimate the true parameter. There is still no clear difference between the adjusted versions of the estimator.

For large *ψ*, similar observations are made as for intermediate *ψ*. However, for skewed distributions the likelihood of pathological data increases substantially, as the predominant lineage is likely to occur in all samples. The result is a substantial underestimation of the true parameter. Also differences between the heuristically corrected versions become obvious (see Fig 6B, 6D and 6F). The HBCMLE1 underestimates the most, and the HBCMLE2 the least. Hence, the HBCMLE2 is the most desirable estimator.

**Fig 7:**
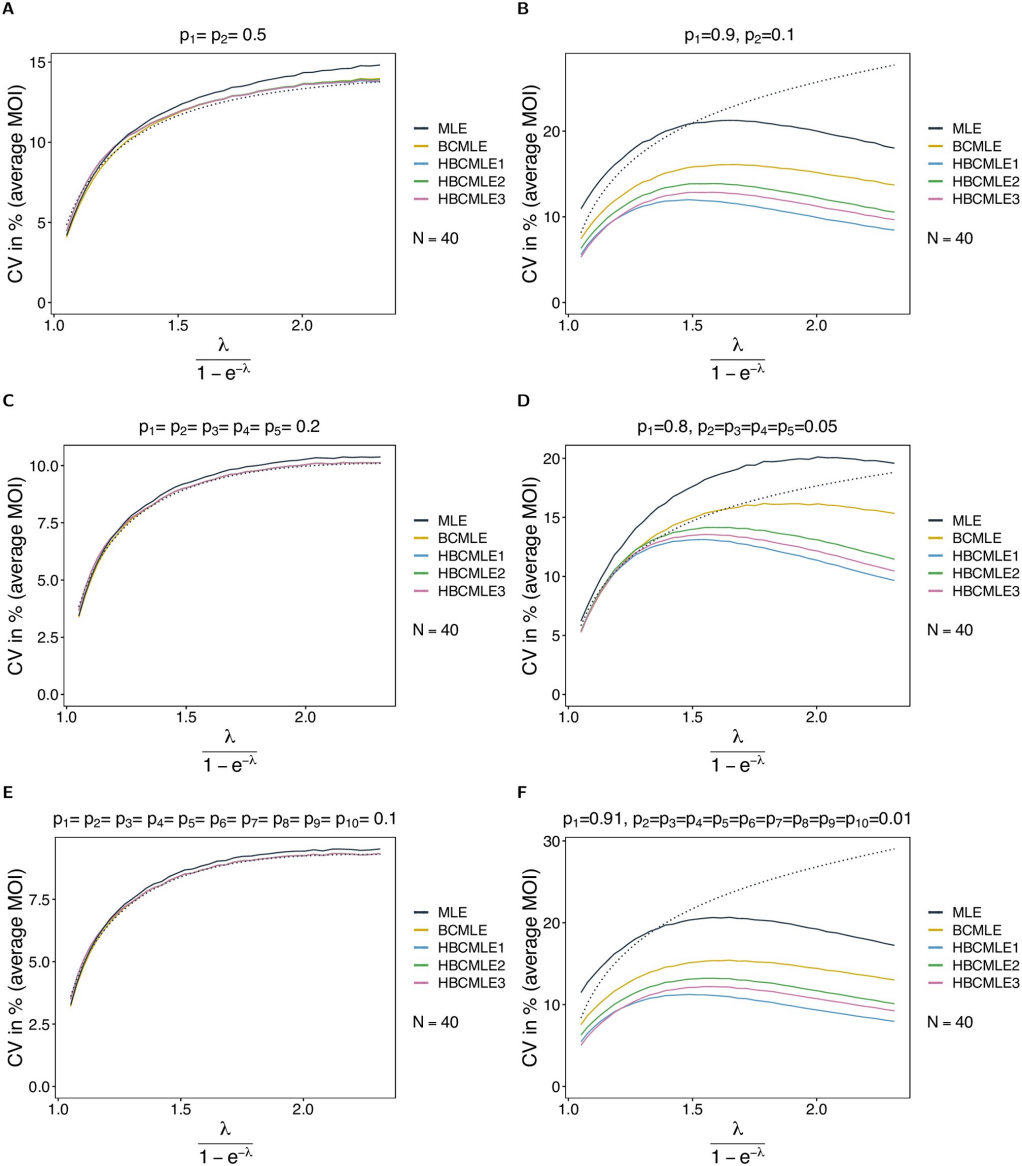
Variance of heuristically adjusted MOI estimators. Similar to Fig 6 but for the coefficient of variation in %. The dotted lines are the respective predictions based on the Cramér-Rao lower bounds.

For balanced lineage-frequency distributions the MLE’s variance was close to the Cramér-Rao lower bound, which coincided with the BCMLE’s variance. Thus, not surprisingly the heuristic adjustments cannot further improve the estimator (see Fig 7A, 7C and 7E). These are hence almost unbiased and their variance coincides with the minimal variance of an unbiased estimator. For skewed lineage-frequency distributions the adjustments lead to a reduction in variance compared with the BCMLE. In fact, the variance is lower than the Cramér-Rao lower bound (note that the estimators are also clearly biased in this case). The HBCMLE1 has the lowest and the HBCMLE2 the highest variance of the adjusted estimators. These differences vanish as sample size increases.

Model violations have the same effect for the heuristically adjusted estimators as for the BCMLE. This is because, similar to $B_{\theta }\left(\hat{\lambda }\right)$, the probability of regular data is derived under the original model and remains independent of the level of overdispersion. However, in the case in which skewness is high, they carry less negative bias under the model violation compared to the original model. This is because, the average MOI is largely overestimated. Similarly, the HBCMLE2 is preferred over the other two adjusted estimators (see Section 5.3, S2 Appendix).

Overall, the HBCMLE2 performs best. In the assessment of bias and variance one needs to consider sample size. In practice, larger sample sizes are feasible for larger *ψ* as disease prevalence is higher. Therefore, the bias correction for small *ψ* is more important than that for large *ψ* (see Section 2, S2 Appendix for comprehensive results).

### Lineage frequencies

Unlike for the average MOI, the MLE for lineage frequencies is almost unbiased [17]. Thus, not surprisingly, the bias correction has little influence on bias of lineage-frequency estimates, particularly for balanced and slightly-skewed lineage-frequency distributions (see S1A Fig; see also Section Relative bias in % in S2 Appendix).

For skewed frequency distributions and small average MOI, the MLE tends to overestimate rare lineages in small datasets. The reason is that rare lineages are easily over-represented. For instance, if *ψ* = 1 (only single infections) a lineage with frequency 10% has an estimated frequency of 12.5% if it occurs in five samples in a dataset of size *N* = 40—an overestimate by 25%. If a lineage with frequency 1% is found in one sample, its frequency is estimated to be 4%—a relative overestimate of 300%. For large *ψ*, predominant frequencies are overestimated while minor frequencies are underestimated. The reason is the over-representation of predominant lineages if MOI is high. For skewed frequency distributions the bias correction successfully removes bias almost independently of sample size. Bias remains only for small *ψ*, but vanishes as sample size increases (see S1B Fig). The MLE is less accurate (i.e. more biased) for lineages with low frequency because uncertainty is higher for them as datasets harbor less information about them. This fact is adequately compensated by the bias correction.

Only for highly-skewed frequency distributions, the bias corrections over-correct and yield inferior estimates compared with the MLE (see Section 4.1 in S2 Appendix).

Regarding the heuristic adjustments, only HBCMLE2 adjusts the frequency estimates. This adjustment is negligible (see S2 Fig).

While frequency estimates are almost unbiased, they have substantial variance in terms of the CV. The variance of the estimates decreases substantially with increasing sample size. Not surprisingly, estimates of small frequencies have high variance. The variance of the estimates is not affected by the bias correction (see S3 Fig).

Measuring the performance of frequency estimates by the Euclidean distance or the Kullback-Leibler information captures bias and variance, indicates overall good performance of the estimators (cf. [17]). The bias correction does not show a significant improvement (see S4 Fig).

## Discussion

The importance of MOI (or complexity of infection) in malaria and other infectious diseases is increasingly being recognized. It is an important metric that scales with transmission intensities [33] and hence allows to monitor the efficiency of control interventions on temporal and spatial scales. Moreover, it mediates the relationship between lineage frequencies and prevalence, e.g., of drug-resistance associated mutations or HRP2/3 gene deletions in malaria. While a lineage’s frequency refers to its relative abundance in the pathogen population, its prevalence refers to the probability of observing the lineage in an infection. The difference, between frequency and prevalence, was argued to be particularly important in the context of seasonal malaria [5]. Despite its widespread applications in empirical studies, the definition of MOI is still ambiguous. In fact, MOI is often derived by heuristic estimates. Whereas such approximations have been useful, these estimators lack a statistical foundation that allows to evaluate their quality. Hence, there is still a gap between empirical applications and theory. With data being generated more systematically and molecular/genetic studies becoming more widespread, there is a high demand to base estimates on a solid statistical framework.

The statistical framework used by [16] allows estimating MOI and lineage frequencies based on a concise statistical framework by maximum likelihood—and thus also by Bayesian methods. The method is appropriate for lineages characterized by a single molecular marker (microsatellite marker, SNP) or haplotypes in a short non-recombining region. Importantly MOI is defined here as the number of super-infections, which is an unobservable quantity. Notably, the interpretation of the statistical model is more flexible and it can be interpreted as modelling super- and co-infections (cf. Model background). In empirical studies MOI is often defined differently. In section Alternative definitions of MOI it is explained how these estimates are simply derived from the estimator proposed here. The large- and finite-sample properties of the estimator have been thoroughly studied [17]. In fact it has been shown, that the estimator satisfies the usual desirable (asymptotic) properties, e.g., asymptotic unbiasedness, strong consistency, efficiency, sufficiency [17]. However, the estimator, particularly for MOI is biased if the sample size is small. This is particularly true if average MOI is low, i.e., in endemic areas of low transmission, where large sample sizes are often infeasible.

In areas of high transmission, bias can be reduced by increasing sample size, however only in relative terms. In absolute terms bias will be still larger than in low-transmission settings. Furthermore, another source of bias is skewed lineage frequency distributions. Neutral molecular markers such as microsatellite markers at chromosomes without known genes under selection seem most appropriate. In general, MOI can be estimated from any molecular marker. In case of malaria, microsatellite markers in the vicinity of genes conferring drug resistance, might be strongly affected by genetic hitchhiking and thus have very skewed frequency distributions (see [5, 50]). A similar logic applies to PCR-RFLP of msp genes, which have been repeatedly found to be under selection [51]. Applying bias corrections in such situations is important to improve the quality of the estimates. Similarly, when aiming to compare recent with retrospective data, or when aiming to regularly estimate the frequency distributions and MOI in the course of a national malaria control program sample, researchers might be forced to work with relatively small samples, in which cases bias corrections are essential.

To compensate for the systematic errors of the maximum-likelihood method in small samples, we derived a bias-corrected version of the original estimator. More precisely, we followed the approach of [31] to derive a bias-corrected estimate, which has bias of order $O(N^{-2})$. To evaluate the performance of the bias-corrected estimator for MOI and lineage frequencies, we conducted a systematic numerical study. We investigated the accuracy (bias) and precision (variance) of the bias-corrected estimator and heuristically adjusted variants for a comprehensive set of parameters.

The bias-corrected estimator clearly outperforms the original estimator for small sample sizes (*N* ≤ 75). For intermediate sample size (70 ≤ *N* ≤ 150) the bias correction still yields relevant improvements. For larger sample sizes the need for a bias correction becomes less relevant. For all sample sizes, the corrected estimator is (almost) unbiased, except for extreme parameter values (very low or high MOI and skewed lineage-frequency distributions). Although the bias-corrected estimator is still biased for extreme parameters, it substantially improves the original estimator. The case of low MOI and skewed lineage-frequency distributions is particularly relevant—e.g., in the context of mutations conferring drug resistance in malaria in areas of low transmission (typically skewed frequency distributions occur at markers linked to positions conferring drug-resistance due to genetic hitchhiking [50, 52]). The bias correction also reduces the estimator’s variance, namely, it matches the minimum variance for an unbiased estimator, i.e., the Cramér-Rao lower bound, except for extreme parameters. For very skewed frequency distributions, the variance of the corrected estimator is even lower than the Cramér-Rao lower bound—this is possible because the estimator is biased.

In areas of low and high transmission the MOI estimates are almost biased (cf. [17]). In such situations, the bias correction substantially improves the estimates. (The improvements for intermediate transmission—intermediate λ—are less noticeable since the original estimates are not as biased in this case.)

We further studied the robustness of the estimators with regard to model violations. Namely, when assuming that MOI is over-dispersed, i.e., it follows a negative binomial distribution rather than a Poisson distribution, the estimators still perform reasonably if overdispersion is not too strong (up to 50% overdispersion). Notice that mosquito biting rates follow a negative binomial distribution [33]. However, not each bite is infectious, and not each infectious bite leads to an infection. The resulting number of infective (infectious bites leading to infection) will still be binomially distributed but overdispersion is reduced to a level that justifies the assumption of Poisson-distributed MOI [5].

The heuristic adjustments to the bias-corrected estimator further reduce bias and variance, particularly the estimator HBCMLE2 (23). The corrected estimators are (almost) unbiased and have variance almost identical to the Cramér-Rao lower bound. This suggests that further improvements are not possible without adding additional information, i.e., increasing sample size or extending the statistical model to include further data (information).

Notably, there exist alternative methods to reduce the bias of estimators. Straightforward, but computationally extensive methods include parametric and non-parametric bootstrap bias corrections [53]. The performance of such methods needs to be explored. Both methods will likely not perform well if the data set does not properly reflect the population. This is particularly true for small sample size for which the bias correction is necessary. The advantage of the method used here, is that the bias correction is derived from the general model, not a particular dataset. Only the parameter estimates from the data are used as plug-ins.

Our comprehensive simulation study serves also as a lookup-table for study design to determine an appropriate sample size to achieve certain performance goals for the estimator. If there is prior knowledge of the true parameter range, the plots in S2 Appendix help to determine the proper sample size to achieve a given accuracy and precision of the estimators.

The maximum-likelihood method of [16] to derive MOI, as well as lineage frequencies and prevalences, is implemented in the R package MLMOI [54], which allows to import and manipulate several types of molecular data in a flexible way, targeted to users without a strong background in programming. The bias-corrected estimates introduced here will be added to the package in the near future. Further improvements on the model, such as allowing for more flexible distributions for MOI, e.g., the negative binomial distribution, are currently in progress.

## Supporting information

S1 Appendix Mathematical appendix. (ZIP)

S2 Appendix Additional figures showing detailed results. (ZIP)

S1 Table Summary of model parameters. (ZIP)

S1 Fig Bias of lineage-frequency estimates. The figure shows the relative bias in % of the BCMLE (plots on the right in each panel) and the MLE (plots on the left in each panel) of lineage frequencies ***p*** as a function of the true parameter *ψ* based on simulated data created by the conditional Poisson model. Each panel assumes a different lineage-frequency distribution ***p*** shown at the top of each panel. In panel **A**, the relative bias in % of only one lineage frequency is illustrated, because all lineage frequencies are equal and their relative bias is almost identical. Different rows in panel **B** correspond to different lineage frequencies. Each colored line corresponds to a different sample size *N*.
(ZIP)

S2 Fig Bias of heuristically adjusted lineage-frequency estimates. As S1 Fig but for the BCMLE *vs* the HBCMLE2.
(ZIP)

S3 Fig Variance of lineage frequency estimates. Similar to S1 Fig but for the coefficient of variation in %. The dotted lines are the respective predictions based on the Cramér-Rao lower bounds.
(ZIP)

S4 Fig Euclidean distance and Kullback-Leiber divergence. The figure shows the Euclidean distance (**A**) and Kullback-Leiber divergence (**B**) between the true frequencies and BCMLEs (solid lines) and the true frequencies and MLEs (dashed lines) for a lineage-frequency distribution as a function of the true parameter *ψ* based on simulated data created by the conditional Poisson model. The lineage-frequency distributions are shown at the top of each panel. Each colored line corresponds to a different sample size *N*.
(ZIP)
