# Supplementary material for: Bias-corrected maximum-likelihood estimation of multiplicity of infection and lineage frequencies
Source: PLoS One. 2021 Dec 29;16(12):e0261889. doi: 10.1371/journal.pone.0261889 (PMC8716058; doi:10.1371/journal.pone.0261889)
Supplement: S1 Appendix — (ZIP) [file pone.0261889.s001.zip › S1_Appendix.pdf]

S1 Appendix

Mathematical Appendix

Before we derive the Cramér-Rao lower bound, we introduce two transformations to facilitate the course of calculations. This is justified by the fact that the MLE is transformation respecting. Let

$$\gamma_k = \frac{1}{e^{\lambda p_k} - 1}, \tag{1}$$

and

$$\gamma_0 = \frac{1}{e^{\lambda} - 1}. \tag{2}$$

Hence, the log-likelihood becomes

$$\ell(\lambda, \boldsymbol{p}) = N \log(\gamma_0) - \sum_{k=1}^{n} N_k \log(\gamma_k). \tag{3}$$

We also need the derivatives of the transformed parameters with respect to the original ones, given by

$$\frac{\partial \gamma_k}{\partial \lambda} = -p_k \gamma_k (\gamma_k + 1), \tag{4a}$$

$$\frac{\partial \gamma_k}{\partial p_k} = -\lambda \gamma_k (\gamma_k + 1), \tag{4b}$$

$$\frac{\partial \gamma_0}{\partial \lambda} = -\gamma_0 (\gamma_0 + 1), \tag{4c}$$

$$\frac{\partial \gamma_0}{\partial p_k} = 0, \tag{4d}$$

$$\frac{\partial \gamma_k}{\partial p_j} = 0, \tag{4e}$$

$$\frac{\partial \gamma_k}{\partial \beta} = \frac{\partial \gamma_0}{\partial \beta} = 0, \tag{4f}$$

for $k, j = 1, 2, \ldots, n$ and $j \neq k$. For obtaining the cumulants and the entries of the Fisher information, we need the expectation of the random variables N_k. Knowing that $N_k = \sum_{j=1}^{N} x_k^{(j)}$, its expectation is derived straightforwardly as

$$\mathbb{E}(N_k) = N \frac{\gamma_0 + 1}{\gamma_k + 1}, \tag{5}$$

[1]. We also need the second and third derivatives of the Lagrange function (6) with respect to the model parameters. For $k, j = 1, \ldots, n$ $(k \neq j)$ the second and third

derivatives are respectively

$$\frac{\partial^2\Lambda}{\partial\lambda^2} = N\gamma_0(\gamma_0+1) - \sum_{k=1}^{n} N_k p_k^2 \gamma_k(\gamma_k+1), \tag{6a}$$

$$\frac{\partial^2\Lambda}{\partial\lambda\partial p_k} = N_k(\gamma_k+1)(\ 1-\lambda p_k\gamma_k), \tag{6b}$$

$$\frac{\partial^2\Lambda}{\partial p_k^2} = -N_k\lambda^2\gamma_k(\gamma_k+1), \tag{6c}$$

$$\frac{\partial^2\Lambda}{\partial p_k\partial p_j} = 0, \tag{6d}$$

$$\frac{\partial^2\Lambda}{\partial p_k\partial\beta} = -1, \tag{6e}$$

$$\frac{\partial^2\Lambda}{\partial\beta\partial\lambda} = 0, \tag{6f}$$

$$\frac{\partial^2\Lambda}{\partial\beta^2} = 0, \tag{6g}$$

and

$$\frac{\partial^3\Lambda}{\partial\lambda^3} = -N\gamma_0(\gamma_0+1)(2\gamma_0+1) + \sum_{k=1}^{n} N_k p_k^3\gamma_k(\gamma_k+1)(2\gamma_k+1), \tag{7a}$$

$$\frac{\partial^3\Lambda}{\partial\lambda^2\partial p_k} = -N_k p_k\gamma_k(\gamma_k+1)\Big(2-\lambda p_k(2\gamma_k+1)\Big), \tag{7b}$$

$$\frac{\partial^3\Lambda}{\partial\lambda\partial p_k^2} = \frac{\lambda}{p_k}\Big(\frac{\partial^3\Lambda}{\partial\lambda^2\partial p_k}\Big), \tag{7c}$$

$$\frac{\partial^3\Lambda}{\partial\lambda\partial p_k\partial p_j} = \frac{\partial^3\Lambda}{\partial p_k\partial p_k\partial p_j} = \frac{\partial^3\Lambda}{\partial p_k\partial p_j\partial p_i} = 0, \tag{7d}$$

$$\frac{\partial^3\Lambda}{\partial p_k^3} = N_k\lambda^3\gamma_k(\gamma_k+1)(2\gamma_k+1), \tag{7e}$$

$$\frac{\partial^3\Lambda}{\partial\beta^3} = \frac{\partial^3\Lambda}{\partial\beta^2\partial\lambda} = \frac{\partial^3\Lambda}{\partial\beta^2\partial p_k} = \frac{\partial^3\Lambda}{\partial\beta\partial\lambda^2} = \frac{\partial^3\Lambda}{\partial\beta\partial\lambda\partial p_k} = \frac{\partial^3\Lambda}{\partial\beta\partial p_k^2} = \frac{\partial^3\Lambda}{\partial\beta\partial p_k\partial p_j} = 0. \tag{7f}$$

Note, that the order of the derivatives in (6) and (7) can be interchanged. In what follows, we adjust the notation. To denote derivatives with respect to the model parameters, we refrain (with some exceptions) from using indices $i, j, k, \ldots = 1, 2, \ldots, (n+2)$, and use the parameters themselves as indices. This results in a more convenient notation for the following proofs.

Proof of Remark 1. The entries of Fisher information are easily derived as $\tau_{ij} := -\mathbb{E}\Big(\frac{\partial^2\Lambda}{\theta_i\theta_j}\Big) = -k_{ij}$ where θ_i belongs to the set of original model parameters, i.e.,

$\theta_i \in \{\lambda, \beta, p_1, \ldots, p_n\}$. They are given by

$$\tau_{\lambda\lambda} = -k_{\lambda\lambda} = -N(\gamma_0 + 1)\Big(\gamma_0 - \sum_{k=1}^{n} p_k^2 \gamma_k\Big), \tag{8a}$$

$$\tau_{\lambda p_k} = -k_{\lambda p_k} = \tau_{p_k \lambda} = -N(\gamma_0 + 1)(1 - \lambda p_k \gamma_k), \tag{8b}$$

$$\tau_{p_k p_k} = -k_{p_k p_k} = \lambda^2 N(\gamma_0 + 1)\gamma_k, \tag{8c}$$

$$\tau_{p_k p_j} = -k_{p_k p_j} = 0, \tag{8d}$$

$$\tau_{p_k \beta} = -k_{p_k \beta} = 1, \tag{8e}$$

$$\tau_{\lambda\beta} = \tau_{\beta\beta} = -k_{\lambda\beta} = -k_{\beta\beta} = 0. \tag{8f}$$

We derive the inverse Fisher information by Woodbury's formula [2]. For this purpose, let

$$U := \begin{pmatrix} 1 & 0 \\ 0 & 1 \\ -1 & \tau_{\lambda p_1} \\ -1 & \tau_{\lambda p_2} \\ \vdots & \vdots \\ -1 & \tau_{\lambda p_n} \end{pmatrix}, \quad V := \begin{pmatrix} 0 & 1 \\ -1 & 0 \\ \tau_{\lambda p_1} & 1 \\ \tau_{\lambda p_2} & 1 \\ \vdots & \vdots \\ \tau_{\lambda p_n} & 1 \end{pmatrix}^T, \tag{9}$$

and

$$A := \begin{pmatrix} B & Z^T \\ Z & C \end{pmatrix}, \tag{10}$$

where

$$B := \begin{pmatrix} \tau_{\lambda\lambda} & 1 \\ -1 & 0 \end{pmatrix}, \quad C := \operatorname{diag}(\tau_{p_1 p_1}, \tau_{p_2 p_2}, \ldots, \tau_{p_n p_n}), \tag{11}$$

and Z is the $2 \times n$ matrix with all entries equal to 0. Note that the Fisher information can be decomposed as $\mathcal{I}_N = A + UV$. By Woodbury's formula we have

$$(A + UV)^{-1} = A^{-1} - A^{-1} U \Sigma^{-1} V A^{-1}, \tag{12}$$

with $\Sigma = I_2 + V A^{-1} U$, where I_2 denotes the 2×2 identity matrix. Its inverse is readily derived to be

$$\Sigma^{-1} = \frac{1}{\det \Sigma} \begin{pmatrix} \displaystyle\sum_{k=1}^{n} \frac{\tau_{\lambda p_k}}{\tau_{p_k p_k}} & \displaystyle\tau_{\lambda\lambda} - \sum_{k=1}^{n} \frac{\tau_{\lambda p_k}^2}{\tau_{p_k p_k}} \\ \displaystyle\sum_{k=1}^{n} \frac{1}{\tau_{p_k p_k}} & \displaystyle -\sum_{k=1}^{n} \frac{\tau_{\lambda p_k}}{\tau_{p_k p_k}} \end{pmatrix}, \tag{13}$$

where

$$\det \Sigma = \frac{1}{\tau_{p_k p_k}} \Big(\sum_{k=1}^{n} \frac{\tau_{\lambda p_k}^2}{\tau_{p_k p_k}} - \tau_{\lambda\lambda} \Big) - \Big(\sum_{k=1}^{n} \frac{\tau_{\lambda p_k}}{\tau_{p_k p_k}} \Big)^2. \tag{14}$$

Straightforward calculations give

$$\Sigma^{-1} = \begin{pmatrix} \dfrac{\gamma - \lambda}{1 - \gamma\gamma_0} & N(\gamma_0 + 1)\Big(\dfrac{\gamma - 2\lambda - \lambda^2 \gamma_0}{1 - \gamma\gamma_0}\Big) \\ \dfrac{\gamma}{N(\gamma_0 + 1)(1 - \gamma\gamma_0)} & -\dfrac{\gamma - \lambda}{1 - \gamma\gamma_0} \end{pmatrix}, \tag{15}$$

where $\gamma = \sum_{k=1}^{n} \frac{1}{\gamma_k} = \sum_{k=1}^{n} e^{\lambda p_k} - 1$. Eventually we derive the entries of inverse Fisher information as following

$$\tau^{(\lambda\lambda)} = \frac{1}{N(\gamma_0+1)} \frac{\gamma}{1-\gamma\gamma_0}, \tag{16a}$$

$$\tau^{(\lambda p_k)} = \frac{1}{\lambda N(\gamma_0+1)\gamma_k} \frac{1-p_k\gamma\gamma_k}{1-\gamma\gamma_0}, \tag{16b}$$

$$\tau^{(p_k p_j)} = \frac{1}{\lambda^2 N(\gamma_0+1)} \left(\frac{p_k p_j \gamma - \frac{p_j}{\gamma_k} - \frac{p_k}{\gamma_j} + \frac{\gamma_0}{\gamma_k\gamma_j}}{(1-\gamma\gamma_0)} \right), \tag{16c}$$

$$\tau^{(p_k p_k)} = \frac{1}{\lambda^2 N(\gamma_0+1)} \left(\frac{1}{\gamma_k} + \frac{p_k^2\gamma - 2\frac{p_k}{\gamma_k} + \frac{\gamma_0}{\gamma_k^2}}{(1-\gamma\gamma_0)} \right). \tag{16d}$$

Since we do not need the entries correspond to the nuisance parameter β, they are not derived here. □

Next follows the proof of Remark 2.

Proof of Remark 2. To derive the Fisher information of the average number of super-infections (MOI), we need the expectation of the second-derivatives with respect to the transformed parameters $\tilde{\boldsymbol{\theta}} = (\psi, \beta, \boldsymbol{p})$. Let $\boldsymbol{T} = (f(\hat{\lambda}), \hat{\beta}, \hat{\boldsymbol{p}})$ be an unbiased estimator of $\tilde{\boldsymbol{\theta}}$ (where $\psi = f(\lambda) = \lambda(\gamma_0 + 1)$). According to [3] (Chapter 7, p. 304) the covariance matrix $\mathrm{var}(\boldsymbol{T})$ satisfies

$$\mathrm{var}(\boldsymbol{T}) \geq \frac{\partial\tilde{\boldsymbol{\theta}}}{\partial\boldsymbol{\theta}^T} I_N^{-1} \frac{\partial\tilde{\boldsymbol{\theta}}^T}{\partial\boldsymbol{\theta}}, \tag{17}$$

where, the matrix $\frac{\partial\tilde{\boldsymbol{\theta}}}{\partial\boldsymbol{\theta}^T}$ is the transpose of $\frac{\partial\tilde{\boldsymbol{\theta}}^T}{\partial\boldsymbol{\theta}}$. In our case, the matrix $\frac{\partial\tilde{\boldsymbol{\theta}}}{\partial\boldsymbol{\theta}^T}$ is given by

$$\frac{\partial\tilde{\boldsymbol{\theta}}}{\partial\boldsymbol{\theta}^T} = \mathrm{diag}(f'(\lambda), 1, 1, \ldots, 1) \tag{18}$$

with

$$f'(\lambda) = (\gamma_0 + 1)(1 - \lambda\gamma_0). \tag{19}$$

The right-hand side of (17) is the Cramér-Rao bound. By matrix multiplication this yields (11), where the entries corresponding to the (co-)variances of nuisance parameter β are omitted. □

The bias of the estimator is given by (19a) and (19c). The respective expression in terms of the model parameters are presented in Result 1. Here, we present the proof.

Proof of Result 1. The bias of the estimator is given by (19a) . We need to express the equations in terms of the original model parameters λ and $\boldsymbol{p}$.

The cumulants (13b) are derived by combining (7) with (1) and (2). Straightforward calculation gives

$$k_{\lambda\lambda\lambda} = -N(\gamma_0+1)\Big(\gamma_0(2\gamma_0+1) - \sum_{k=1}^{n} p_k^3\gamma_k(2\gamma_k+1)\Big), \tag{20a}$$

$$k_{\lambda\lambda p_k} = -Np_k(\gamma_0+1)\gamma_k\Big(2-\lambda p_k(2\gamma_k+1)\Big), \tag{20b}$$

$$k_{\lambda p_k p_k} = -\lambda N(\gamma_0+1)\gamma_k\Big(2-\lambda p_k(2\gamma_k+1)\Big), \tag{20c}$$

$$k_{\lambda p_k p_j} = k_{p_k p_k p_j} = k_{p_k p_j p_i} = 0, \tag{20d}$$

$$k_{p_k p_k p_k} = \lambda^3 N(\gamma_0+1)\gamma_k(2\gamma_k+1), \tag{20e}$$

$$k_{\beta\beta\beta} = k_{\beta\beta\lambda} = k_{\beta\beta p_k} = k_{\beta\lambda\lambda} = k_{\beta\lambda p_k} = k_{\beta p_k p_k} = k_{\beta p_k p_j} = 0. \tag{20f}$$

The cumulants (13a) are given by (8) in the proof of Remark 1. Their derivatives with respect to the relevant model parameters are

$$k_{\lambda\lambda}^{(\lambda)} = -N(\gamma_0+1)\Big(2\gamma_0^2 + \gamma_0\big(1-\sum_{k=1}^{n} p_k^2\gamma_k\big) - \sum_{k=1}^{n} p_k^3(\gamma_k^2+\gamma_k)\Big), \tag{21a}$$

$$k_{\lambda p_k}^{(\lambda)} = -N(\gamma_0+1)\Big(\gamma_0(1-\lambda p_k\gamma_k) + p_k\gamma_k\big(1-\lambda p_k(\gamma_k+1)\big)\Big), \tag{21b}$$

$$k_{p_k p_k}^{(\lambda)} = -\lambda N(\gamma_0+1)\gamma_k\Big(2-\lambda\gamma_0-\lambda p_k(\gamma_k+1)\Big), \tag{21c}$$

$$k_{p_k p_j}^{(\lambda)} = k_{\lambda p_k}^{(p_j)} = 0, \tag{21d}$$

$$k_{\lambda\lambda}^{(p_k)} = -Np_k(\gamma_0+1)\gamma_k\Big(2-\lambda p_k(\gamma_k+1)\Big), \tag{21e}$$

$$k_{\lambda p_k}^{(p_k)} = -\lambda N(\gamma_0+1)\gamma_k\Big(1-\lambda p_k(\gamma_k+1)\Big), \tag{21f}$$

$$k_{p_k p_k}^{(p_k)} = \lambda^3 N(\gamma_0+1)\gamma_k(\gamma_k+1), \tag{21g}$$

$$k_{p_k p_j}^{(p_k)} = k_{p_j p_j}^{(p_k)} = k_{p_i p_j}^{(p_k)} = 0, \tag{21h}$$

$$k_{\beta\beta}^{(\beta)} = k_{\beta\beta}^{(\lambda)} = k_{\beta\lambda}^{(\beta)} = k_{\beta\lambda}^{(\lambda)} = k_{\lambda\lambda}^{(\beta)} = 0, \tag{21i}$$

$$k_{\beta\beta}^{(p_k)} = k_{\beta p_k}^{(\beta)} = k_{\beta p_k}^{(p_k)} = k_{p_k p_k}^{(\beta)} = 0, \tag{21j}$$

$$k_{\beta p_k}^{(p_j)} = k_{p_k p_j}^{(\beta)} = k_{\beta\lambda}^{(p_k)} = k_{\beta p_k}^{(\lambda)} = k_{\lambda p_k}^{(\beta)} = 0, \tag{21k}$$

where $k, j, i = 1, \ldots, n$ are pairwise different. To derive the entries of the matrix A defined by (18), we need to combine (20) and (21). The entries of matrix $A^{(\lambda)}$ are derived to be

$$a_{\lambda\lambda}^{(\lambda)} = -N(\gamma_0+1)\Big(\gamma_0^2 + \frac{\gamma_0}{2} - \gamma_0\sum_{k=1}^{n} p_k^2\gamma_k - \frac{1}{2}\sum_{k=1}^{n} p_k^3\gamma_k\Big), \tag{22a}$$

$$a_{\lambda p_k}^{(\lambda)} = -N(\gamma_0+1)\Big(\gamma_0\big(1-\lambda p_k\gamma_k\big) - \frac{\lambda p_k^2\gamma_k}{2}\Big), \tag{22b}$$

$$a_{p_k p_k}^{(\lambda)} = -\lambda N(\gamma_0+1)\gamma_k\Big(1-\lambda\gamma_0-\frac{\lambda p_k}{2}\Big), \tag{22c}$$

$$a_{p_k p_j}^{(\lambda)} = 0. \tag{22d}$$

The entries of the matrix $A^{(\beta)}$ are all zero. Finally, the entries of the matrices $A^{(p_k)}$ are

$$a_{\lambda\lambda}^{(p_k)} = -N(\gamma_0+1)p_k\gamma_k\Big(1-\frac{\lambda p_k}{2}\Big), \tag{22e}$$

$$a_{\lambda p_k}^{(p_k)} = \frac{1}{2}\lambda^2 N(\gamma_0+1)p_k\gamma_k, \tag{22f}$$

$$a_{p_k p_k}^{(p_k)} = \frac{1}{2}\lambda^3 N(\gamma_0+1)\gamma_k, \tag{22g}$$

$$a_{\lambda p_j}^{(p_k)} = a_{p_j p_j}^{(p_k)} = a_{p_k p_j}^{(p_k)} = a_{p_i p_j}^{(p_k)} = 0, \tag{22h}$$

where $k, j, i = 1, \ldots, n$ are pairwise different.

Let

$$\gamma = \sum_{k=1}^{n}\frac{1}{\gamma_k} = \sum_{k=1}^{n} e^{\lambda p_k} - 1.$$

The bias written in matrix form is given by (16). To derive this, we need the product

$$A\,\mathrm{vec}(I_N^{-1}) = (a_\lambda, 0, a_{p_1}, \ldots, a_{p_n})^T, \tag{23}$$

where

$$a_\lambda = \frac{1}{2} + \gamma_0 - \frac{1}{1-\gamma\gamma_0}\left(\frac{\gamma\gamma_0}{2} + \frac{1}{\lambda} - \frac{\gamma}{\lambda}\sum_{k=1}^{n} p_k^2\gamma_k - \frac{\gamma_0}{2}\sum_{k=1}^{n}\frac{p_k}{\gamma_k}\right), \tag{24}$$

and

$$a_{p_k} = \frac{\lambda}{2} - \frac{1}{1-\gamma\gamma_0}\left(1 + \gamma\gamma_0 - \lambda\gamma_0 - p_k\gamma\gamma_k - \frac{\lambda\gamma_0}{2\gamma_k}\right). \tag{25}$$

The bias vector is calculated by multiplying the inverse Fisher information (8) with the vector (23) according to equation (16). The bias vector (disregarding the term for the nuisance parameter β) has the entries

$$B_{\boldsymbol{\theta}}(\hat{\lambda}) = \frac{1}{N(\gamma_0+1)(1-\gamma\gamma_0)}\left(\frac{\gamma(2\gamma_0+1)}{2} - \frac{\gamma_0}{2(1-\gamma\gamma_0)}\Big(\gamma^2 - \sum_{j=1}^{n}\frac{1}{\gamma_j^2}\Big)\right) + \mathcal{O}(N^{-2}). \tag{26}$$

Similarly the second-order bias for lineage frequencies are derived as

$$\begin{aligned} B_{\boldsymbol{\theta}}(\hat{p}_k) = \frac{1}{\lambda N(\gamma_0+1)(1-\gamma\gamma_0)} \Bigg(&\Big(\frac{1}{\gamma_k} - p_k\gamma\Big)\Big(\gamma_0 + \frac{1}{2} - \frac{1}{\lambda}\Big) + \frac{\gamma_0}{2\gamma_k^2} \\ &+ \frac{\gamma_0}{2(1-\gamma\gamma_0)}\bigg(\Big(p_k\gamma^2 - \frac{\gamma}{\gamma_k}\Big) + \Big(\frac{\gamma_0}{\gamma_k} - p_k\Big)\sum_{j=1}^{n}\frac{1}{\gamma_j^2}\bigg)\Bigg) + \mathcal{O}(N^{-2}), \end{aligned} \tag{27}$$

for $k = 1, \ldots, n$. Replacing γ_k and γ_0 by their equivalents given in (1) and (2) finishes the proof.

□

Simulation study

We conducted systematic numerical simulations to investigate the performance of the estimates of interest for a wide range of parameter choices including (i) a range of values for the MOI parameter (λ), (ii) different numbers of lineages (n), (iii) different

lineage-frequency distributions ($\boldsymbol{p}$) for a given number of lineages, and (iv) different sample sizes (N). We further investigated the robustness of the estimators with regard to model violations, i.e., how well the estimators perform if the true model that generates the observations departs form the Poisson model described in section Model Background. For the departing model we assumed that the number of super-infections follows a negative binomial rather than a Poisson distribution (see below).

Measuring the performance of estimators

The accuracy to estimate parameters of interest is typically measured in terms of bias. The magnitude of an estimator's bias depends on the value of the true parameter. To compare bias across a range of parameters it is more appropriate to consider the relative bias. Because bias cannot be calculated analytically, the relative bias was approximated by the empirical bias divided by the true parameter. More precisely, given a true parameter vector $\boldsymbol{\theta}$ and sample size N, we generated randomly S datasets $\mathcal{X}_1, \ldots, \mathcal{X}_S$, and derived the (empirical) relative bias of a parameter θ as

$$\frac{\mathbb{E}_S(\hat{\theta}) - \theta}{\theta}, \tag{28}$$

where $\hat{\theta}$ is an estimator of the parameter θ, e.g., $\hat{\psi}^{(\mathrm{bc})}$ the BCMLE of ψ, and $\mathbb{E}_S$ denotes the sample mean, i.e.,

$$\mathbb{E}_S(\hat{\theta}) = \frac{1}{S} \sum_{s=1}^{S} \hat{\theta}^{(s)}, \tag{29}$$

where $\hat{\theta}^{(s)}$ is the estimate of θ derived from the dataset $\mathcal{X}_s$.

The variance of an estimator also depends on the parameter choice, and makes comparisons across parameter ranges difficult. We therefore used the coefficient of variation (CV) to measure an estimator's precision. An estimator's CV for a parameter θ was calculated as the sample standard deviation divided by the true parameter, i.e.,

$$\frac{\sqrt{\mathrm{Var}_S(\hat{\theta})}}{\theta}, \tag{30}$$

where Var_S denotes the sample variance, derived as

$$\mathrm{Var}_S(\hat{\theta}) = \frac{1}{S-1} \sum_{s=1}^{S} \left(\hat{\theta}^{(s)} - \theta\right)^2. \tag{31}$$

In our case estimates are meaningless for pathological data ($N_k = N$ for at least one k, or $\sum_{k=1}^{n} N_k = N$) or might not exist. Hence, the empirical mean and variance of an estimator must be derived only from regular data. We therefore assume the S datasets $\mathcal{X}_1, \ldots, \mathcal{X}_S$ to be regular.

Setup of the simulation study

For the Poisson model, first we measured the performance of different estimators for the average MOI $\psi = \frac{\lambda}{1-e^{-\lambda}}$, this is the quantity of interest. Notably, the MLE $\hat{\psi}$ of ψ is given by

$$\hat{\psi} = \frac{\hat{\lambda}}{1-e^{-\hat{\lambda}}}. \tag{32}$$

For each choice of the model parameters $\boldsymbol{\theta} = (\lambda, \boldsymbol{p})$ and sample size N, we randomly generated $S = 100,000$ regular datasets $\mathcal{X}_1, \ldots, \mathcal{X}_S$. For each dataset, the MLE of the average MOI $\hat{\psi}$ along with the bias-corrected versions $\hat{\psi}^{(\mathrm{bc})}$, $\hat{\psi}^{(\mathrm{hbc1})}$, $\hat{\psi}^{(\mathrm{hbc2})}$ and $\hat{\psi}^{(\mathrm{hbc3})}$ were derived. We then obtained the relative bias and CV of these estimators as described above.

From these datasets we also derived the relative bias and CV for the different versions of lineage frequency estimates. We derived the MLE $\hat{\boldsymbol{p}} = (\hat{p}_1, \ldots, \hat{p}_n)$ and the bias-corrected versions $\hat{\boldsymbol{p}}^{(bc)} = (\hat{p}_1^{(\mathrm{bc})}, \ldots, \hat{p}_n^{(\mathrm{bc})})$, and $\hat{\boldsymbol{p}}^{(hbc2)} = (\hat{p}_1^{(hbc2)}, \ldots, \hat{p}_n^{(hbc2)})$. (Note, the estimators $\hat{\boldsymbol{p}}^{(hbc1)}$ and $\hat{\boldsymbol{p}}^{(hbc3)}$ are the same as $\hat{\boldsymbol{p}}^{(bc)}$; see Remarks 3 and 5).

We derived the relative bias and CV for each lineage frequency p_k separately. Comparing the performance of frequency estimates for distributions with different number of lineages (different n) is not straightforward. We therefore also employed distance measures to evaluate how close the estimates $\hat{\boldsymbol{p}}$ are on average to the true parameters $\boldsymbol{p}$. As a distance measure we used the Euclidian-norm. (Note that all distances in $\mathbb{R}^n$ are equivalent, therefore one expects quantitative but not qualitative differences between distances.) We also used the Kullback–Leibler divergence, $\sum_{k=1}^{n} \hat{p}_k \log \frac{\hat{p}_k}{p_k}$, which is not a distance because it is not symmetric.

Constructing a dataset under the Poisson model

To construct a dataset $\mathcal{X}_s$ under the Poisson model we proceeded as follows.

For a given choice of parameters $\boldsymbol{\theta} = (\lambda, \boldsymbol{p})$ a dataset of sample size N consists of N 0-1 vectors $\boldsymbol{x}^{(1)}, \boldsymbol{x}^{(2)}, \ldots, \boldsymbol{x}^{(N)}$, indicating absence and presence of lineages. Each 0-1 vector $\boldsymbol{x}^{(j)}$ is constructed as follows. First, multiplicity of infection m is determined by randomly choosing a number m from a conditional Poisson distribution (1) with parameter λ, i.e,

$$P(Y = m) = \frac{1}{e^{\lambda} - 1} \frac{\lambda^m}{m!} \quad \text{for } m \geq 1. \tag{33}$$

In the second step, we choose a random vector $\boldsymbol{m} = (m_1, m_2, \ldots, m_n)$ from a multinomial distribution with parameters m and $\boldsymbol{p}$, i.e.,

$$P(\boldsymbol{m}|Y = m) = \frac{m!}{m_1! \ldots m_n!} p_1^{m_1} \ldots p_n^{m_n}. \tag{34}$$

Finally, the observation $\boldsymbol{x}^{(j)}$ is obtained as

$$\boldsymbol{x}^{(j)} = \big(\operatorname{sign}(m_1), \operatorname{sign}(m_2), \ldots, \operatorname{sign}(m_n)\big). \tag{35}$$

This procedure is repeated N times to yield a dataset $\mathcal{X}_s$ of size N. If the dataset was pathologic, it was discarded and the procedure was repeated until we obtained a regular data set $\mathcal{X}_s$.

Constructing a dataset under the negative binomial model

To study the robustness of the estimator with regard to model violations, we constructed data from a different probabilistic model, in which MOI follows a negative binomial distribution.

Datasets under the negative binomial model were constructed as under the Poisson model. However, instead MOI m was not sampled from a conditional Poisson

distribution with parameter λ, but from a conditional negative binomial distribution with parameters r and p, i.e.,

$$P(Y=m)=\frac{\Gamma(m+r)}{m!\Gamma(r)}\frac{p^r}{1-p^r}(1-p)^m. \tag{36}$$

Hence, the parameters under the negative binomial model are not $(\lambda, \boldsymbol{p})$ but $(r, p, \boldsymbol{p})$.

Note the average MOI under the Poisson model is

$$\psi=\psi_{CPD}=\mathbb{E}(Y)=\frac{\lambda}{1-e^{-\lambda}}. \tag{37}$$

whereas that under the negative binomial model is

$$\psi=\psi_{CNB}=\mathbb{E}(Y)=\frac{r(1-p)}{p(1-p^r)}. \tag{38}$$

The negative binomial distribution captures overdispersion in the mosquito biting rate [4]. We chose parameter values for the negative binomial distribution with the same means but larger variances than the choices for the Poisson distribution. For a given Poisson parameter λ, we chose parameters p and r for the negative binomial distribution that yielded the same mean but a variance that was over-dispersed by a given amount α. Particularly, for given λ and α, we found p and r by solving

$$\lambda=\frac{r(1-p)}{p}, \qquad \text{and} \qquad \alpha\,\lambda=\frac{r(1-p)}{p^2}.$$

$$r=\frac{\lambda}{\alpha-1}, \qquad \text{and} \qquad p=\frac{1}{\alpha}. \tag{39}$$

The empirical relative bias of the estimators for ψ_{CPD} under the model violation, i.e., under the negative binomial model, was calculated as

$$\frac{\mathbb{E}_S(\hat{\psi}_{CPD})-\psi_{CNB}}{\psi_{CNB}}=\frac{p(1-p^r)\,\mathbb{E}_S(\hat{\psi}_{CPD})-r(1-p)}{r(1-p)}, \tag{40}$$

where $\mathbb{E}_S(\hat{\psi}_{CPD})$ is calculated as described above, but with the underlying datasets $\mathcal{X}_1,\dots,\mathcal{X}_S$ being generated under the negative binomial model. Similarly, the CV was calculated as

$$\frac{\sqrt{\mathrm{Var}_S(\hat{\psi}_{CPD})}}{\psi_{CNB}}. \tag{41}$$

Parameter choices and implementation

We studied the bias and variance of the estimators for a wide range of parameters. The parameters used in the simulations are summarized in S1 Table. Simulations were either based on the true model (Poisson model) or, to investigate the robustness of the estimators, on the negative binomial distribution. For the latter we chose parameters r and p that matched the range of values for λ by solving (39) for different levels of overdispersion α. In particular, for all combinations of $\lambda=0.1,0.15,\dots,1.95,2$ and $\alpha=1.05,1.1,\dots,1.95,2$, we solved (39) to obtain the parameters r and p.

All numerical simulations were implemented in R [5]. Generating datasets for each combination of parameters took substantial computational time over the whole range of parameters. To facilitate computational speed, we generated datasets $\mathcal{X}_s$ for each

parameter combination using the maximum sample size $N = 400$. Each dataset was reused for all smaller sample sizes $\tilde{N}$, by retaining successively the first $\tilde{N} = 300$, 200, 150, 100, 80, 70, 60, 50, and 40 records, respectively. If a dataset that retained only the first $\tilde{N}$ records was pathological, a new dataset of size $\tilde{N}$ was created. We continued in this fashion until a regular dataset for the minimum sample size 40 was reached. Note for each sample size, this resulted in $S = 100,000$ independent datasets. However, the datasets used for different values of N were not independent – notably independence is not needed for comparison of the estimators across different sample sizes. This results in a correlation of the zigzag pattern in Figs 2-5 and in the Figs in S1 Additional Figures. The zigzag patterns emerge due to outliers in the generated datasets $\mathcal{X}_s$, and vanish as $S \to \infty$.

The MLE and all bias corrected version were calculated from the same datasets. This is adequate because we aimed to compare the different estimators. Therefore, we also see correlations in the zigzag patterns between the various estimators in the results.

References

1. Schneider KA, Escalante AA. A Likelihood Approach to Estimate the Number of Co-Infections. PLoS ONE. 2014;9(7):e97899. Available from: https://doi.org/10.1371/journal.pone.0097899.

2. Hager W. Updating the Inverse of a Matrix. SIAM Rev. 1989;31:221–239.

3. Davison AC. Statistical Models. Cambridge University Press; 2003. Available from: https://www.cambridge.org/core/product/identifier/9780511815850/type/book.

4. Smith D, Hay S. Endemicity response timelines for Plasmodium falciparum elimination. Malaria Journal. 2009;8(1):87. Available from: http://www.malariajournal.com/content/8/1/87.

5. R Core Team. R: A Language and Environment for Statistical Computing. Vienna, Austria; 2020. Available from: https://www.R-project.org/.
