# Supplementary material for: Bias-corrected maximum-likelihood estimation of multiplicity of infection and lineage frequencies
Source: PLoS One. 2021 Dec 29;16(12):e0261889. doi: 10.1371/journal.pone.0261889 (PMC8716058; doi:10.1371/journal.pone.0261889)
Supplement: S2 Appendix — (ZIP) [file pone.0261889.s002.zip › S2_Appendix.pdf]

Additional Figures

Meraj Hashemi, Kristan Alexander Schneider

Contents

1 The BCMLE of the average MOI **2**
1.1 Relative bias in % . . . 2
1.2 CV in % . . . 8

2 Heuristically adjusted estimates of the average MOI **14**
2.1 Relative bias in % . . . 14
2.2 CV in % . . . 20

3 Probability of regular data **26**

4 The BCMLE of lineage frequencies **28**
4.1 Relative bias in % . . . 28
4.2 CV in % . . . 33
4.3 Euclidean distance . . . 38
4.4 Kullback–Leibler divergence . . . 44

5 Model Violations **50**
5.1 Relative bias of the BCMLE in % . . . 50
5.1.1 Different levels of overdispersion . . . 50
5.1.2 Different sample sizes . . . 61
5.2 CV of the BCMLE in % . . . 72
5.2.1 Different levels of overdispersion . . . 72
5.2.2 Different sample sizes . . . 83
5.3 Heuristically adjusted estimates . . . 94
5.3.1 Relative bias in % . . . 94
5.3.2 CV in % . . . 103

1 The BCMLE of the average MOI

1.1 Relative bias in %

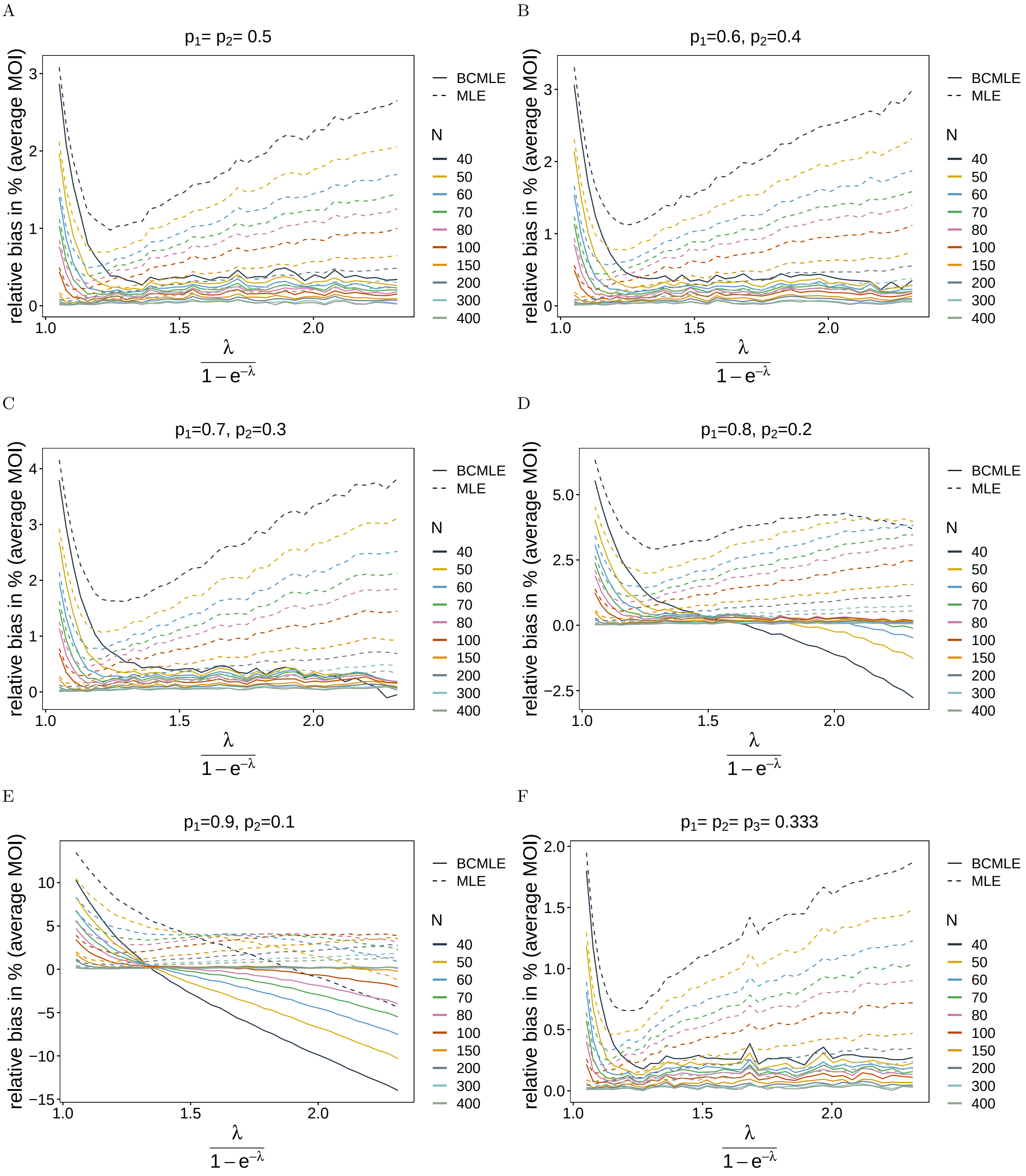

Figure 1: **Bias of MOI estimates**. The figure shows the relative bias in % of the BCMLE $\hat{\psi}^{(\mathrm{bc})}$ (solid lines) and MLE $\hat{\psi}$ (dashed lines) as a function of the true parameter ψ based on simulated data created by the conditional Poisson model. Each panel assumes a different lineage-frequency distribution $\boldsymbol{p}$ shown at the top of each panel. Each colored line corresponds to a different sample size N.

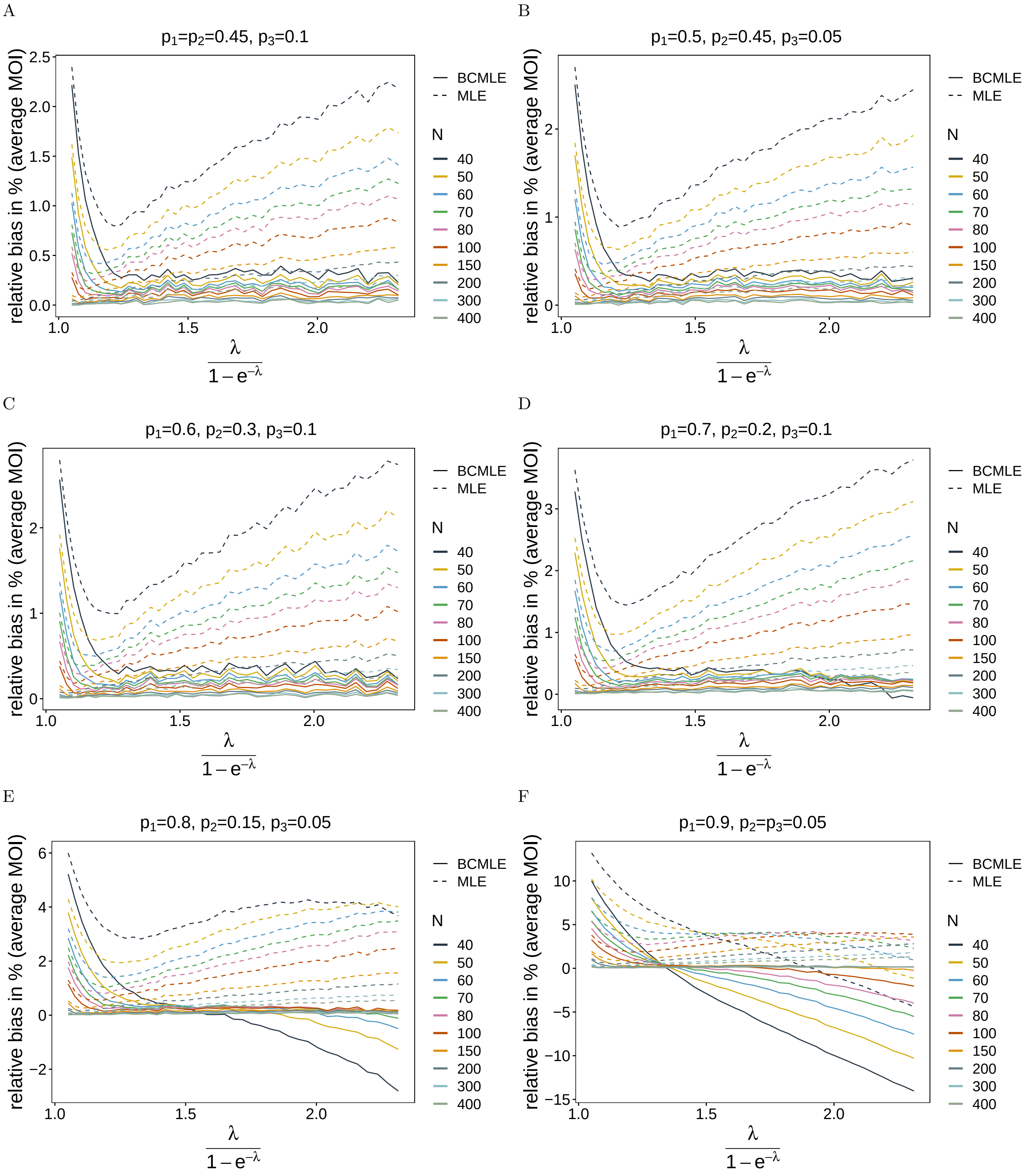

Figure 2: Similar to Figure 1 but for different lineage-frequency distributions.

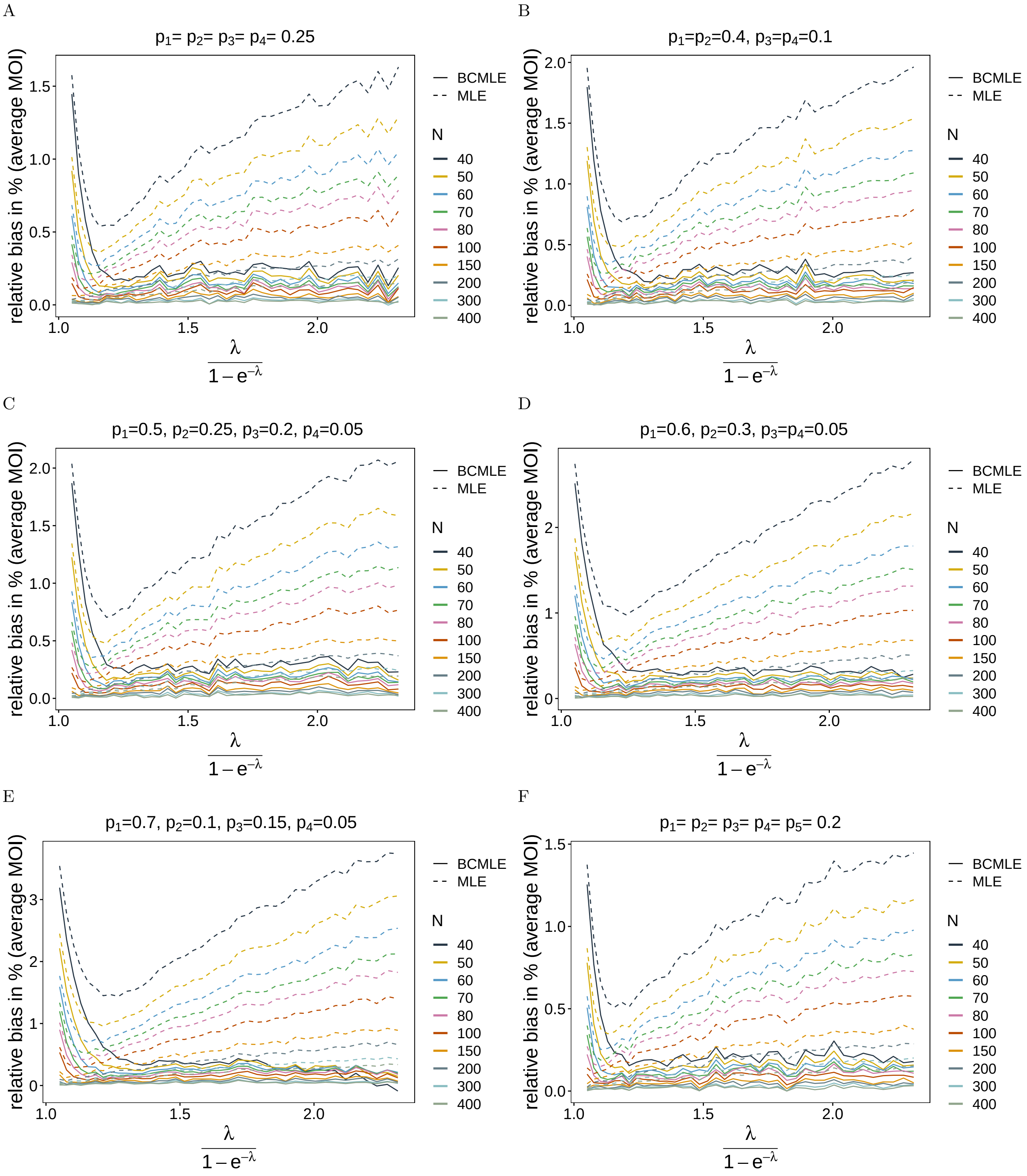

Figure 3: Similar to Figure 1 but for different lineage-frequency distributions.

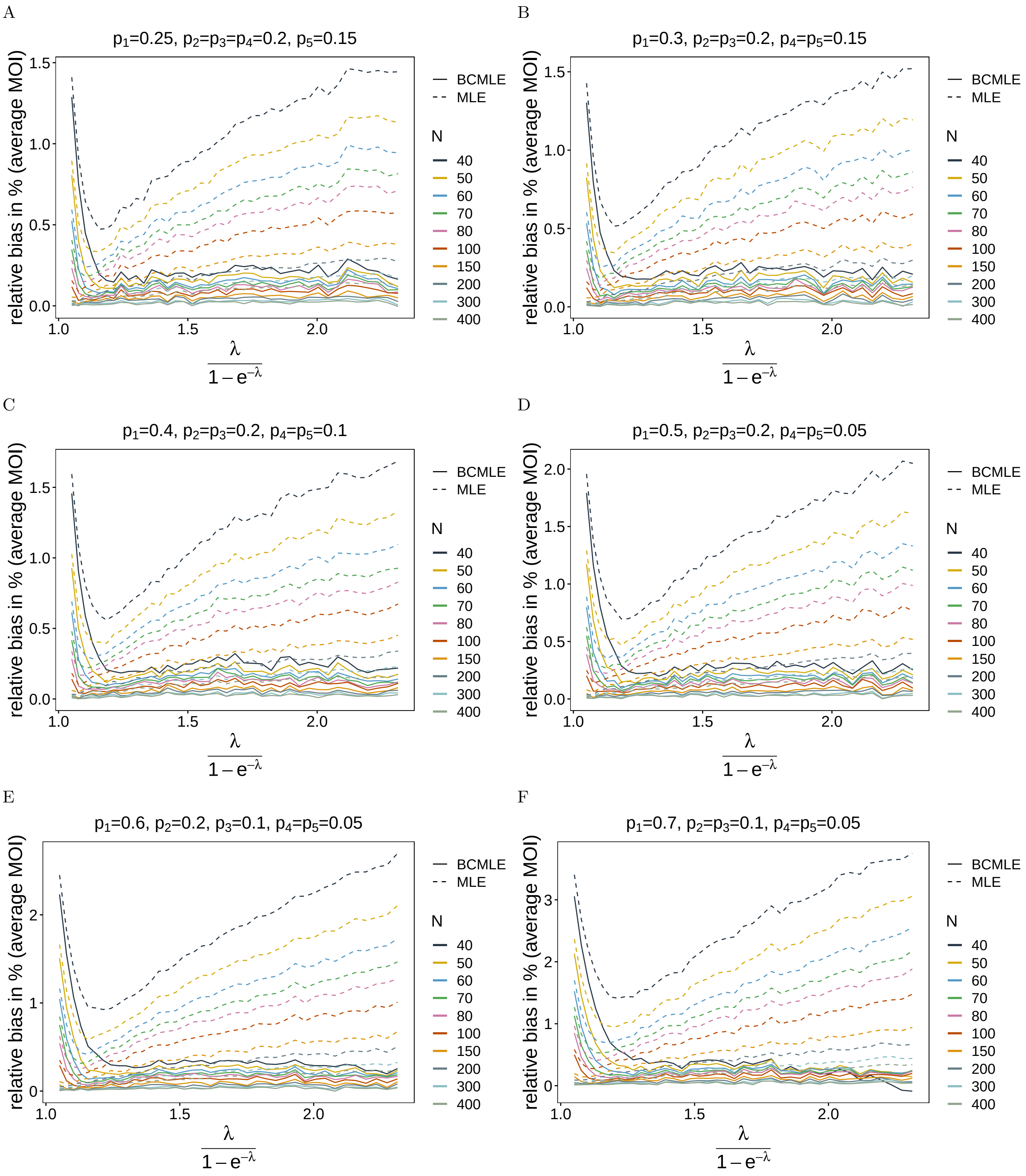

Figure 4: Similar to Figure 1 but for different lineage-frequency distributions.

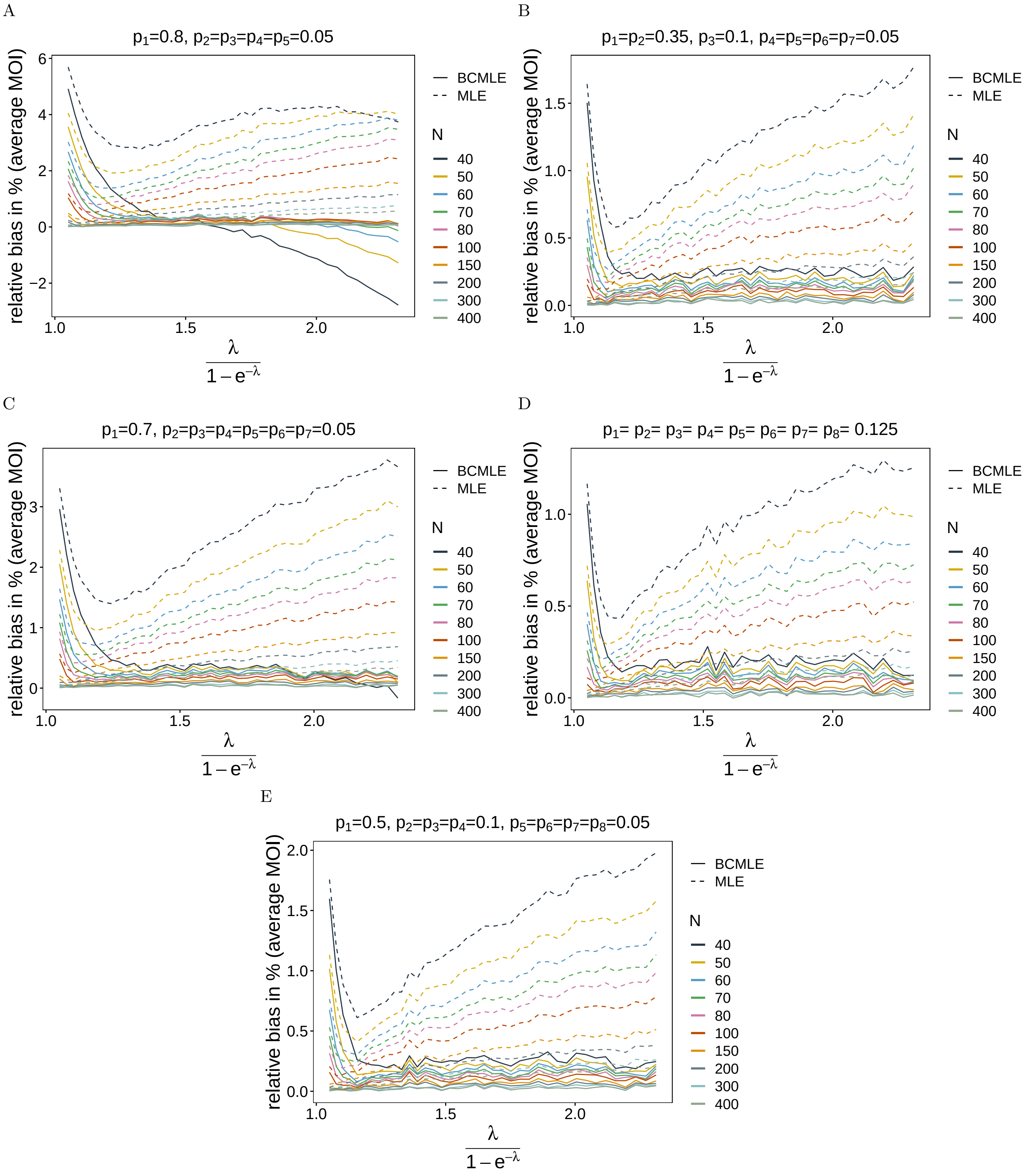

Figure 5: Similar to Figure 1 but for different lineage-frequency distributions.

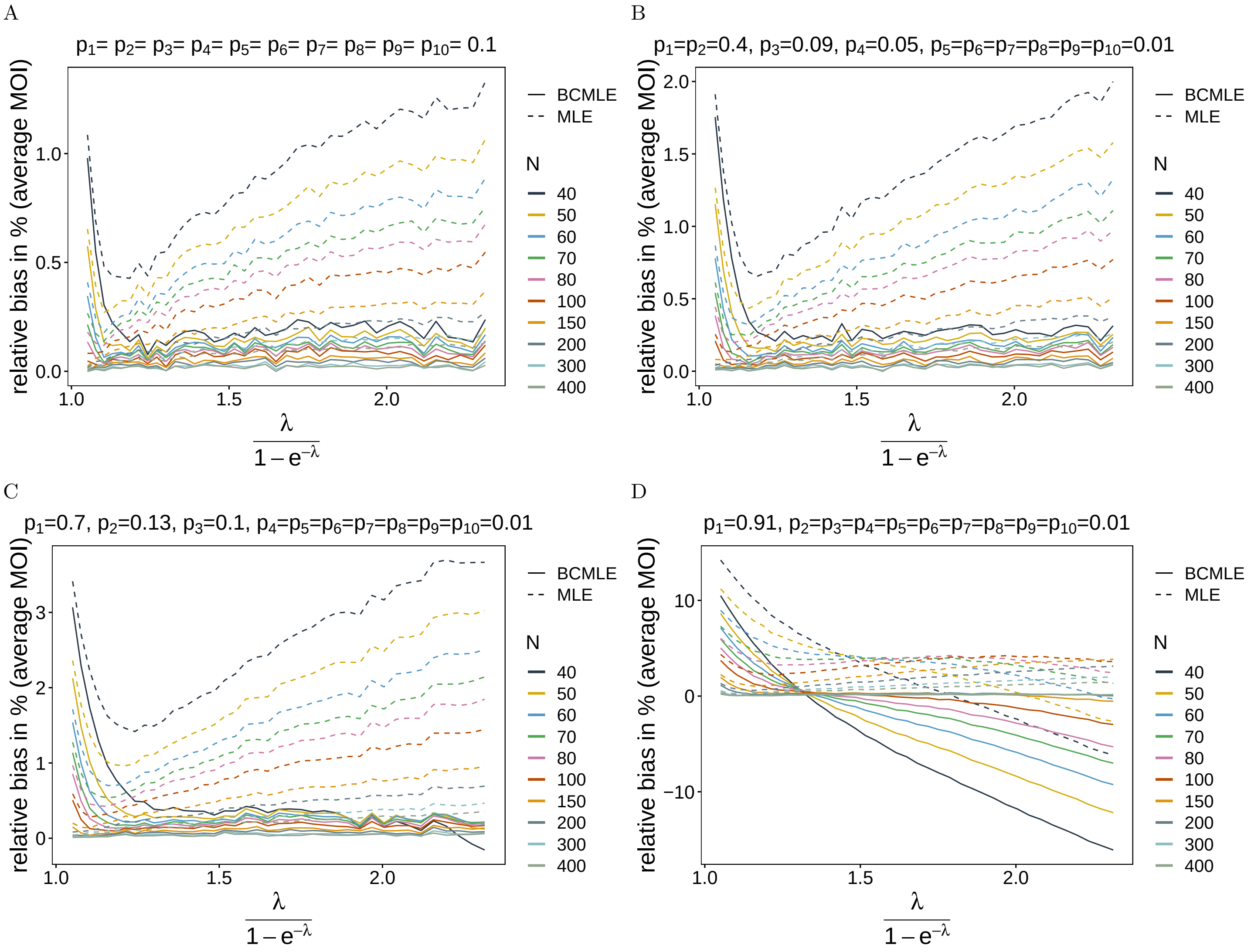

Figure 6: Similar to Figure 1 but for different lineage-frequency distributions.

1.2 CV in %

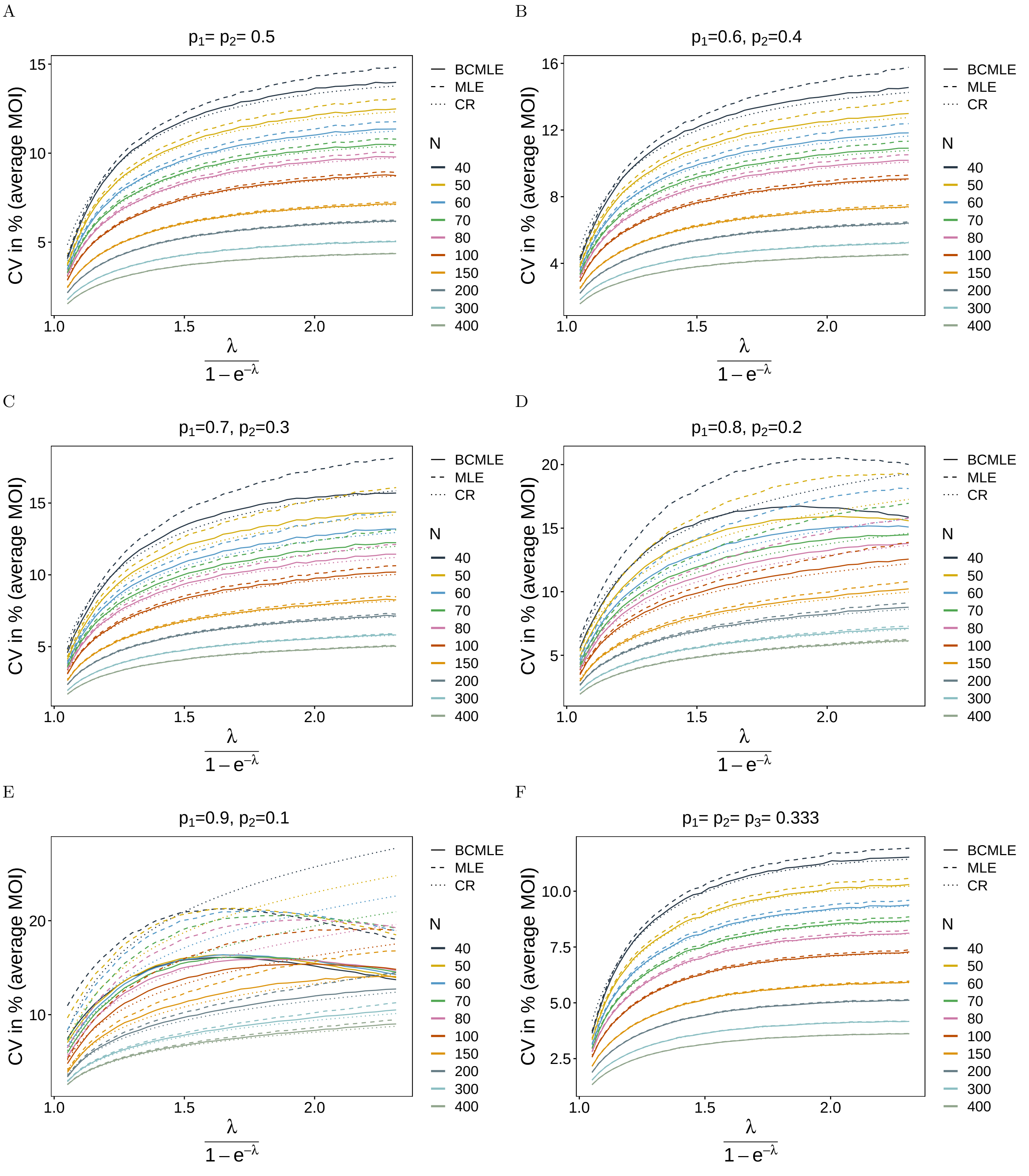

Figure 7: **Variance of MOI estimates**. Similar to Fig 1 but for the coefficient of variation in %. The dotted lines are the respective predictions based on the Cramér-Rao lower bounds.

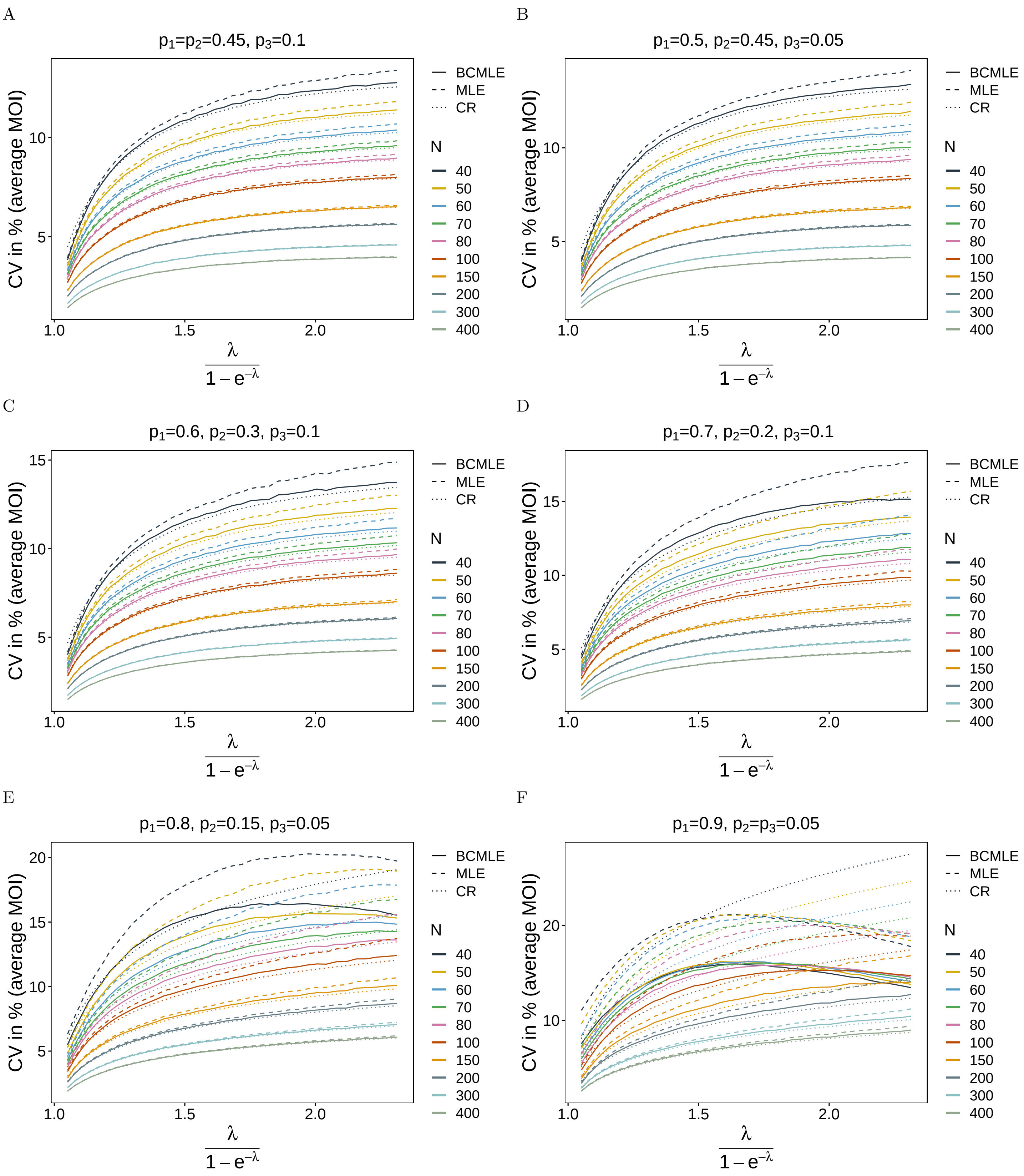

Figure 8: Similar to Figure 7 but for different lineage-frequency distributions.

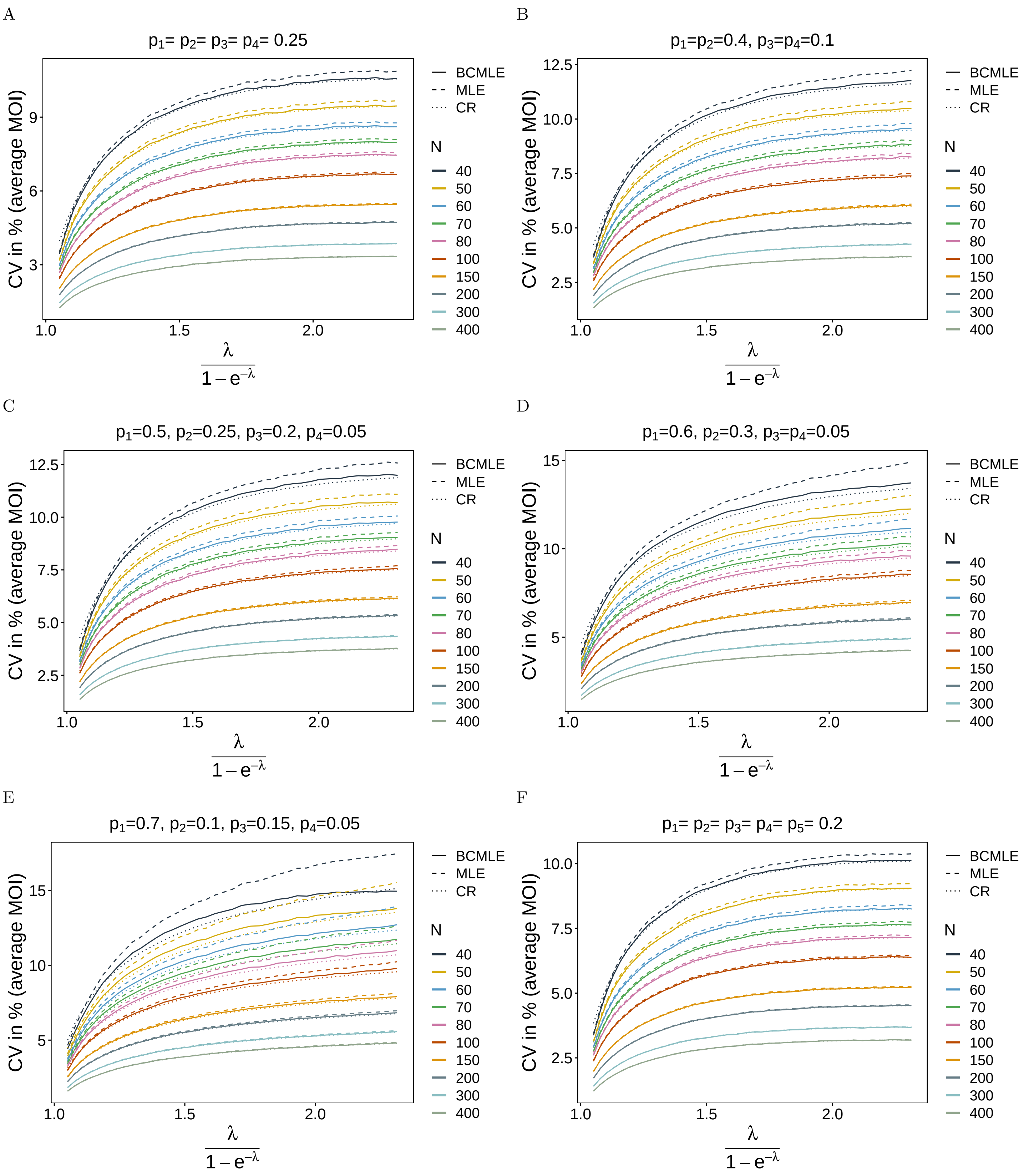

Figure 9: Similar to Figure 7 but for different lineage-frequency distributions.

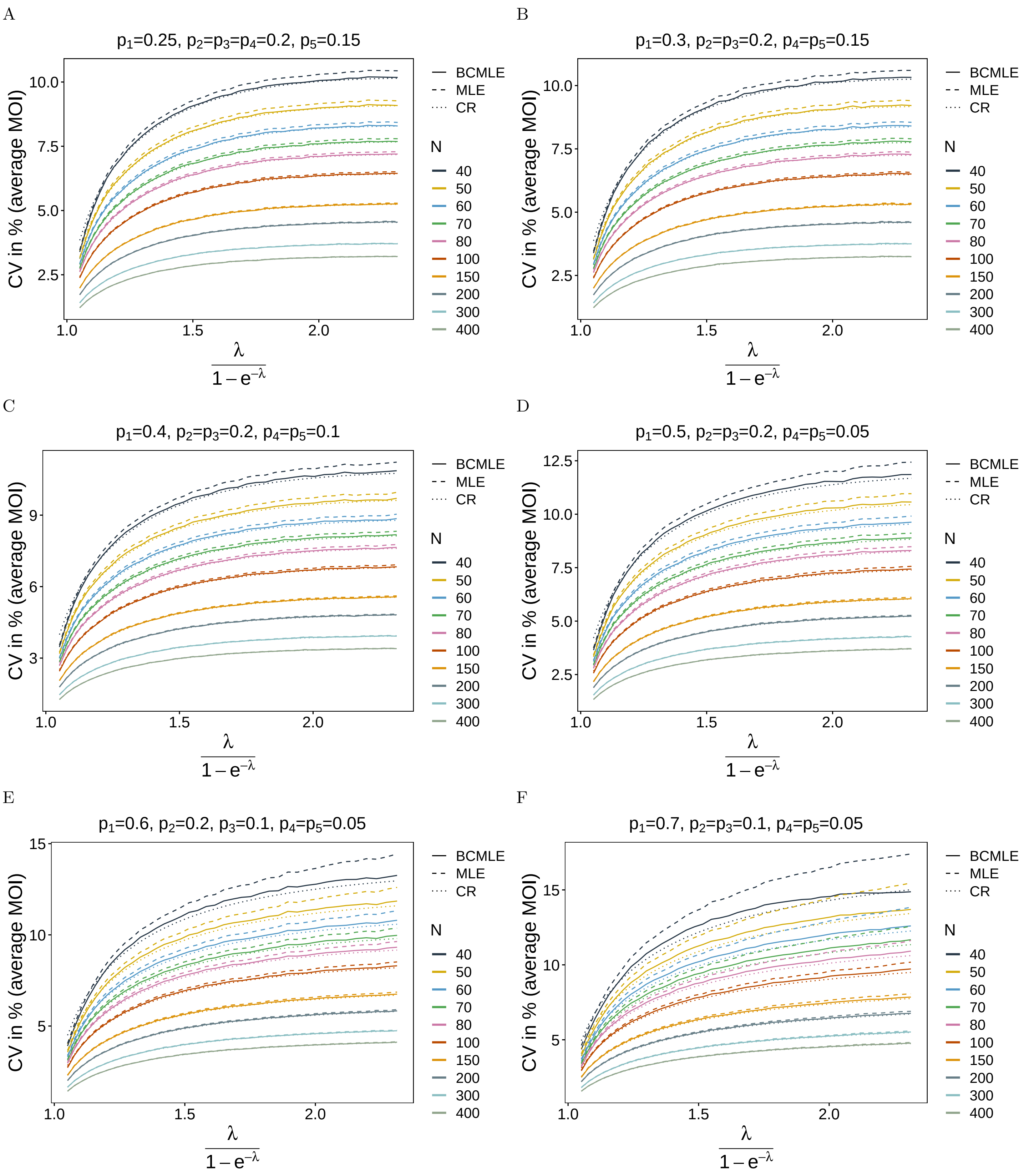

Figure 10: Similar to Figure 7 but for different lineage-frequency distributions.

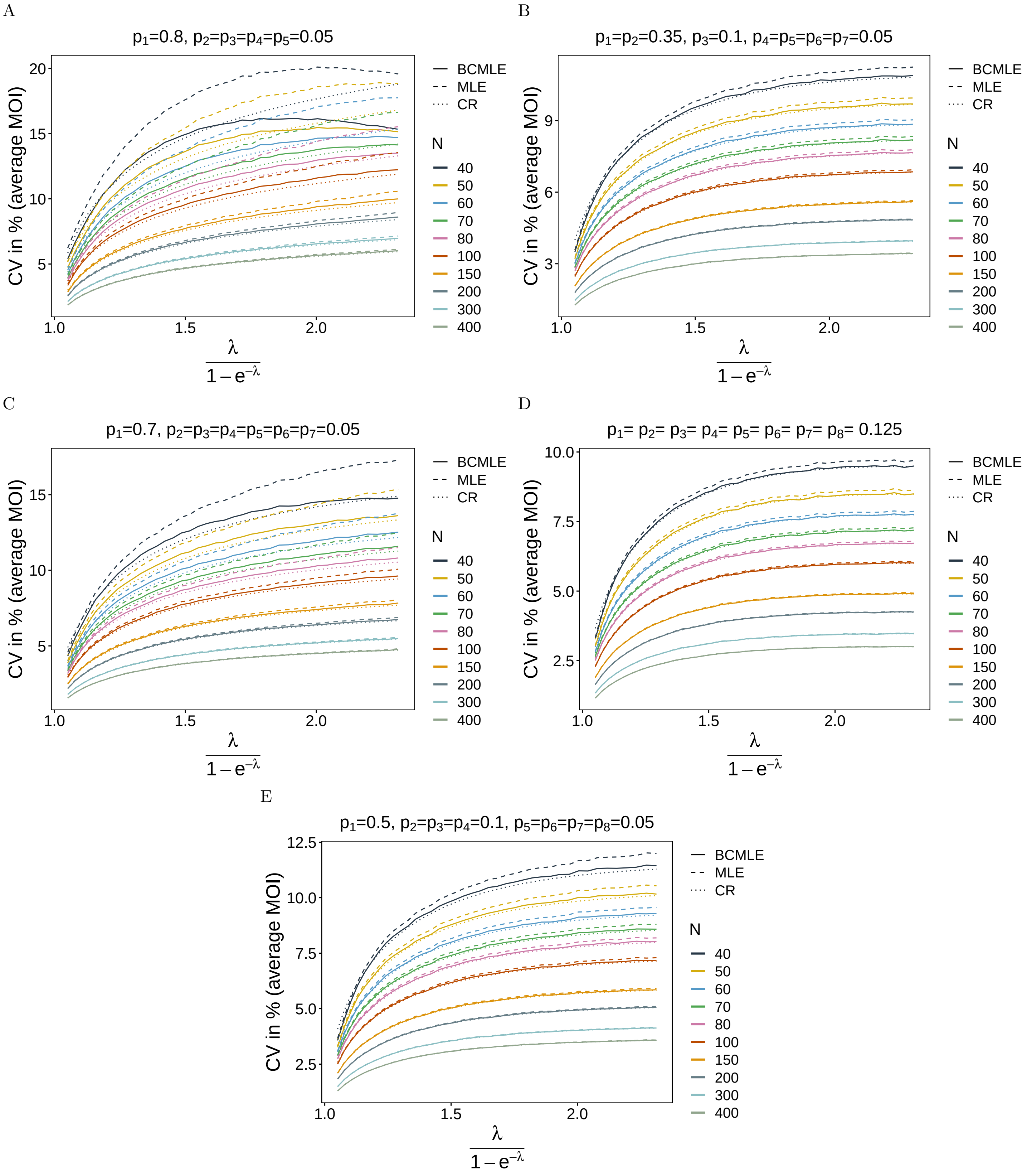

Figure 11: Similar to Figure 7 but for different lineage-frequency distributions.

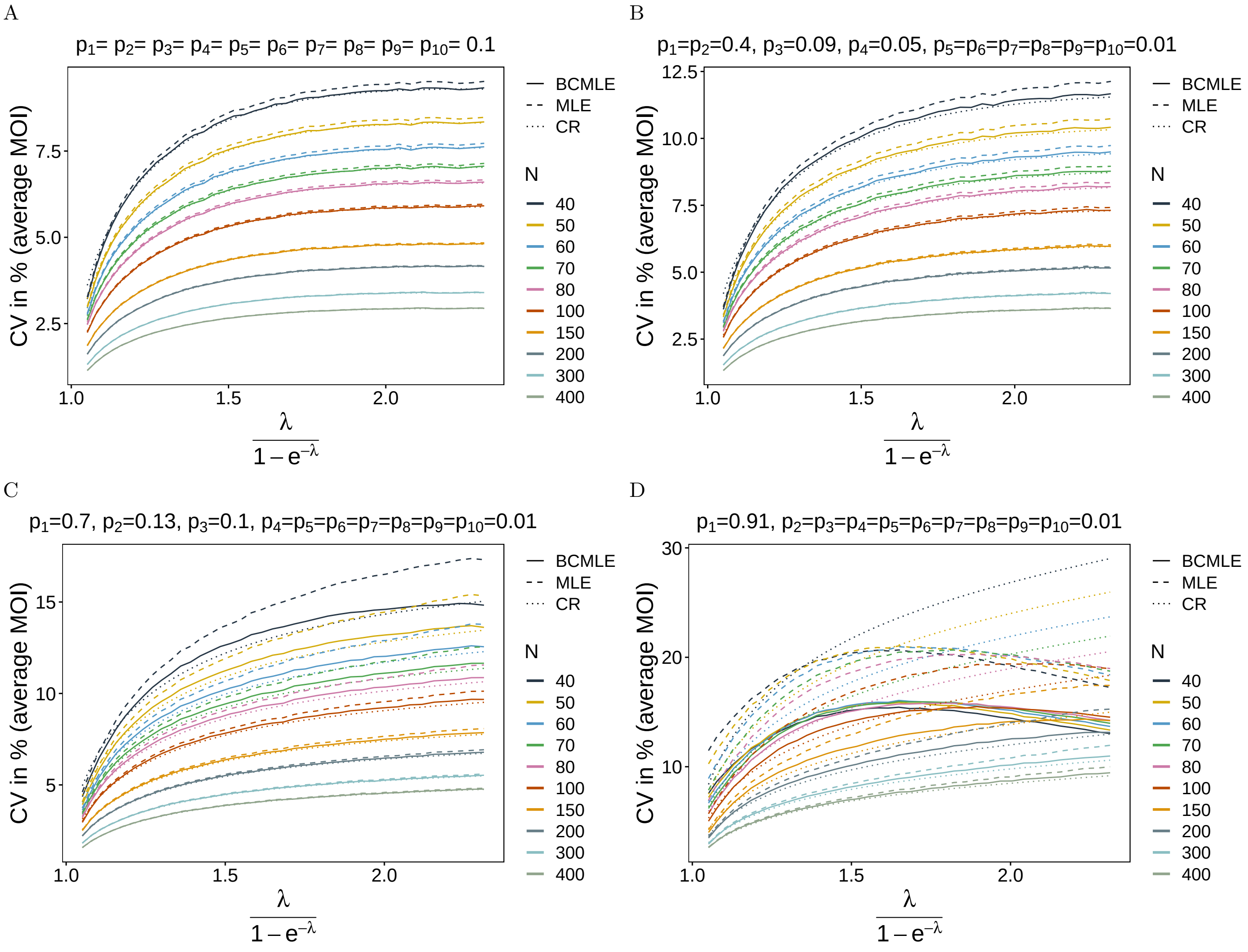

Figure 12: Similar to Figure 7 but for different lineage-frequency distributions.

2 Heuristically adjusted estimates of the average MOI

2.1 Relative bias in %

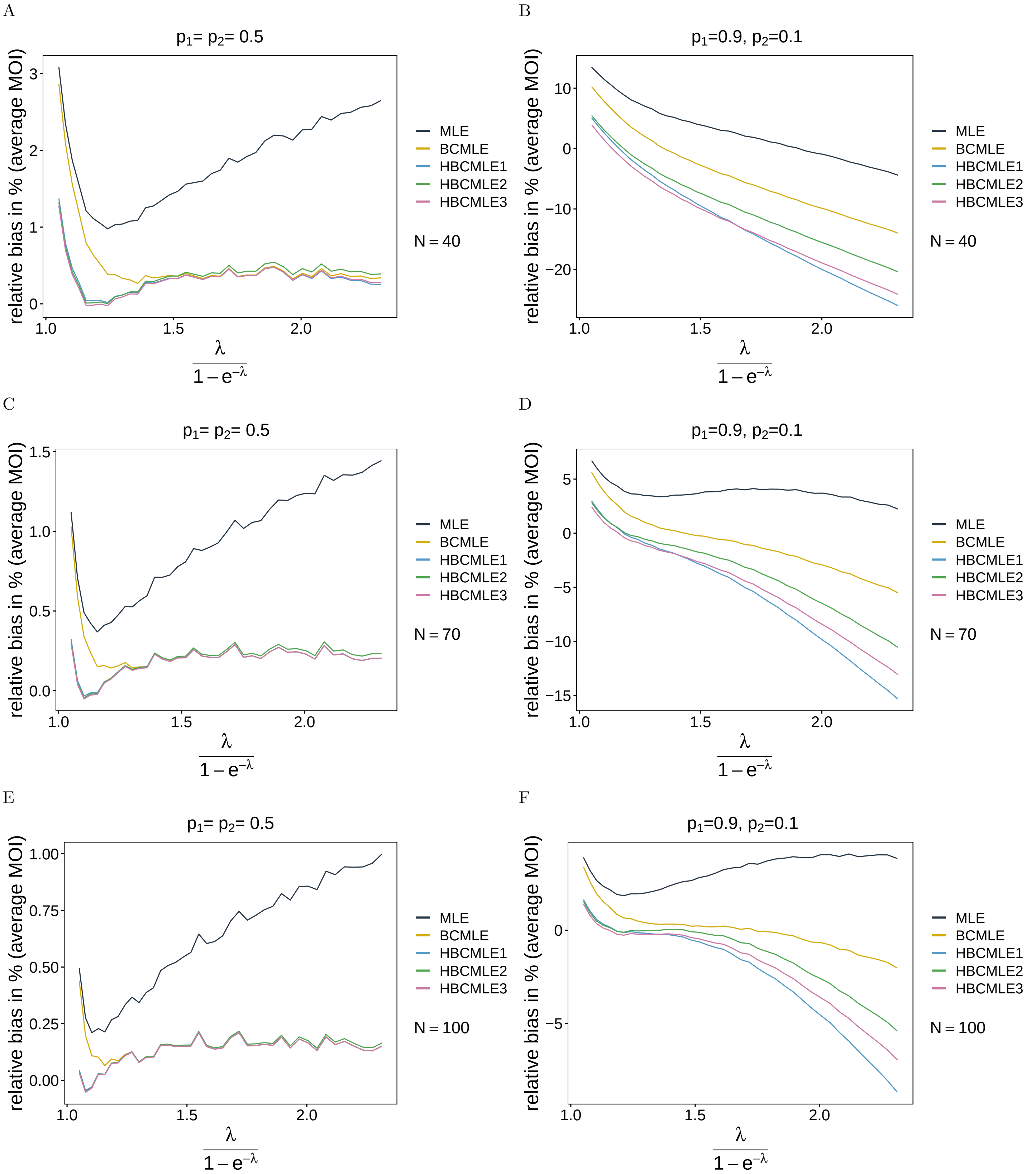

Figure 13: **Bias of heuristically adjusted MOI estimators**. Shown is the relative bias in % of the heuristically adjusted estimators (HBCMLE1 - $\hat{\psi}^{(hbc1)}$, HBCMLE2 - $\hat{\psi}^{(hbc2)}$, HBCMLE3 - $\hat{\psi}^{(hbc3)}$) along with the relative bias in % of the MLE $\hat{\psi}$ and BCMLE $\hat{\psi}^{(\mathrm{bc})}$ as a function of the true parameter ψ based on simulated data created by the conditional Poisson model. Each panel assumes a different lineage-frequency distribution $\boldsymbol{p}$ shown at the top of each panel. Colors correspond to different estimators. The relative bias in each panel is derived from $S = 100,000$ randomly generated datasets of sample size 40.

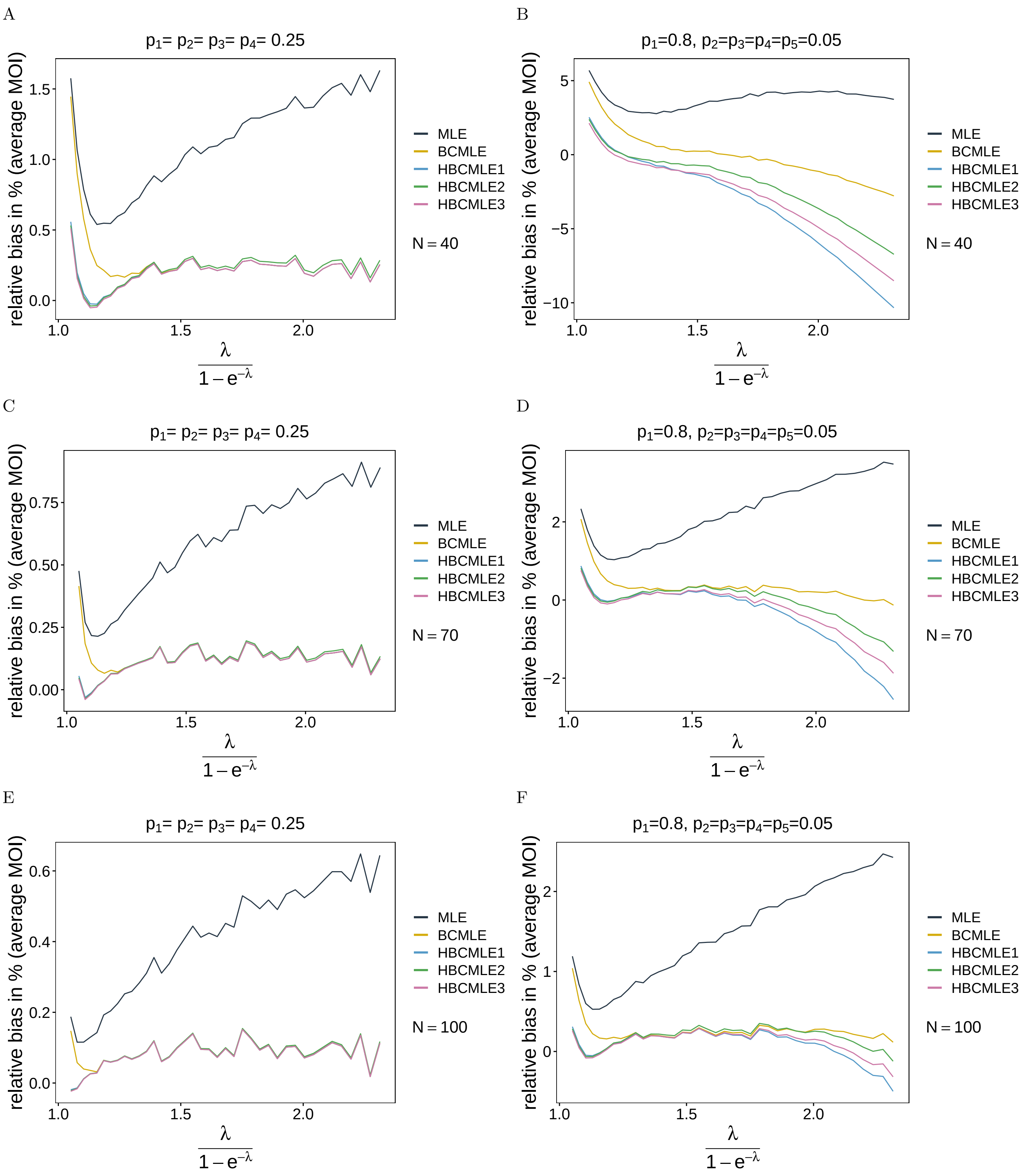

Figure 14: Similar to Figure 13 but for different lineage-frequency distributions.

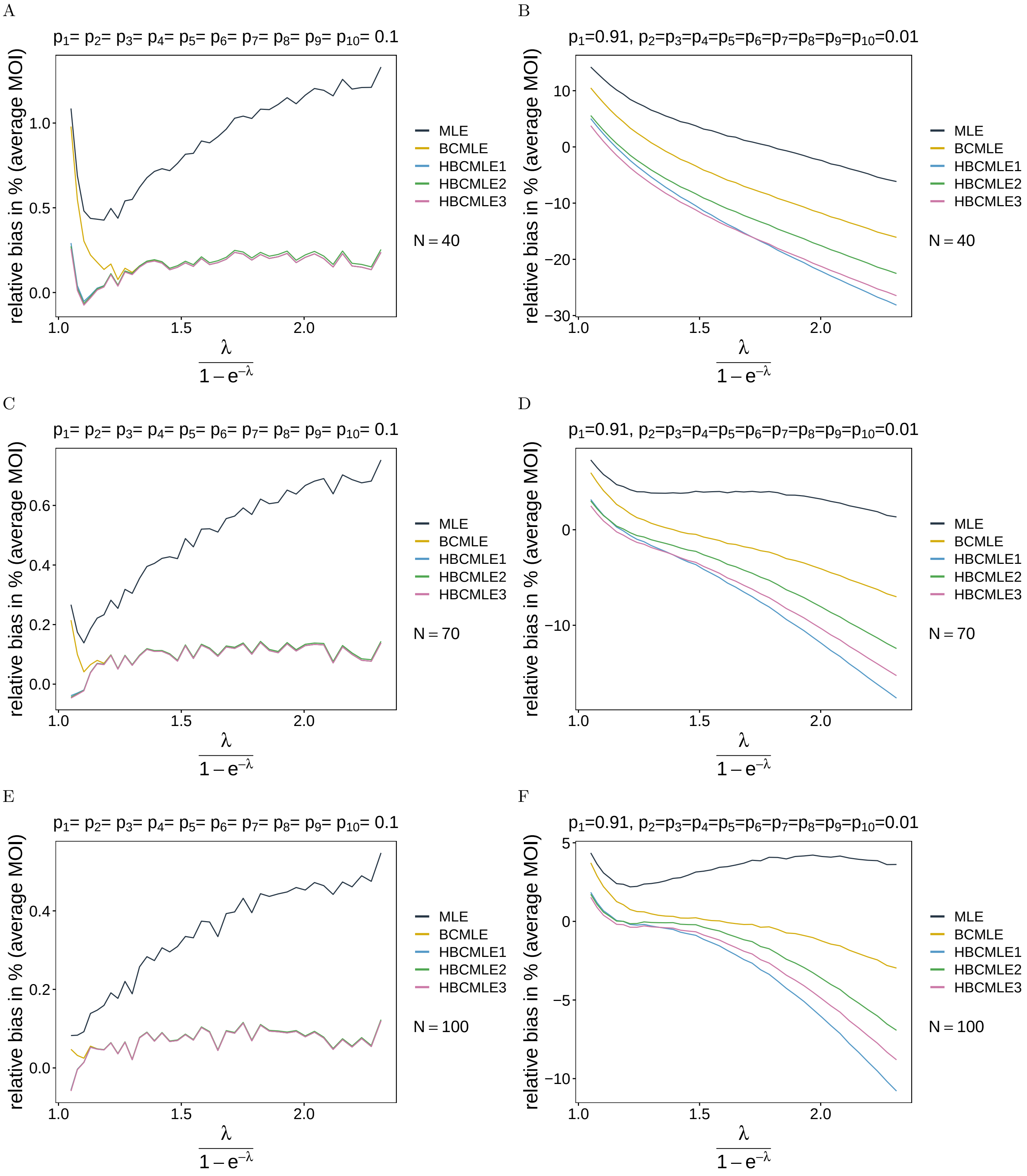

Figure 15: Similar to Figure 13 but for different lineage-frequency distributions.

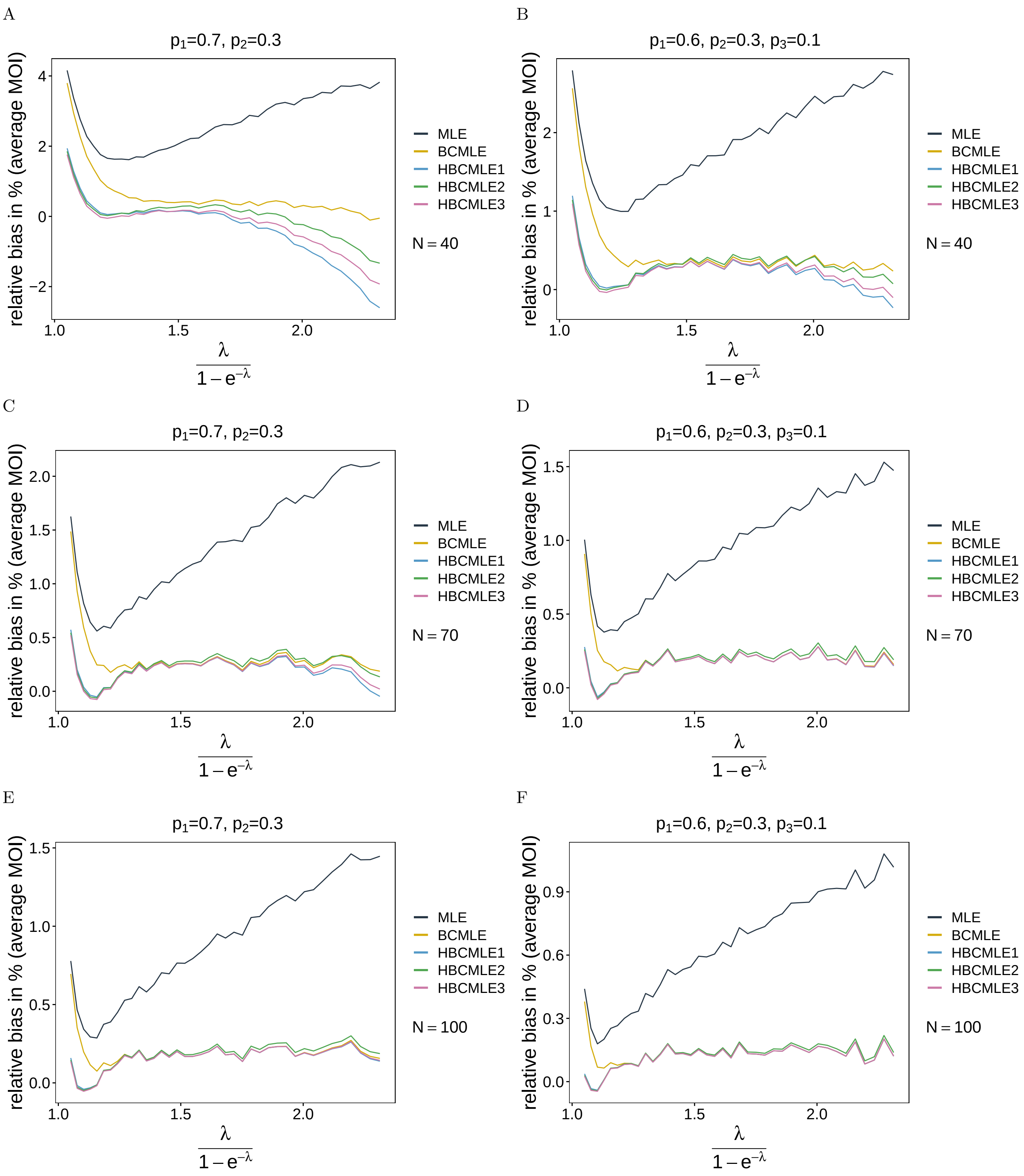

Figure 16: Similar to Figure 13 but for different lineage-frequency distributions.

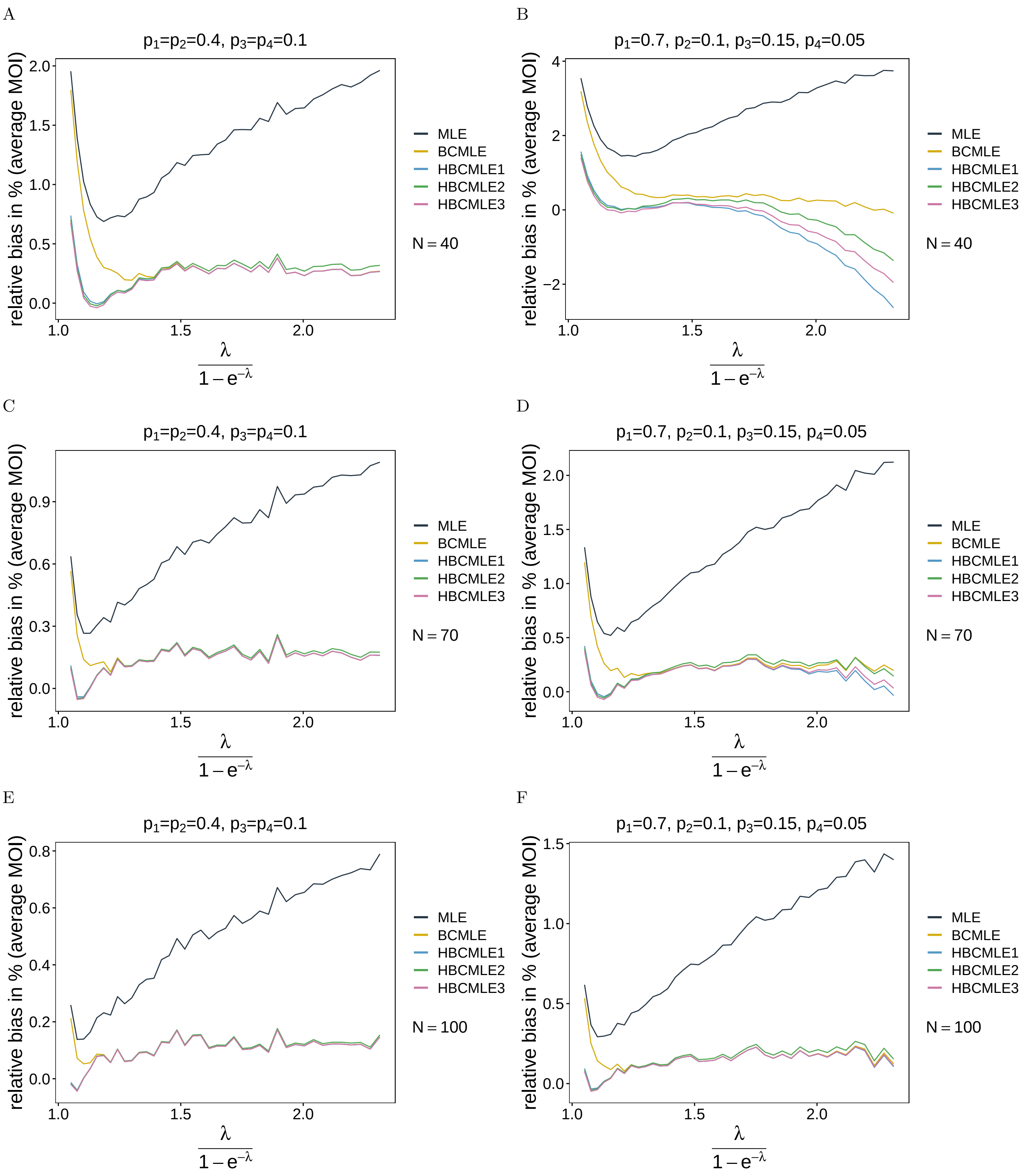

Figure 17: Similar to Figure 13 but for different lineage-frequency distributions.

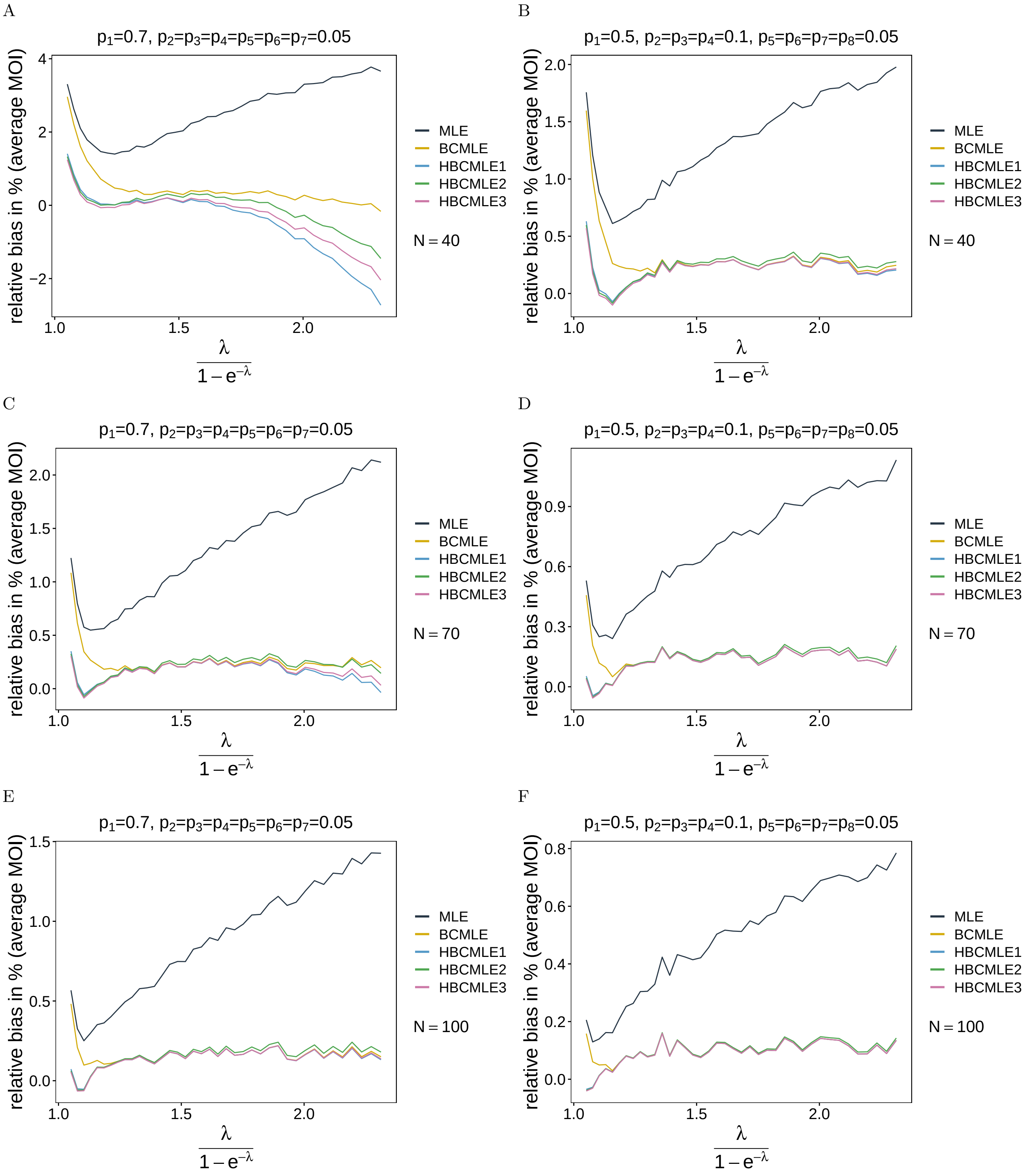

Figure 18: Similar to Figure 13 but for different lineage-frequency distributions.

2.2 CV in %

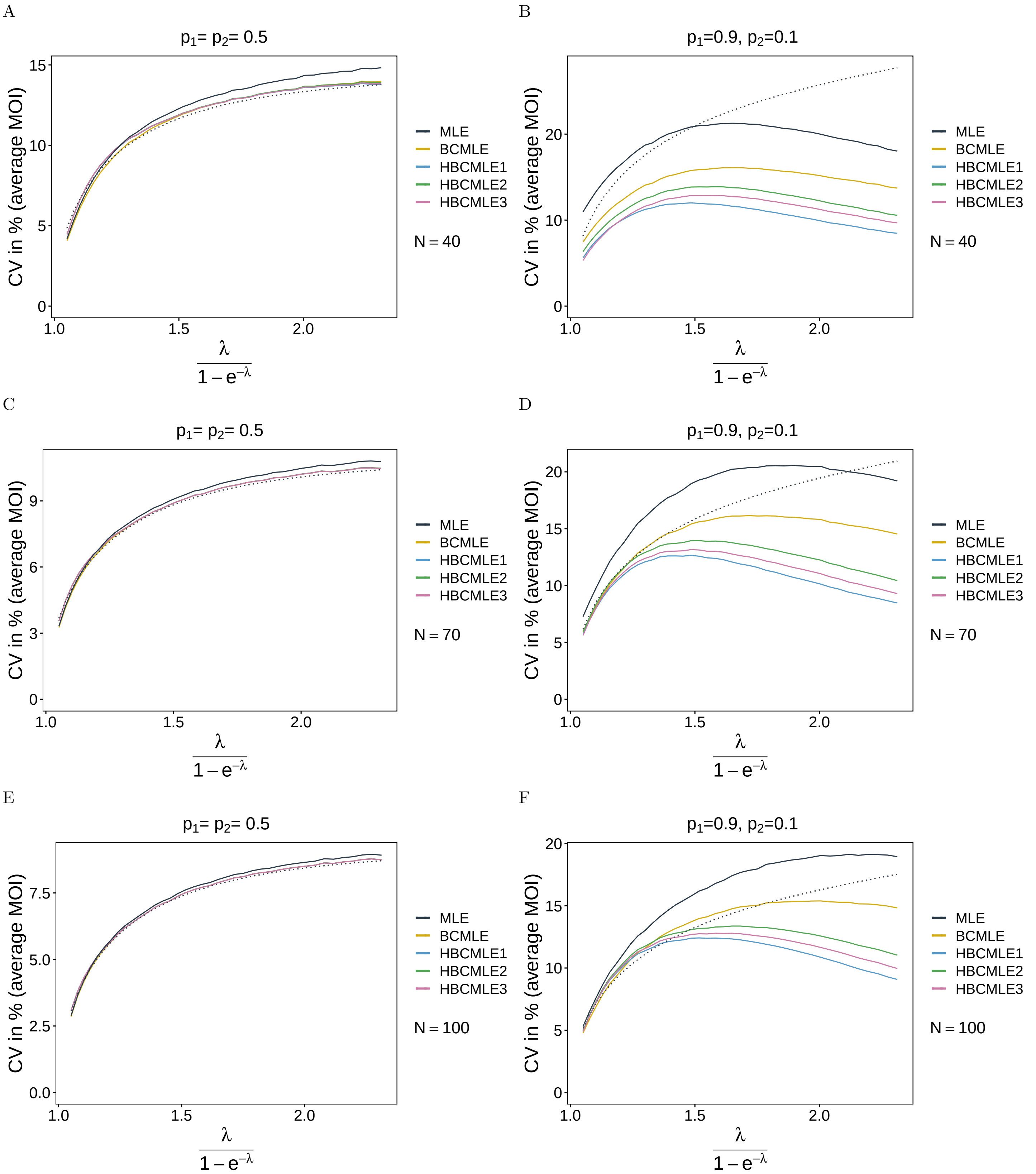

Figure 19: **Variance of heuristically adjusted MOI estimators.** Similar to Fig 13 but for the coefficient of variation in %. The dotted lines are the respective predictions based on the Cramér-Rao lower bounds.

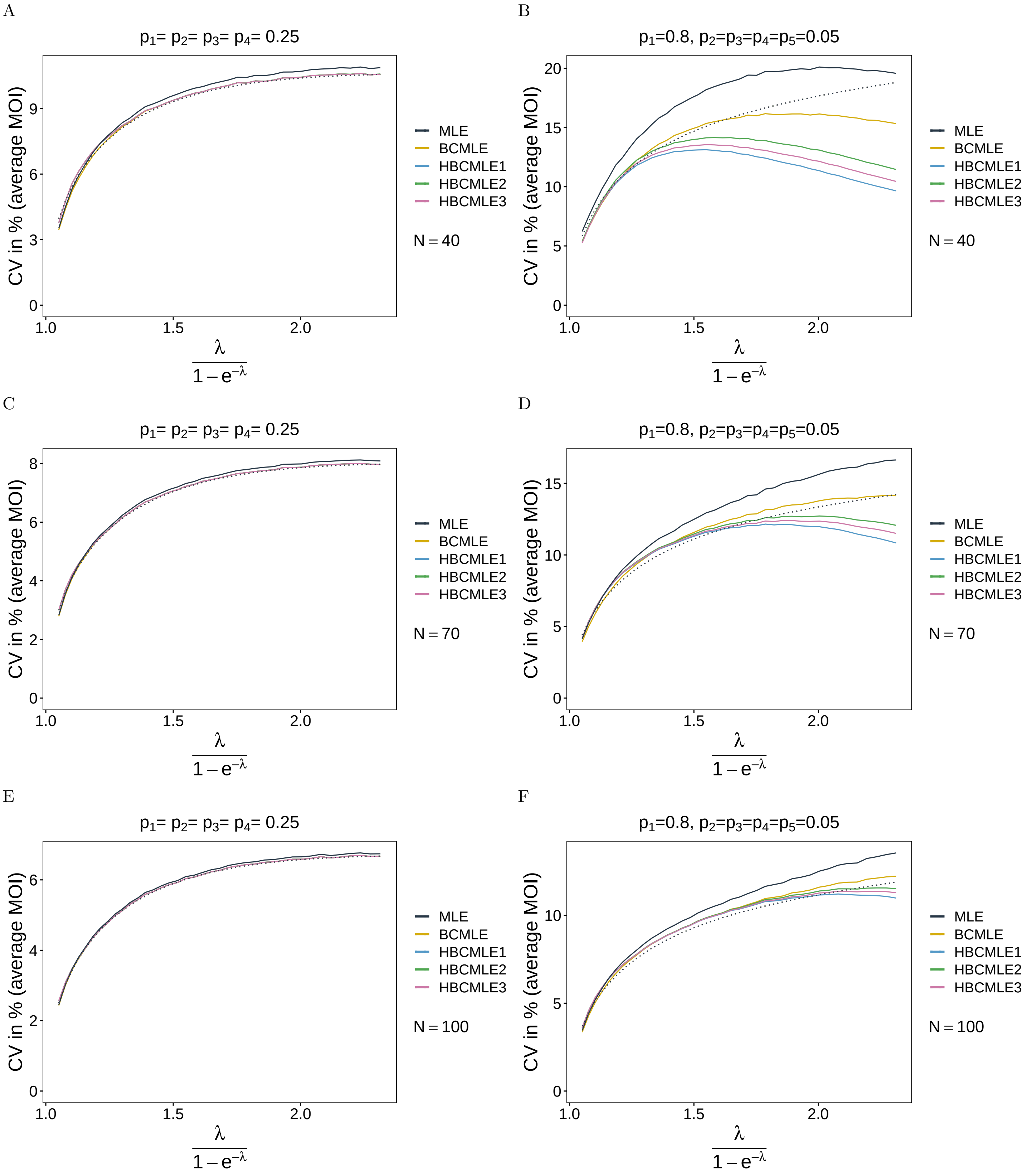

Figure 20: Similar to Figure 19 but for different lineage-frequency distributions.

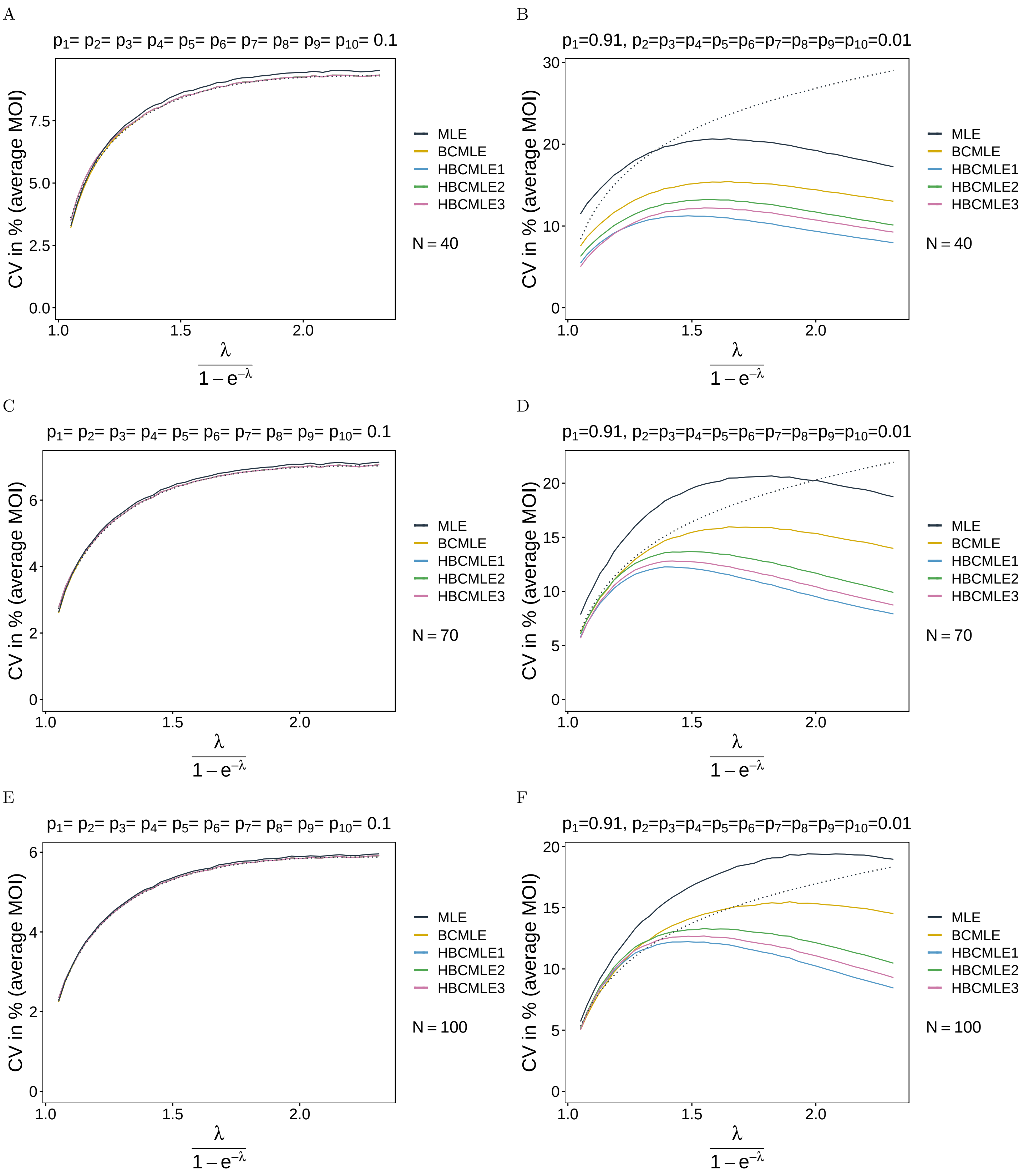

Figure 21: Similar to Figure 19 but for different lineage-frequency distributions.

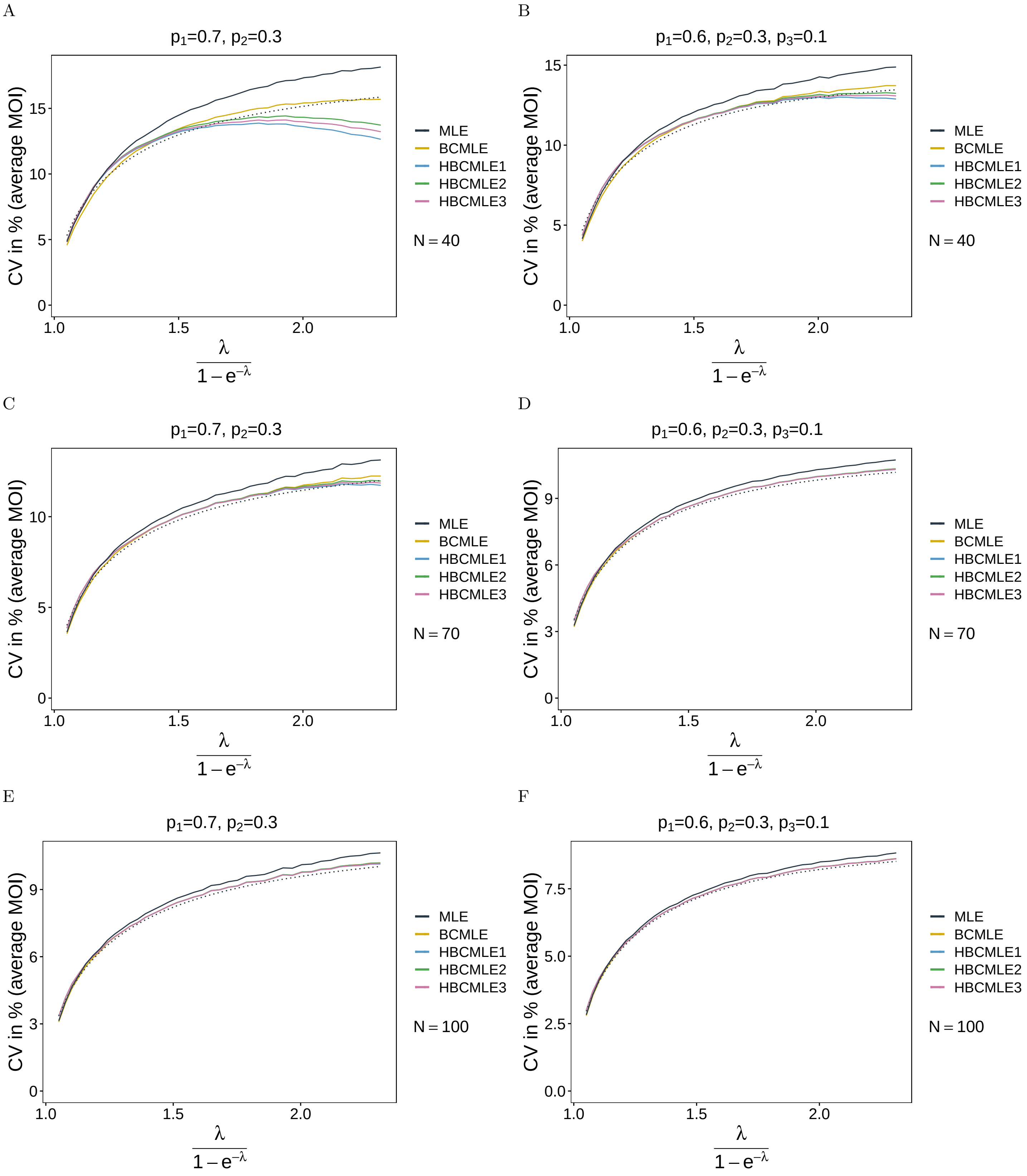

Figure 22: Similar to Figure 19 but for different lineage-frequency distributions.

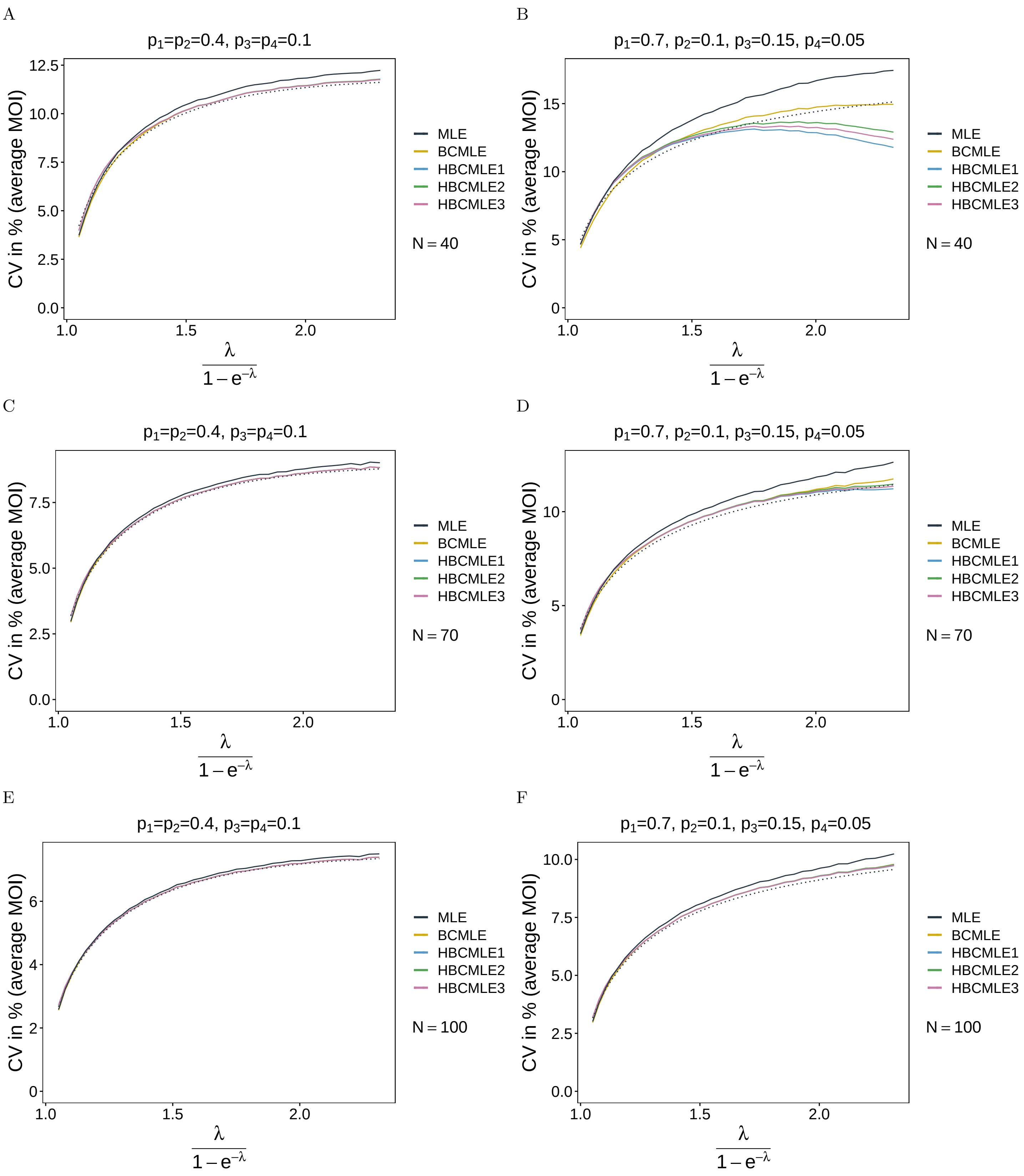

Figure 23: Similar to Figure 19 but for different lineage-frequency distributions.

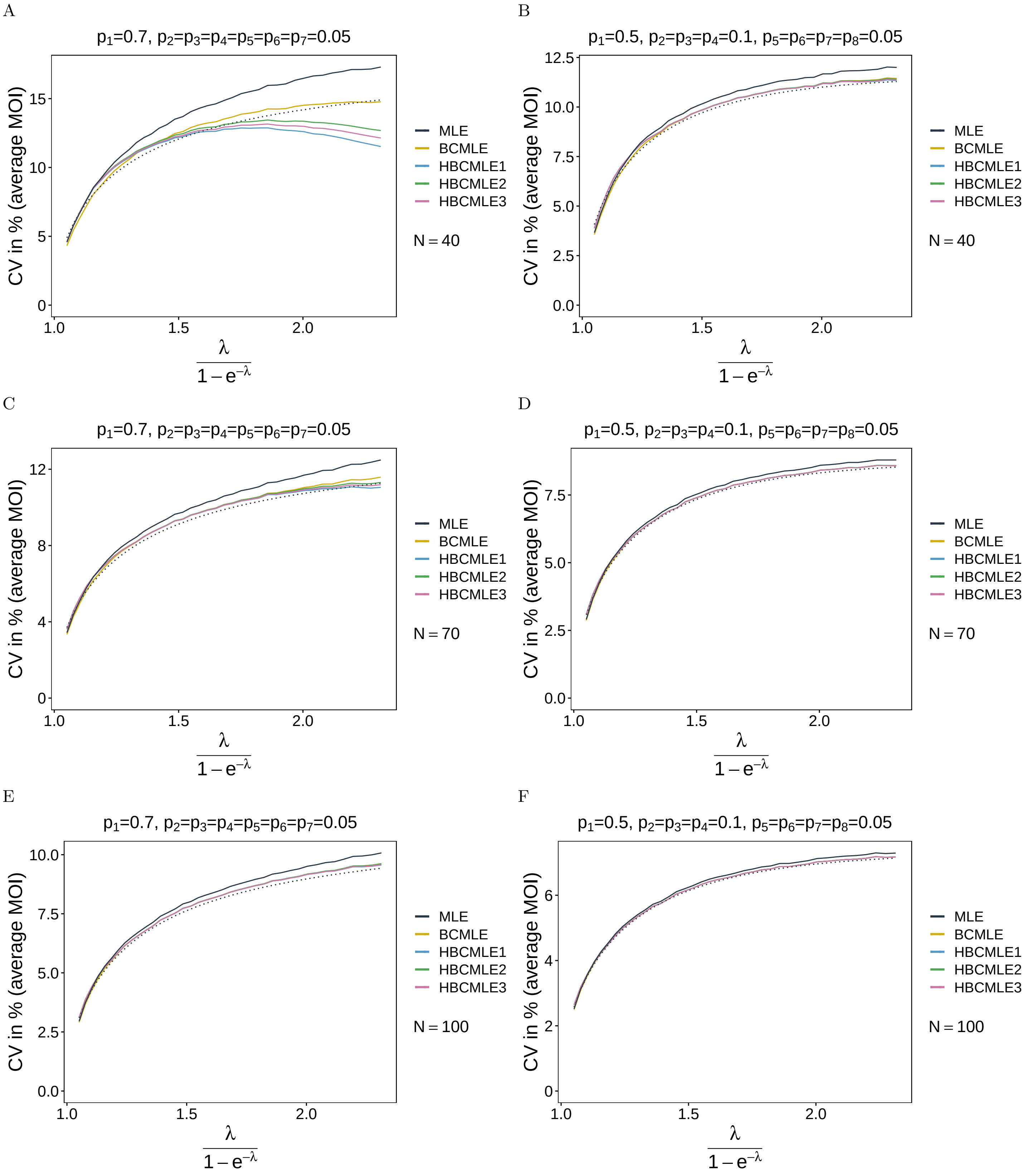

Figure 24: Similar to Figure 19 but for different lineage-frequency distributions.

3 Probability of regular data

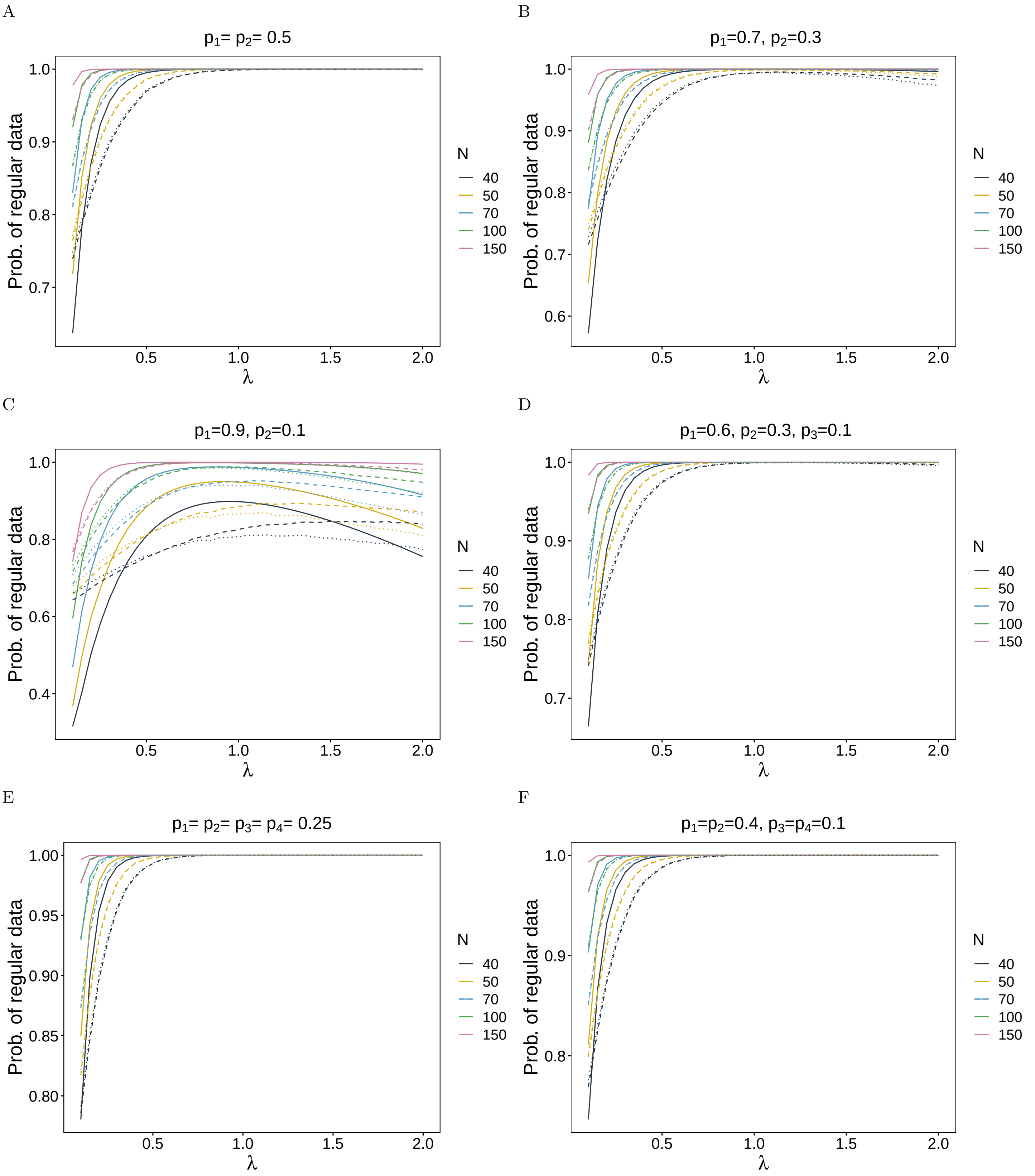

Figure 25: **Probability of regular data**. The figure illustrates the probability of regular data $1-q$ as a function of the true parameter λ. The solid lines are the respective $1-q$ at the true parameter $\boldsymbol{\theta} = (\lambda, \boldsymbol{p})$. The figure also shows the empirical mean of $1-\hat{q}^{(bc)}$ the probability of regular data evaluated at the BCMLE (dotted lines) and the empirical mean of $1-\hat{q}$, the probability of regular data evaluated at the MLE (dashed lines) from $S = 10,000$ generated datasets created by the conditional Poisson model. Each panel assumes a different lineage-frequency distribution $\boldsymbol{p}$ shown at the top of each panel. Each colored line corresponds to a different sample size.

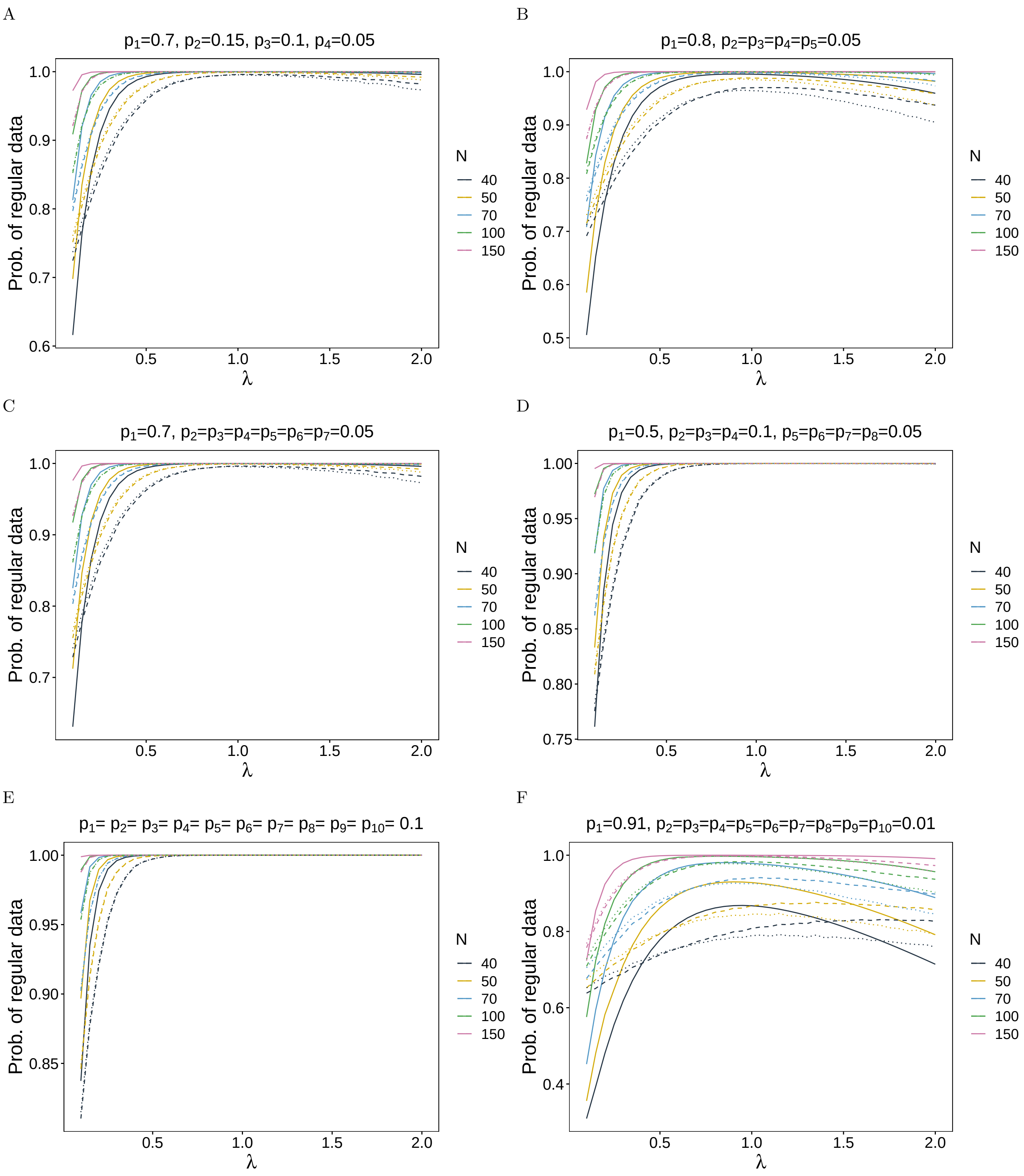

Figure 26: Similar to Figure 25 but for different lineage-frequency distributions.

4 The BCMLE of lineage frequencies

4.1 Relative bias in %

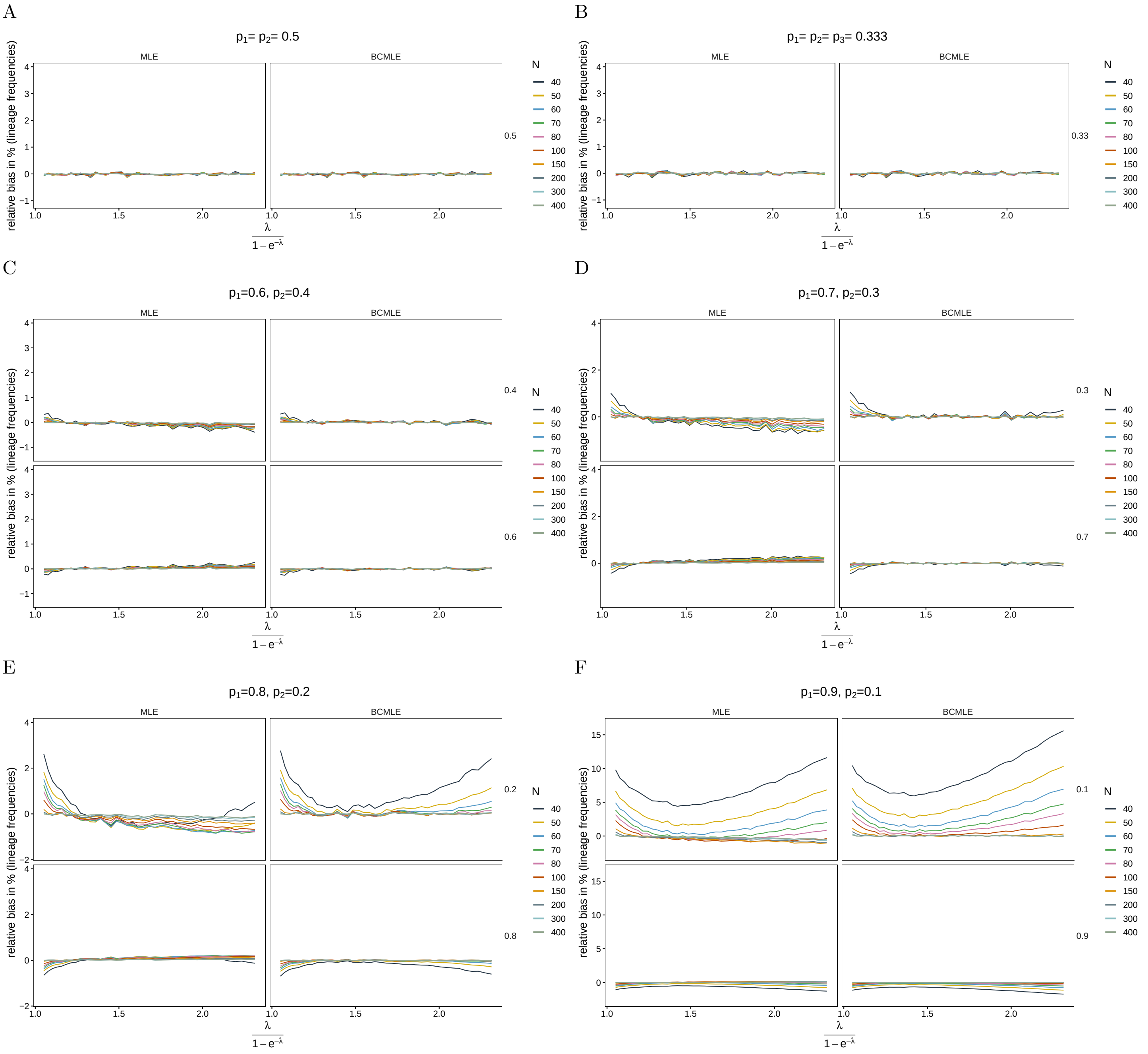

Figure 27: **Bias of lineage-frequency estimates**. The figure shows the relative bias in % of the BCMLE (plots on the right in each panel) and the MLE (plots on the left in each panel) of lineage frequencies $\boldsymbol{p}$ as a function of the true parameter ψ based on simulated data created by the conditional Poisson model. Each panel assumes a different lineage-frequency distribution $\boldsymbol{p}$ shown at the top of each panel. In panel **A**, the relative bias in % of only one lineage frequency is illustrated, because all lineage frequencies are equal and their relative bias is almost identical. Different rows in panel **B** correspond to different lineage frequencies. Each colored line corresponds to a different sample size N.

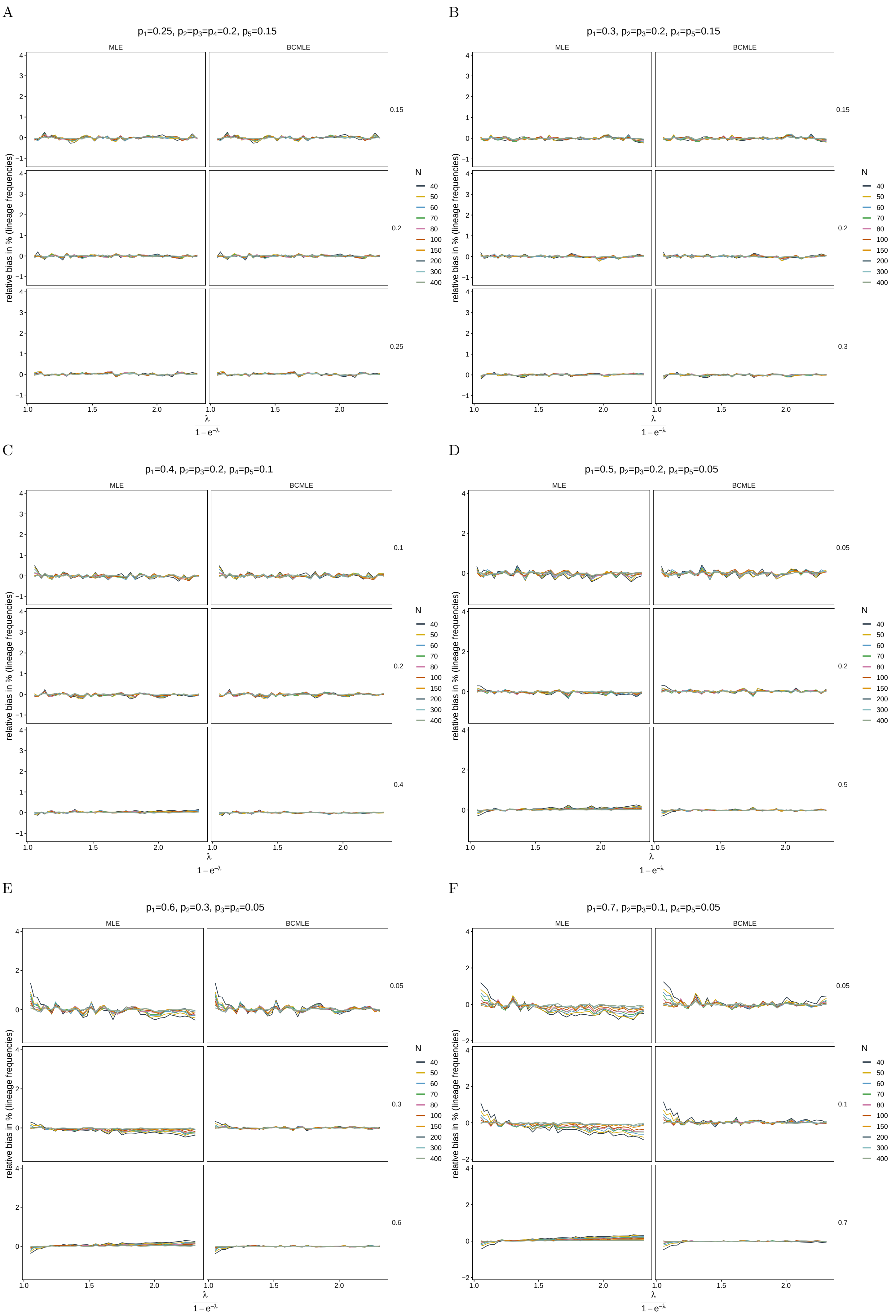

Figure 28: Similar to Figure 27 but for different lineage-frequency distributions.

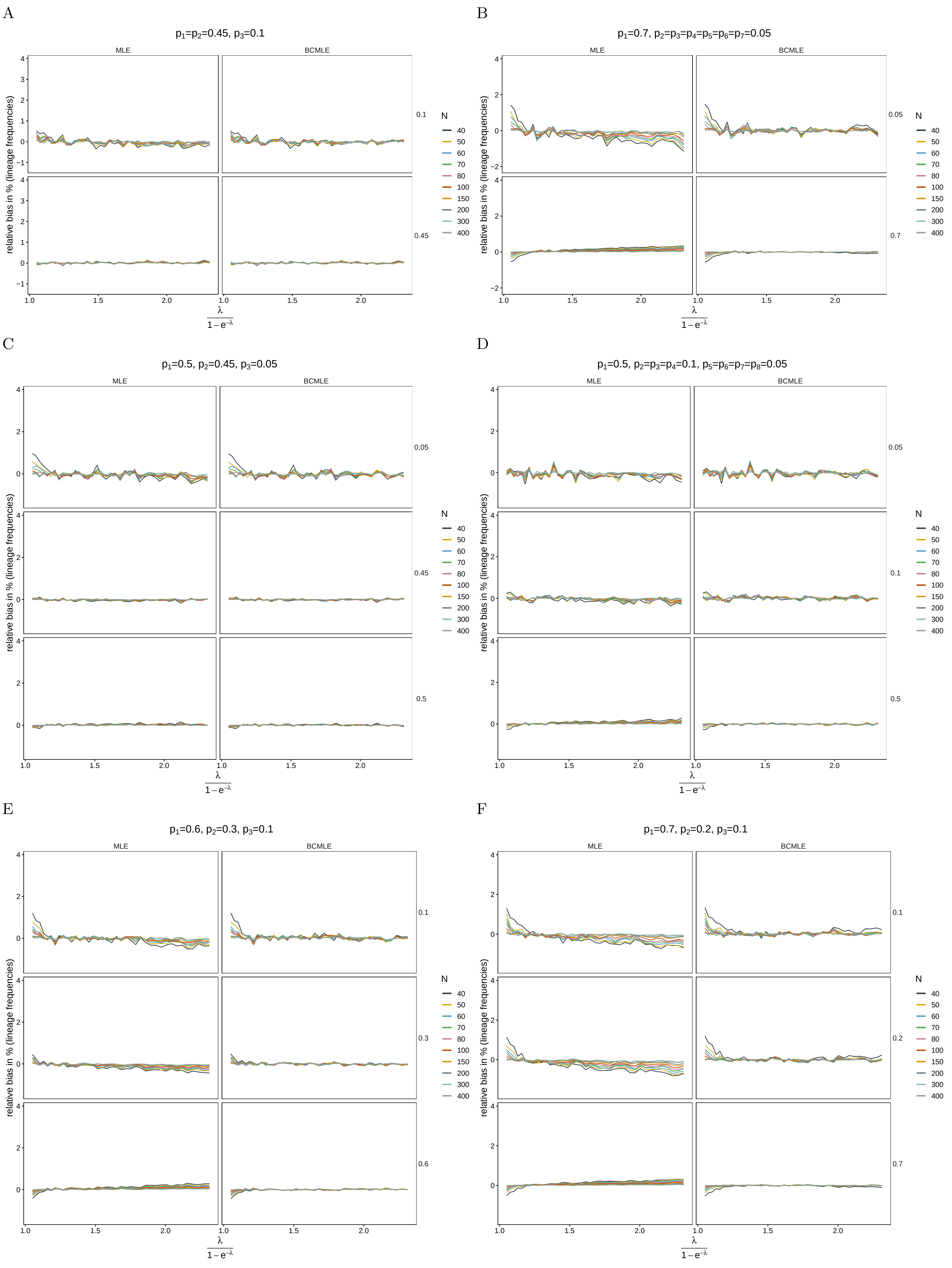

Figure 29: Similar to Figure 27 but for different lineage-frequency distributions.

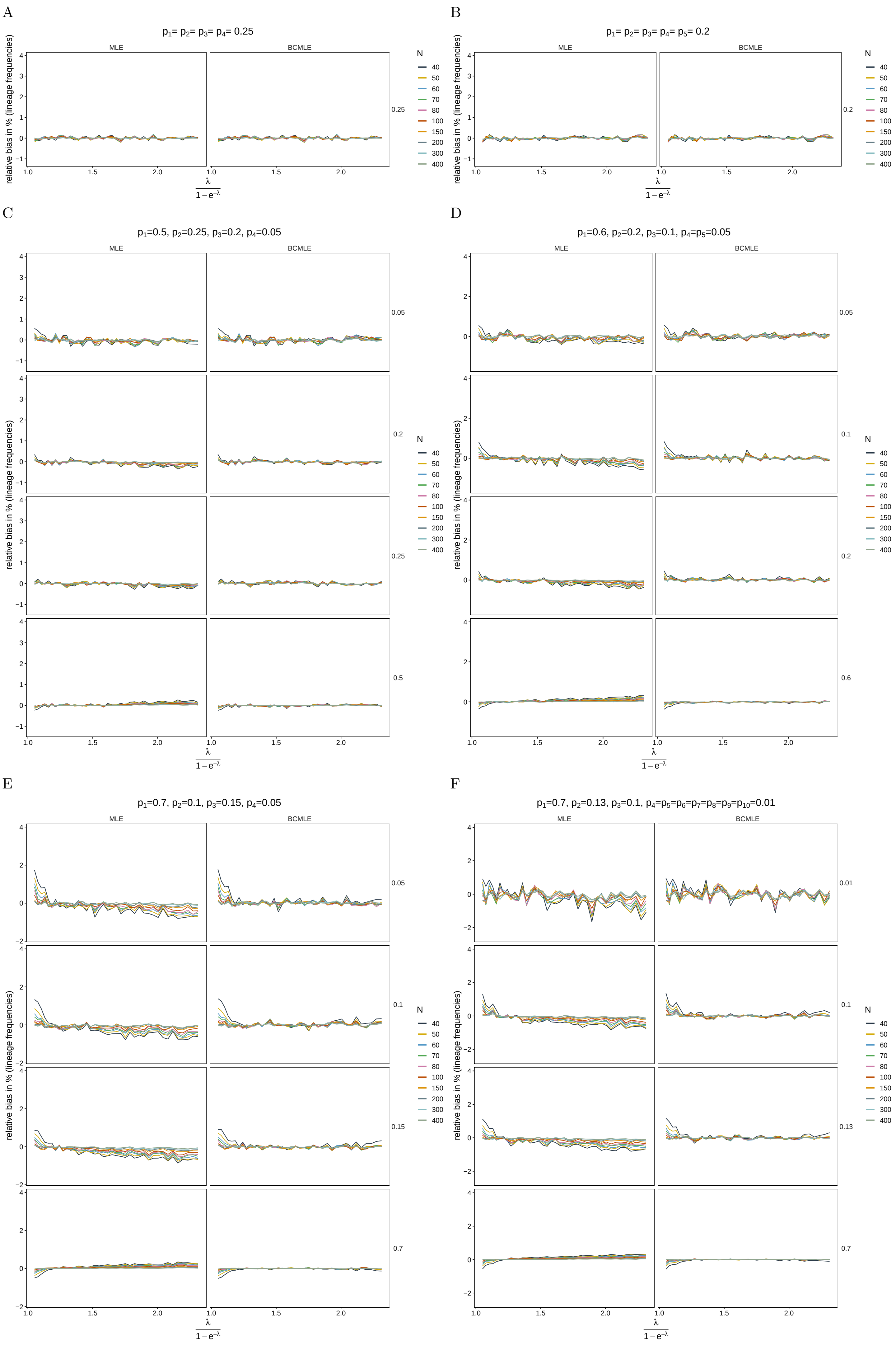

Figure 30: Similar to Figure 27 but for different lineage-frequency distributions.

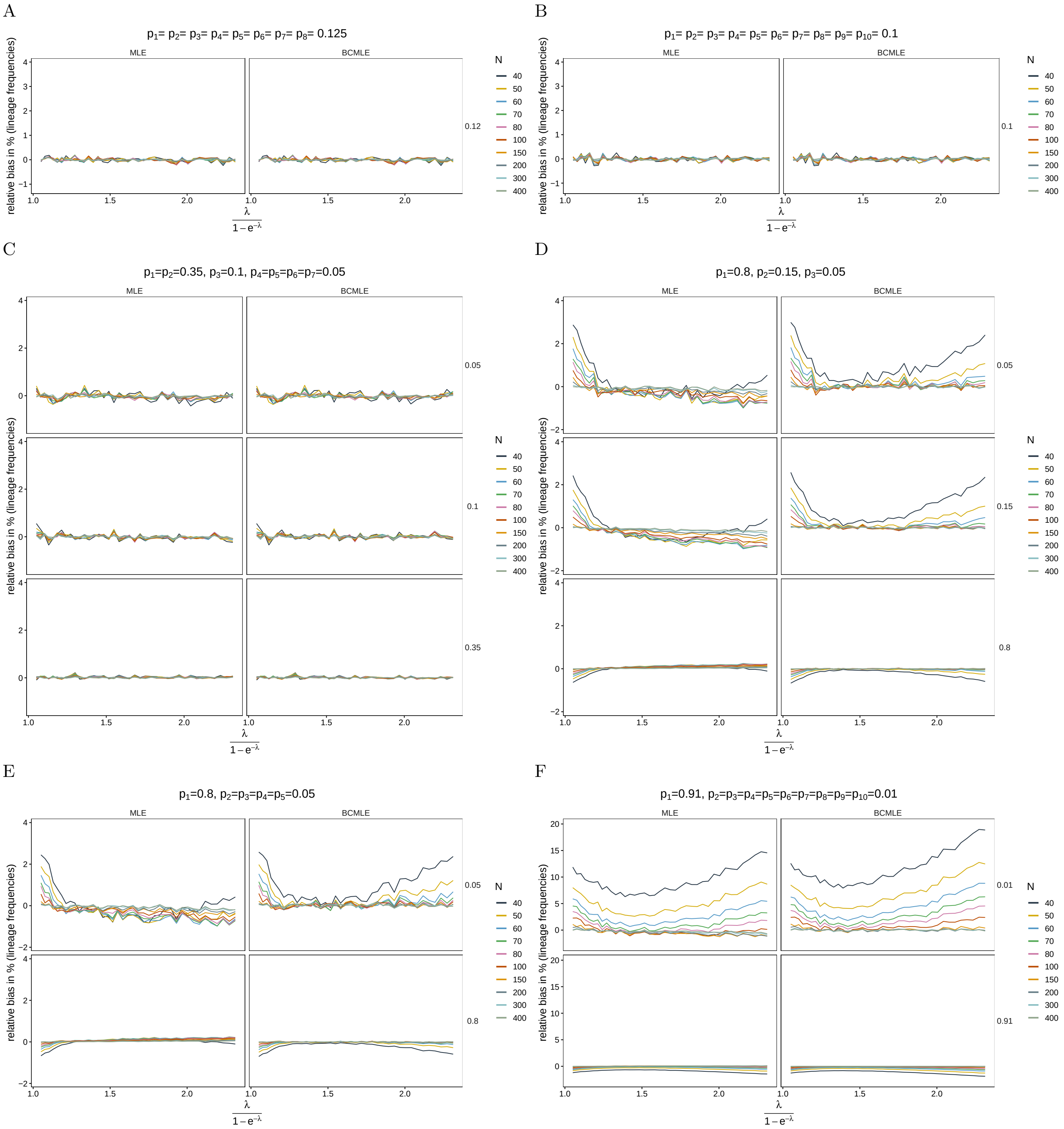

Figure 31: Similar to Figure 27 but for different lineage-frequency distributions.

4.2 CV in %

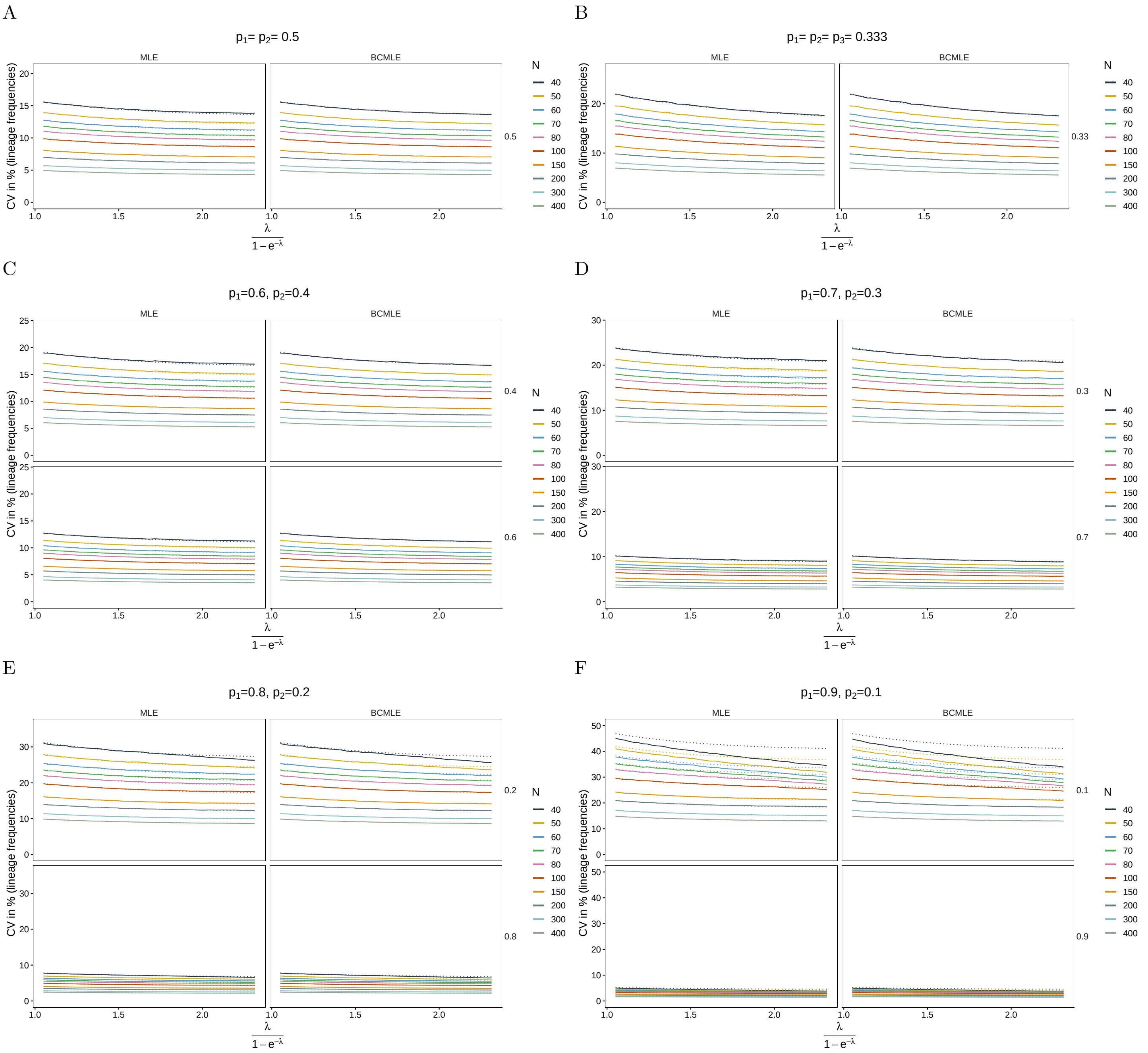

Figure 32: **Variance of lineage frequency estimates**. Similar to Fig 27 but for the coefficient of variation in %. The dotted lines are the respective predictions based on the Cramér-Rao lower bounds.

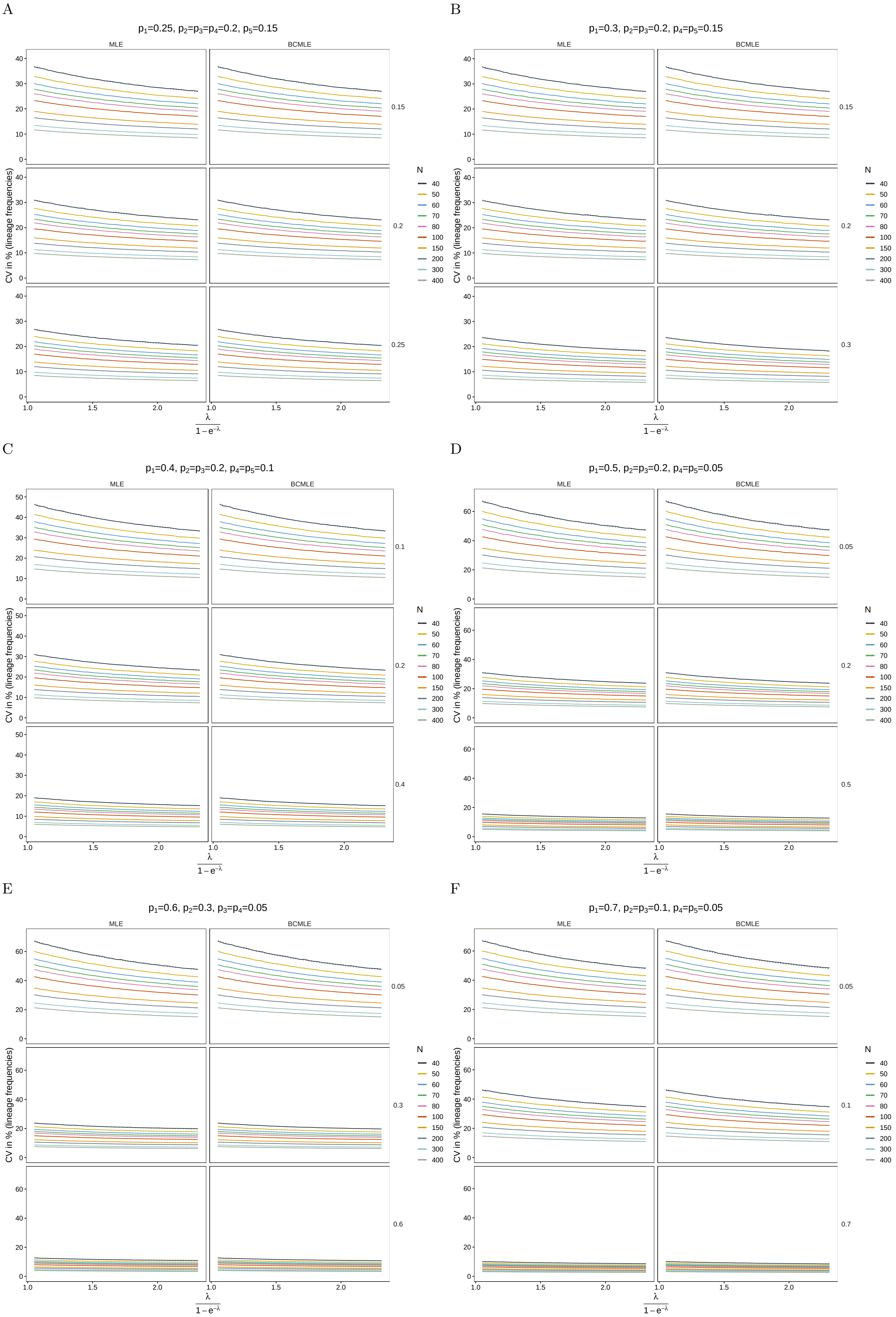

Figure 33: Similar to Figure 32 but for different lineage-frequency distributions.

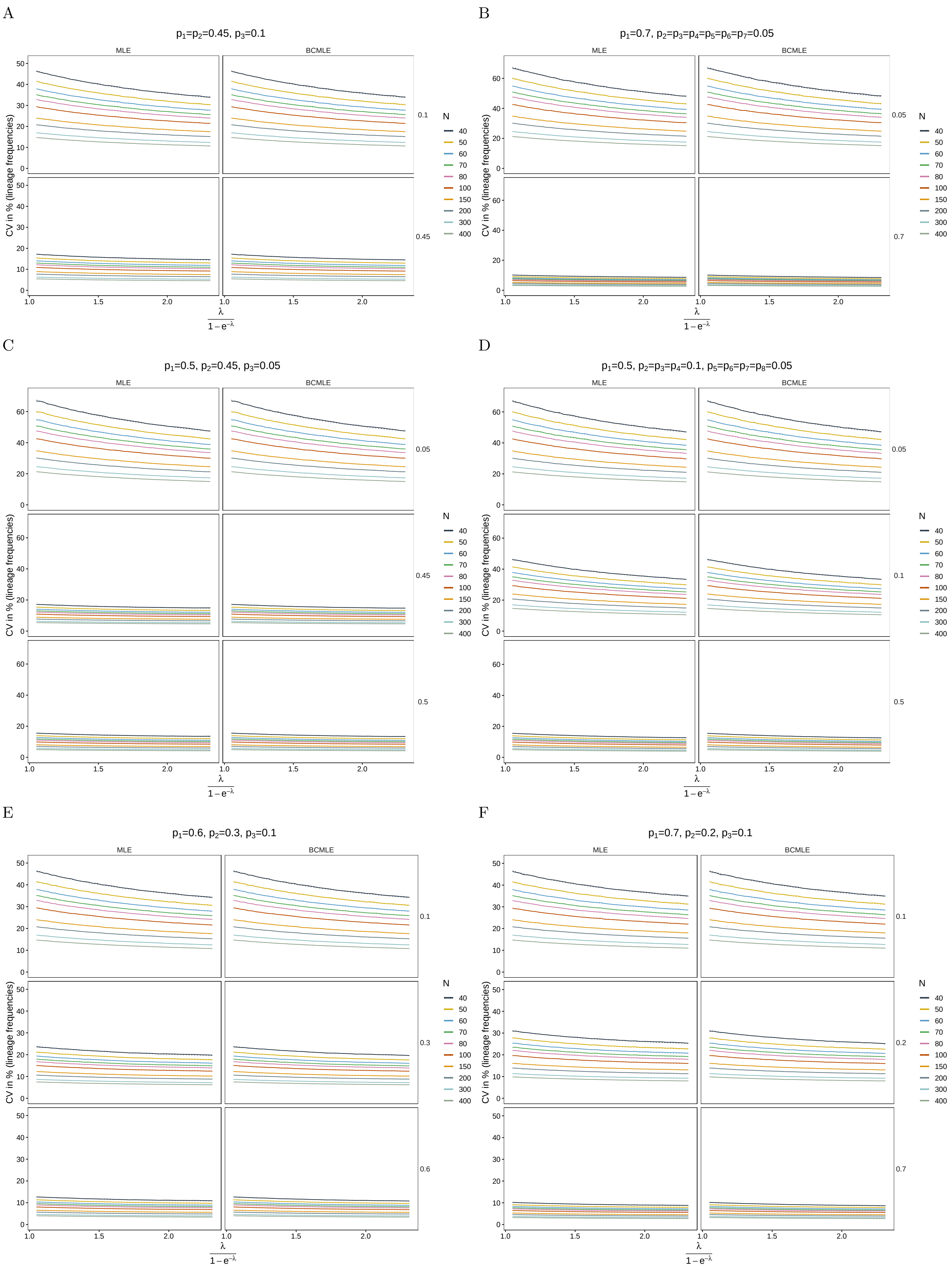

Figure 34: Similar to Figure 32 but for different lineage-frequency distributions.

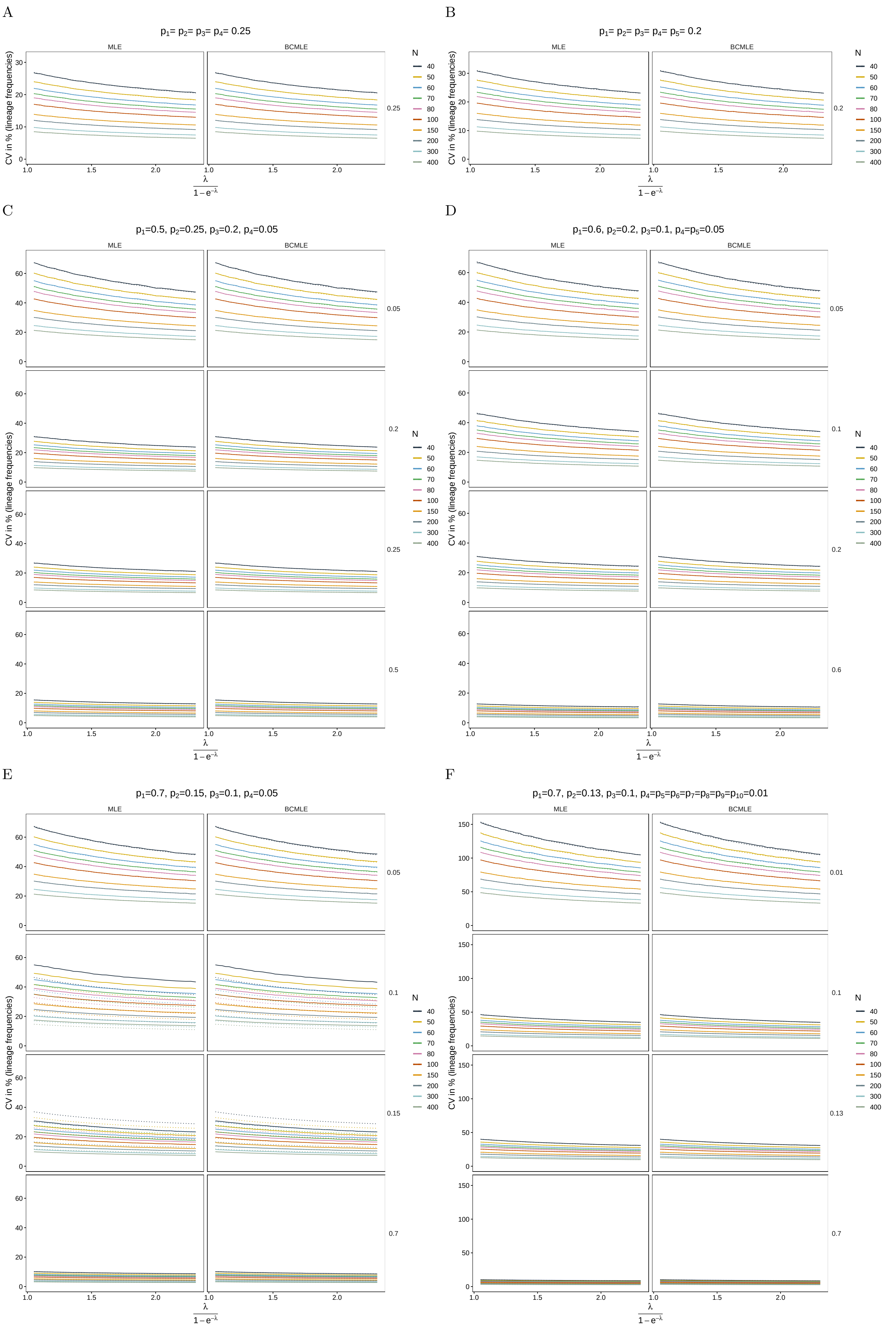

Figure 35: Similar to Figure 32 but for different lineage-frequency distributions.

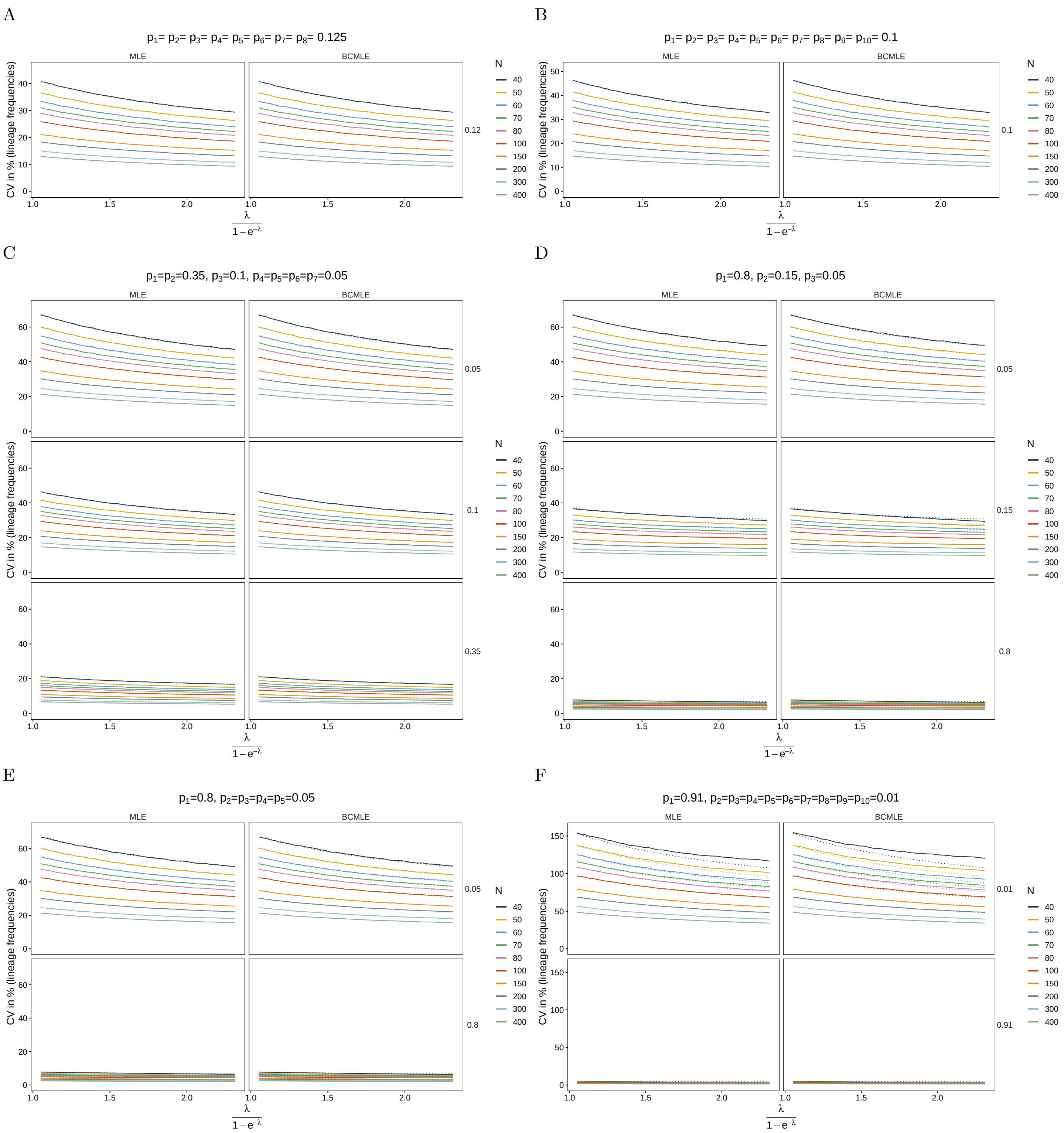

Figure 36: Similar to Figure 32 but for different lineage-frequency distributions.

4.3 Euclidean distance

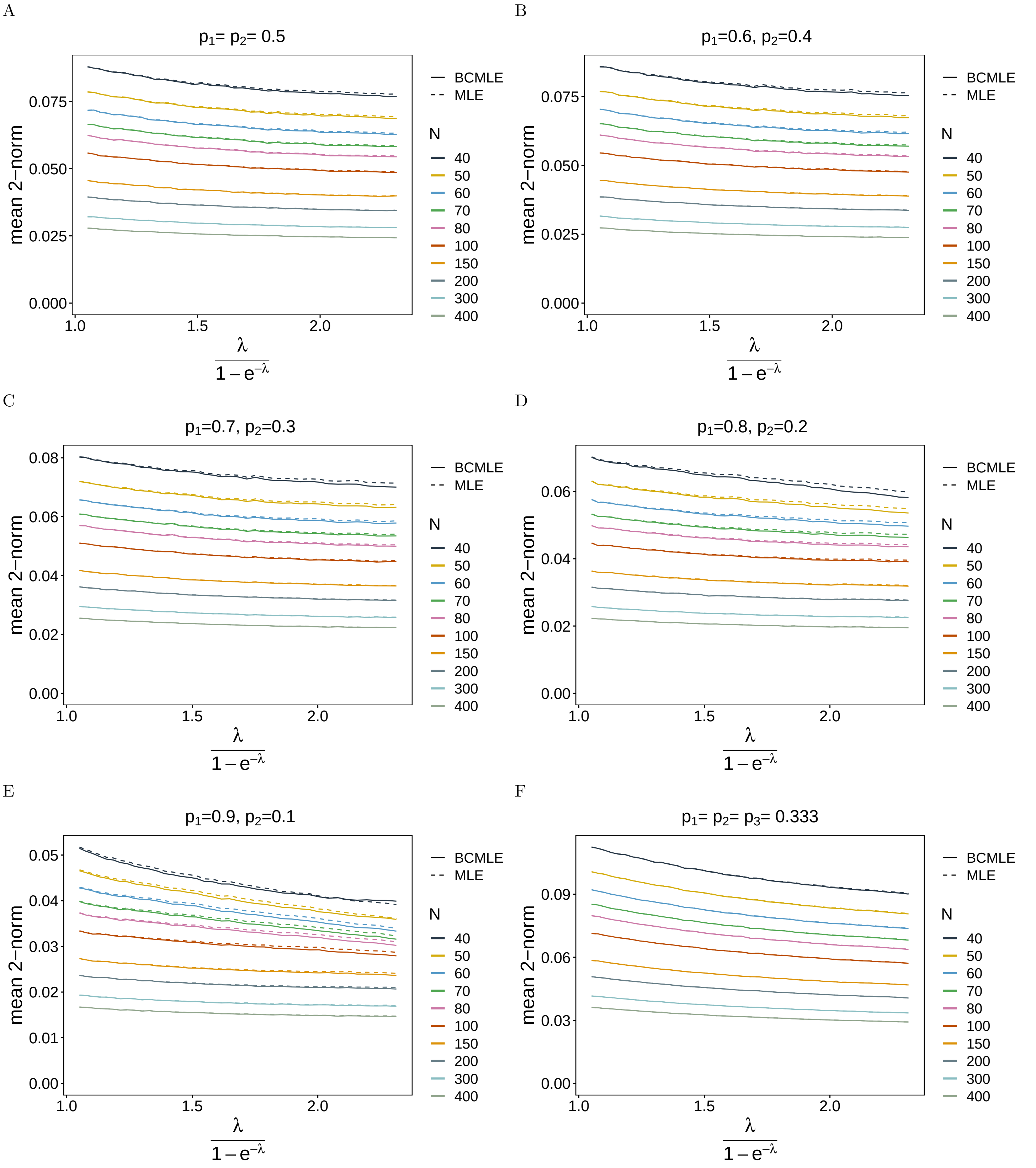

Figure 37: **Euclidean distance**. Shown is the Euclidean distance between the true frequencies and the BCMLEs (solid lines) and the true frequencies and the MLEs (dashed lines) based on simulated data created by the conditional Poisson model. Each panel assumes a different lineage-frequency distribution $\boldsymbol{p}$ shown at the top of each panel. Each colored line corresponds to a different sample size N.

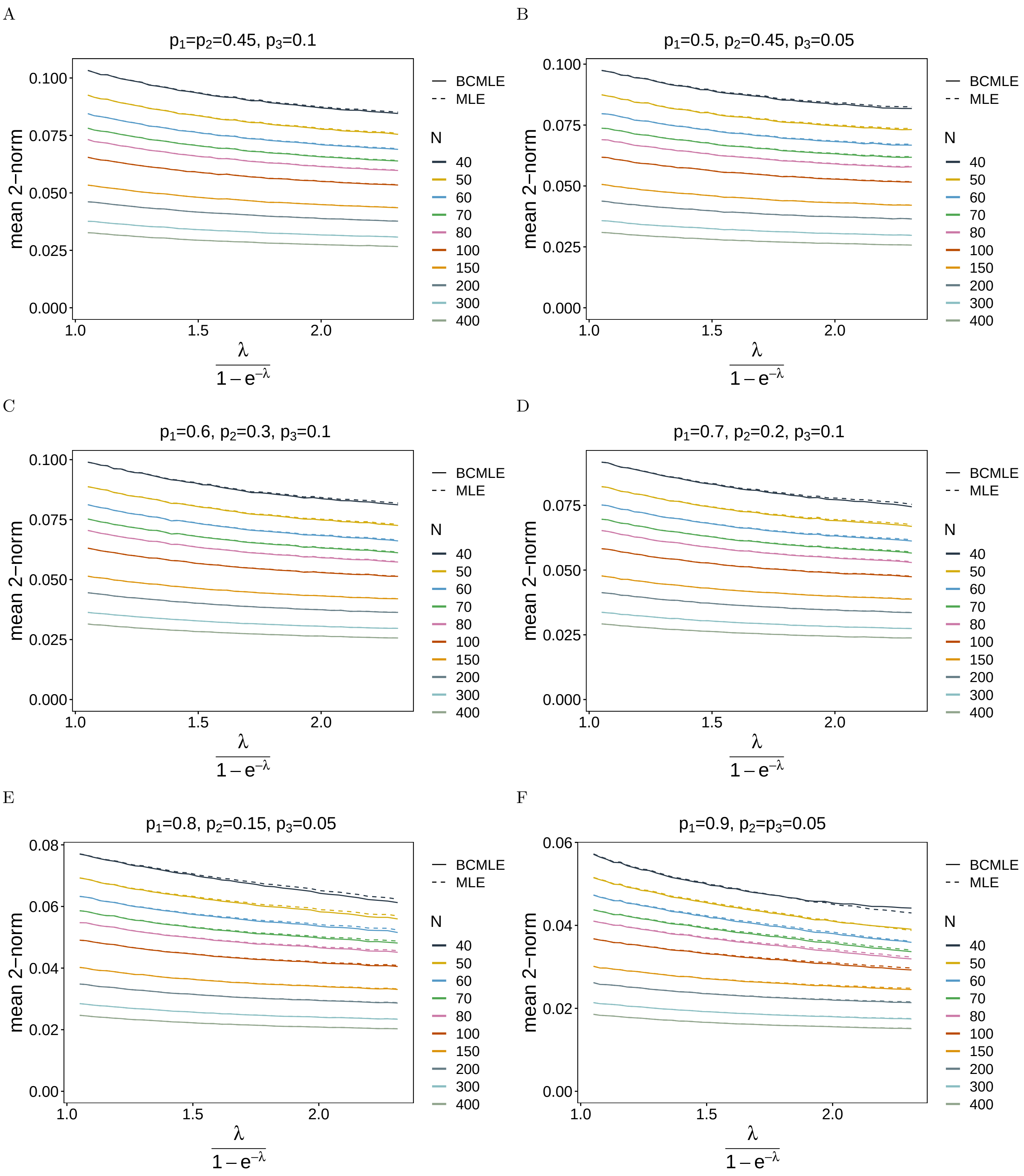

Figure 38: Similar to Figure 37 but for different lineage-frequency distributions.

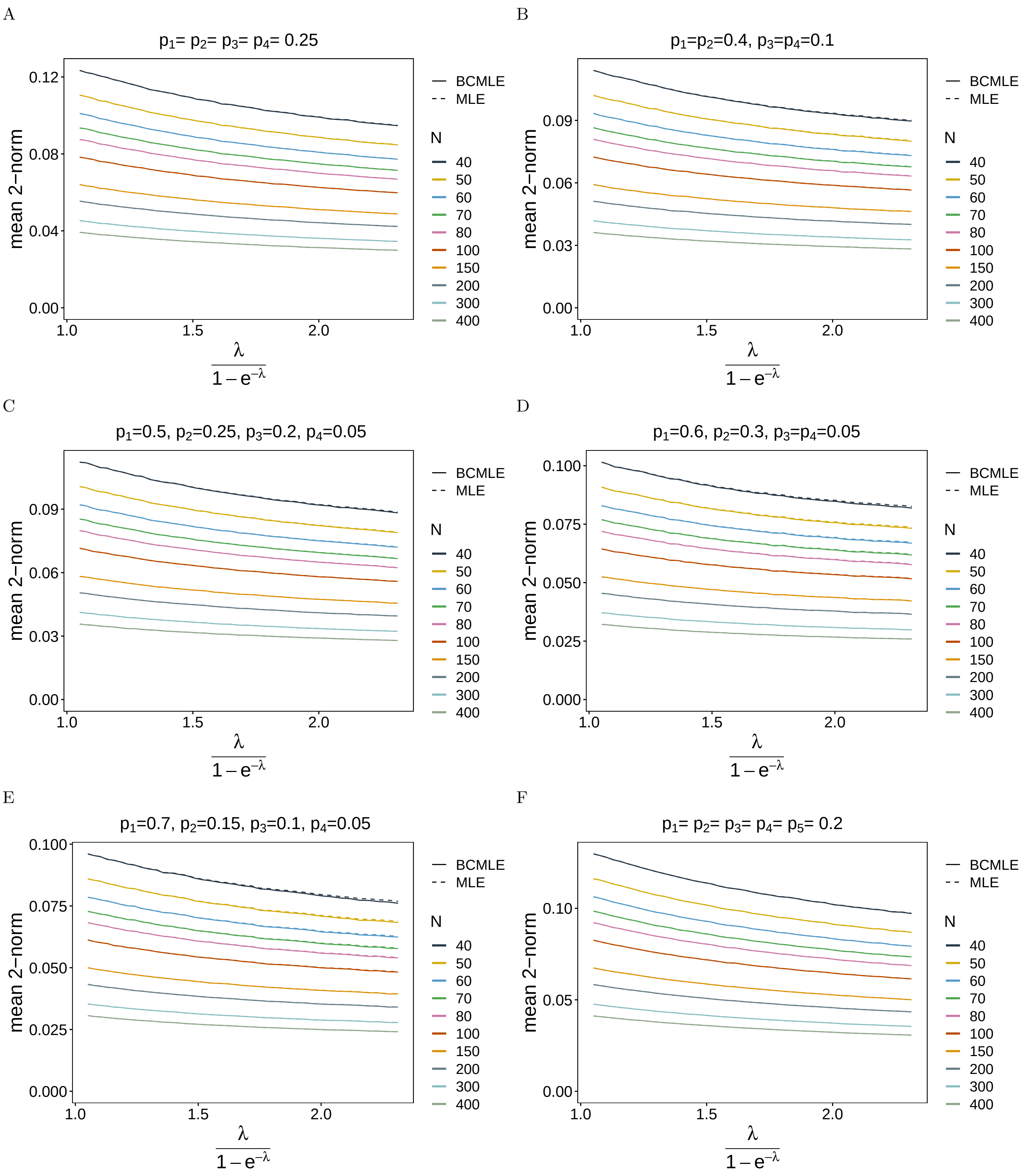

Figure 39: Similar to Figure 37 but for different lineage-frequency distributions.

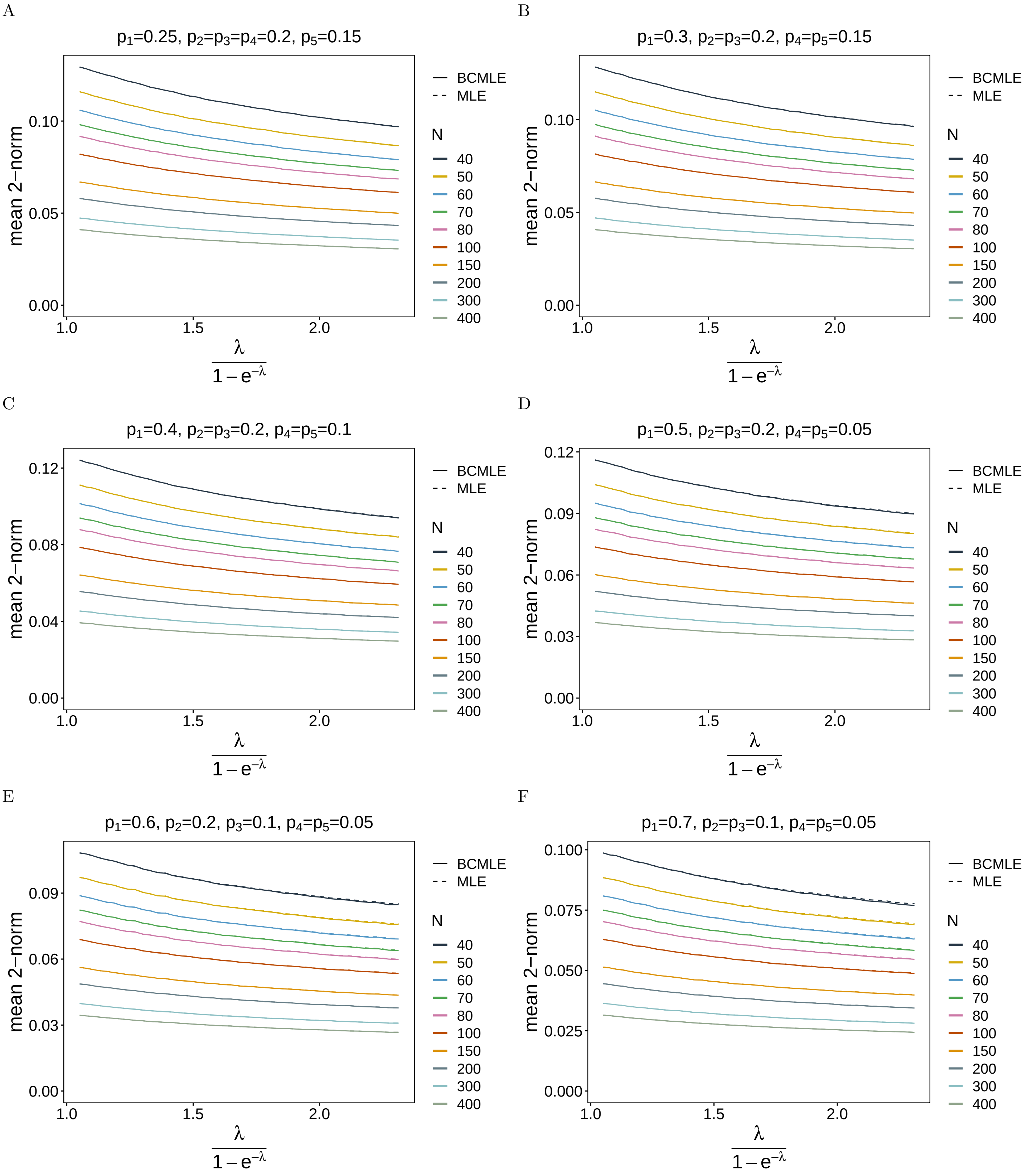

Figure 40: Similar to Figure 37 but for different lineage-frequency distributions.

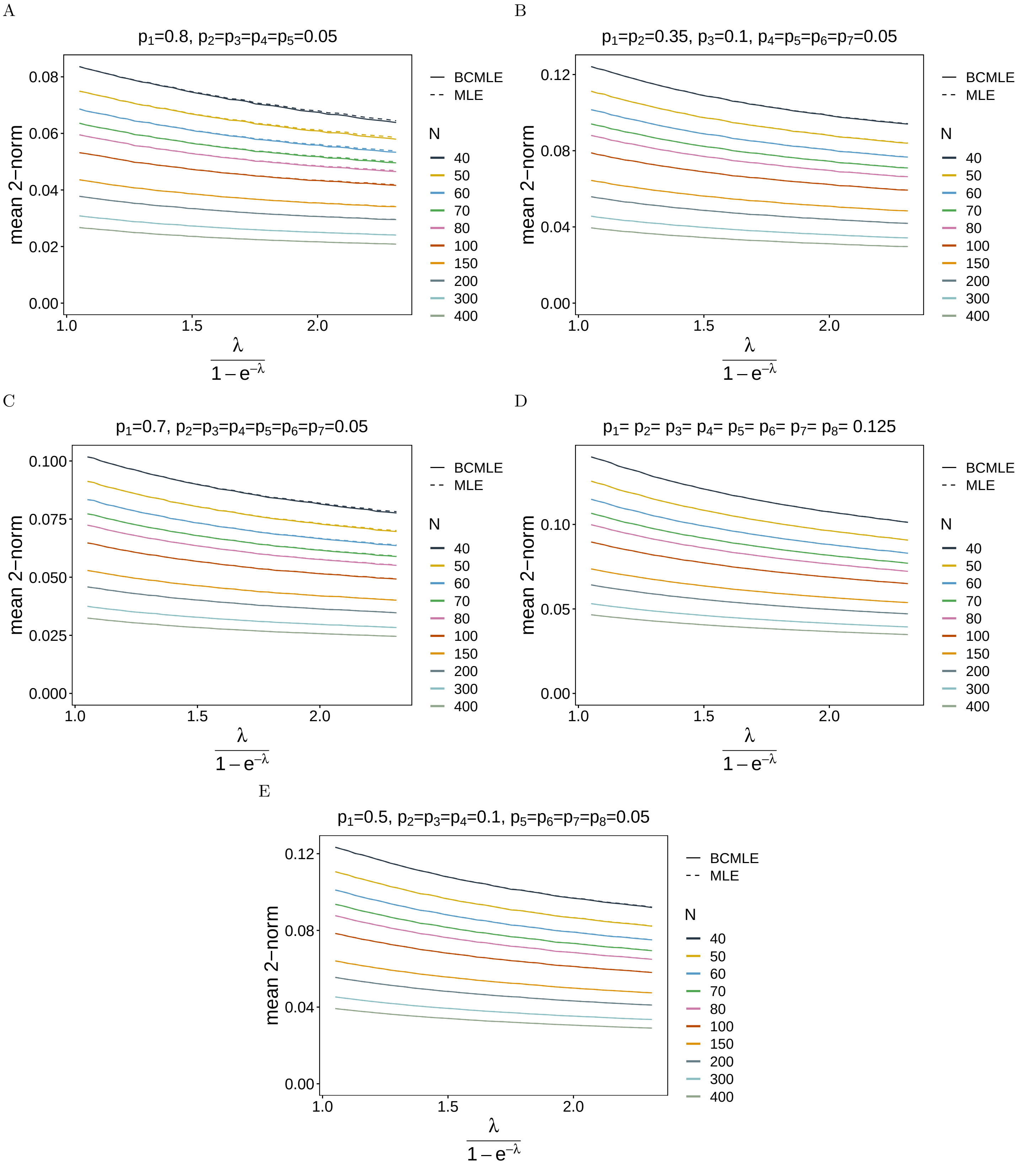

Figure 41: Similar to Figure 37 but for different lineage-frequency distributions.

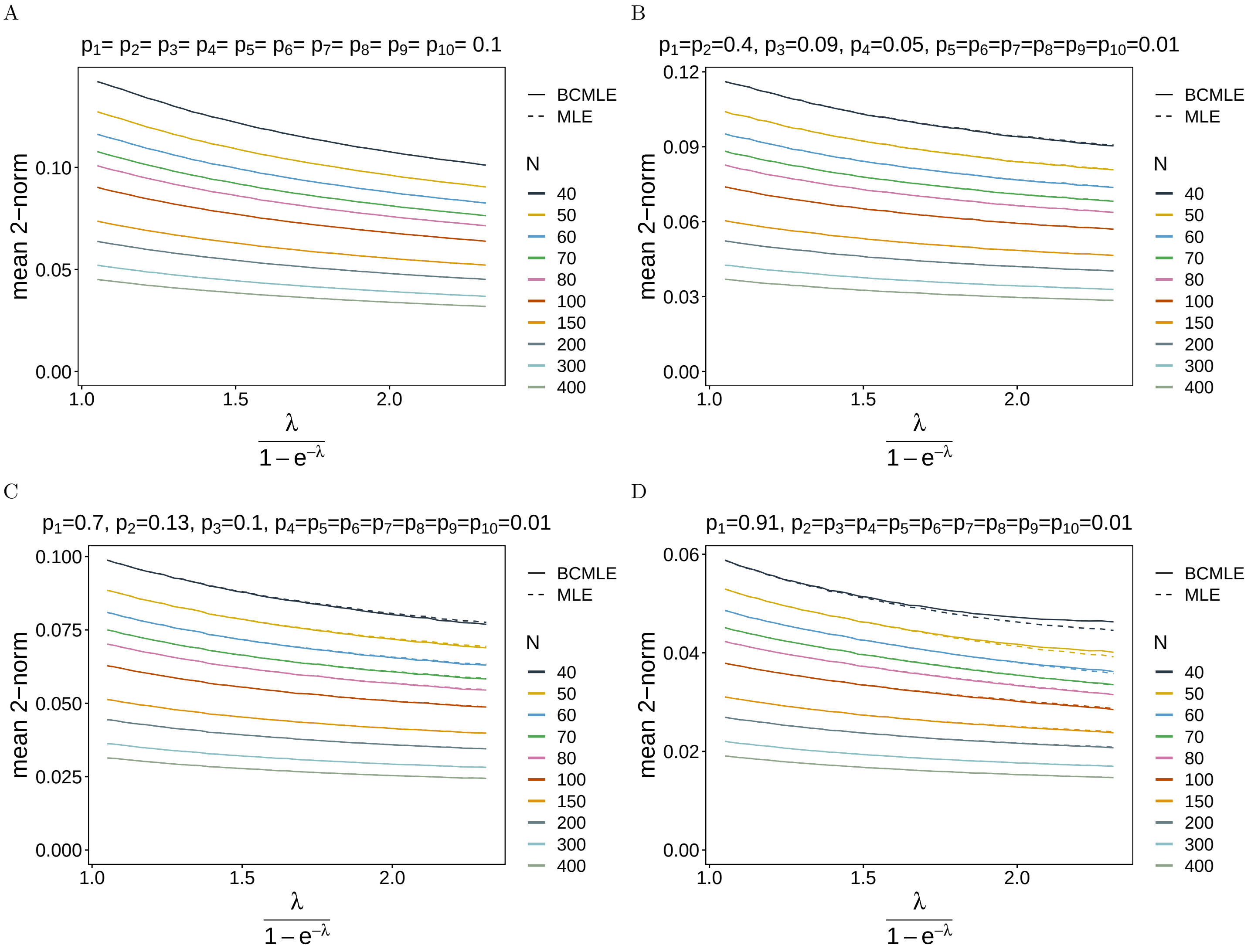

Figure 42: Similar to Figure 37 but for different lineage-frequency distributions

4.4 Kullback–Leibler divergence

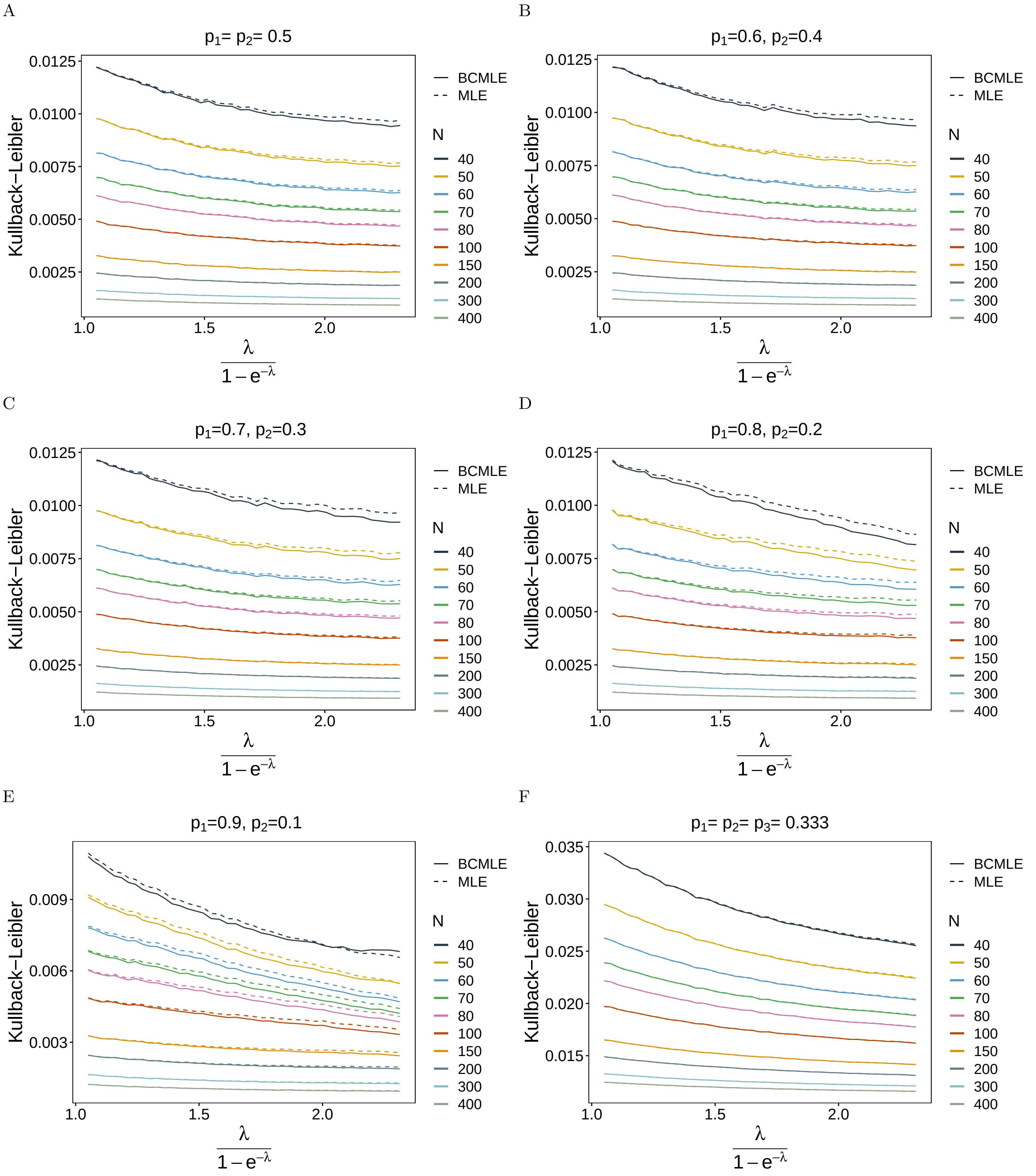

Figure 43: **Kullback-Leibler divergence - lineage frequencies**. Similar to Figure 37 but for the Kullback-Leiber divergence.

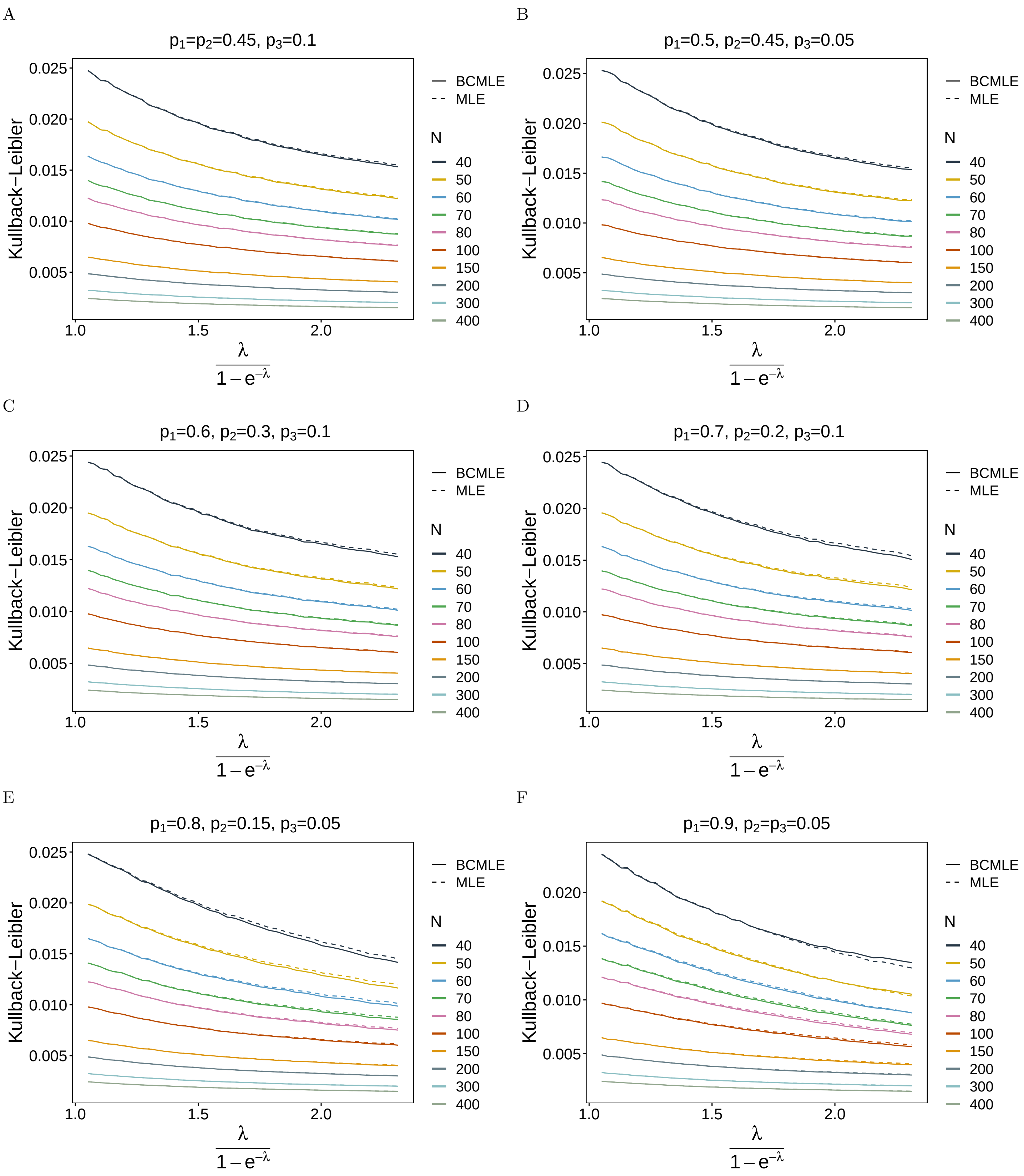

Figure 44: Similar to Figure 43 but for different lineage-frequency distributions.

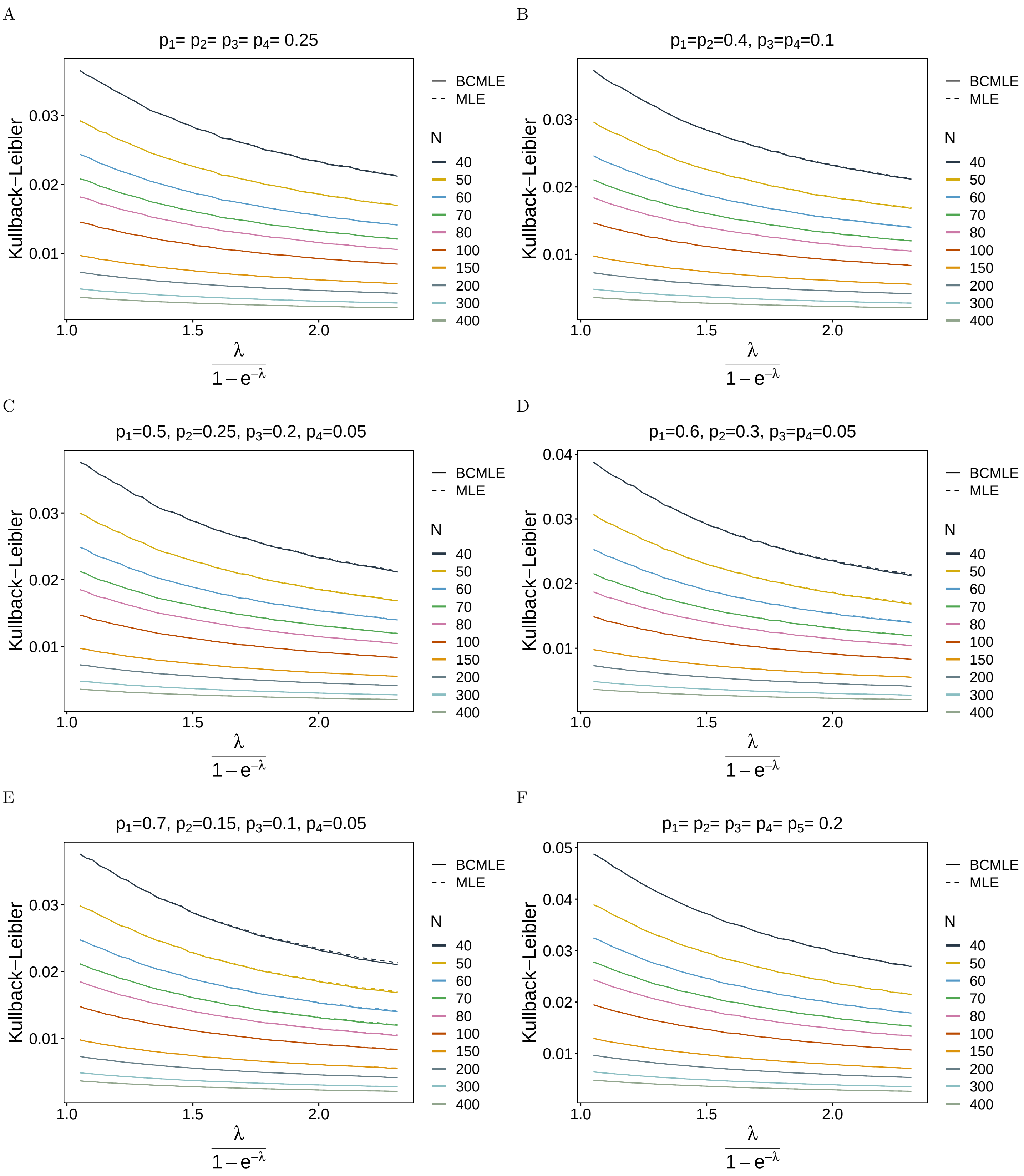

Figure 45: Similar to Figure 43 but for different lineage-frequency distributions.

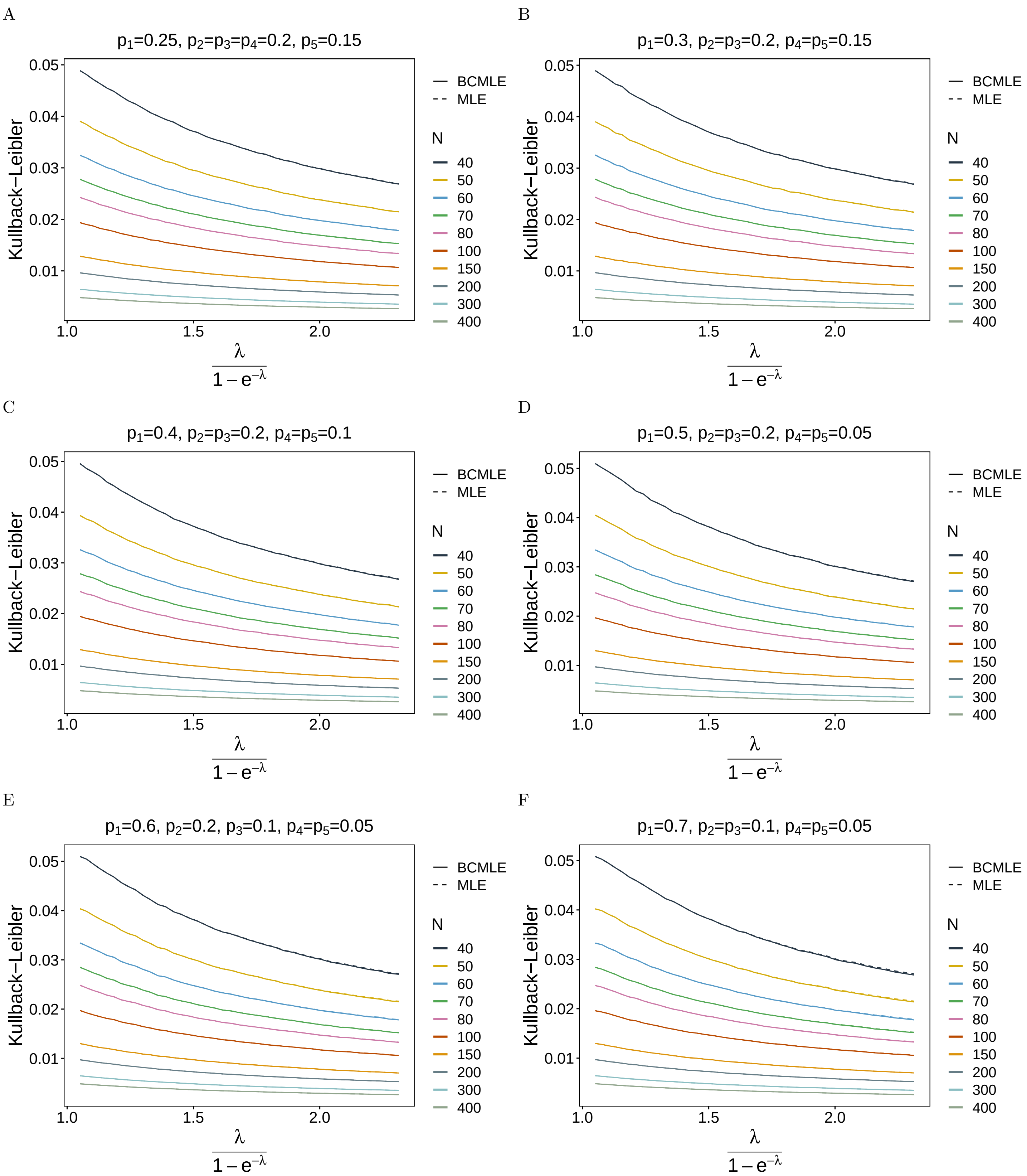

Figure 46: Similar to Figure 43 but for different lineage-frequency distributions.

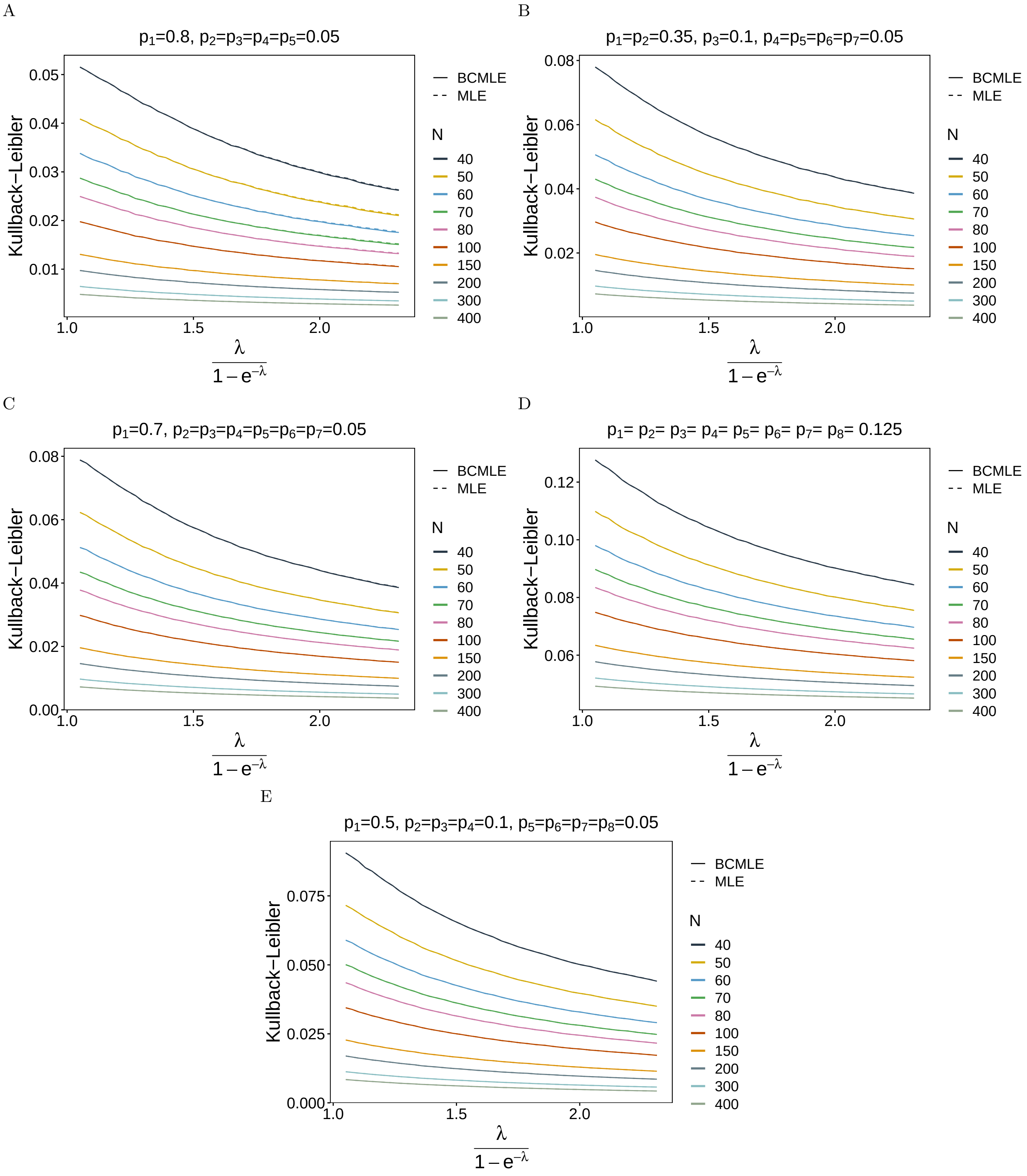

Figure 47: Similar to Figure 43 but for different lineage-frequency distributions.

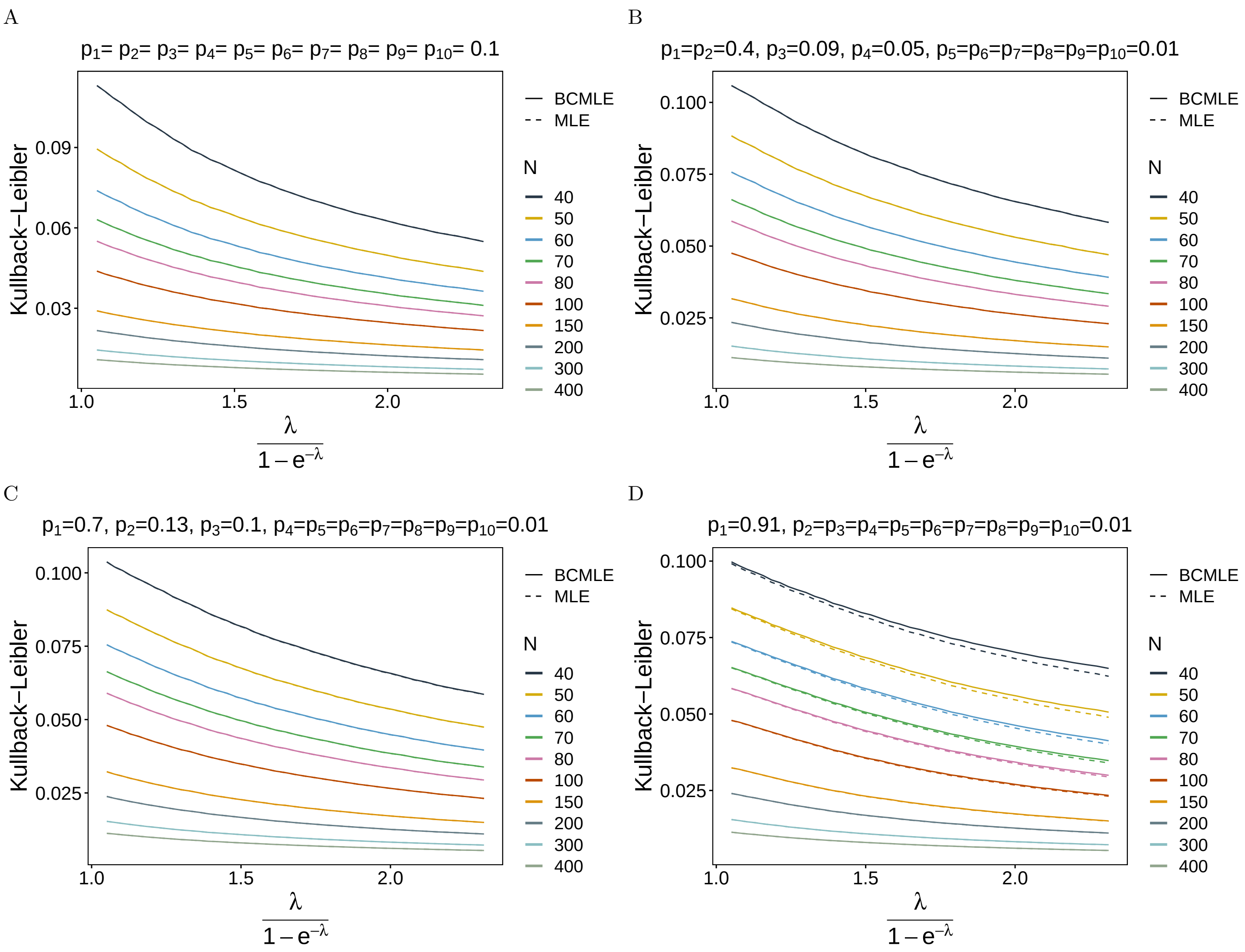

Figure 48: Similar to Figure 43 but for different lineage-frequency distributions.

5 Model Violations

5.1 Relative bias of the BCMLE in %

5.1.1 Different levels of overdispersion

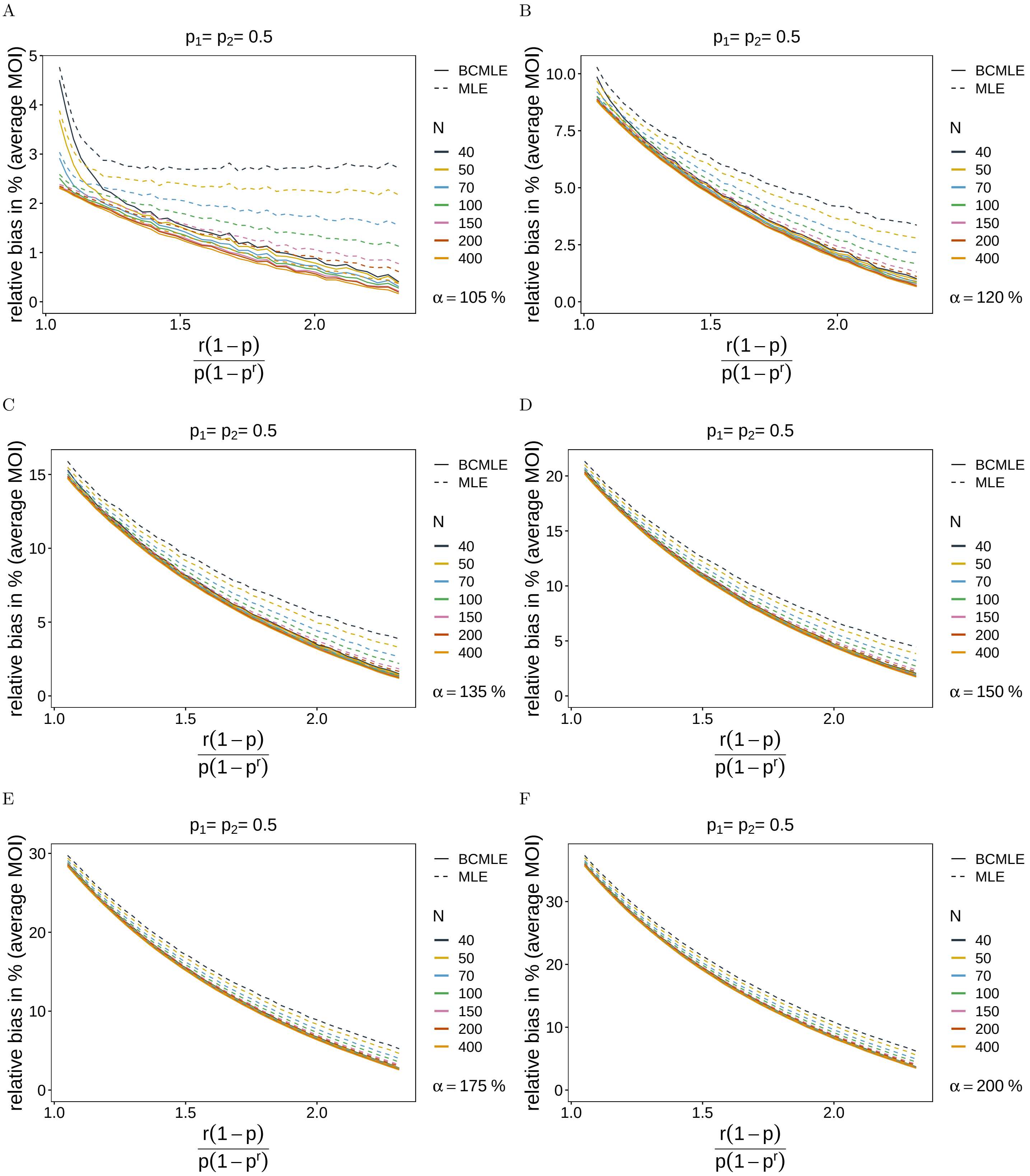

Figure 49: **Robustness of MOI estimates against model violations**. The figure shows the bias of the BCMLE $\hat{\psi}^{(\mathrm{bc})}$ (solid lines) and MLE $\hat{\psi}$ (dashed lines) in % as a function of the true parameter $\psi = \frac{r(1-p)}{p(1-p^r)}$. The datasets are generated from the conditional negative binomial model whereas the estimates are derived from the conditional Poisson model. The panels in different rows correspond to different levels of oversispersion indicated by α. Panels on the left and right assume a different lineage-frequency distributions $\boldsymbol{p}$ shown at the top of each panel. Line colors correspond to a different sample sizes (N).

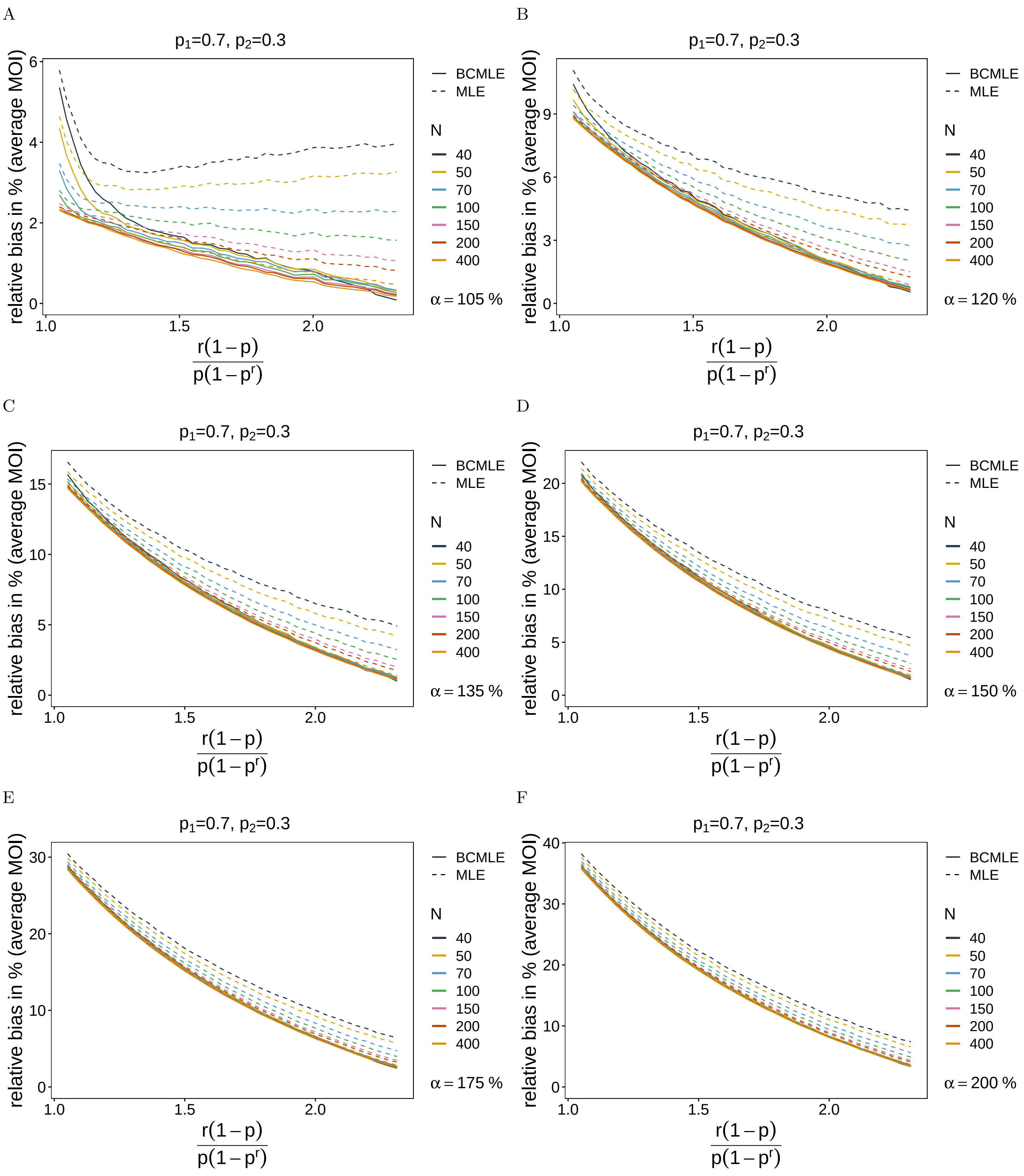

Figure 50: Similar to Figure 49 but for different lineage-frequency distributions.

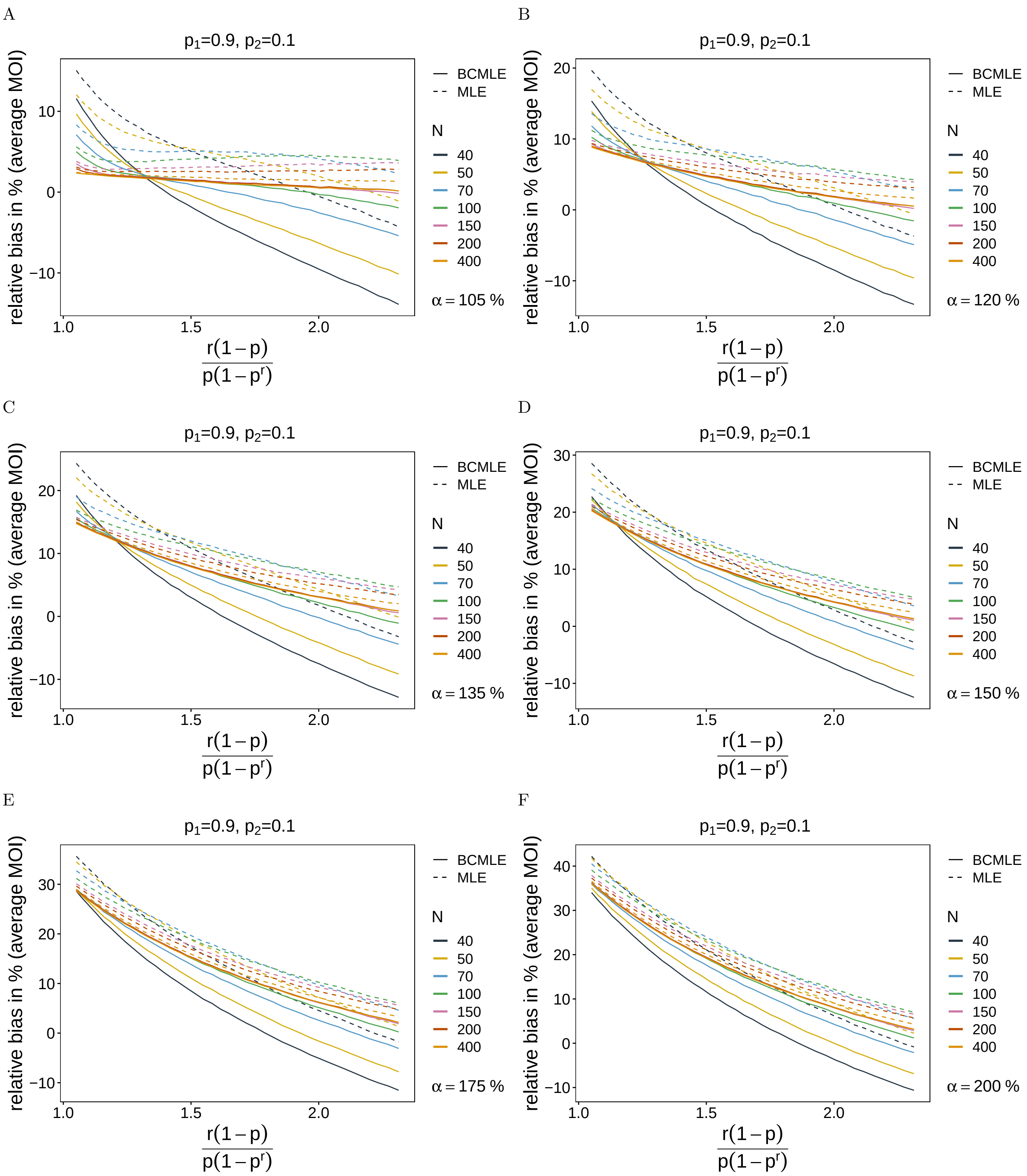

Figure 51: Similar to Figure 49 but for different lineage-frequency distributions.

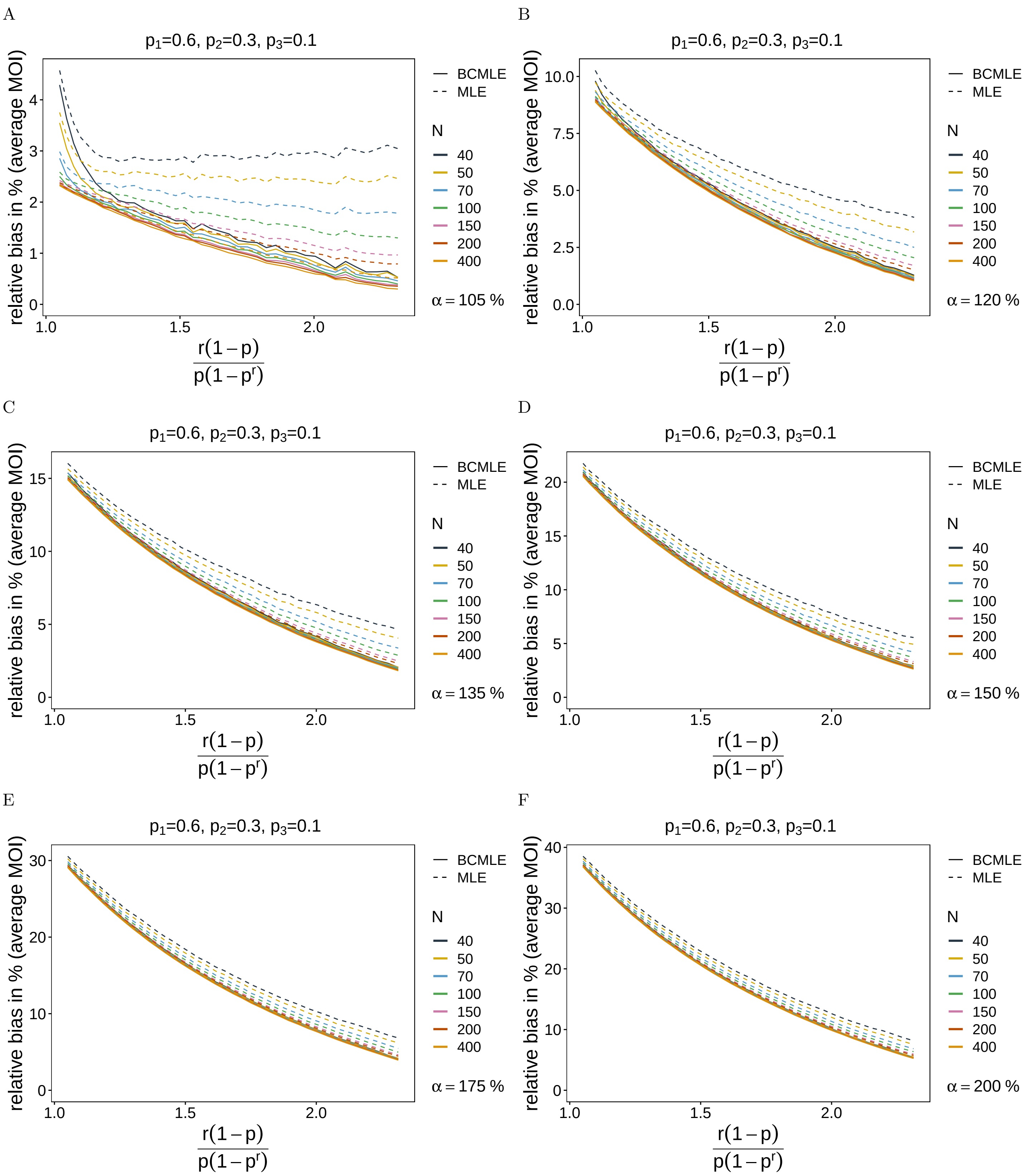

Figure 52: Similar to Figure 49 but for different lineage-frequency distributions.

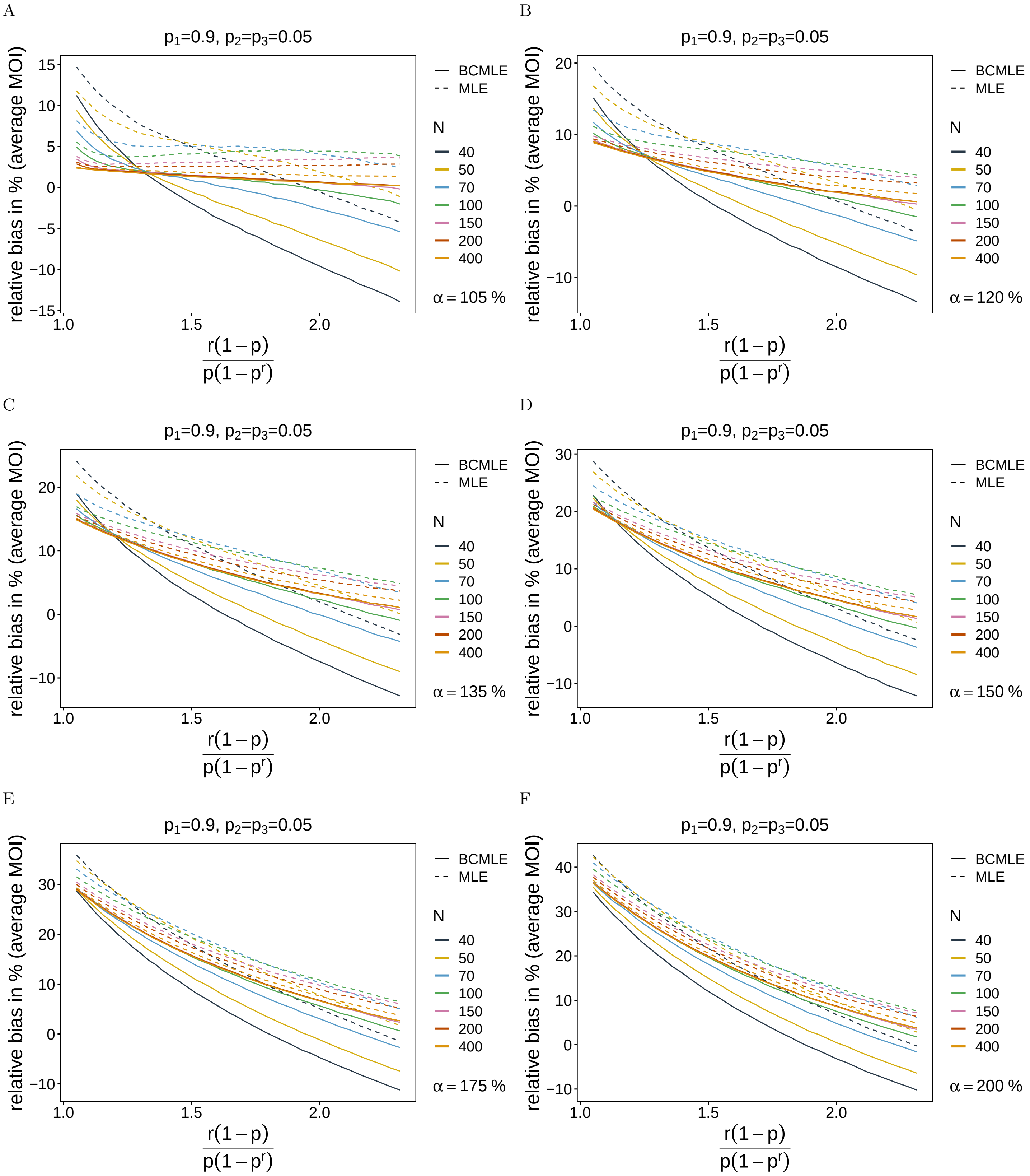

Figure 53: Similar to Figure 49 but for different lineage-frequency distributions.

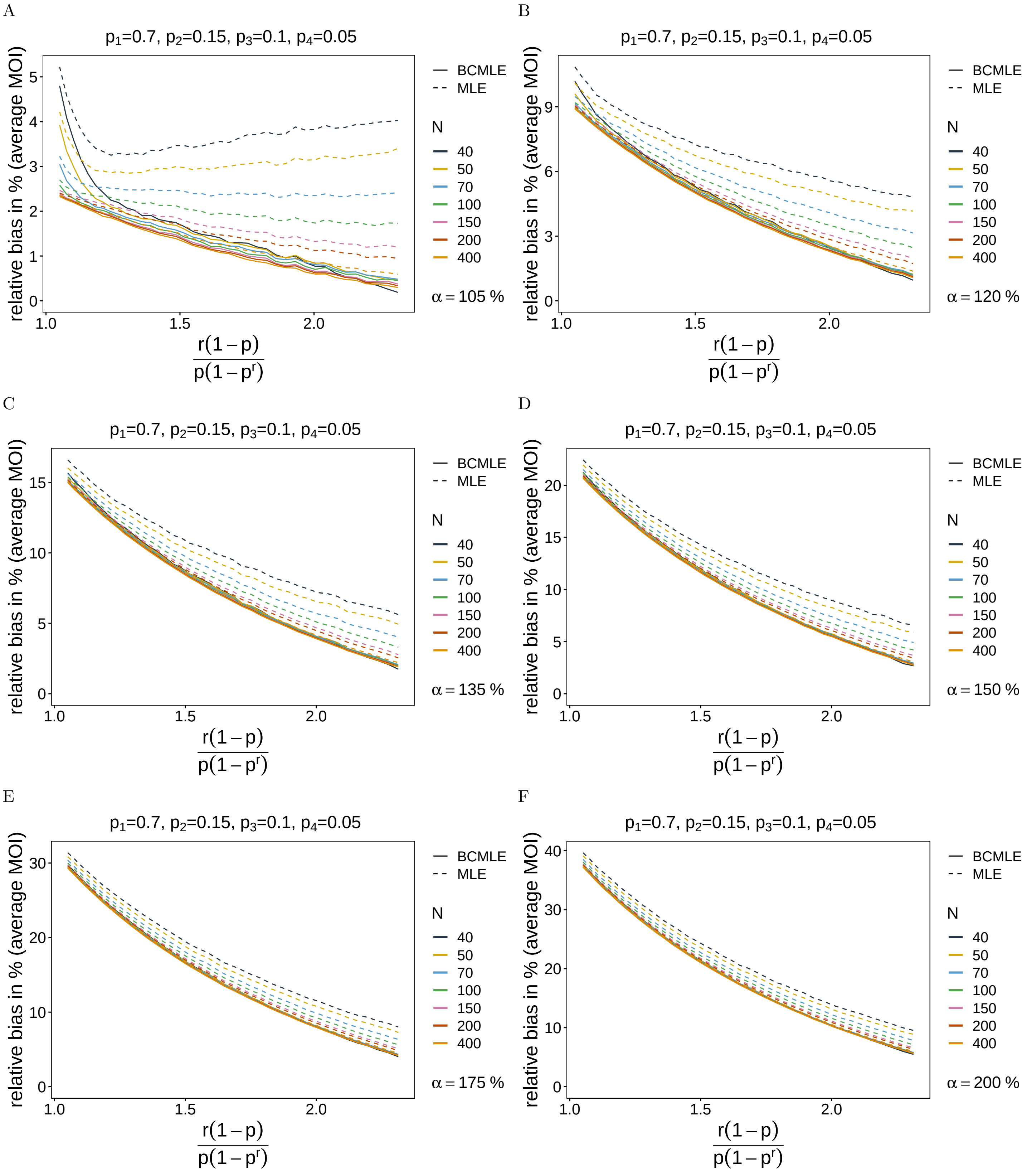

Figure 54: Similar to Figure 49 but for different lineage-frequency distributions.

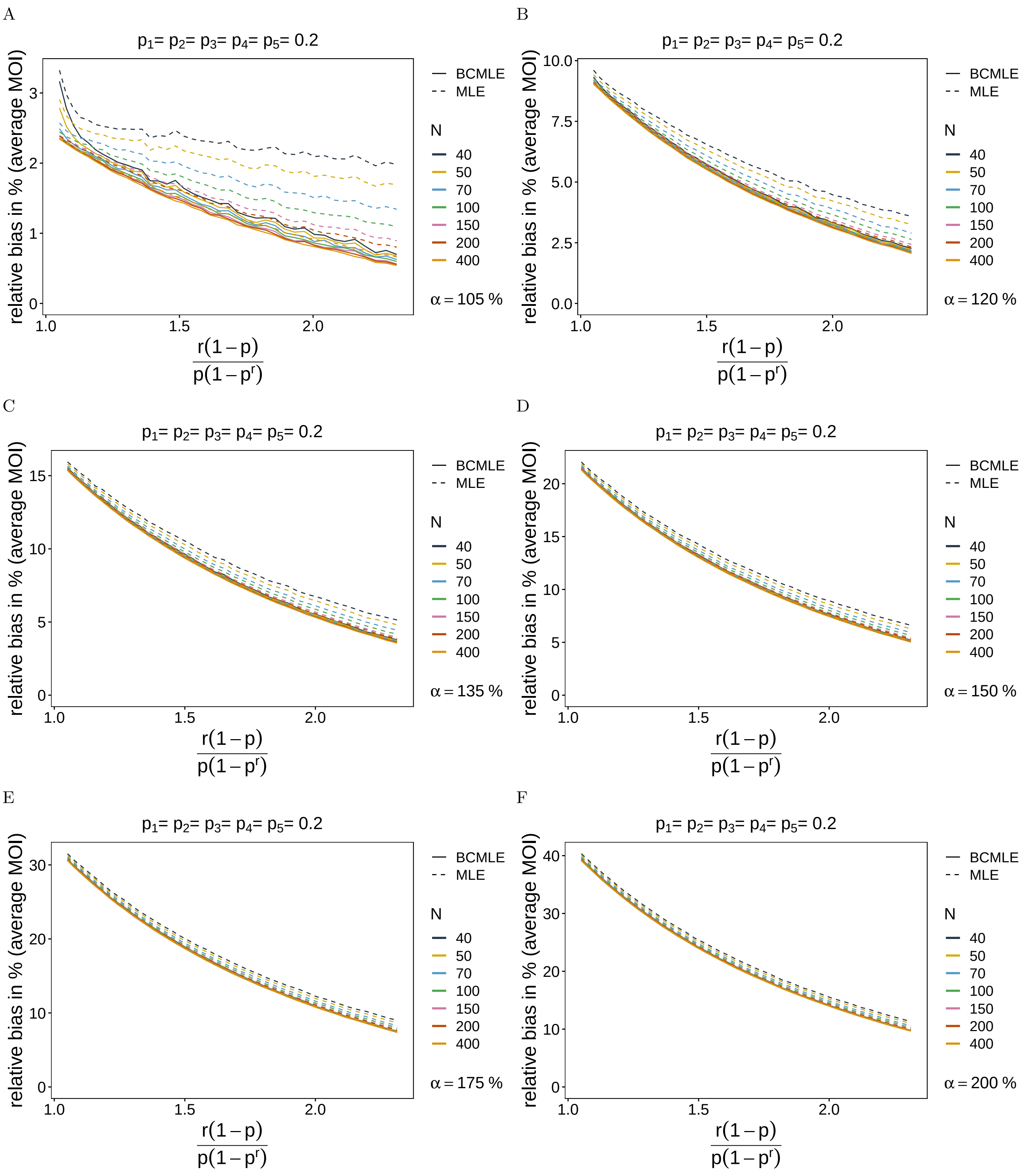

Figure 55: Similar to Figure 49 but for different lineage-frequency distributions.

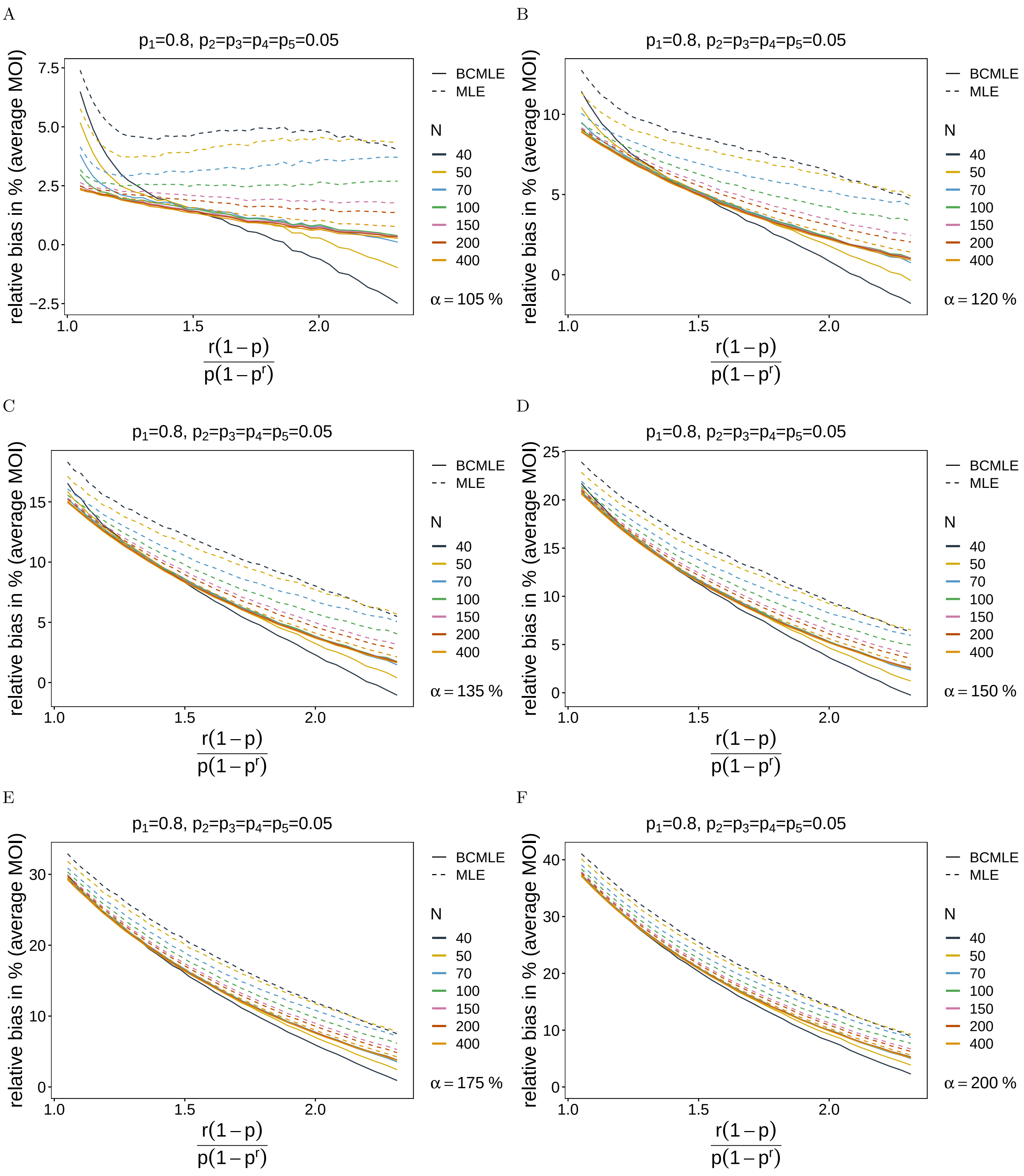

Figure 56: Similar to Figure 49 but for different lineage-frequency distributions.

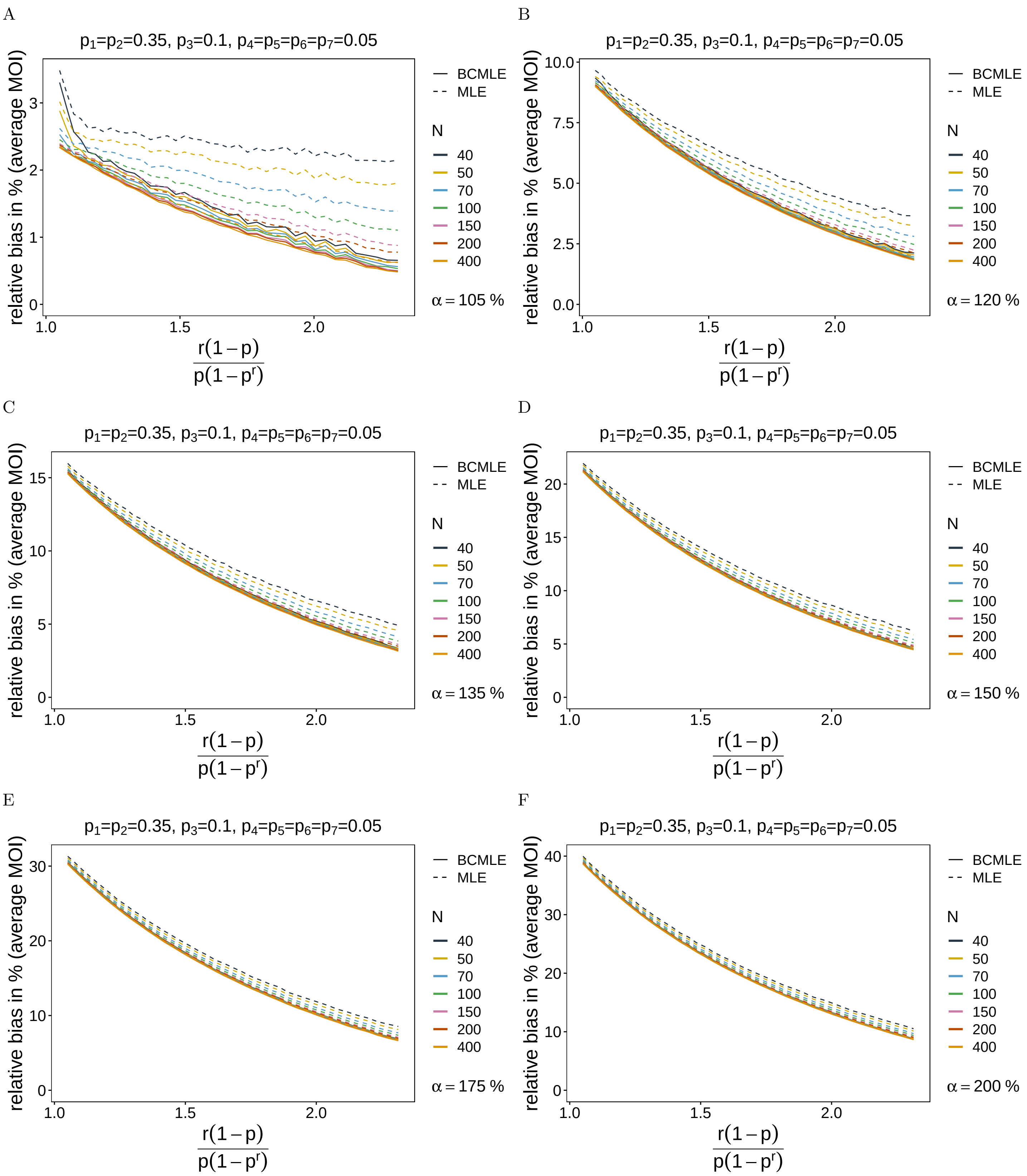

Figure 57: Similar to Figure 49 but for different lineage-frequency distributions.

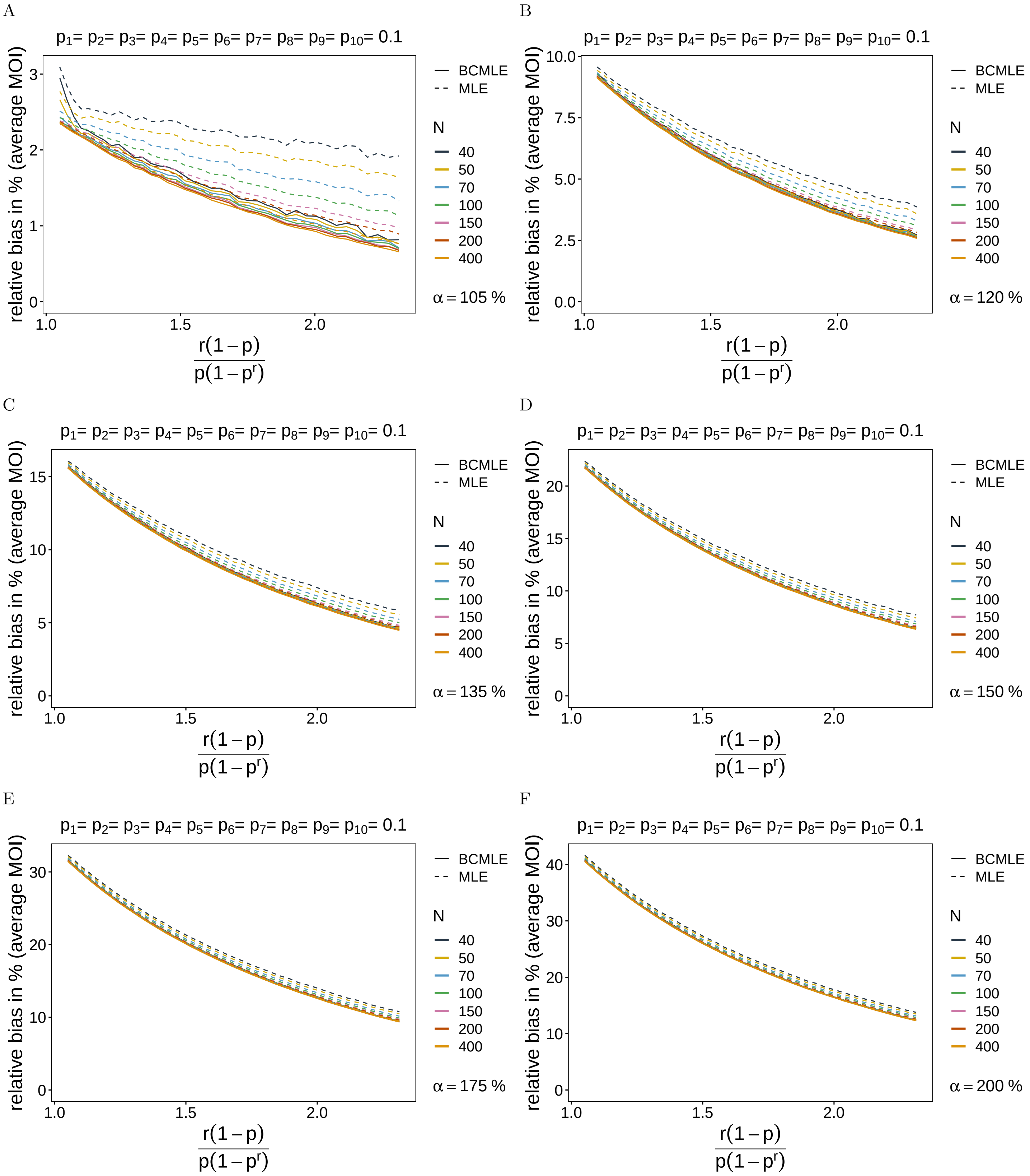

Figure 58: Similar to Figure 49 but for different lineage-frequency distributions.

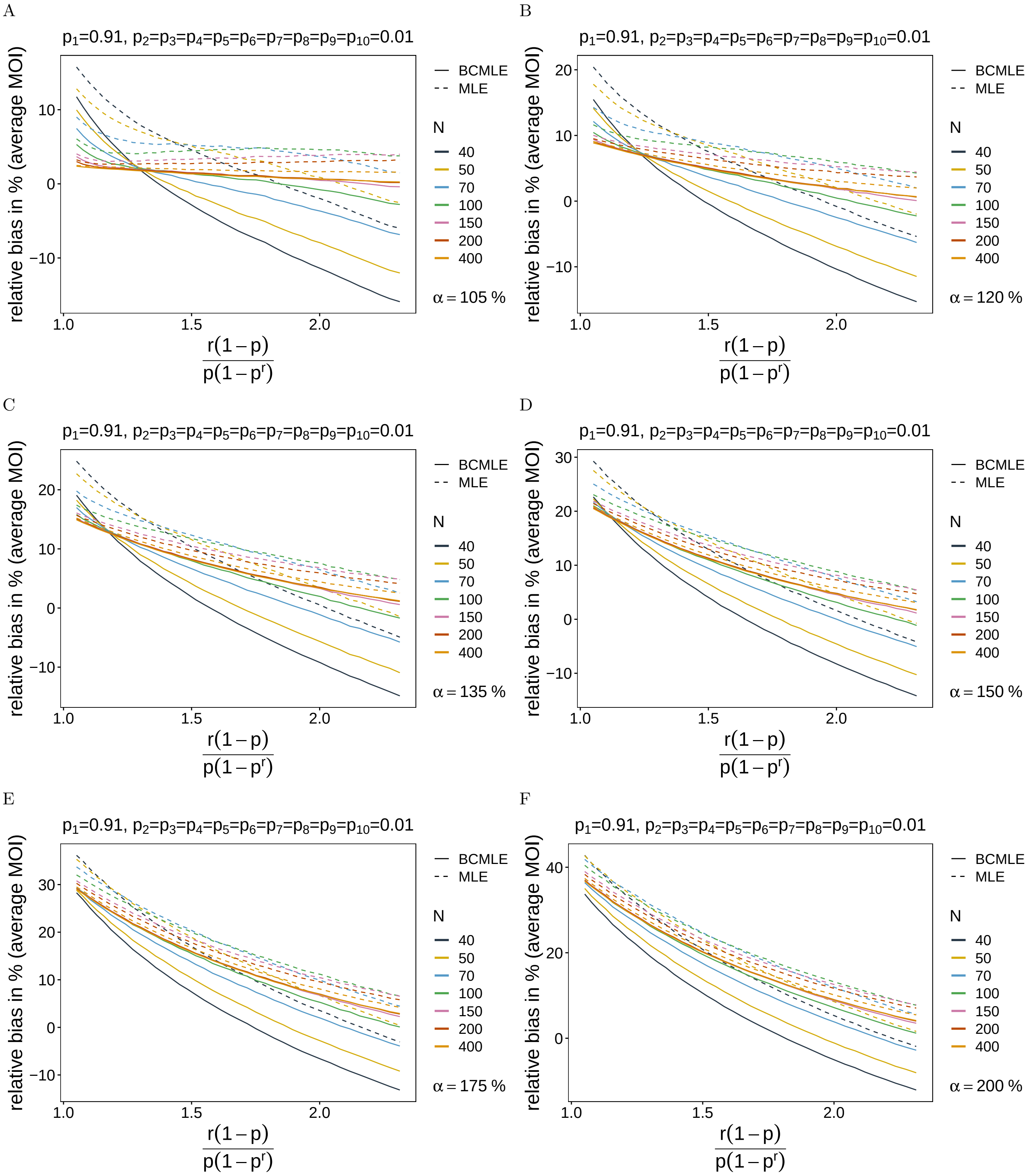

Figure 59: Similar to Figure 49 but for different lineage-frequency distributions.

5.1.2 Different sample sizes

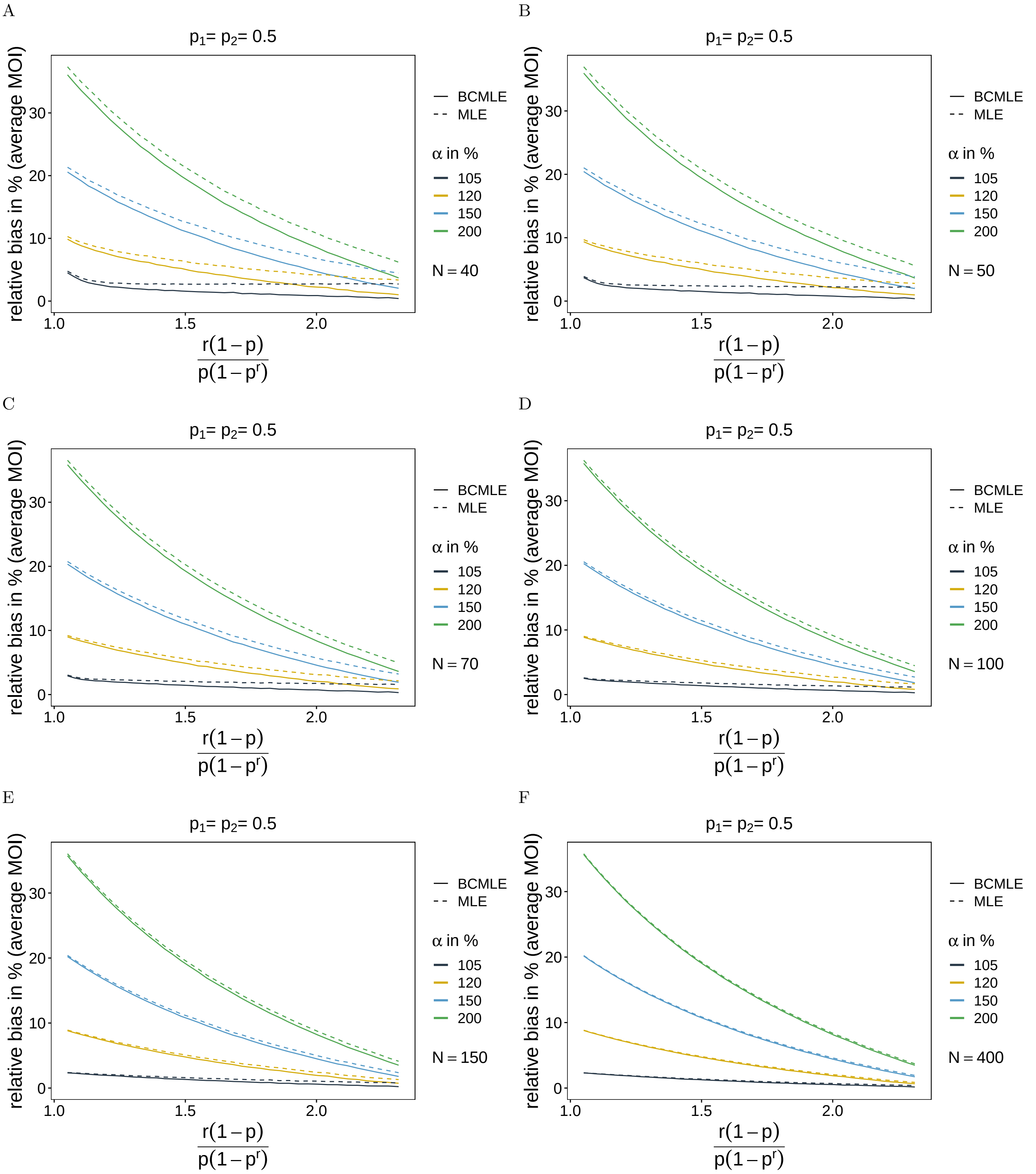

Figure 60: **Robustness of MOI estimates against model violations.**. The figure shows the bias in % of the BCMLE $\hat{\psi}^{(\text{bc})}$ (solid lines) and the MLE $\hat{\psi}$ (dashed lines) as a function of the true parameter $\psi = \frac{r(1-p)}{p(1-p^r)}$. The datasets are generated from the conditional negative binomial model whereas the estimates are derived from the conditional Poisson model. The panels correspond to different sample sizes N. All panels assume the same lineage-frequency distribution $\boldsymbol{p}$ shown at the top of each panel. Each colored line corresponds to a different over-dispersion level α specified in %.

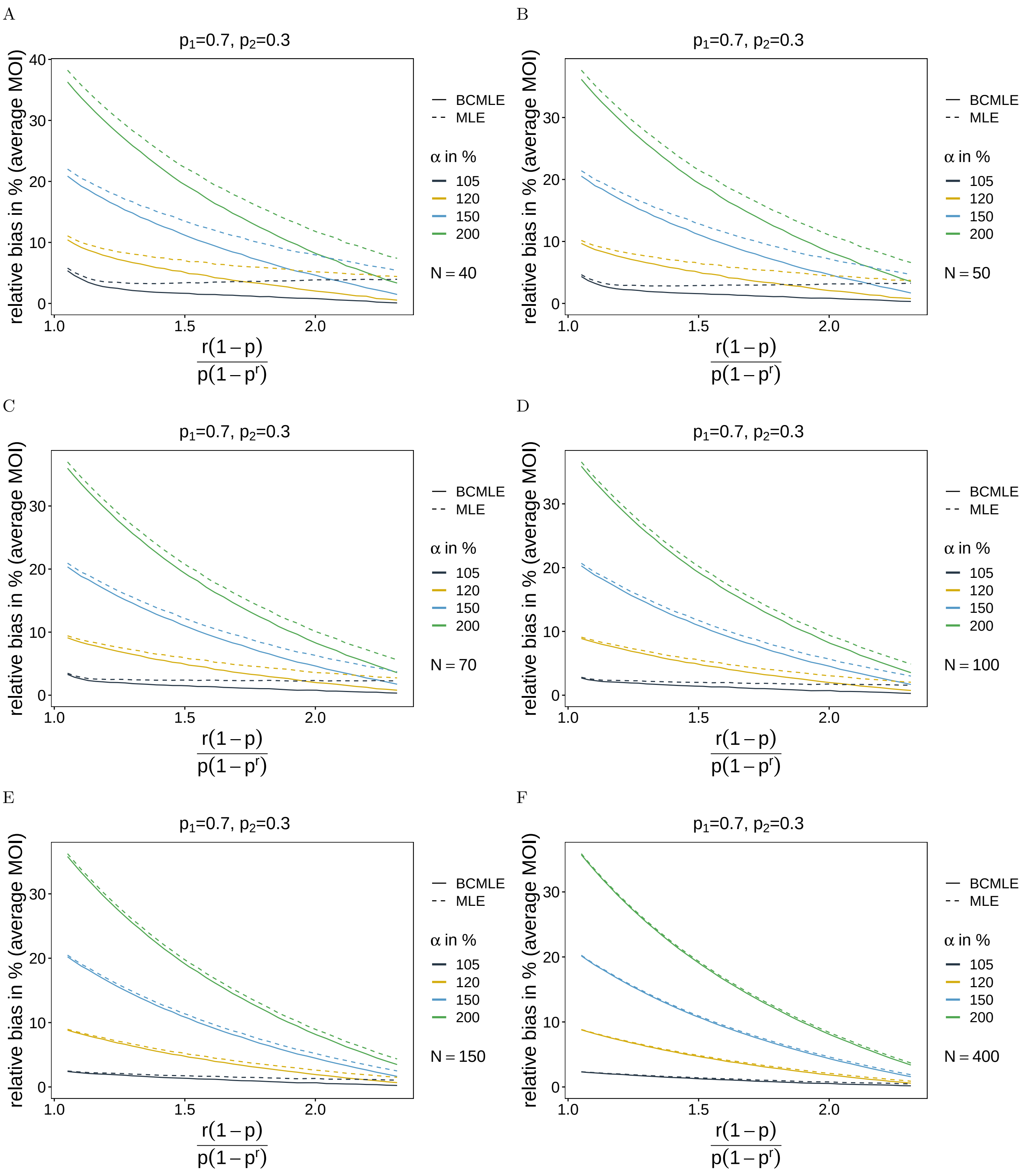

Figure 61: Similar to Figure 60 but for different lineage-frequency distributions.

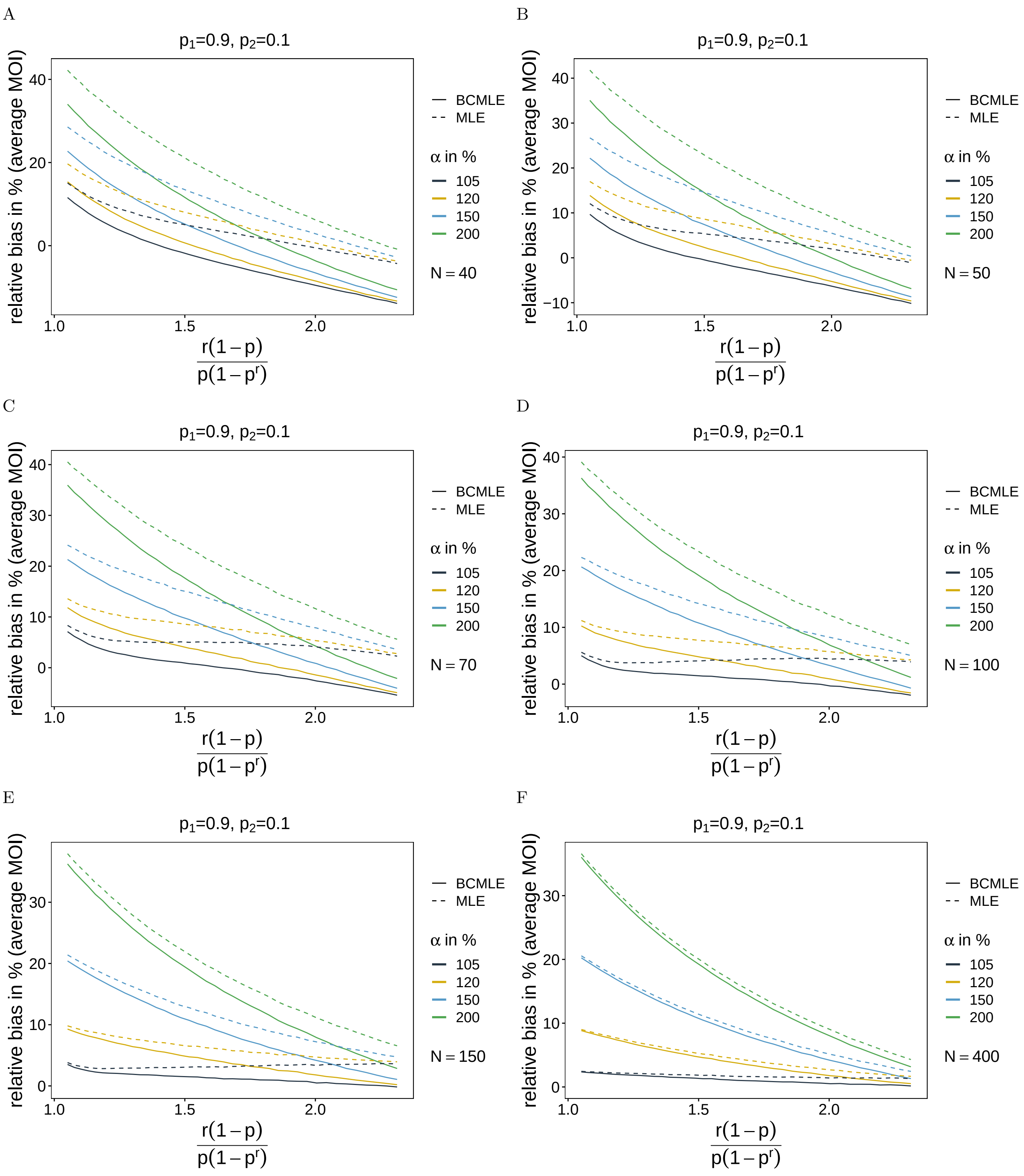

Figure 62: Similar to Figure 60 but for different lineage-frequency distributions.

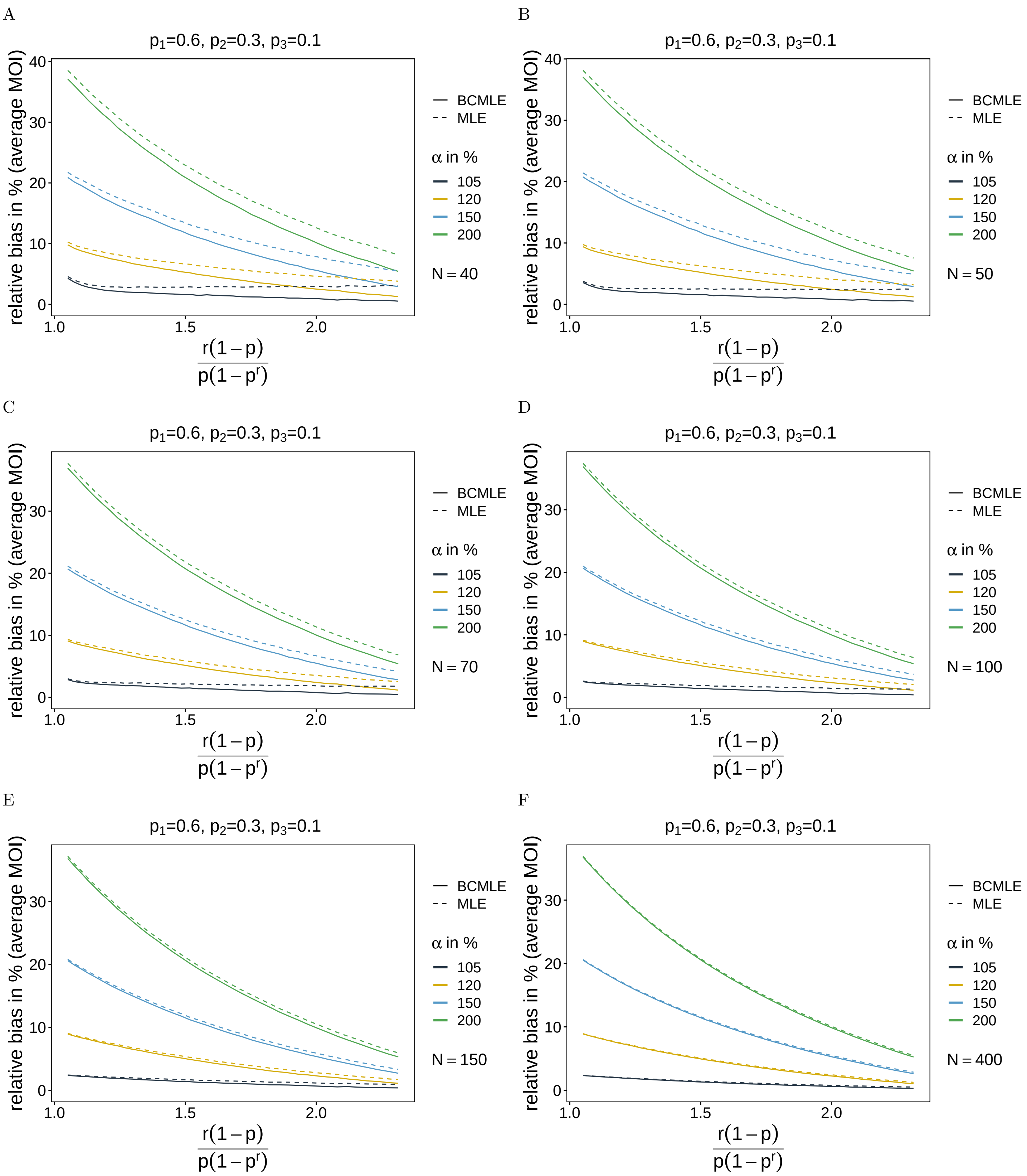

Figure 63: Similar to Figure 60 but for different lineage-frequency distributions.

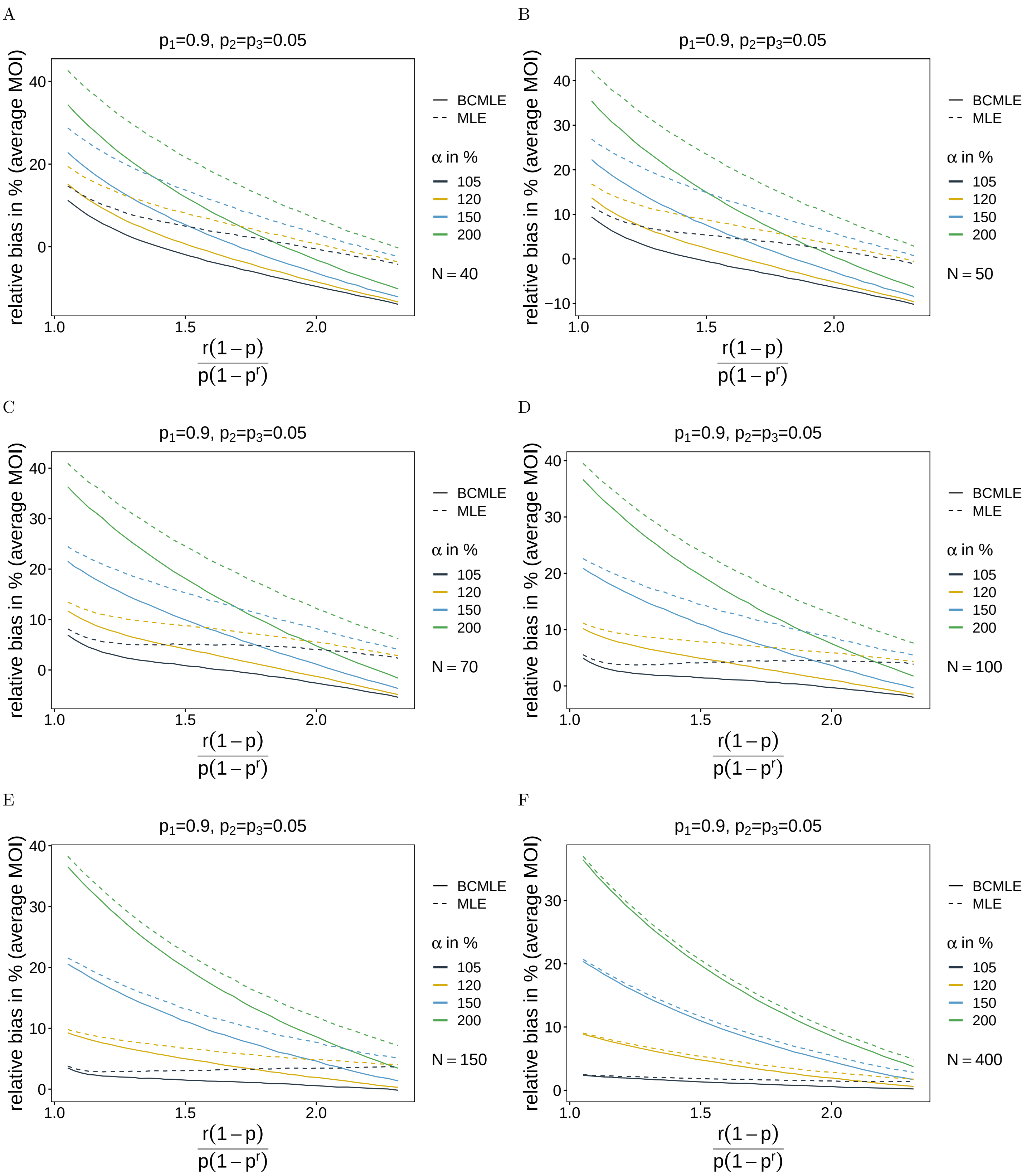

Figure 64: Similar to Figure 60 but for different lineage-frequency distributions.

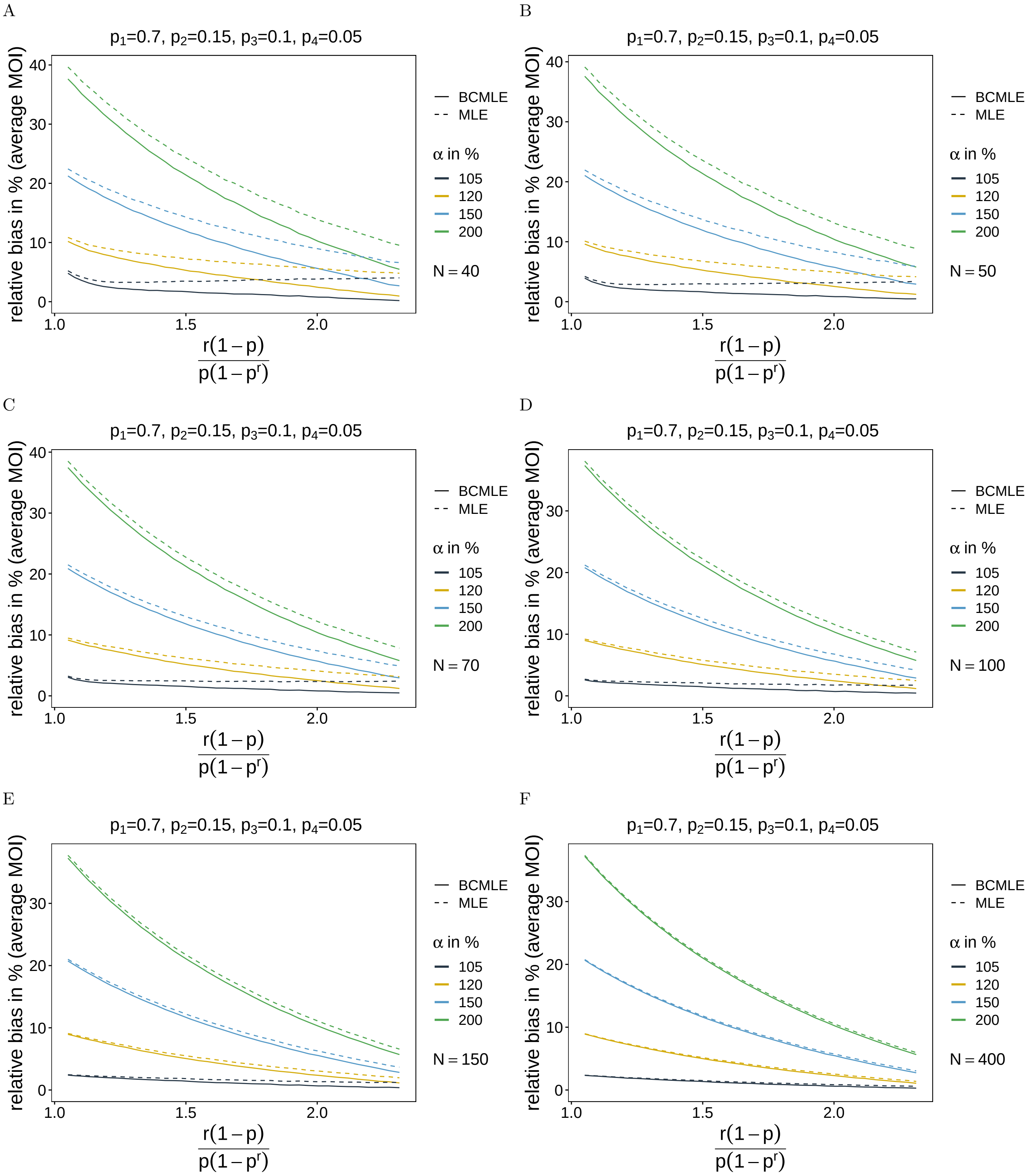

Figure 65: Similar to Figure 60 but for different lineage-frequency distributions.

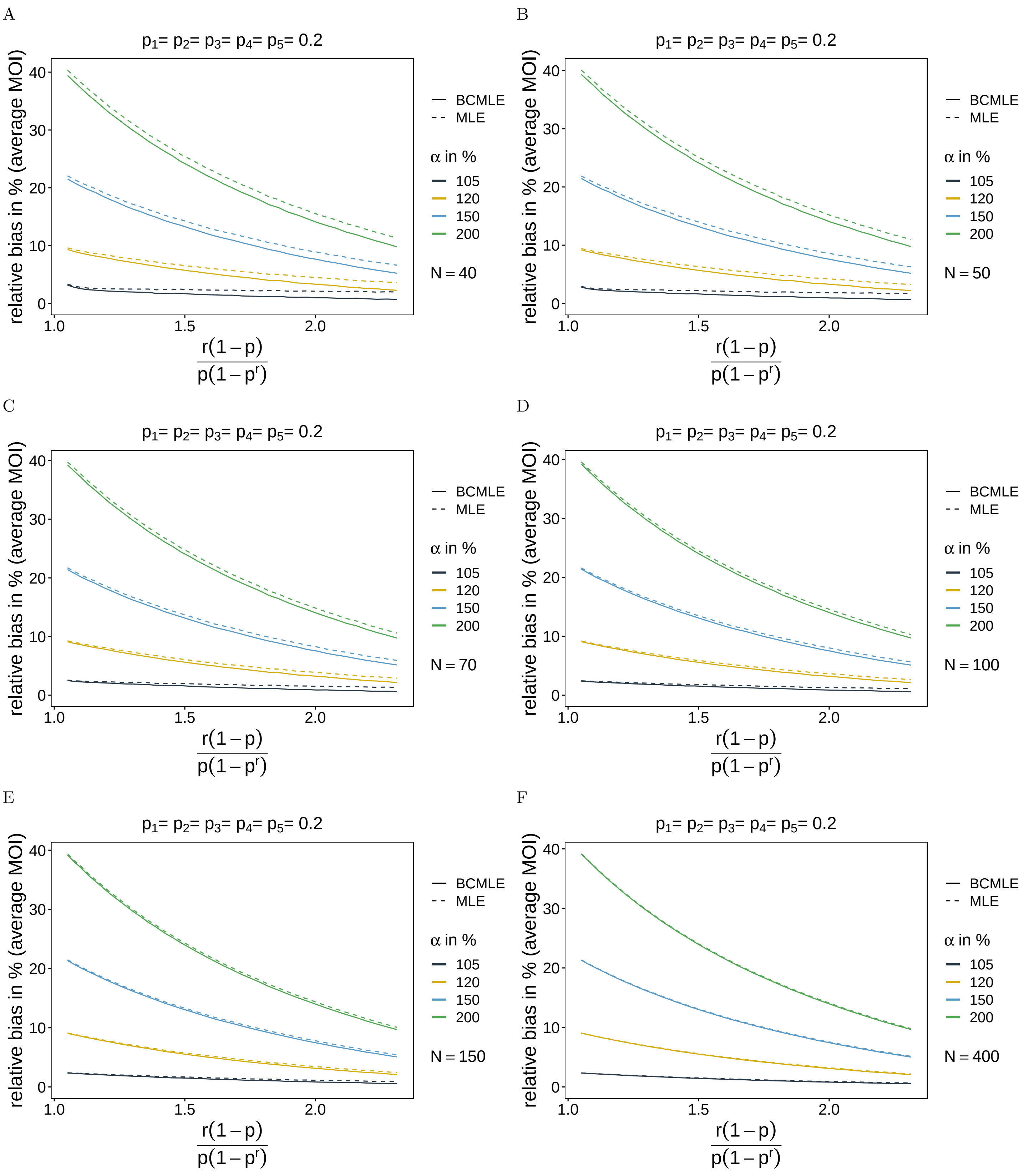

Figure 66: Similar to Figure 60 but for different lineage-frequency distributions.

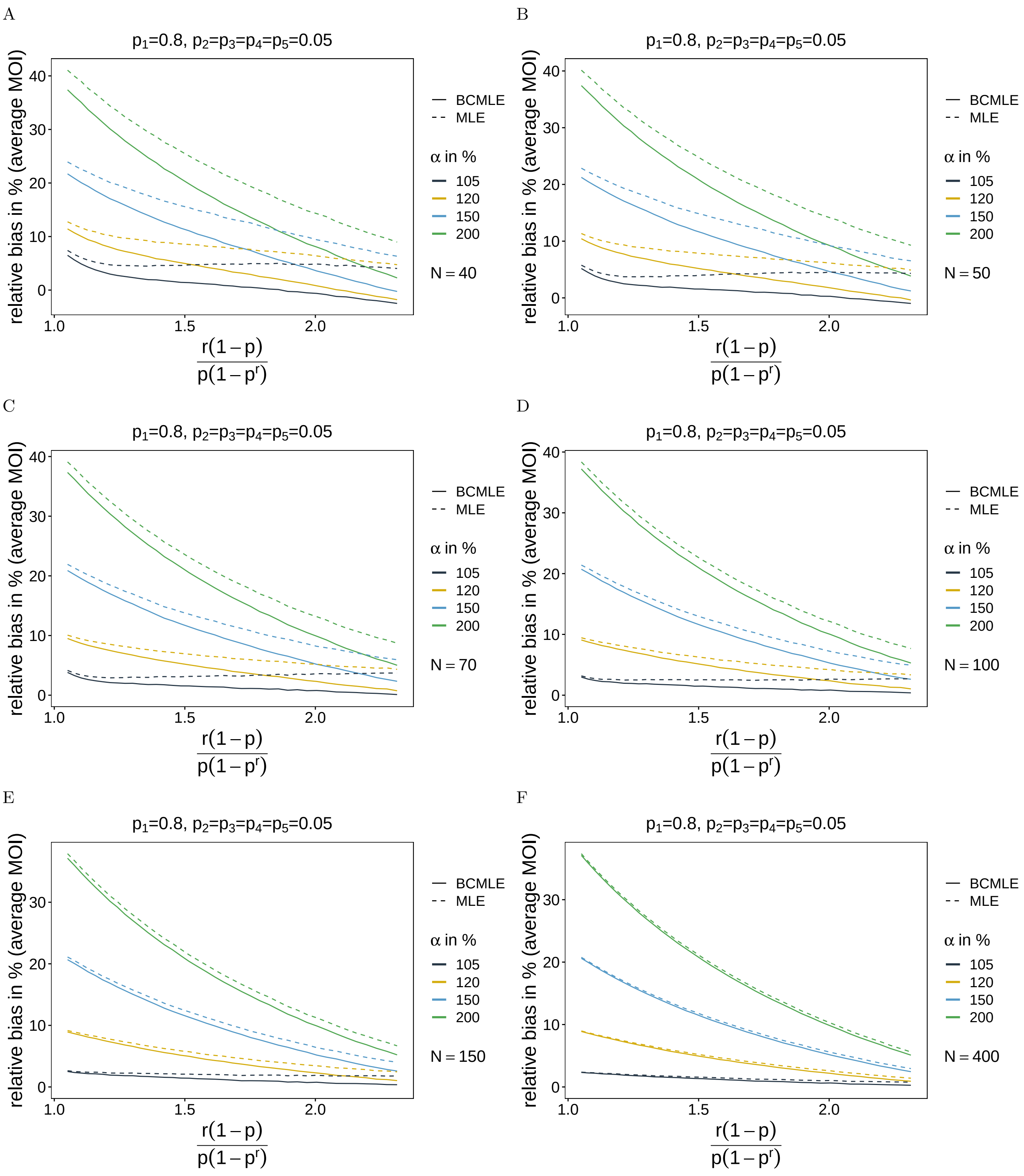

Figure 67: Similar to Figure 60 but for different lineage-frequency distributions.

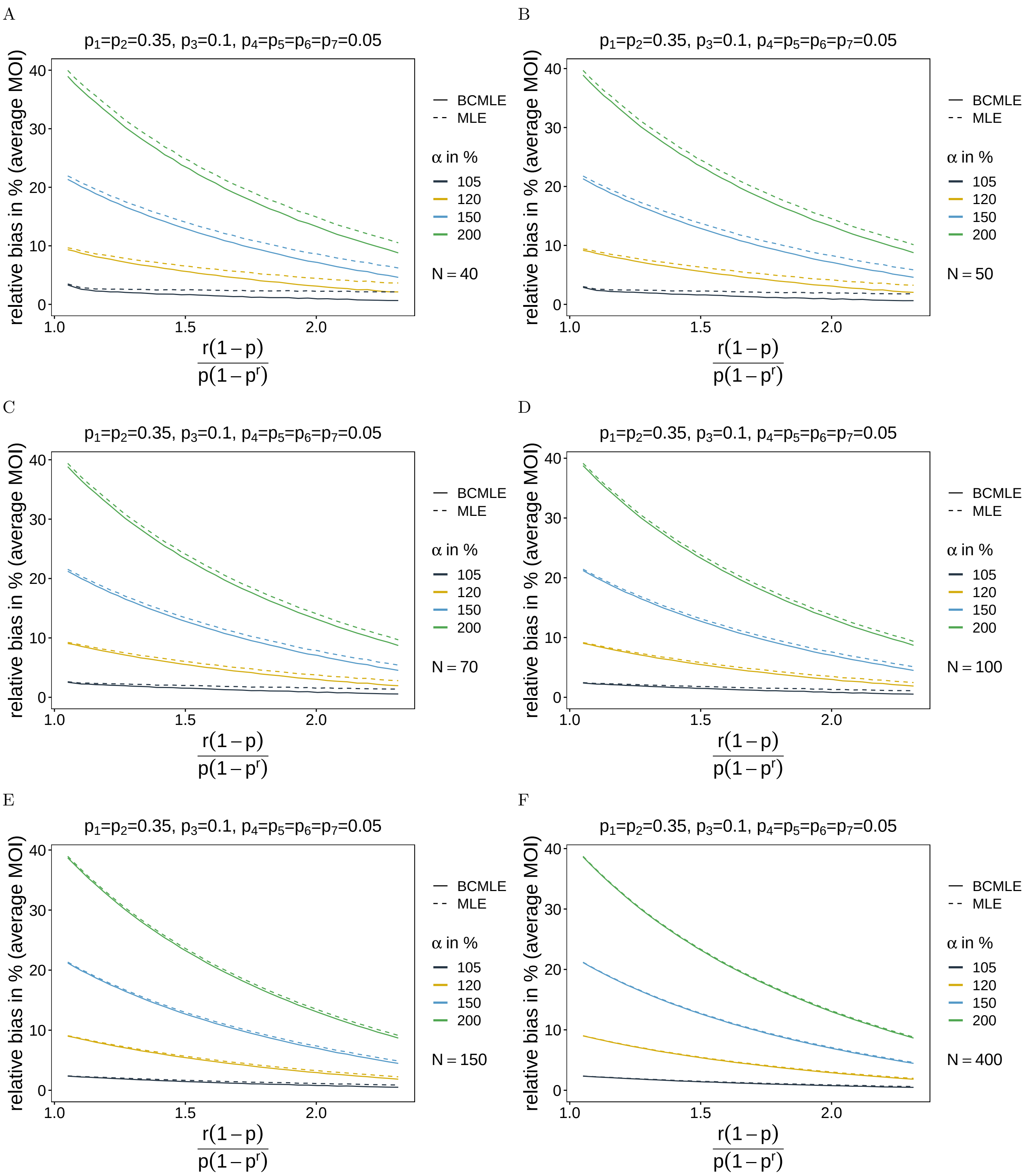

Figure 68: Similar to Figure 60 but for different lineage-frequency distributions.

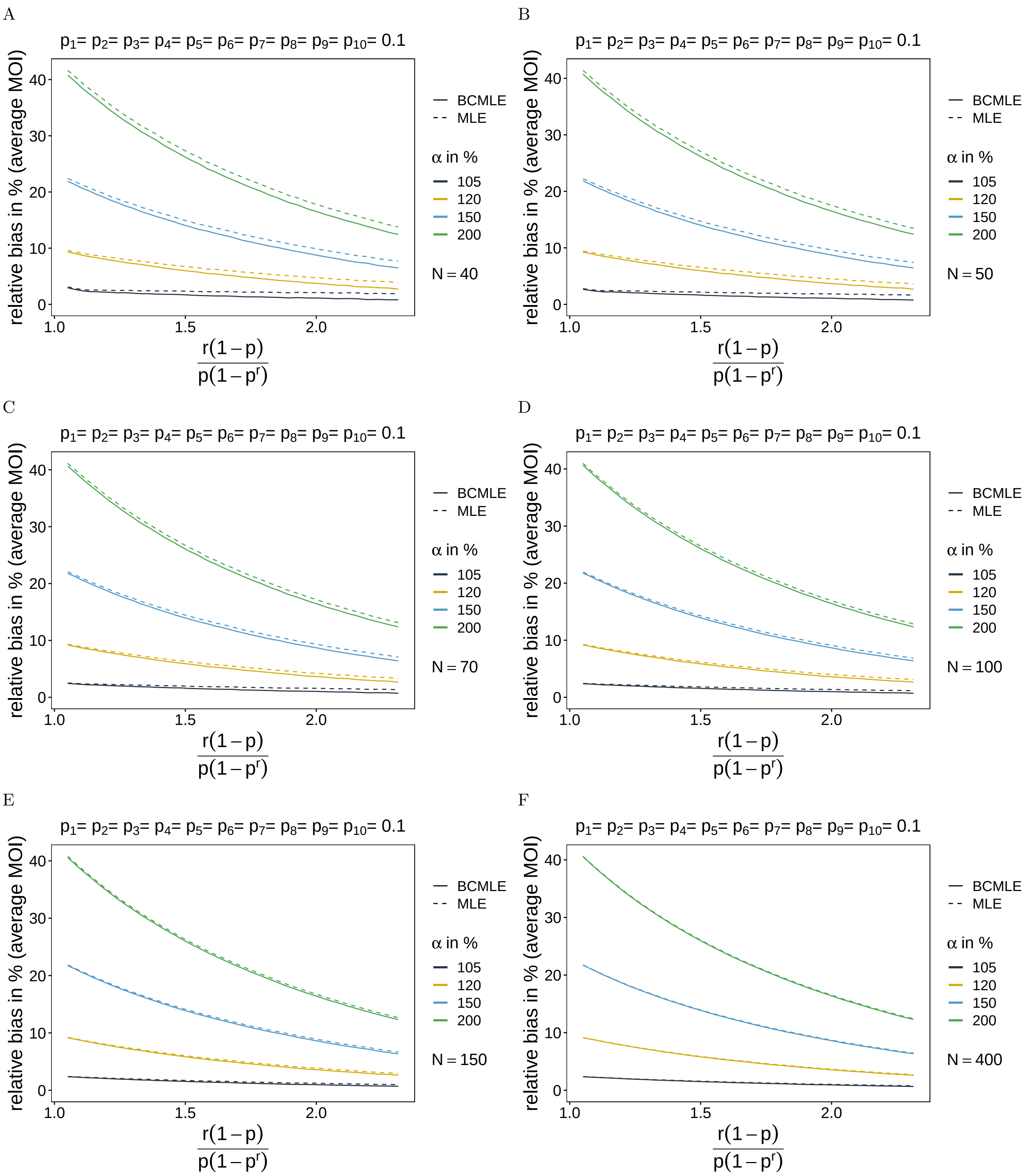

Figure 69: Similar to Figure 60 but for different lineage-frequency distributions.

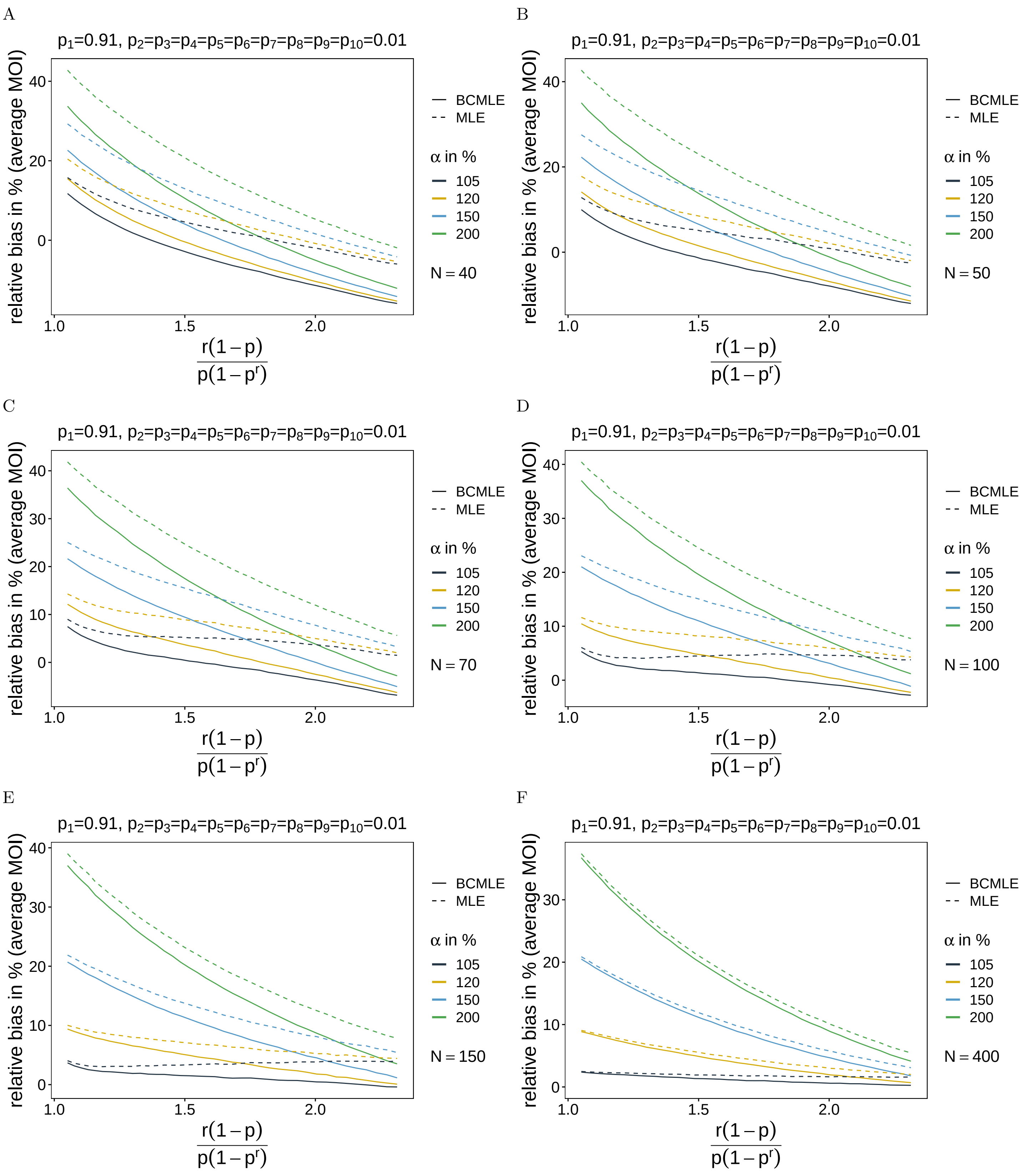

Figure 70: Similar to Figure 60 but for different lineage-frequency distributions.

5.2 CV of the BCMLE in %

5.2.1 Different levels of overdispersion

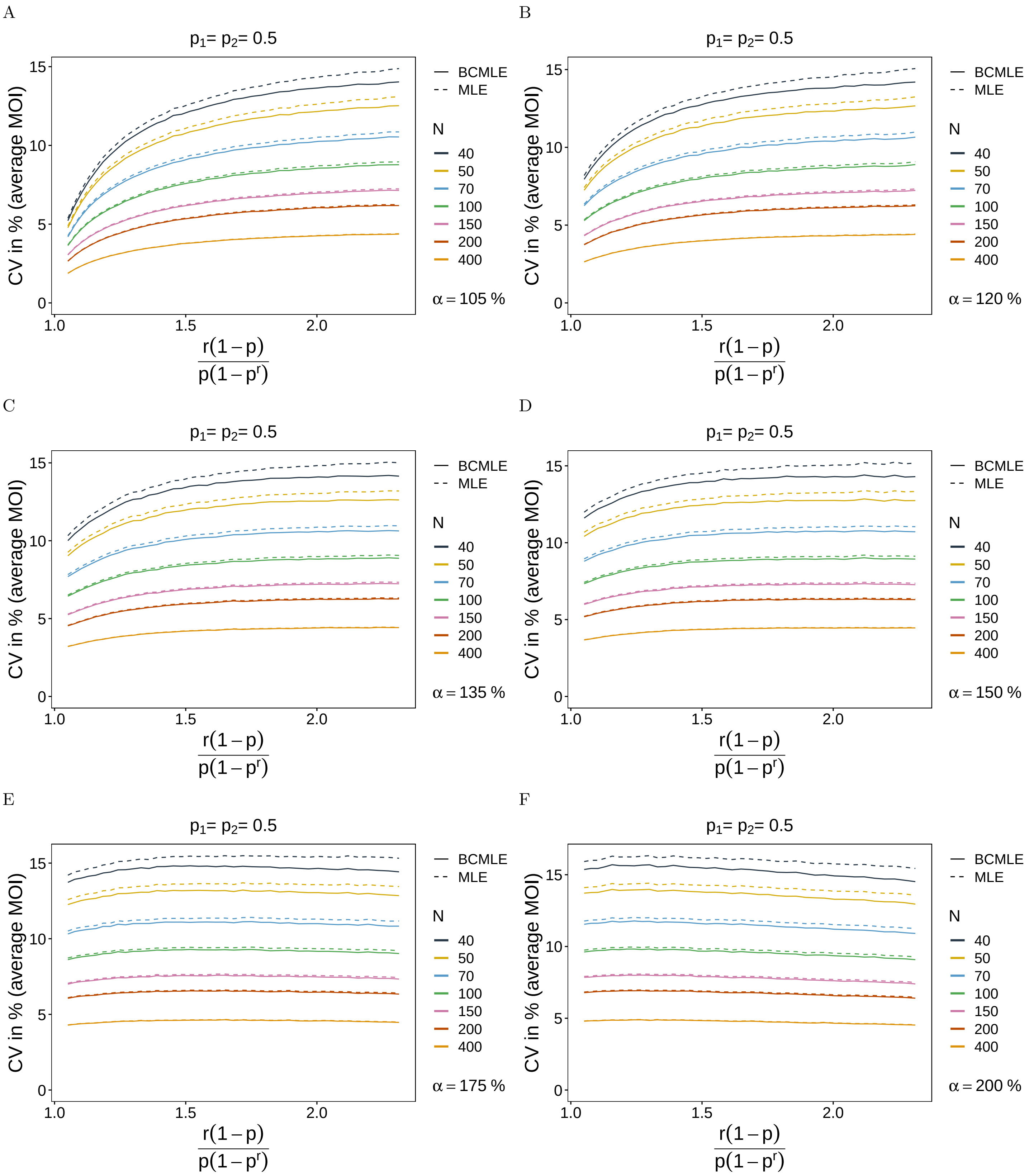

Figure 71: **Variance of MOI estimates under model violations**. Similar to Figure 49 but for the coefficient of variation in %.

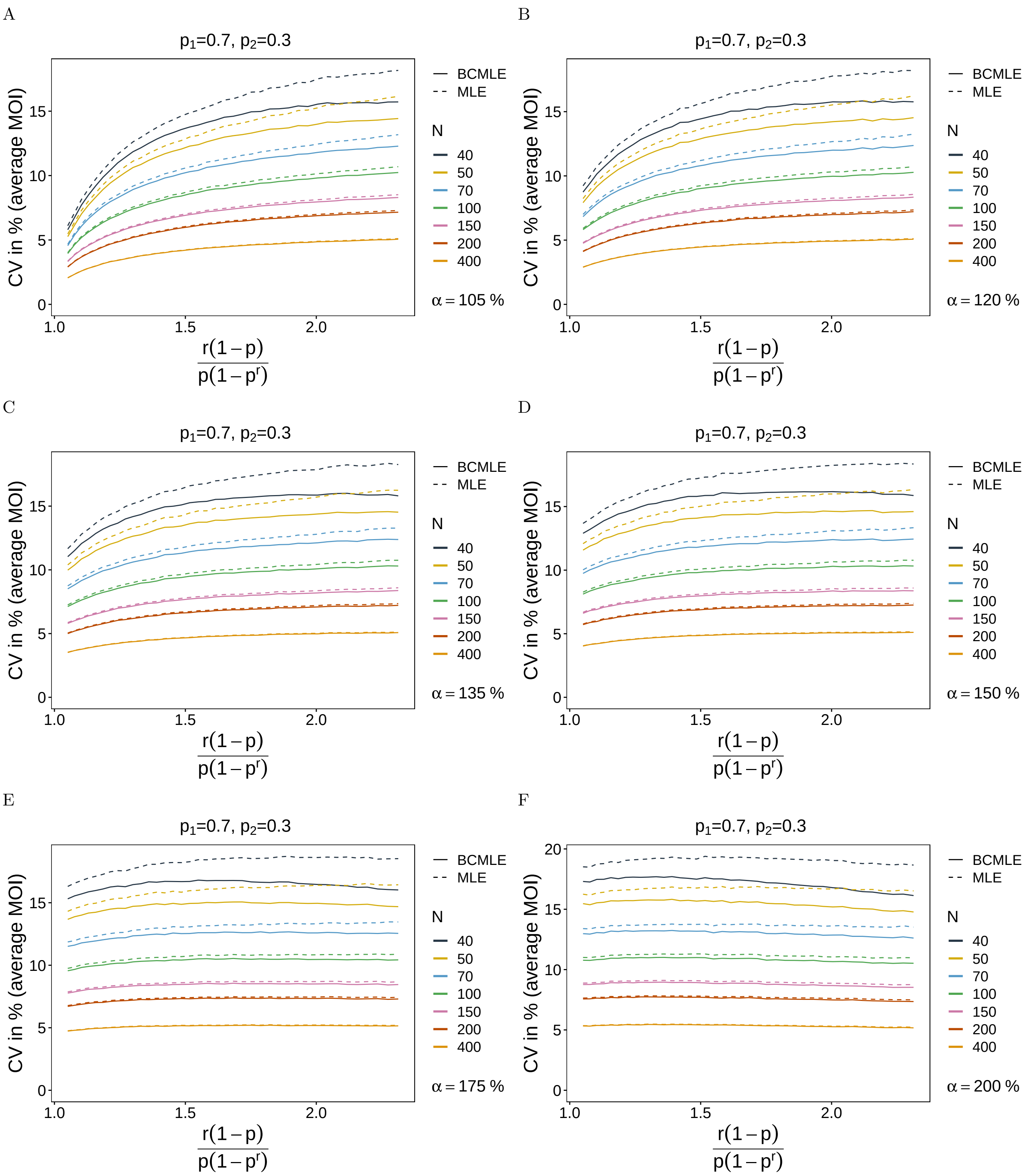

Figure 72: Similar to Figure 71 but for different lineage-frequency distributions.

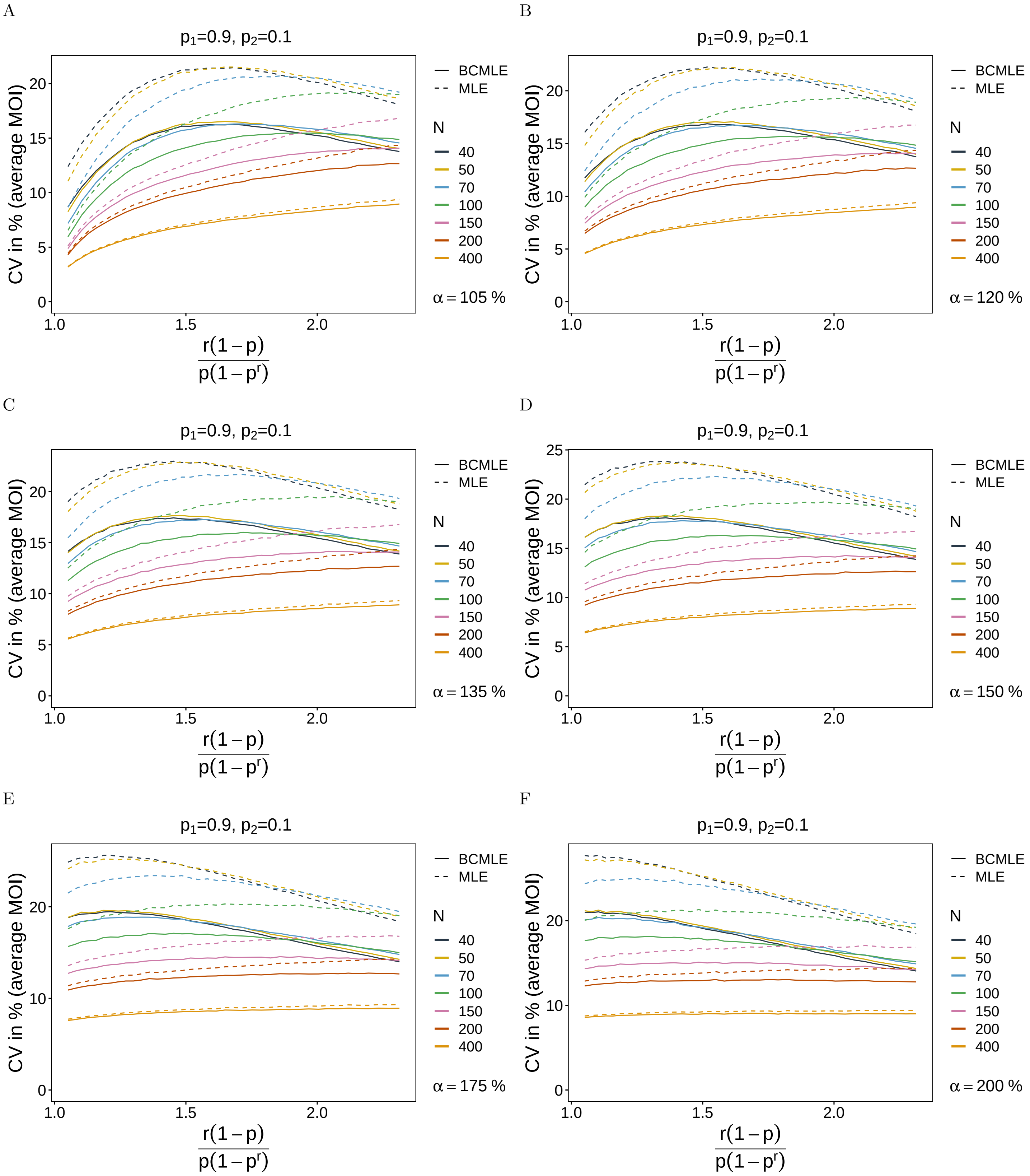

Figure 73: Similar to Figure 71 but for different lineage-frequency distributions.

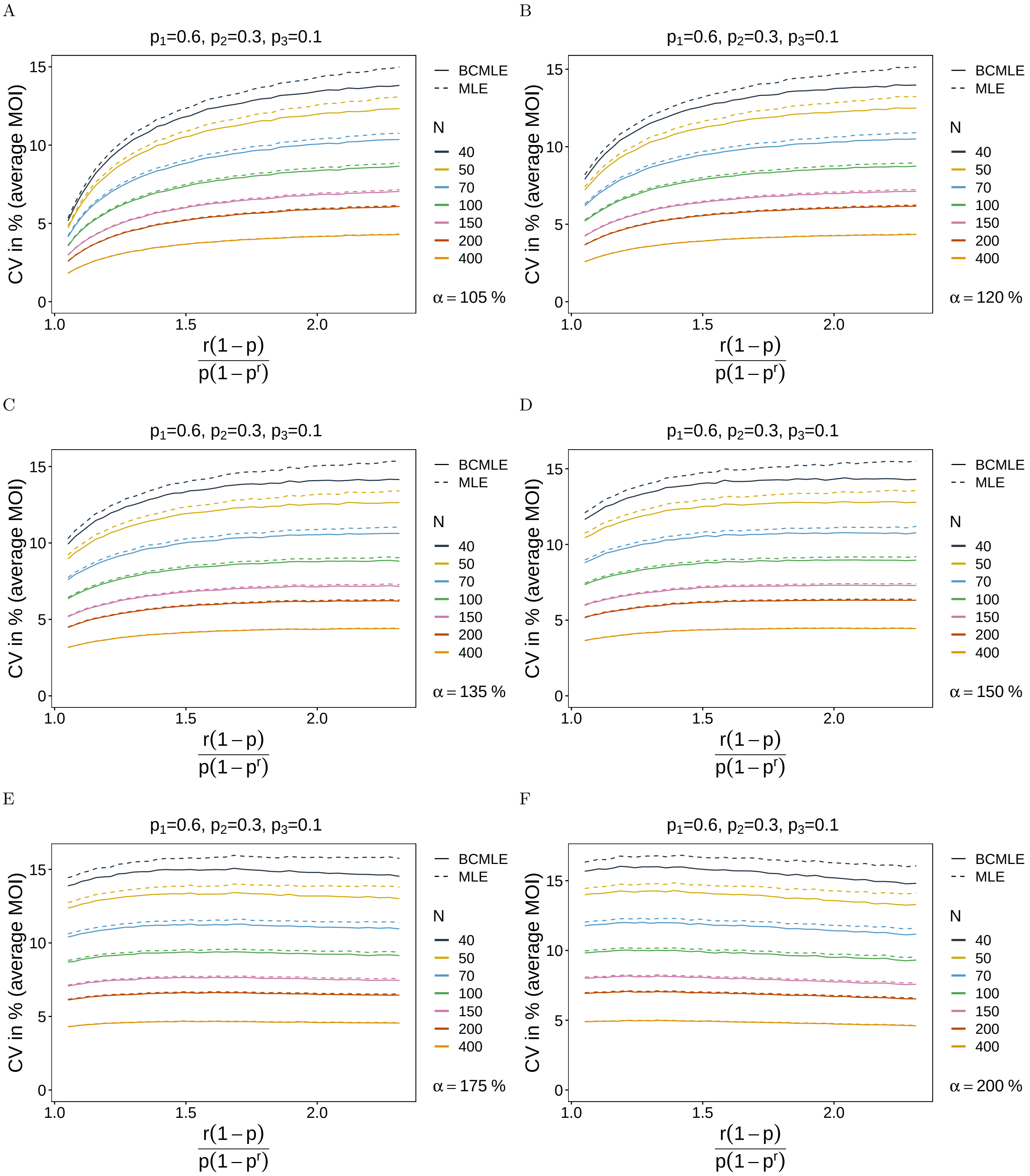

Figure 74: Similar to Figure 71 but for different lineage-frequency distributions.

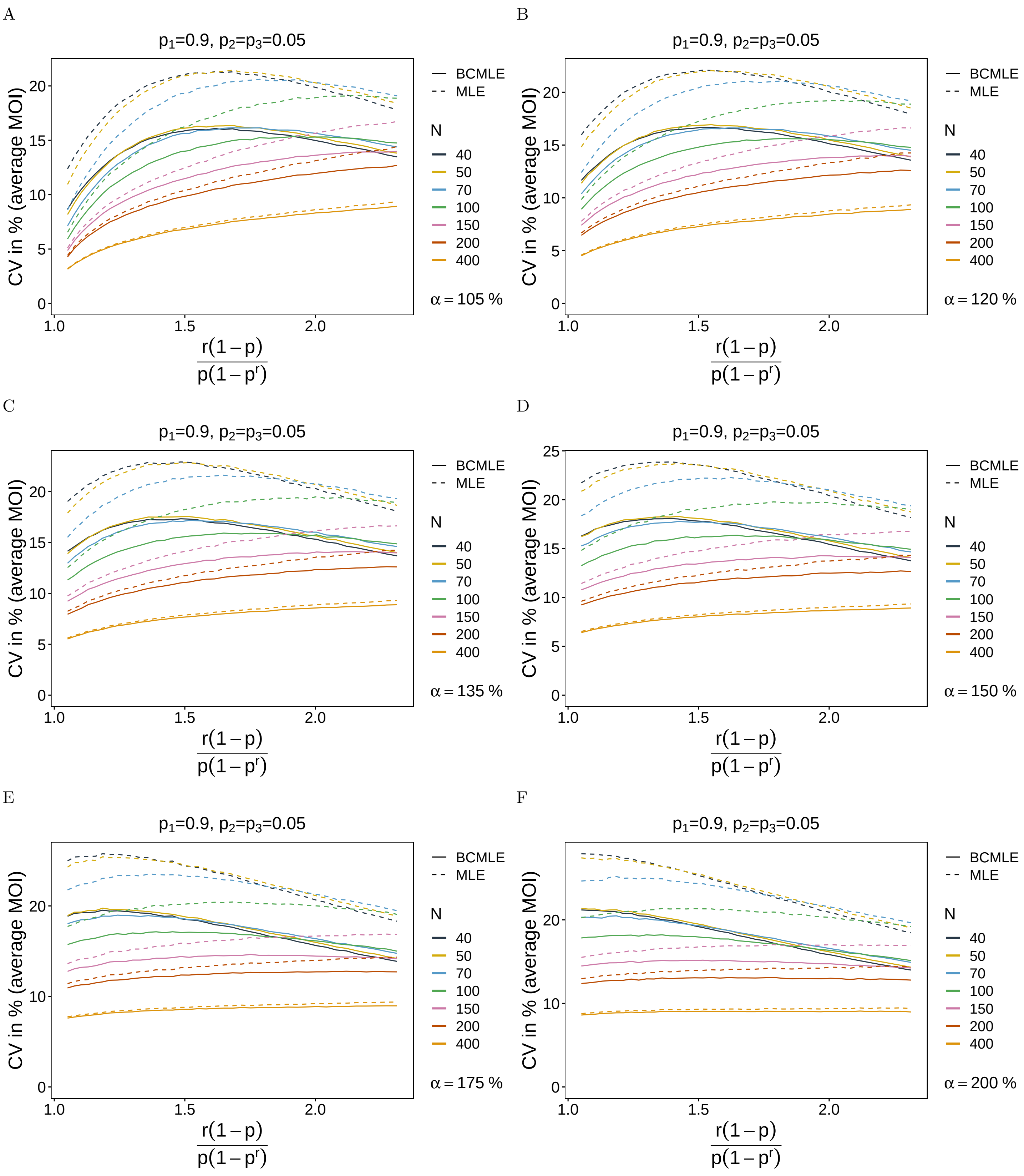

Figure 75: Similar to Figure 71 but for different lineage-frequency distributions.

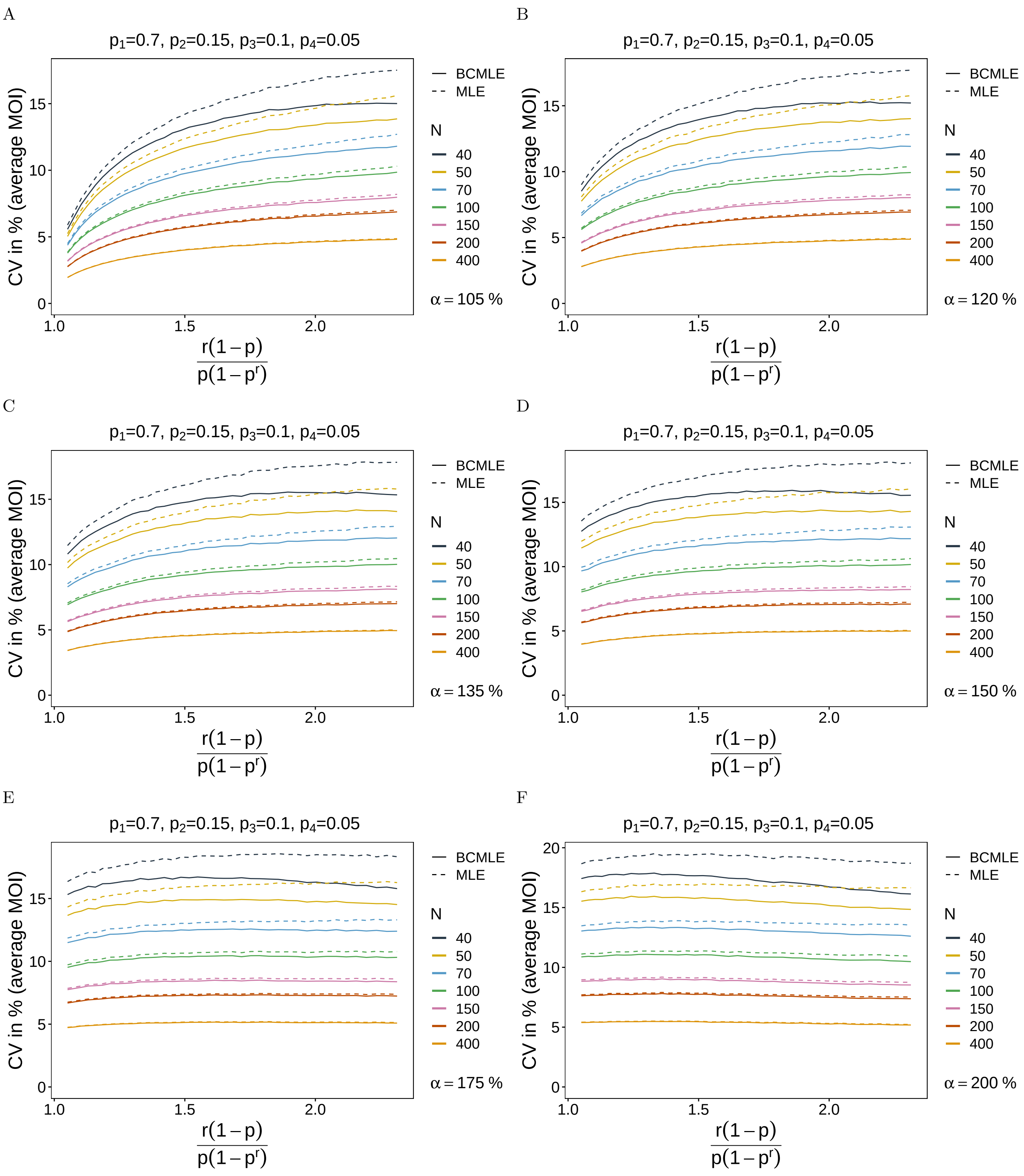

Figure 76: Similar to Figure 71 but for different lineage-frequency distributions.

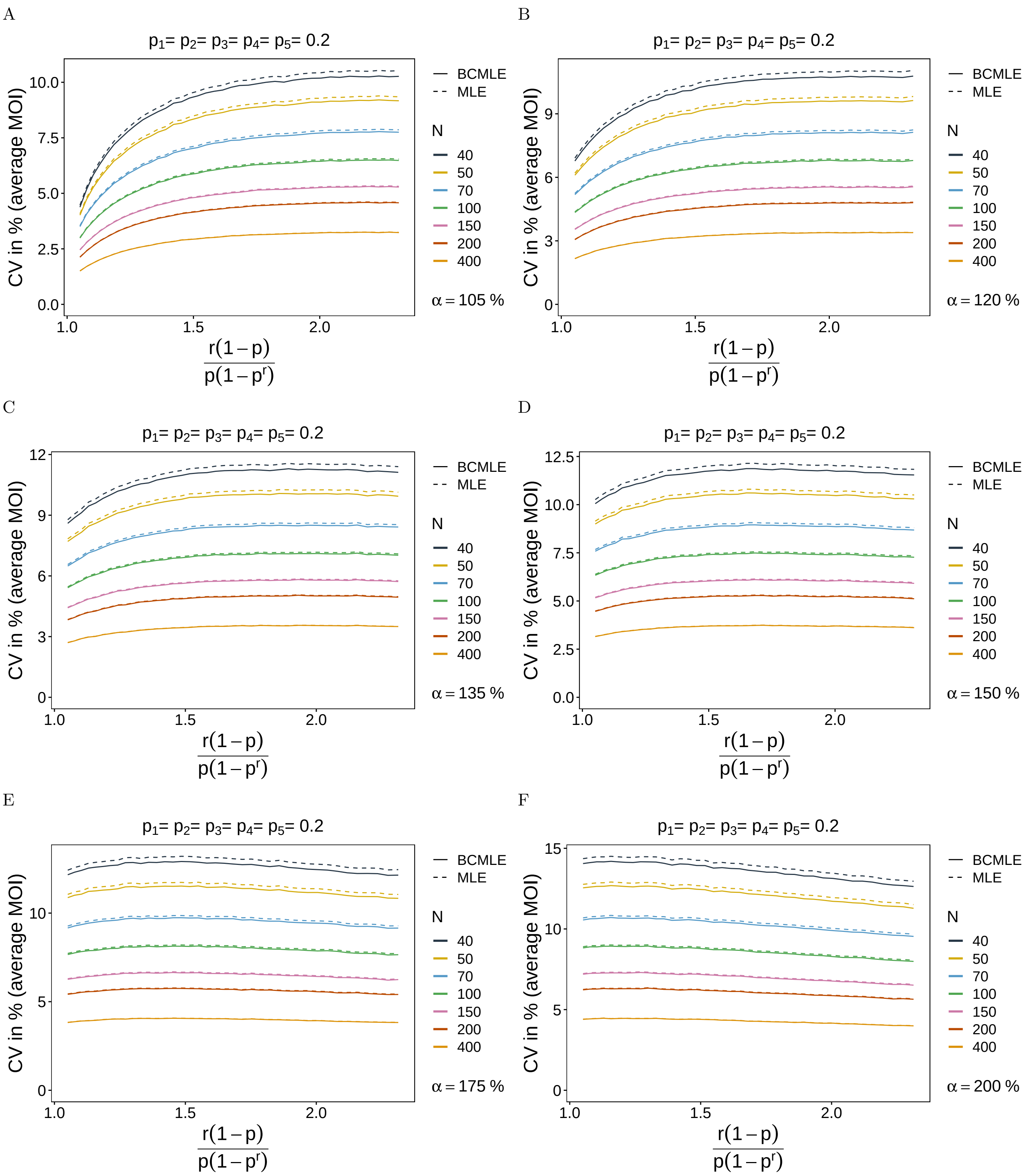

Figure 77: Similar to Figure 71 but for different lineage-frequency distributions.

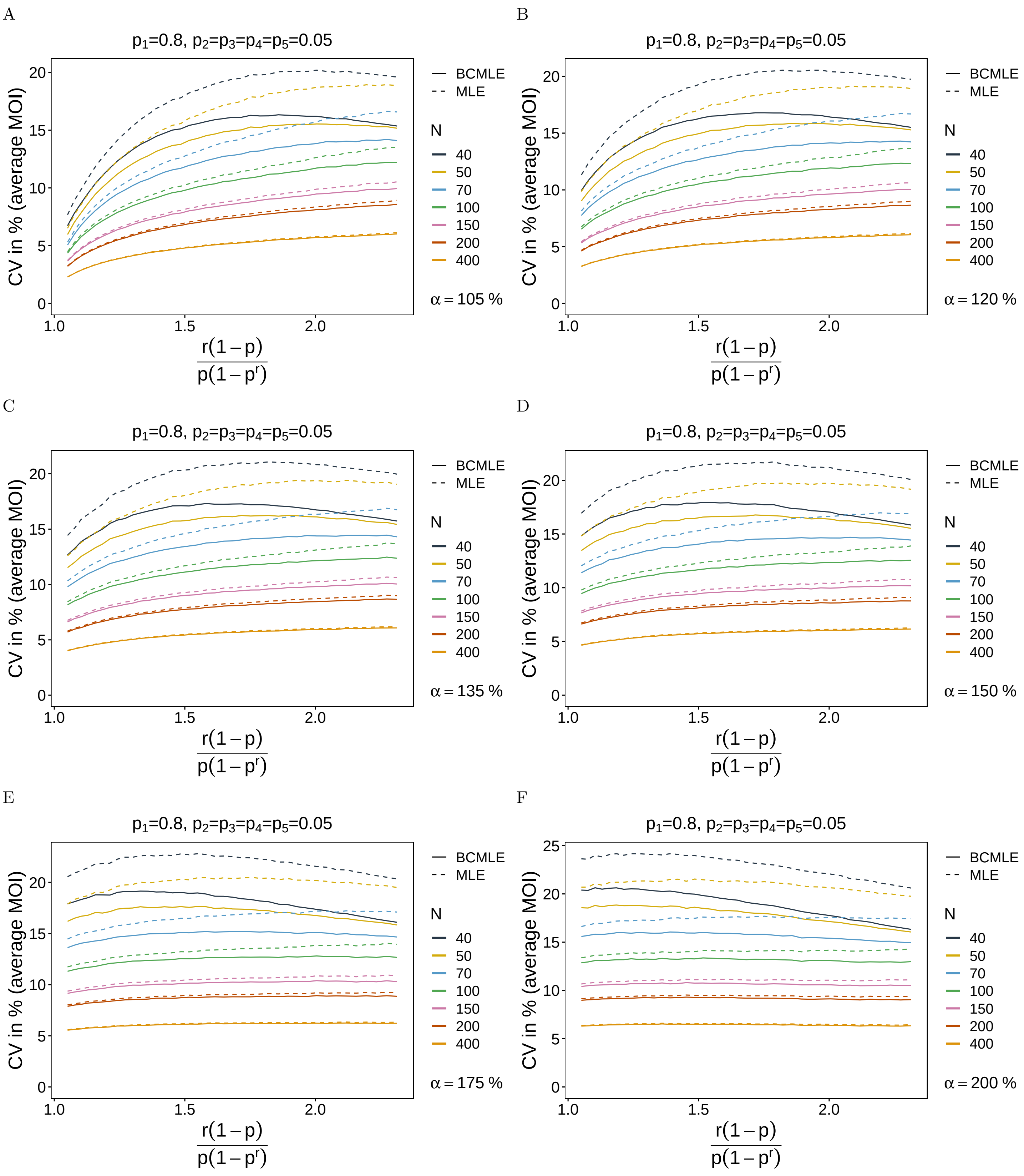

Figure 78: Similar to Figure 71 but for different lineage-frequency distributions.

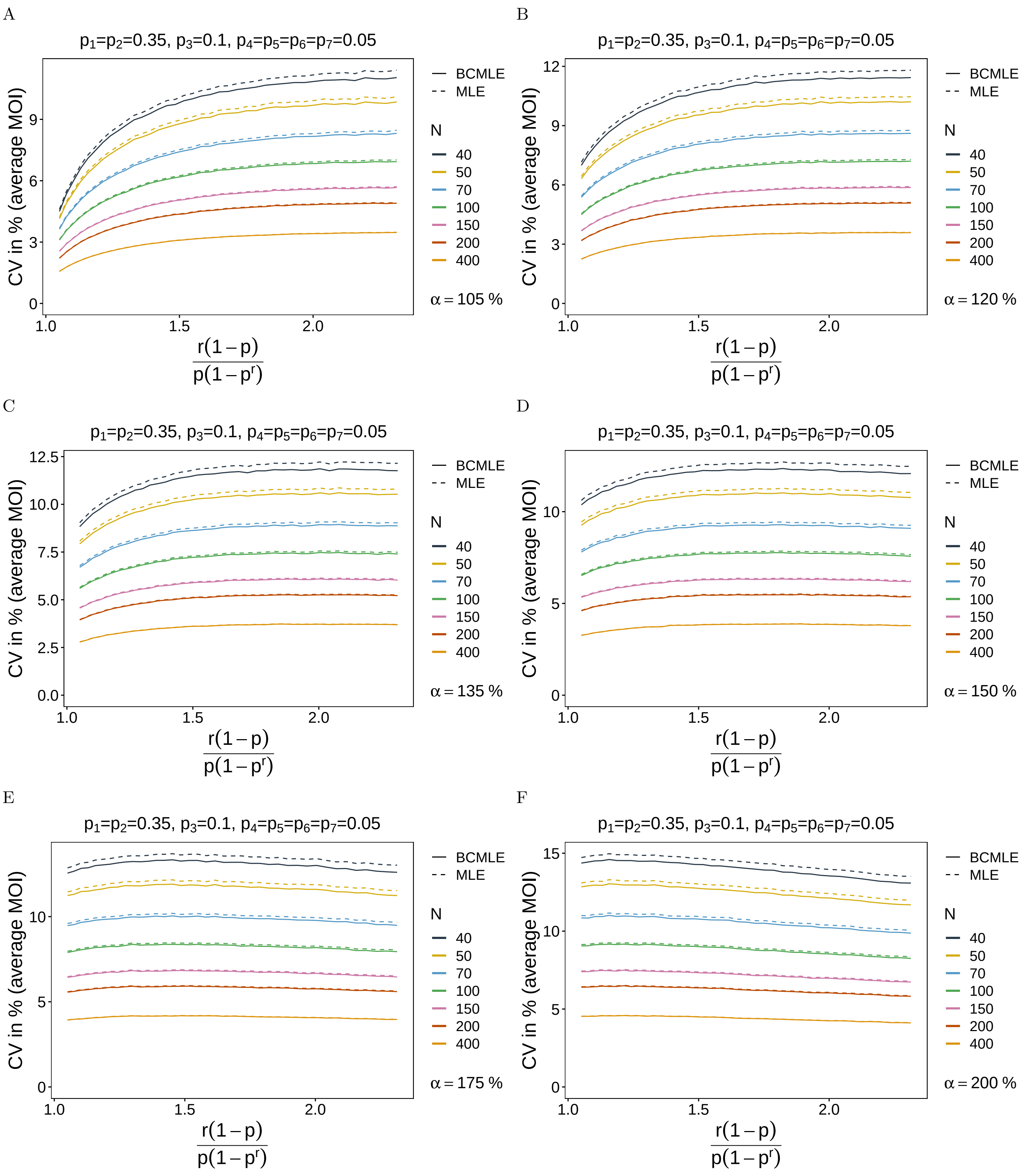

Figure 79: Similar to Figure 71 but for different lineage-frequency distributions.

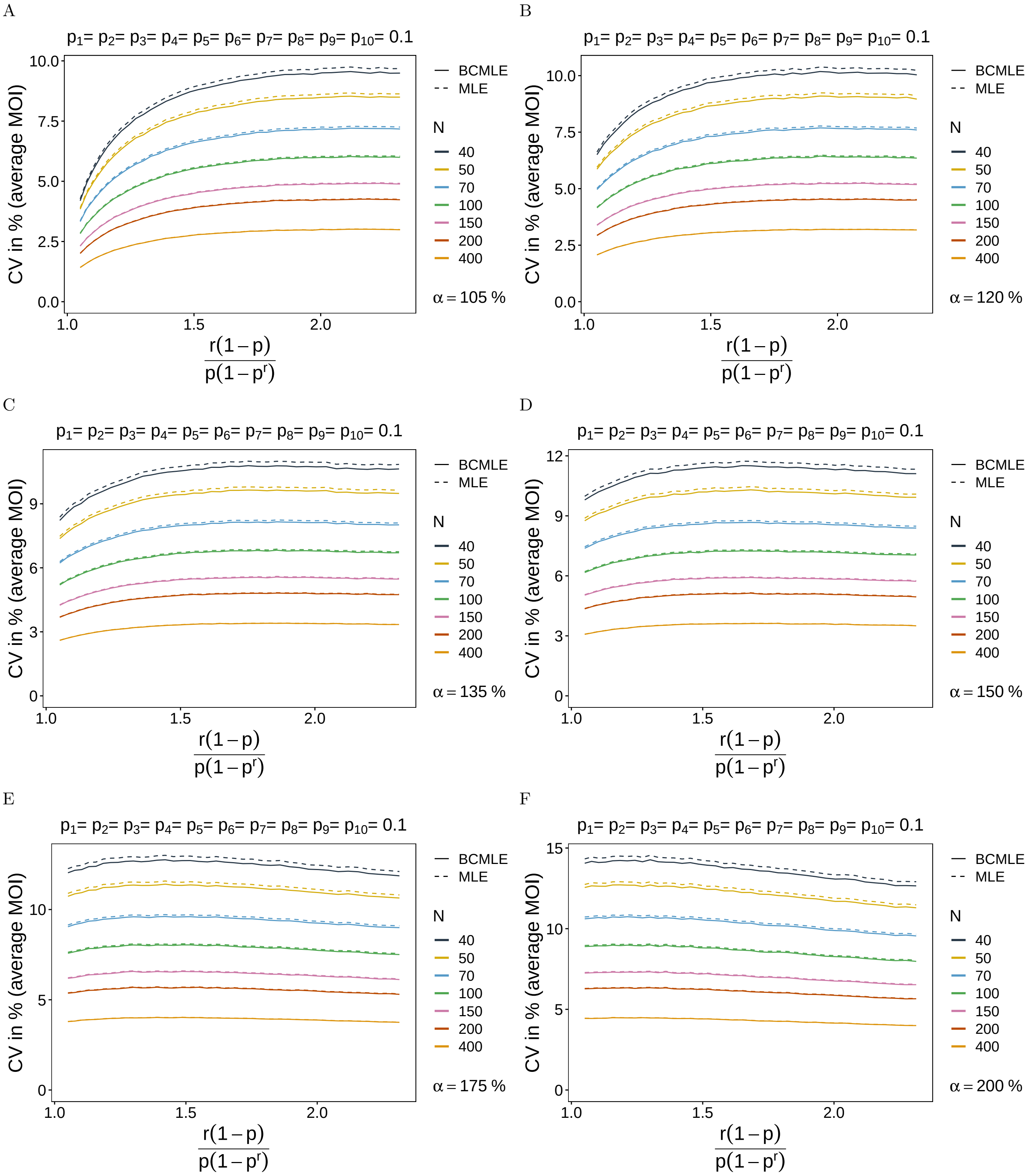

Figure 80: Similar to Figure 71 but for different lineage-frequency distributions.

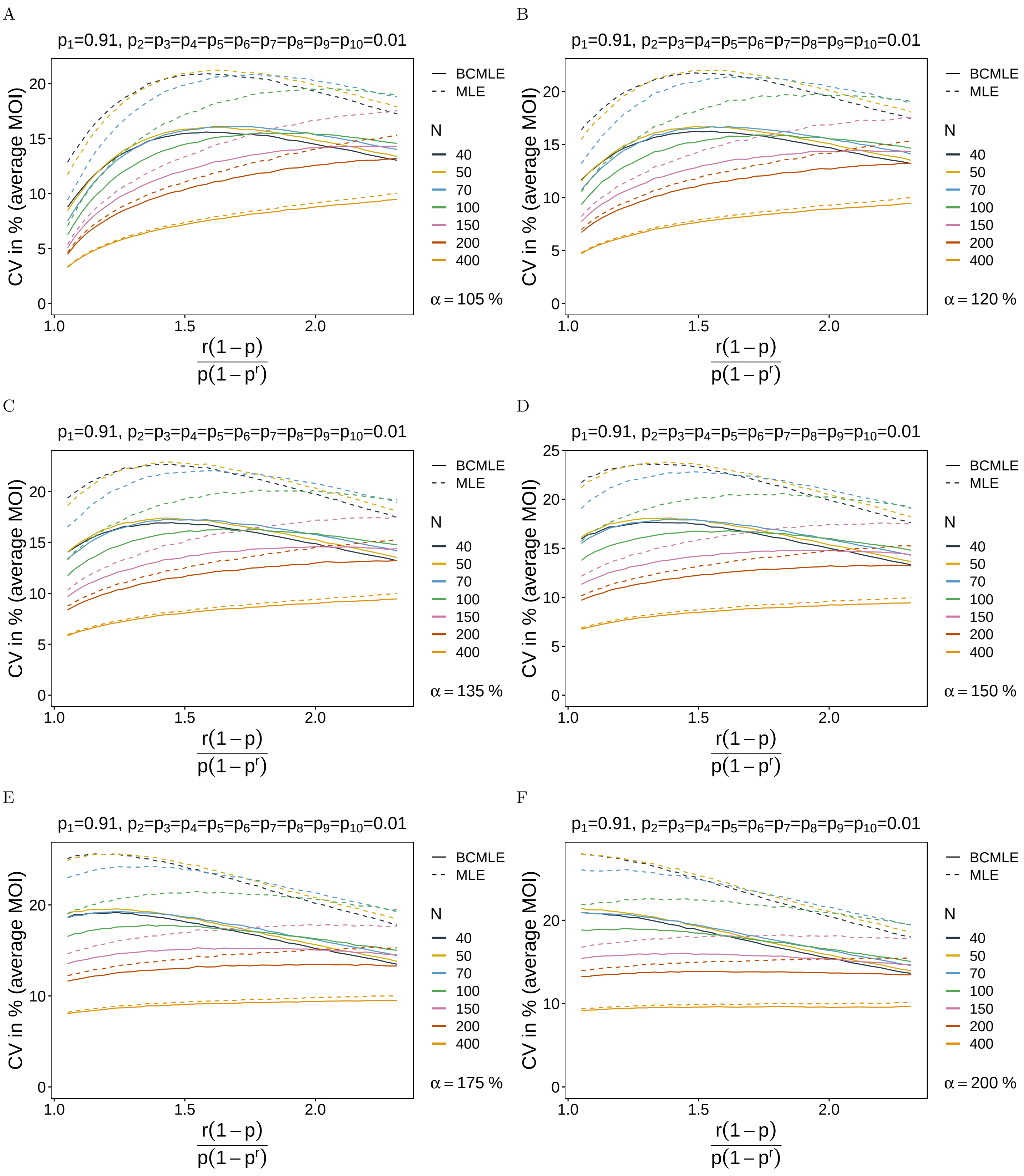

Figure 81: Similar to Figure 71 but for different lineage-frequency distributions.

5.2.2 Different sample sizes

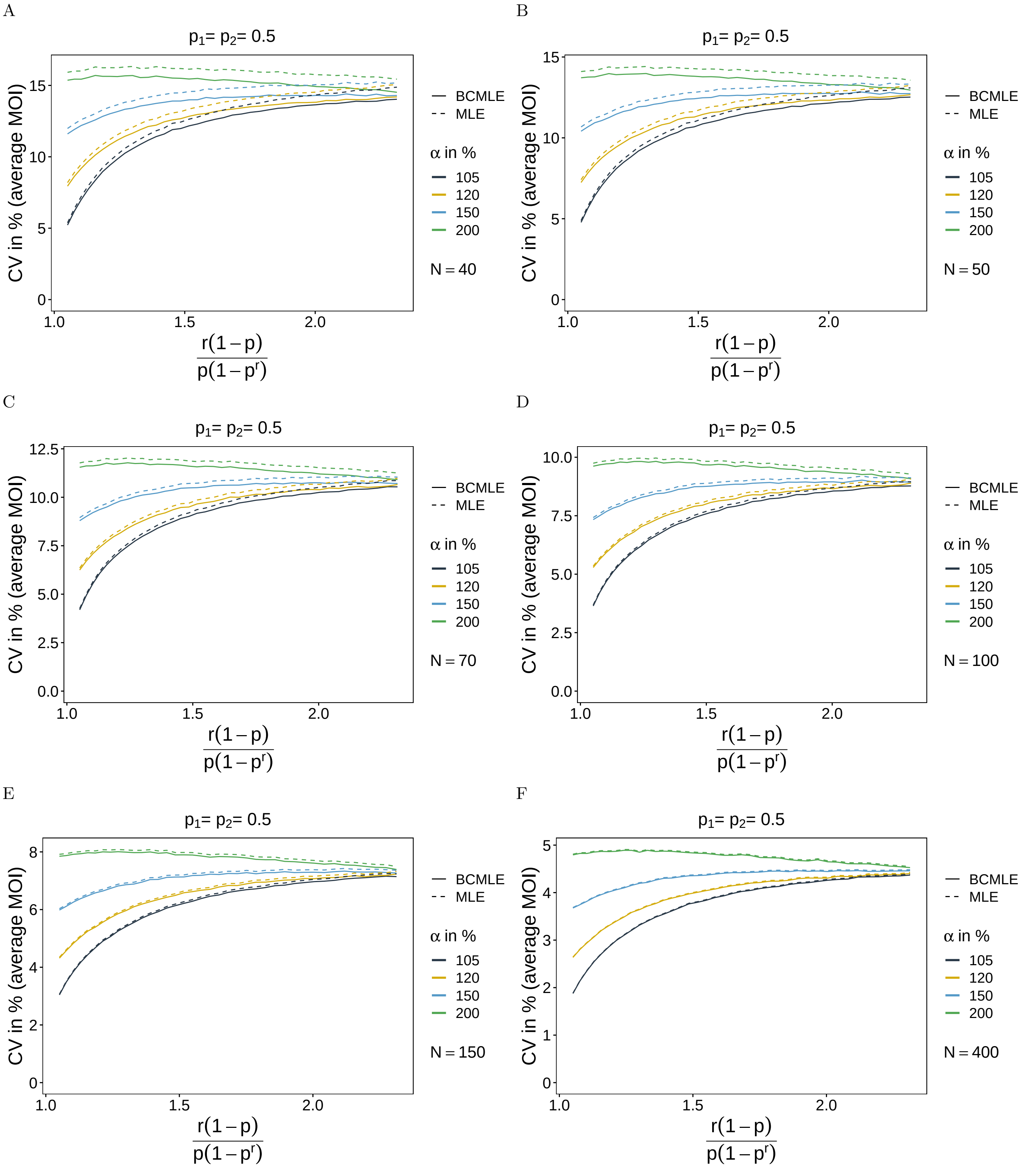

Figure 82: **Variance of MOI estimates under model violations**. Similar to Figure 60 but for the coefficient of variation in %.

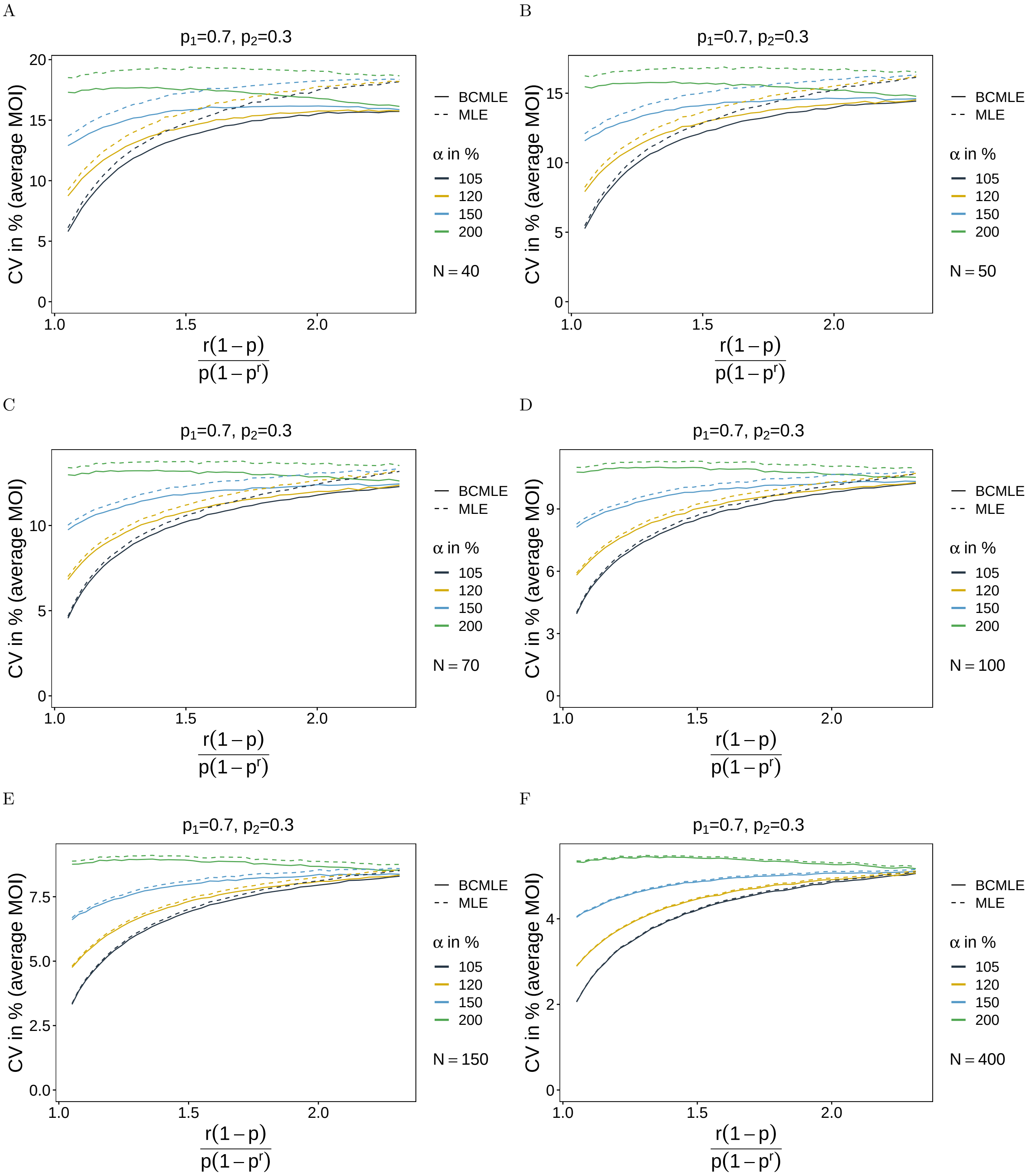

Figure 83: Similar to Figure 82 but for different lineage-frequency distributions.

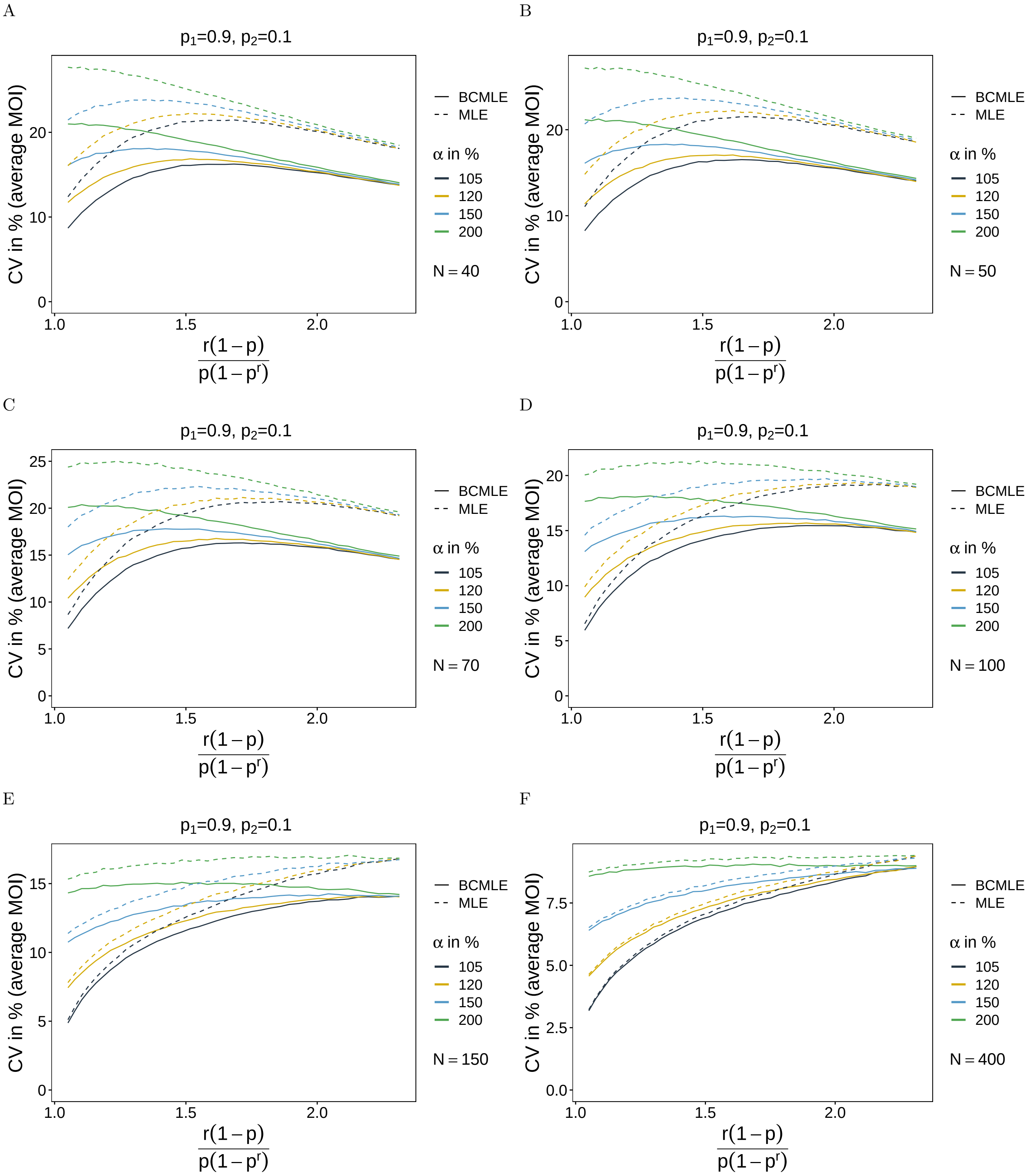

Figure 84: Similar to Figure 82 but for different lineage-frequency distributions.

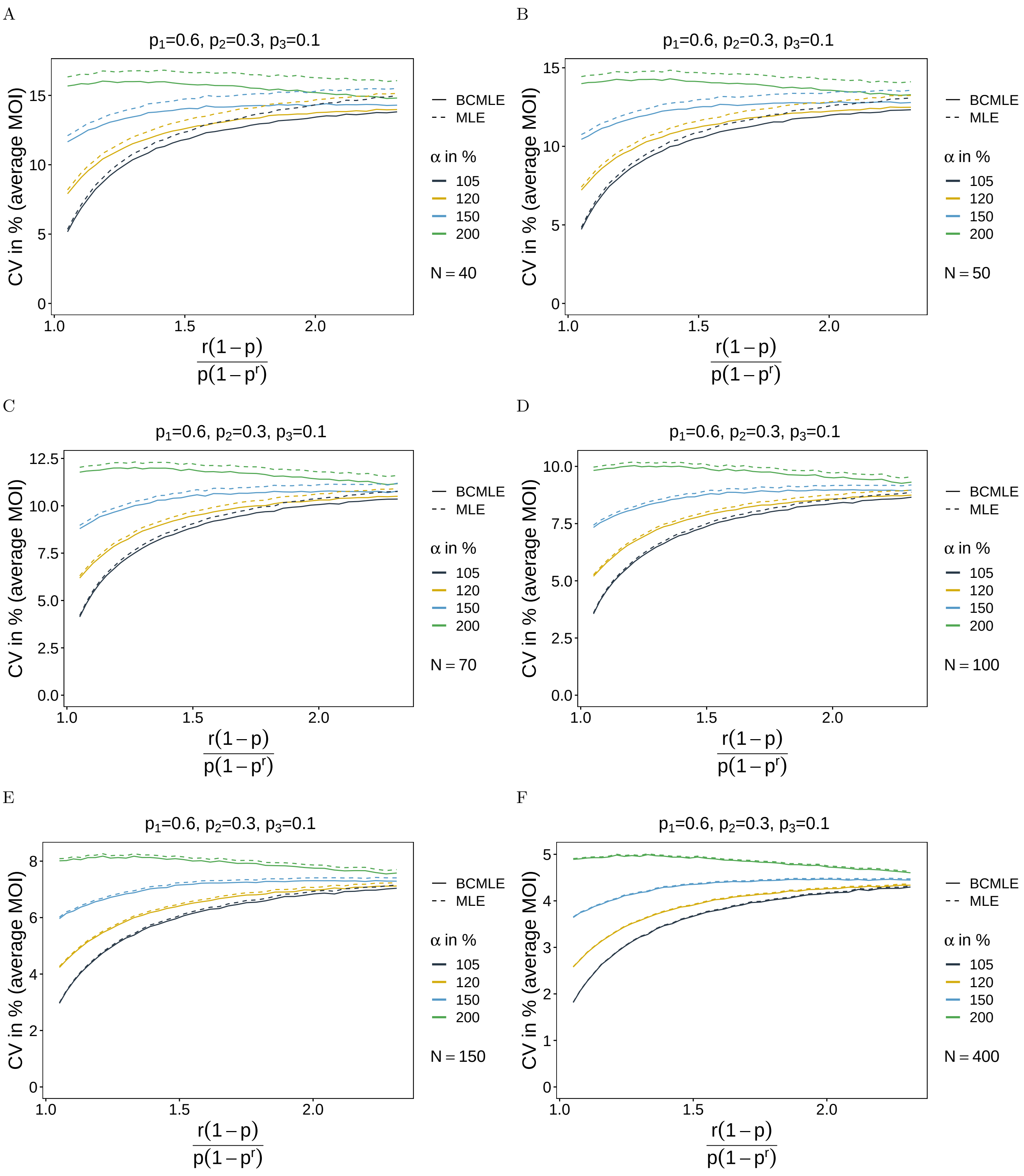

Figure 85: Similar to Figure 82 but for different lineage-frequency distributions.

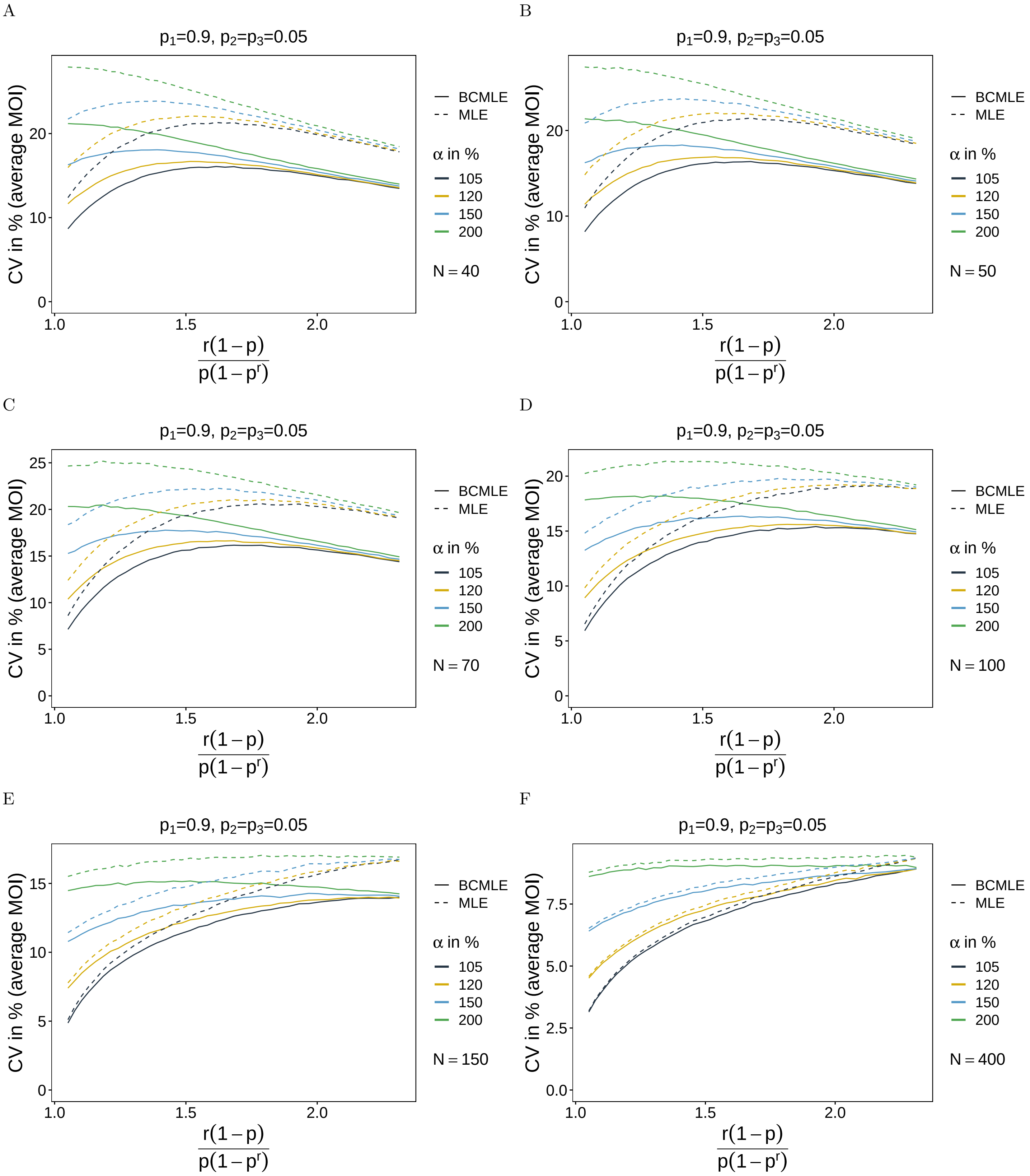

Figure 86: Similar to Figure 82 but for different lineage-frequency distributions.

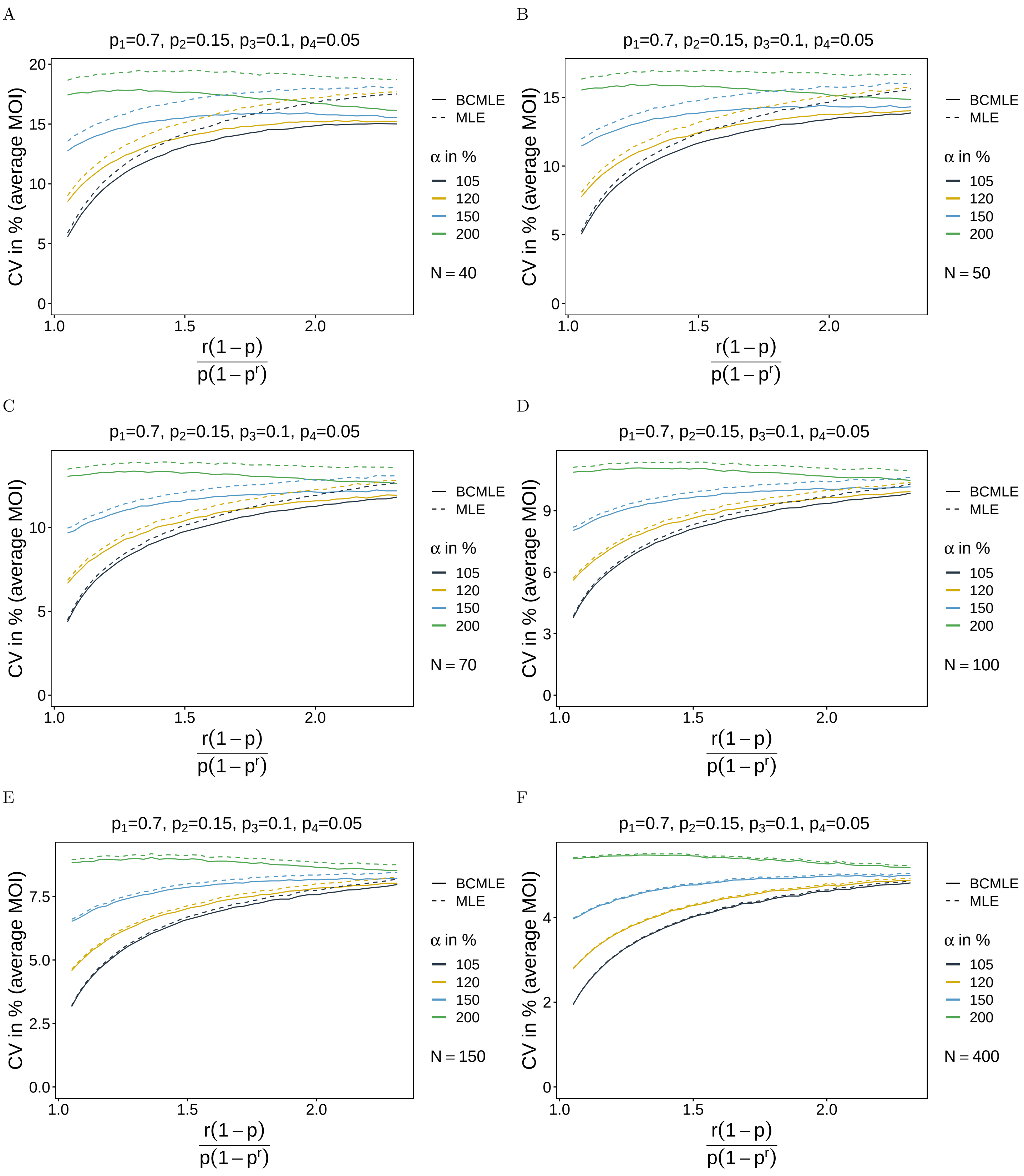

Figure 87: Similar to Figure 82 but for different lineage-frequency distributions.

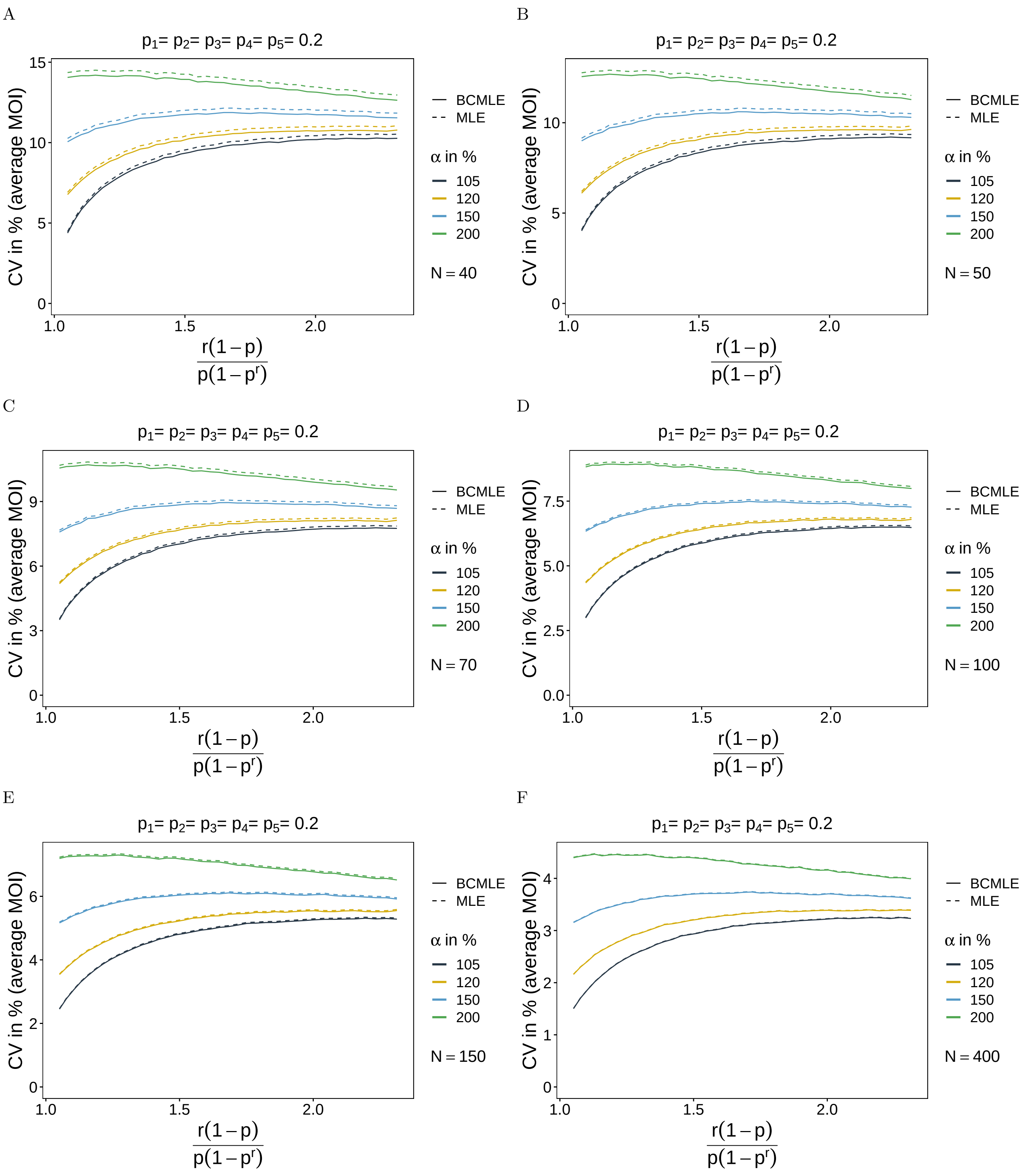

Figure 88: Similar to Figure 82 but for different lineage-frequency distributions.

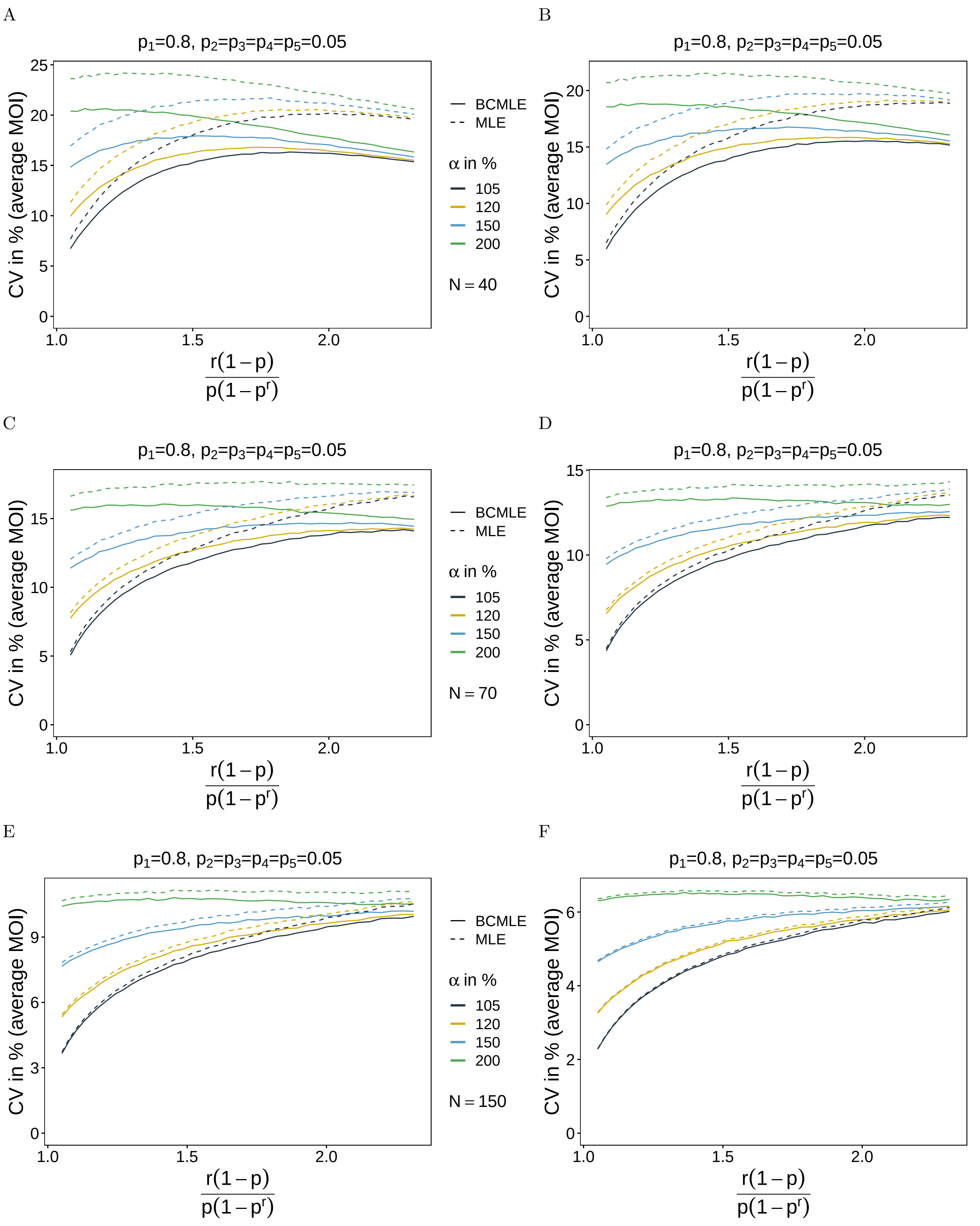

Figure 89: Similar to Figure 82 but for different lineage-frequency distributions.

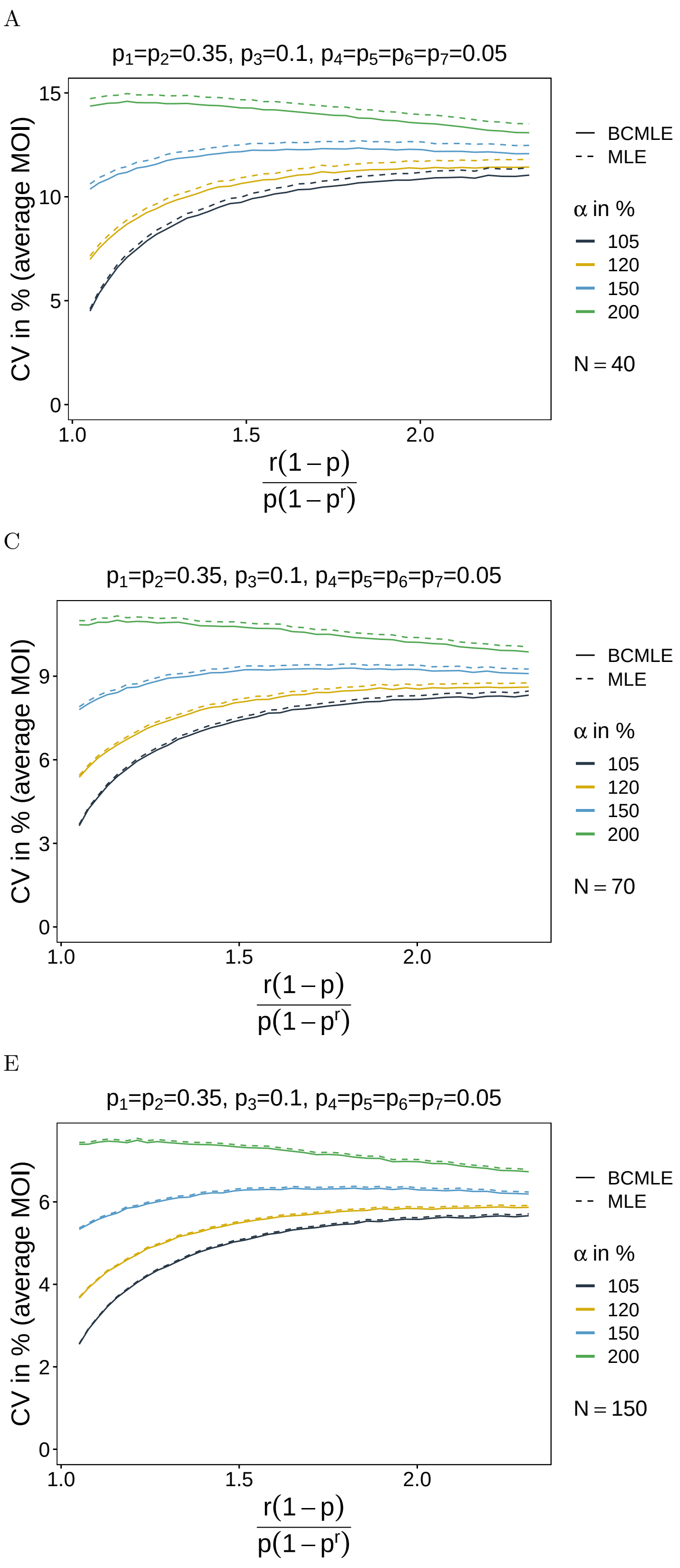

Figure 90: Similar to Figure 82 but for different lineage-frequency distributions.

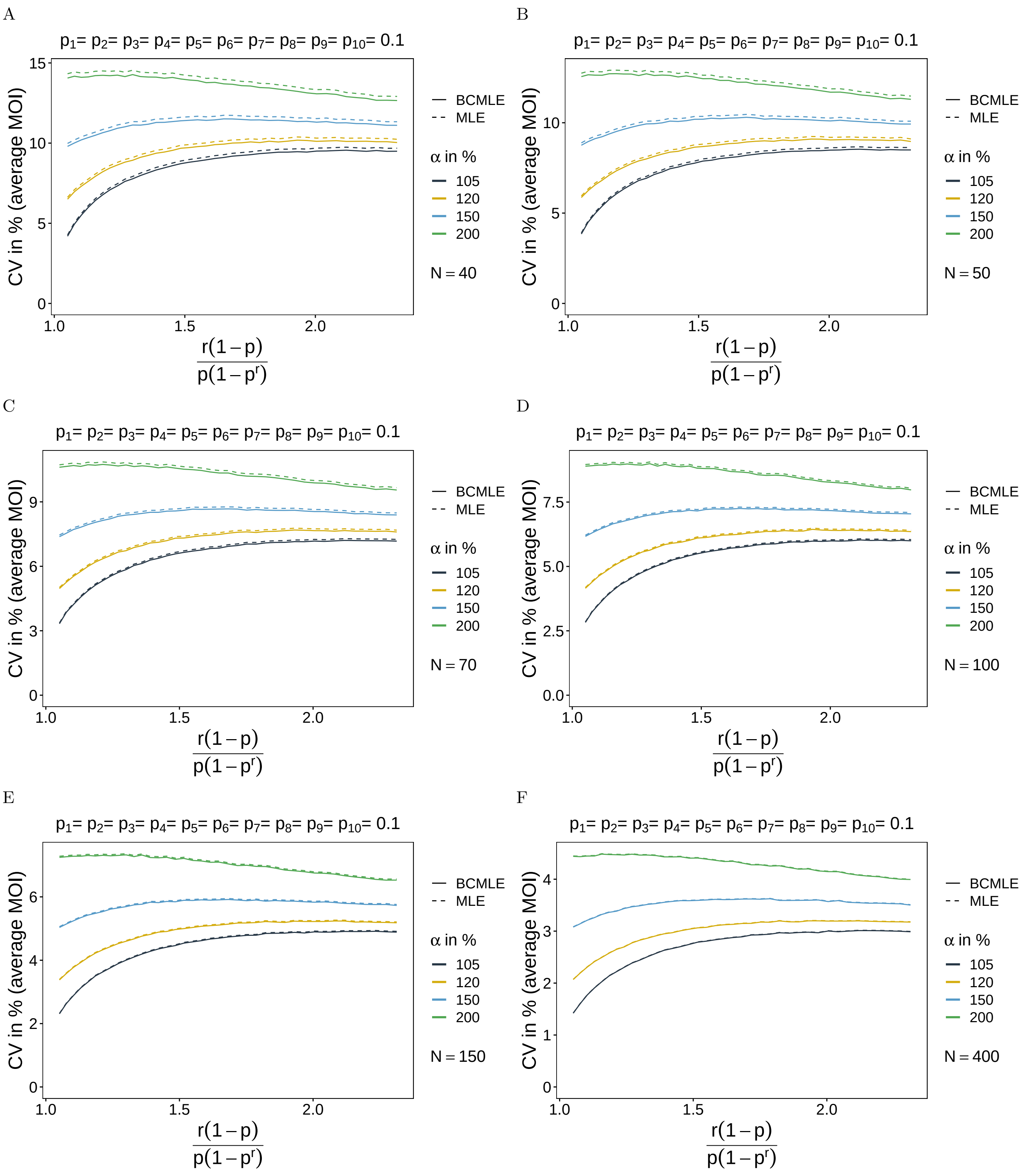

Figure 91: Similar to Figure 82 but for different lineage-frequency distributions.

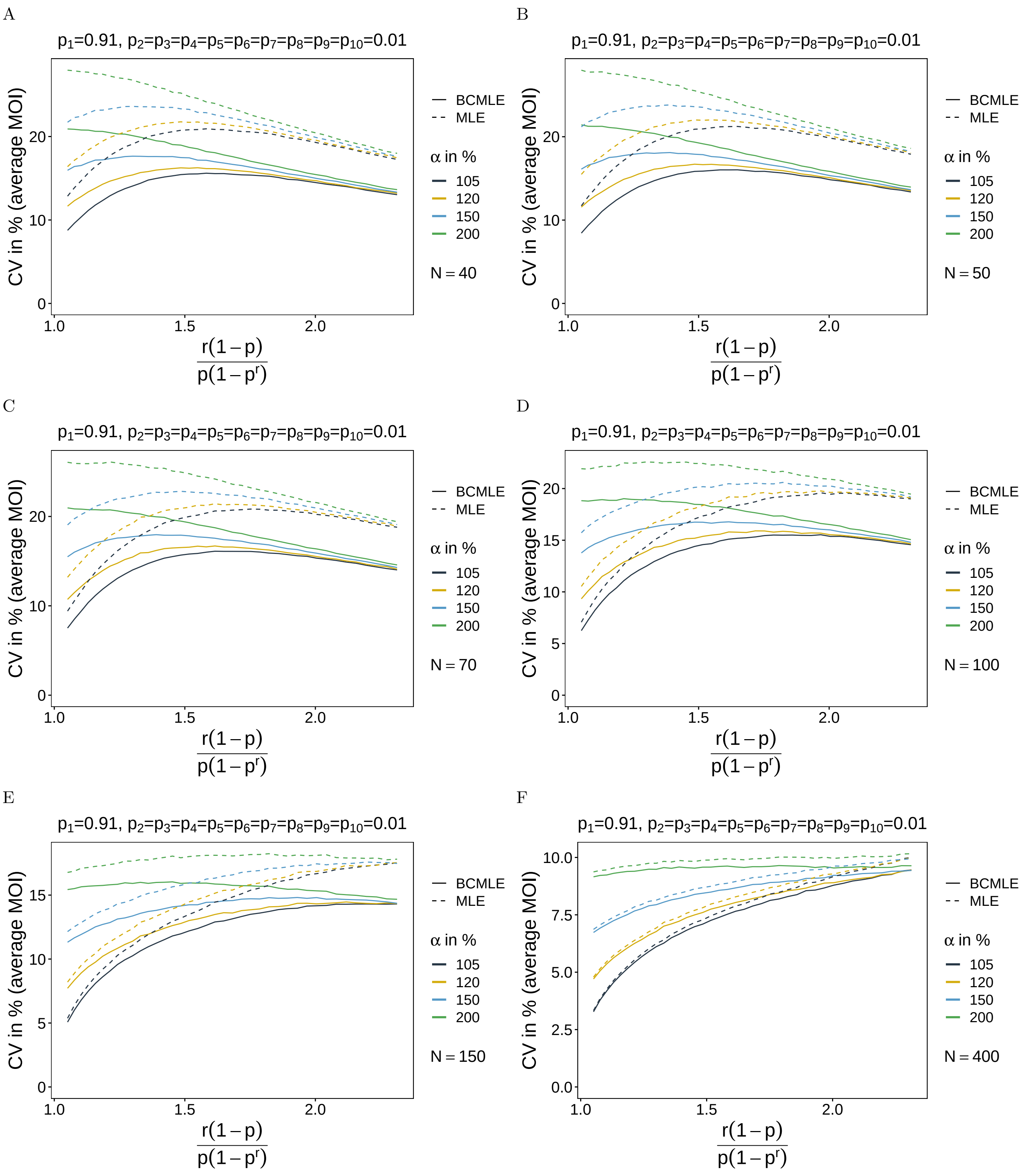

Figure 92: Similar to Figure 82 but for different lineage-frequency distributions.

5.3 Heuristically adjusted estimates

5.3.1 Relative bias in %

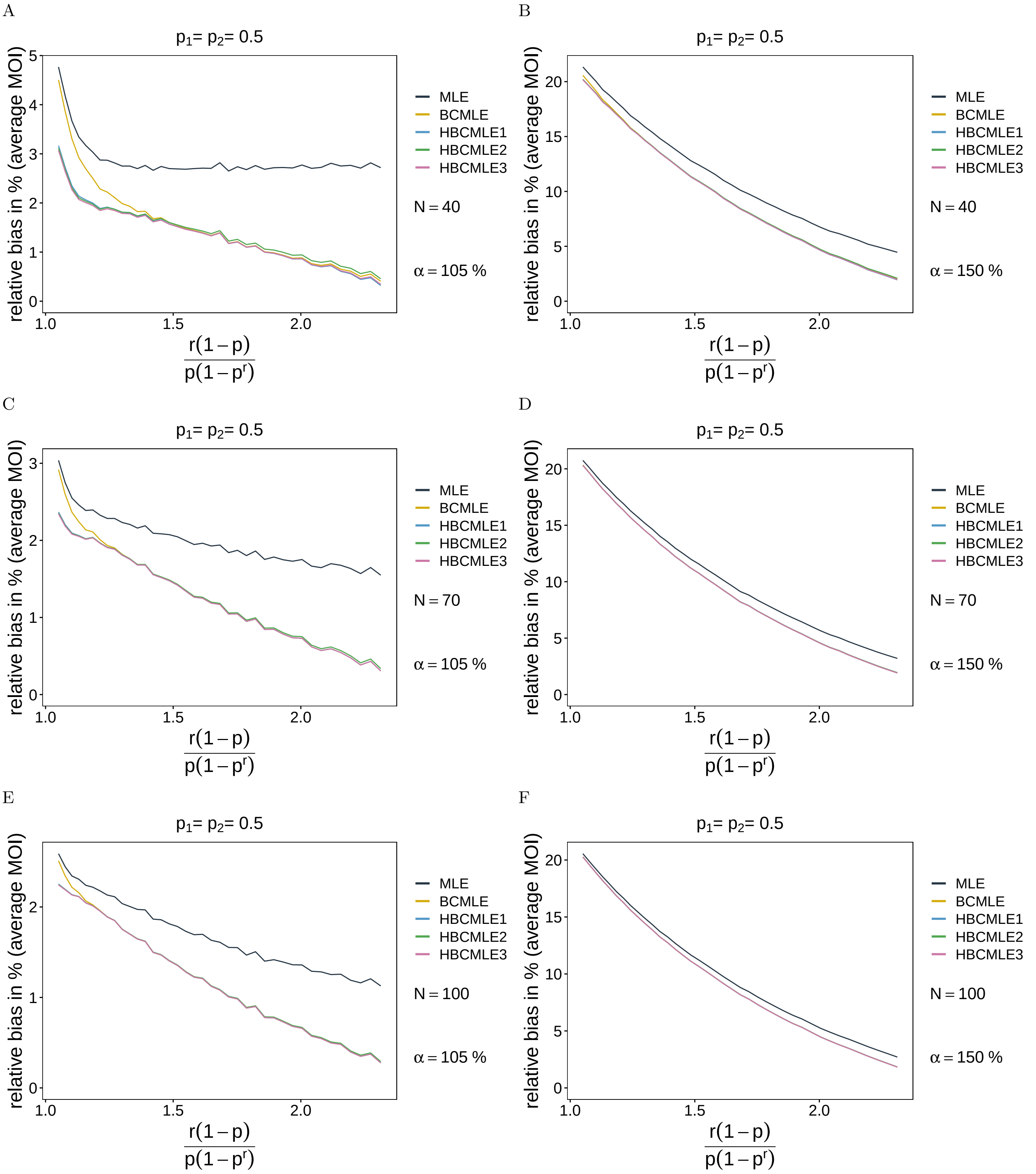

Figure 93: **Robustness of heuristically adjusted estimates against model violations.** The figure shows the relative bias in % of heuristically adjusted estimators (HBCMLE1 = $\hat{\psi}^{(hbc1)}$, HBCMLE2 = $\hat{\psi}^{(hbc2)}$ and HBCMLE3 = $\hat{\psi}^{(hbc3)}$) along with the relative bias in % of the MLE $\hat{\psi}$ and the BCMLE $\hat{\psi}^{(\mathrm{bc})}$ as a function of the true parameter $\psi = \frac{r(1-p)}{p(1-p^r)}$. The datasets are generated from the conditional negative binomial model whereas the estimates are derived from the conditional Poisson model. The panels assume a unique lineage-frequency distribution $\boldsymbol{p}$ shown at the top of each panel. Each panel is different in level of over-dispersion (specified by α in percent), and sample size N. The colored lines correspond to different estimators. The relative bias in each panel is derived from $S = 10,000$ simulated datasets of the specified sample size.

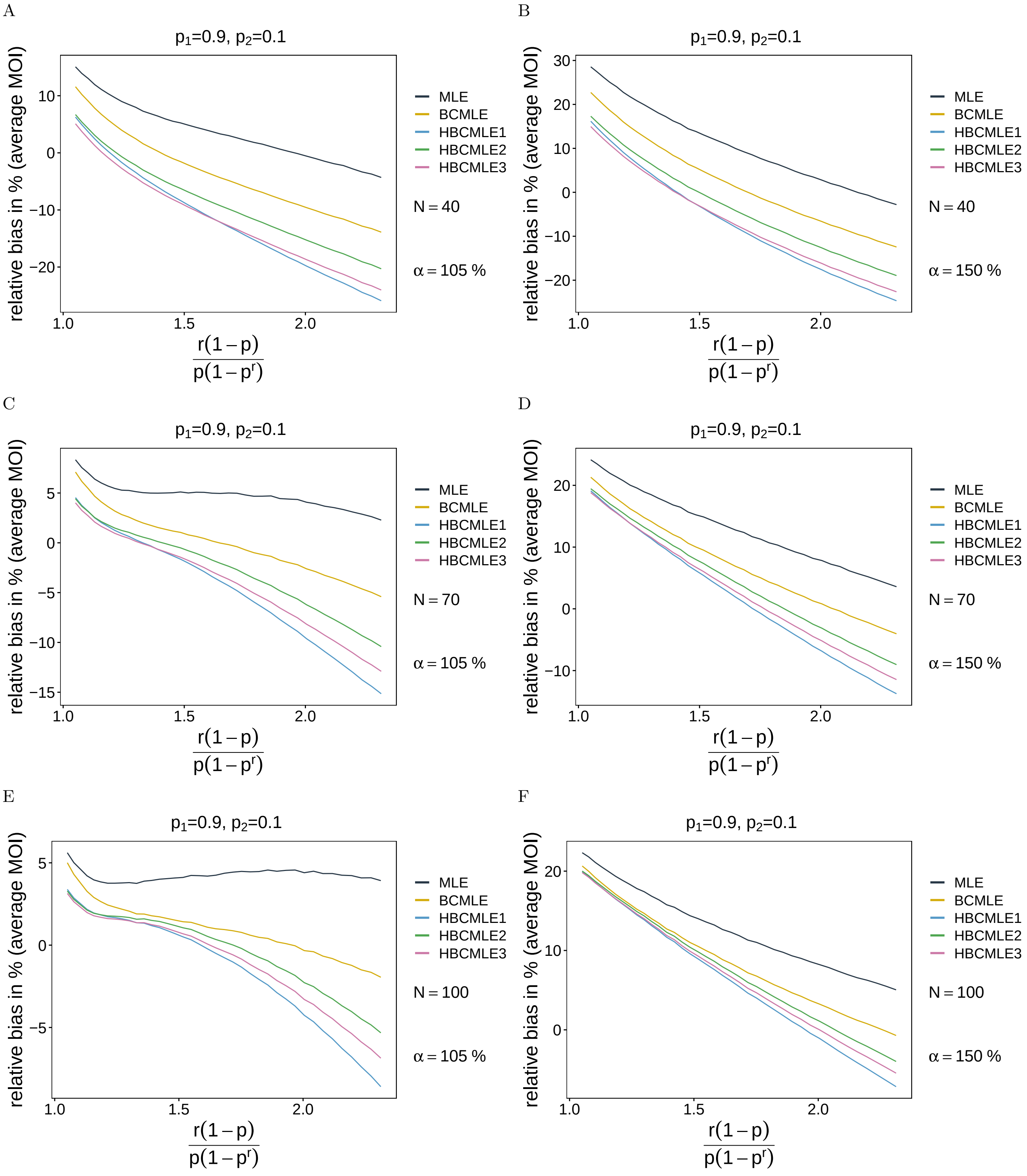

Figure 94: Similar to Figure 93 but for a different lineage-frequency distribution.

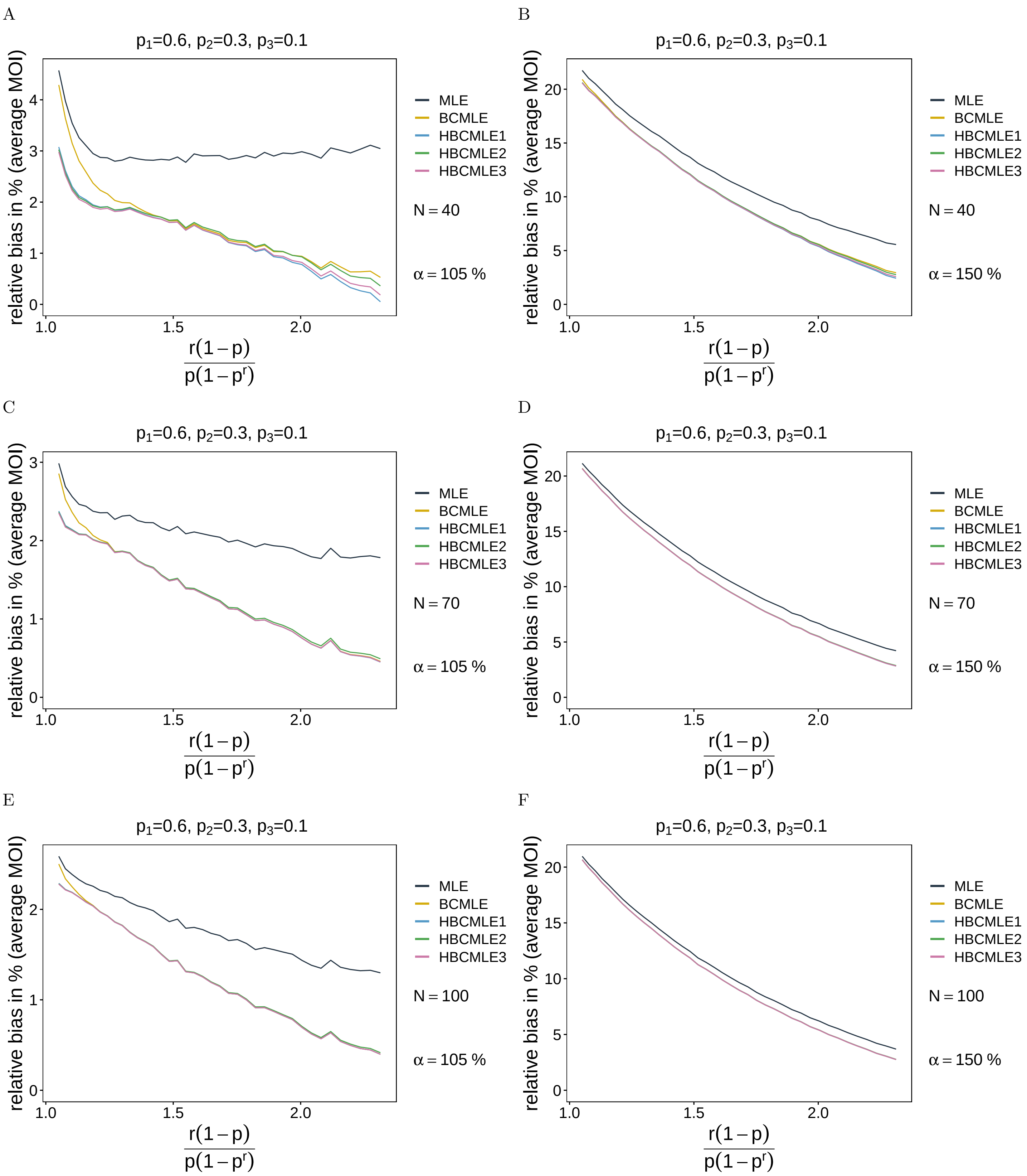

Figure 95: Similar to Figure 93 but for a different lineage-frequency distribution.

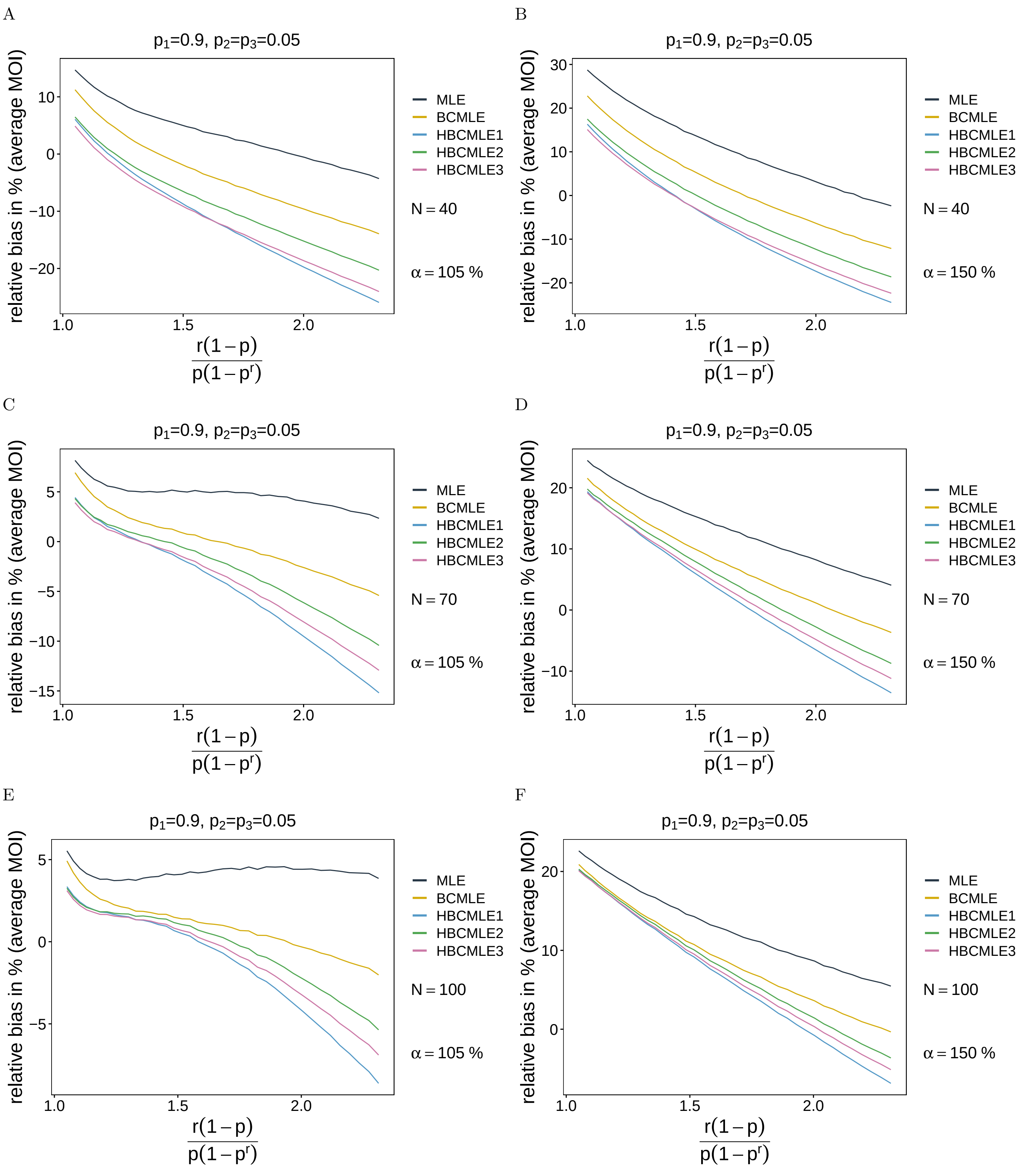

Figure 96: Similar to Figure 93 but for a different lineage-frequency distribution.

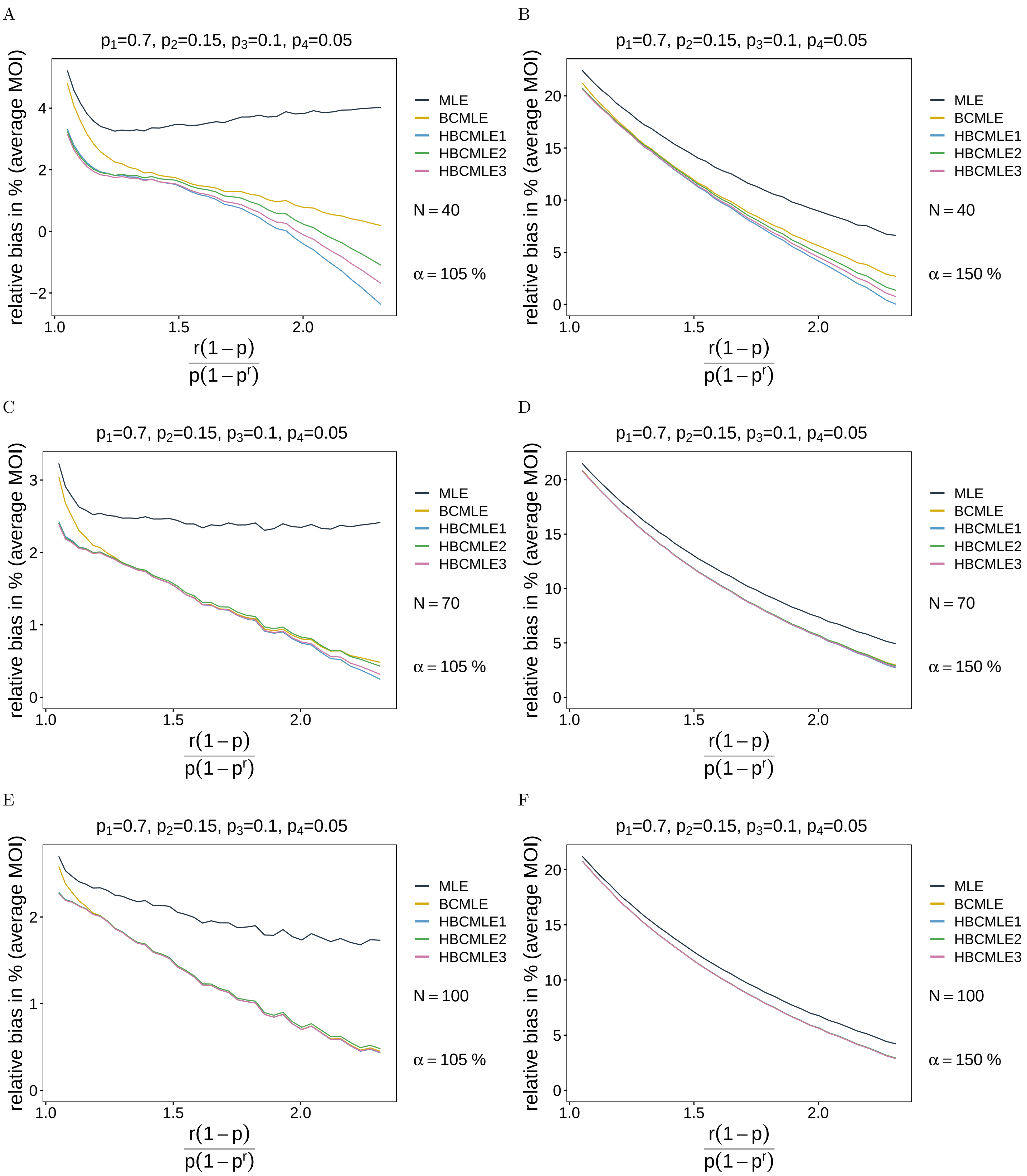

Figure 97: Similar to Figure 93 but for a different lineage-frequency distribution.

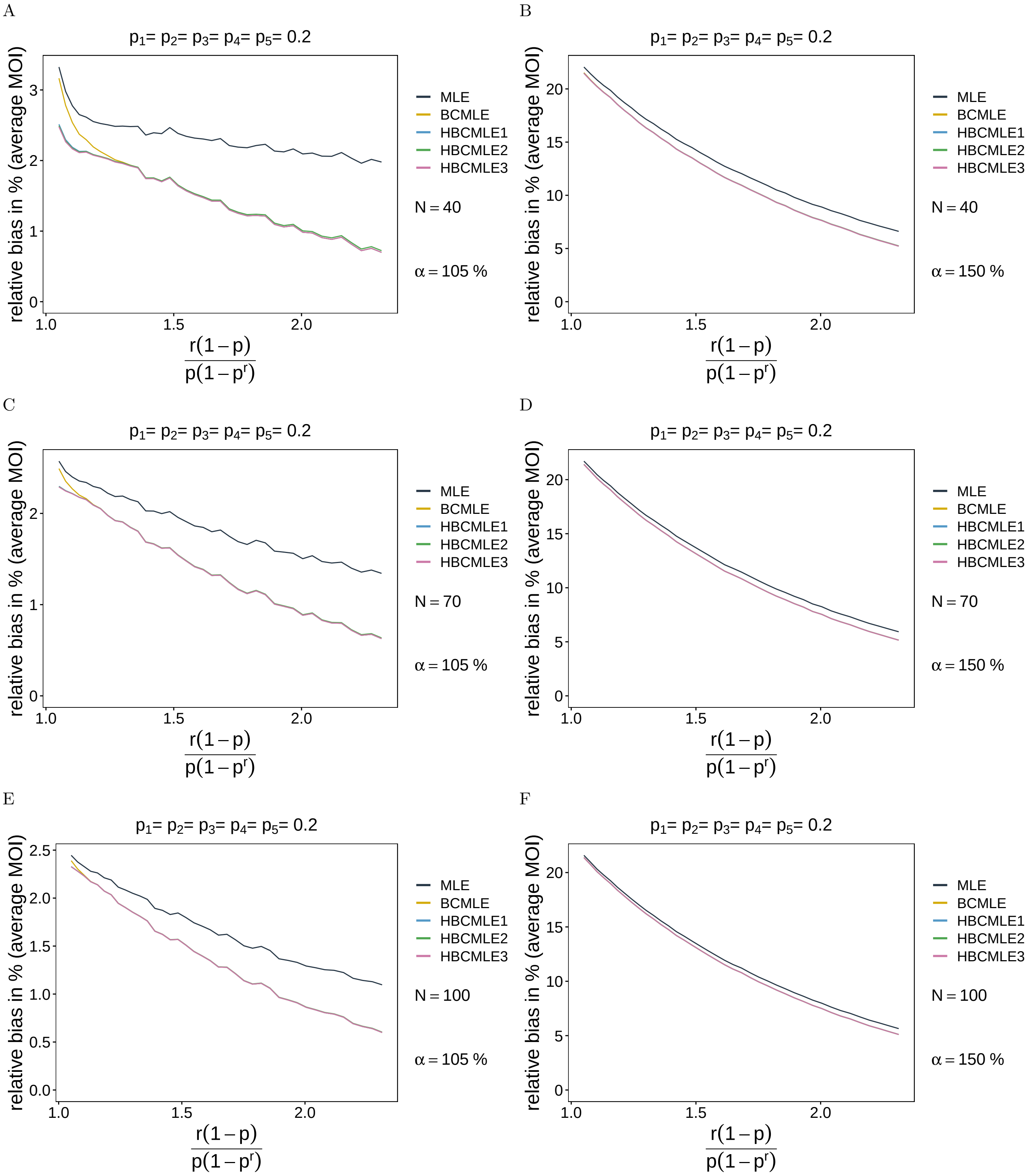

Figure 98: Similar to Figure 93 but for a different lineage-frequency distribution.

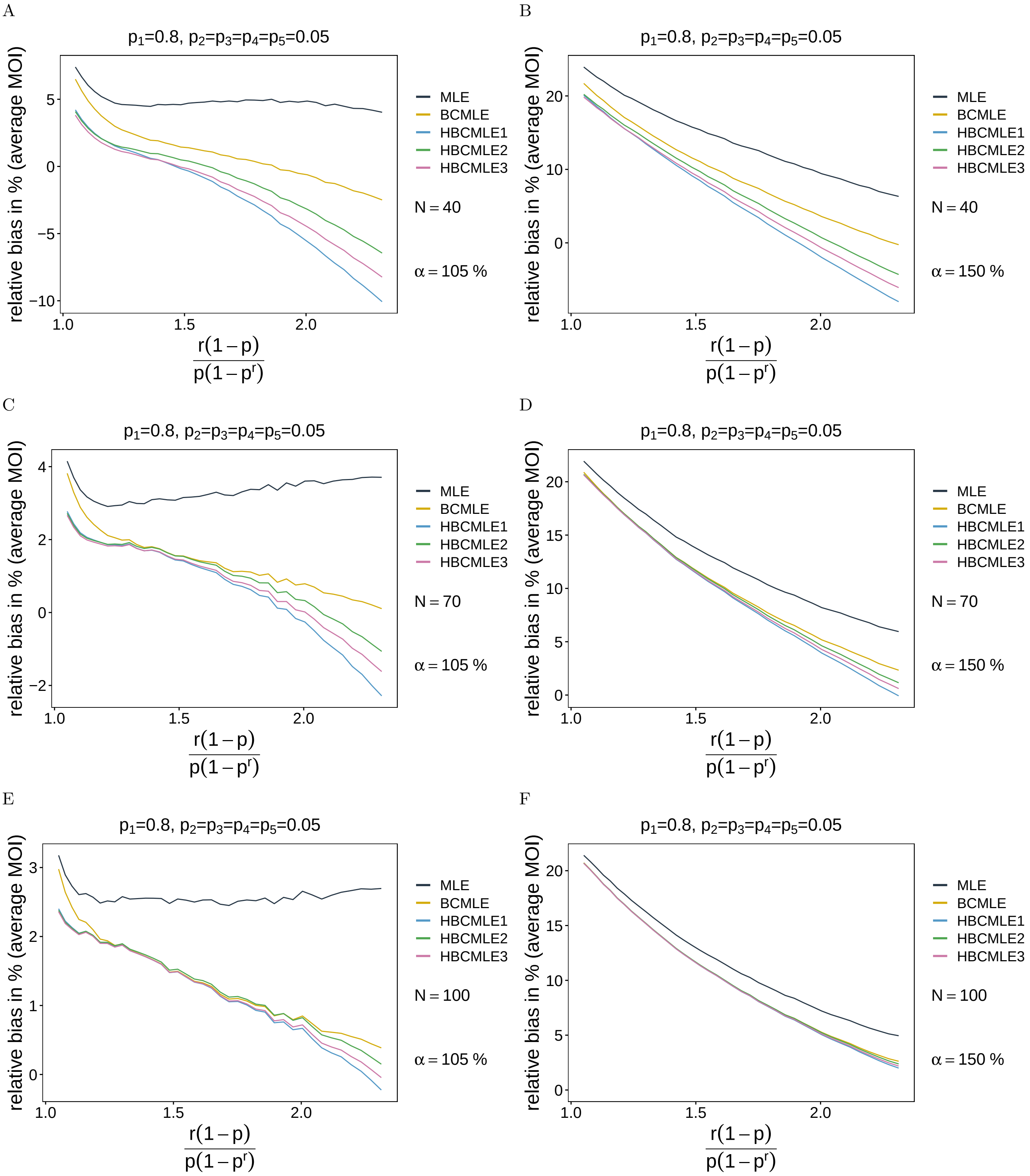

Figure 99: Similar to Figure 93 but for a different lineage-frequency distribution.

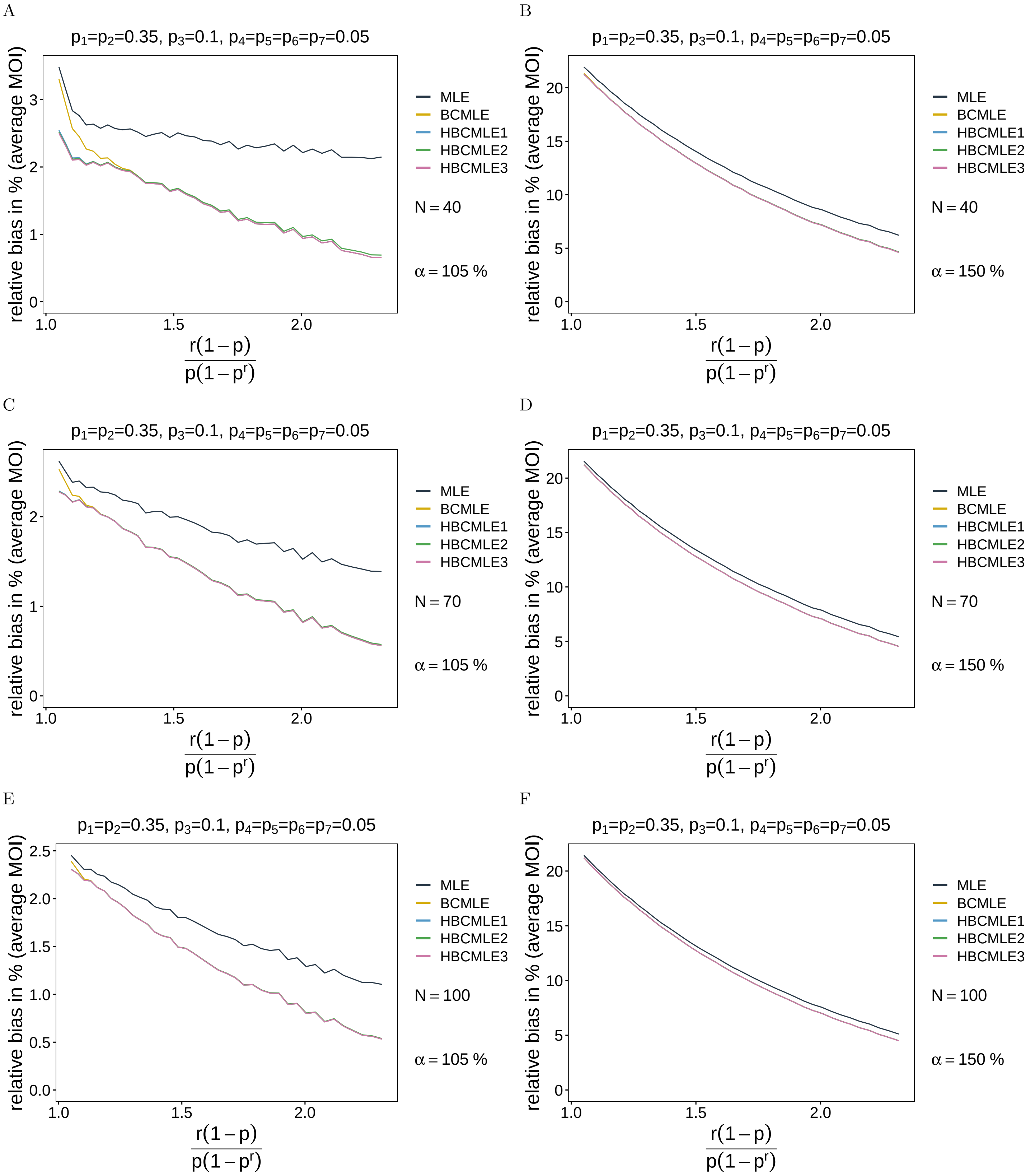

Figure 100: Similar to Figure 93 but for a different lineage-frequency distribution.

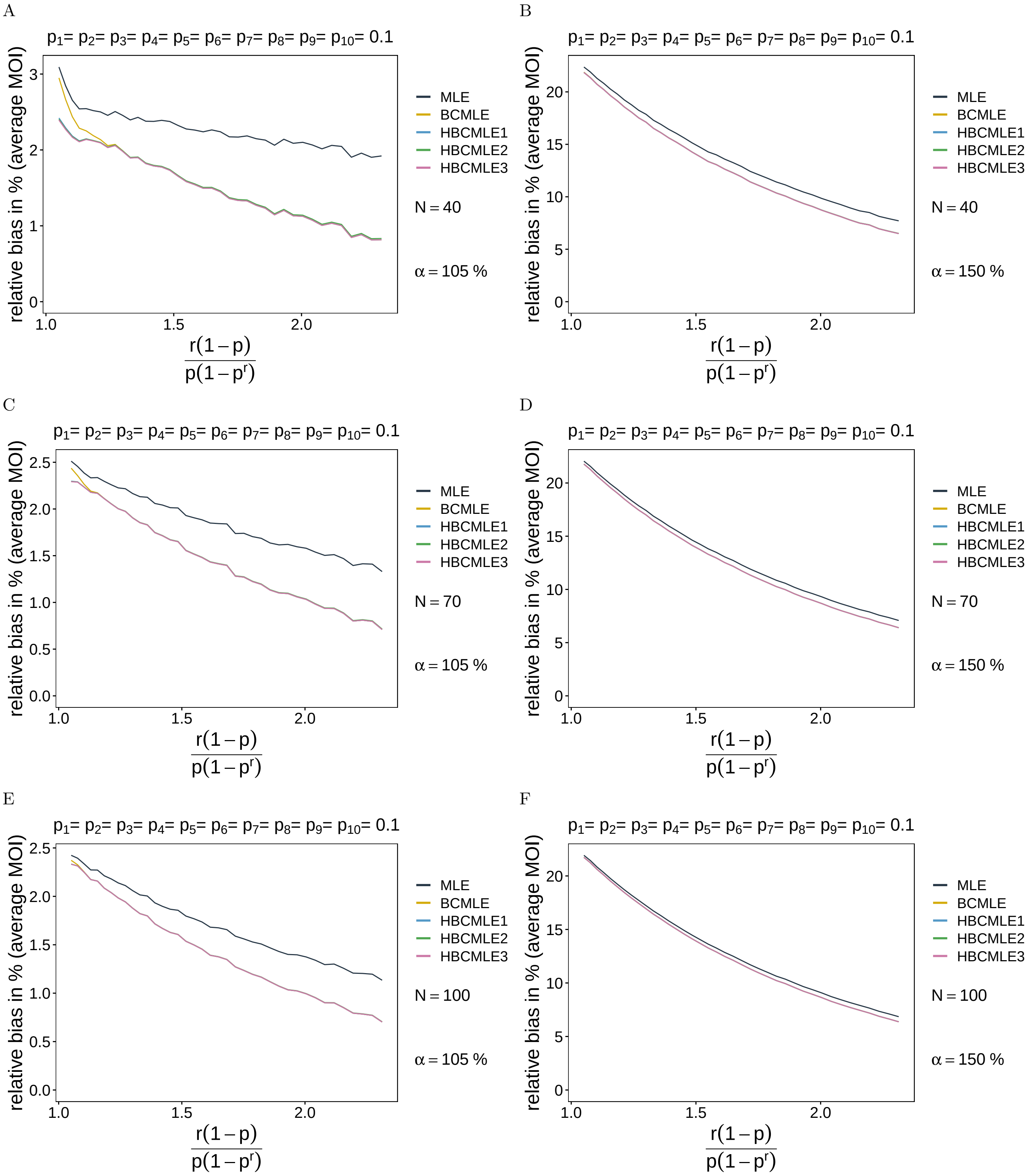

Figure 101: Similar to Figure 93 but for a different lineage-frequency distribution.

5.3.2 CV in %

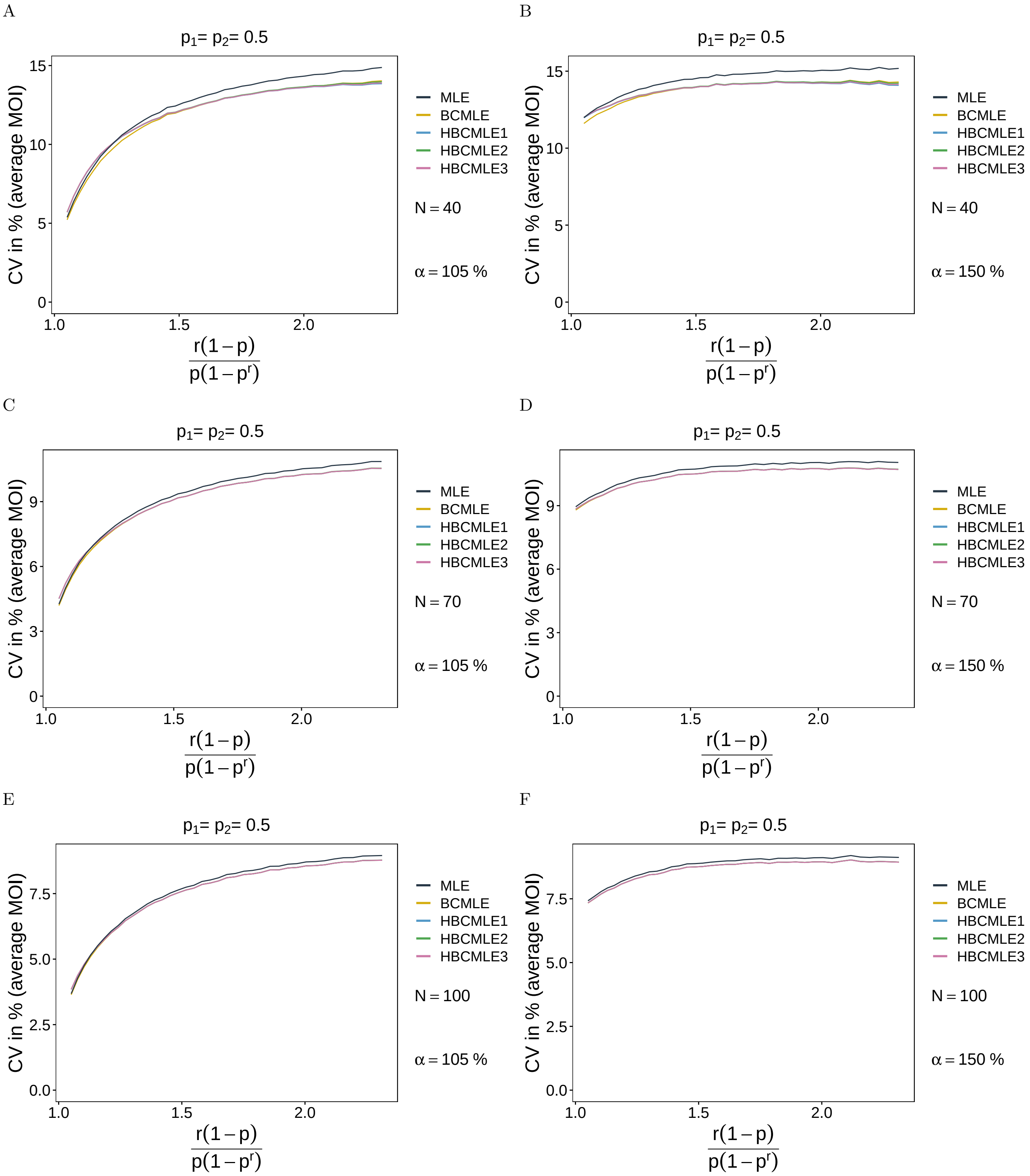

Figure 102: **Variance of heuristically adjusted estimates against model violations.**. Similar to Figure 93 but for the coefficient of variation in %.

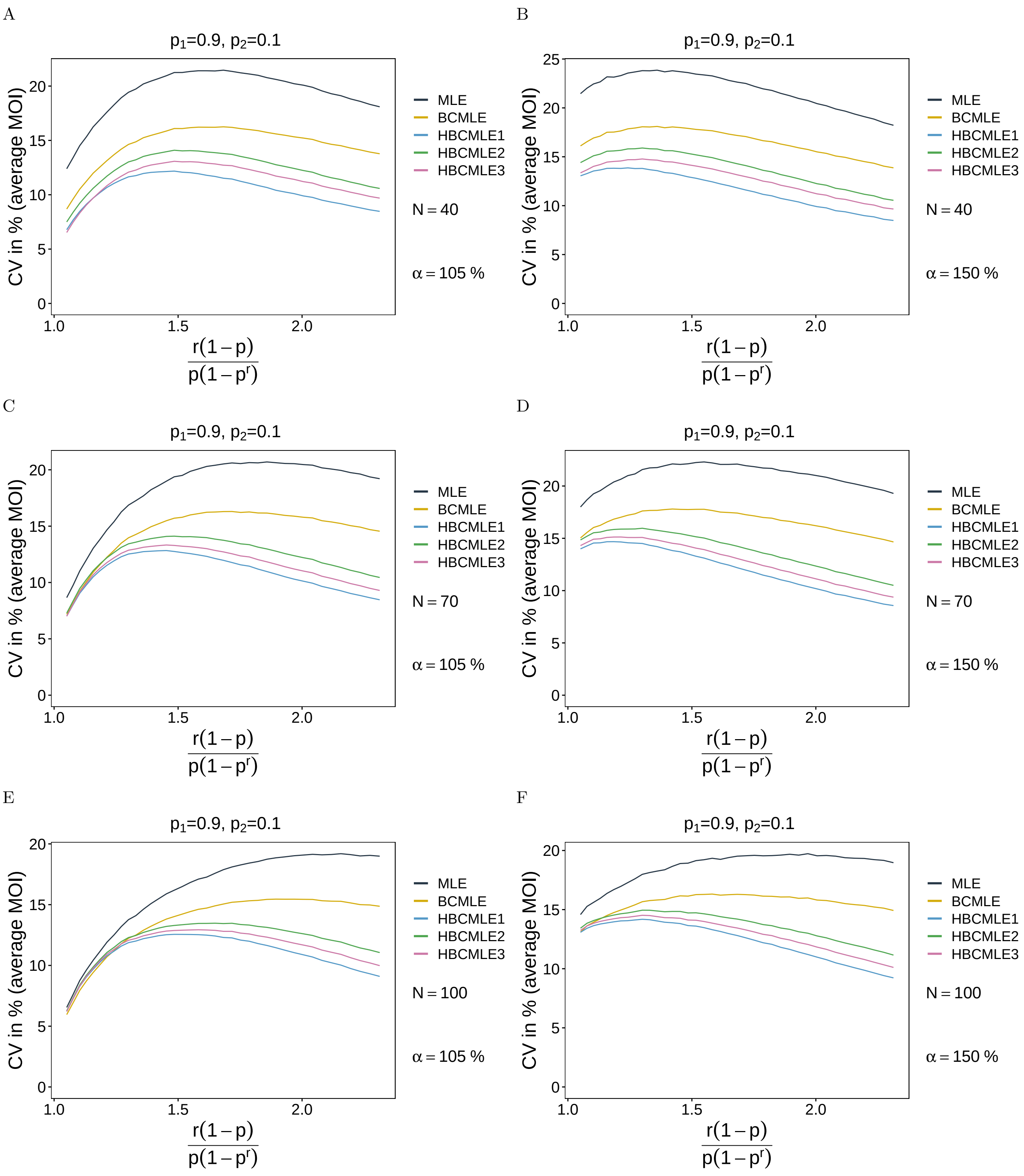

Figure 103: Similar to Figure 102 but for a different lineage-frequency distribution.

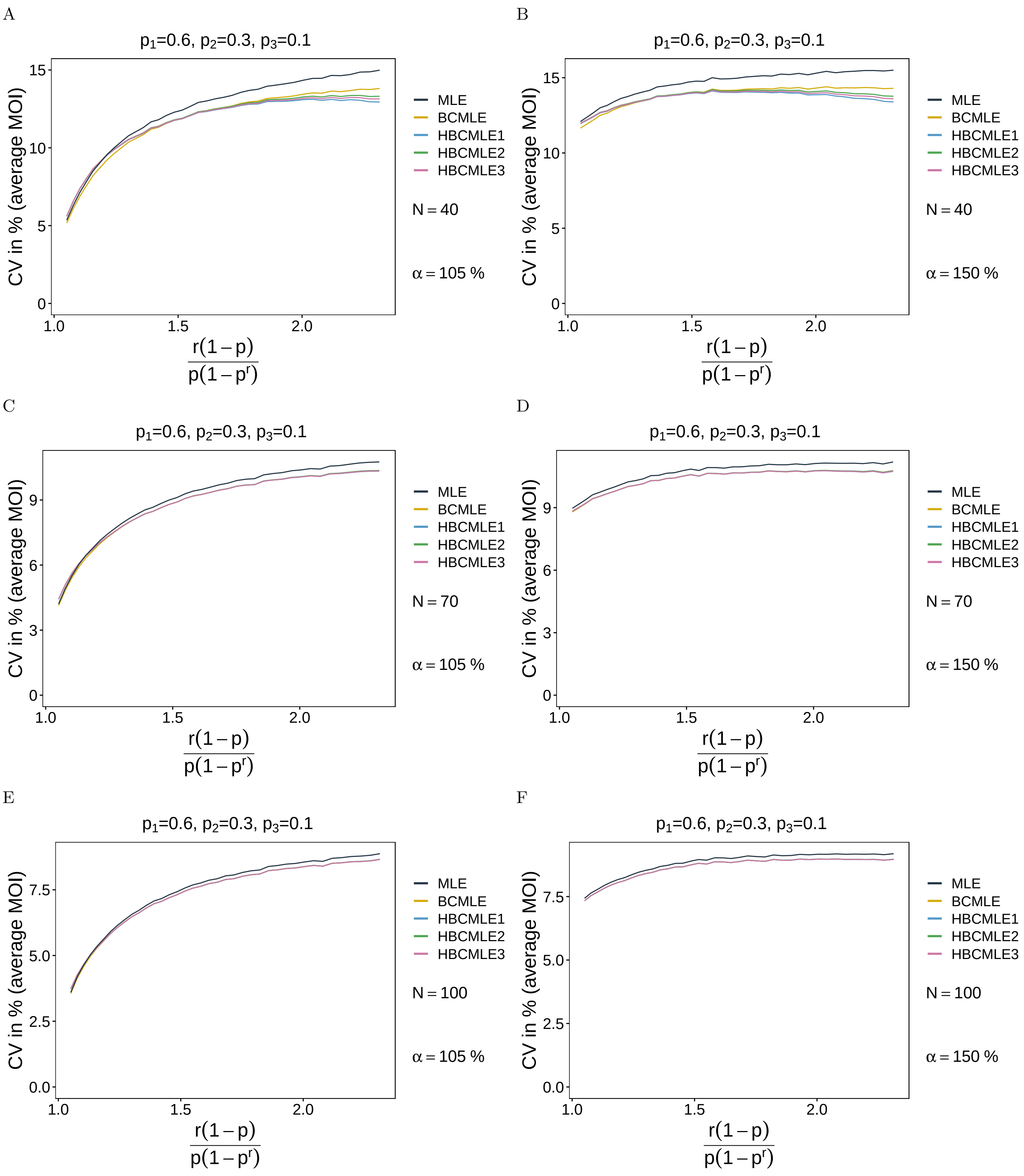

Figure 104: Similar to Figure 102 but for a different lineage-frequency distribution.

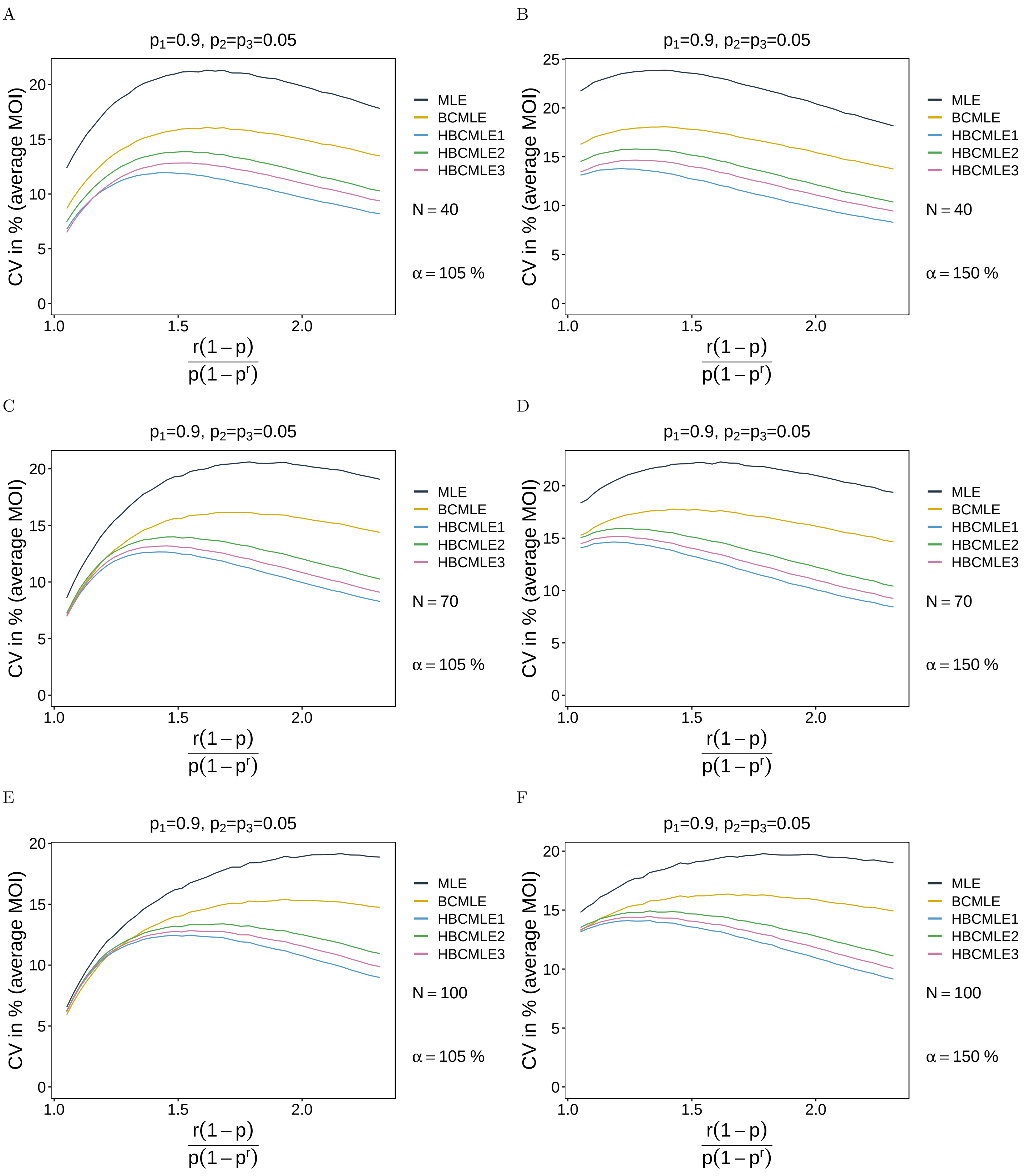

Figure 105: Similar to Figure 102 but for a different lineage-frequency distribution.

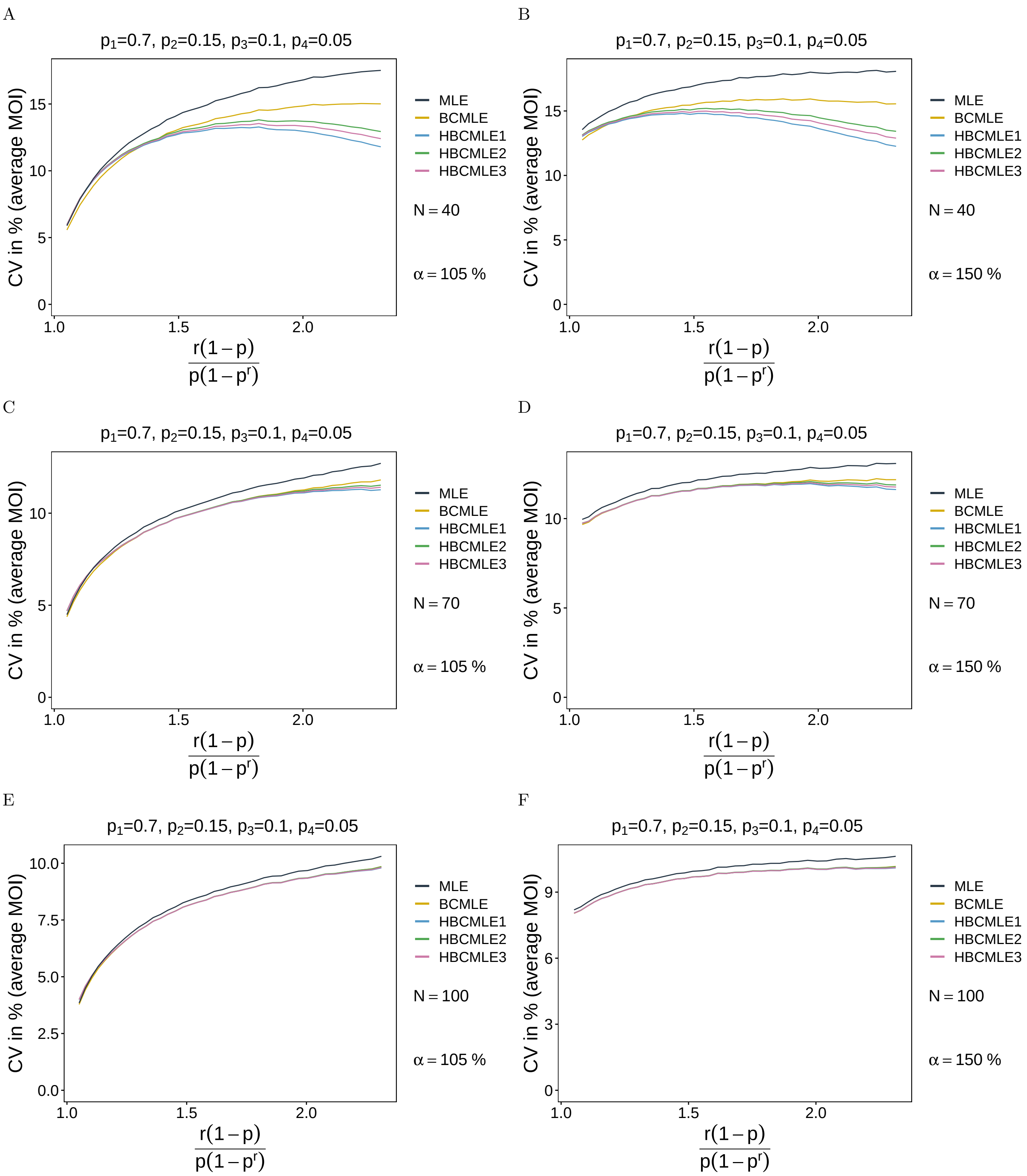

Figure 106: Similar to Figure 102 but for a different lineage-frequency distribution.

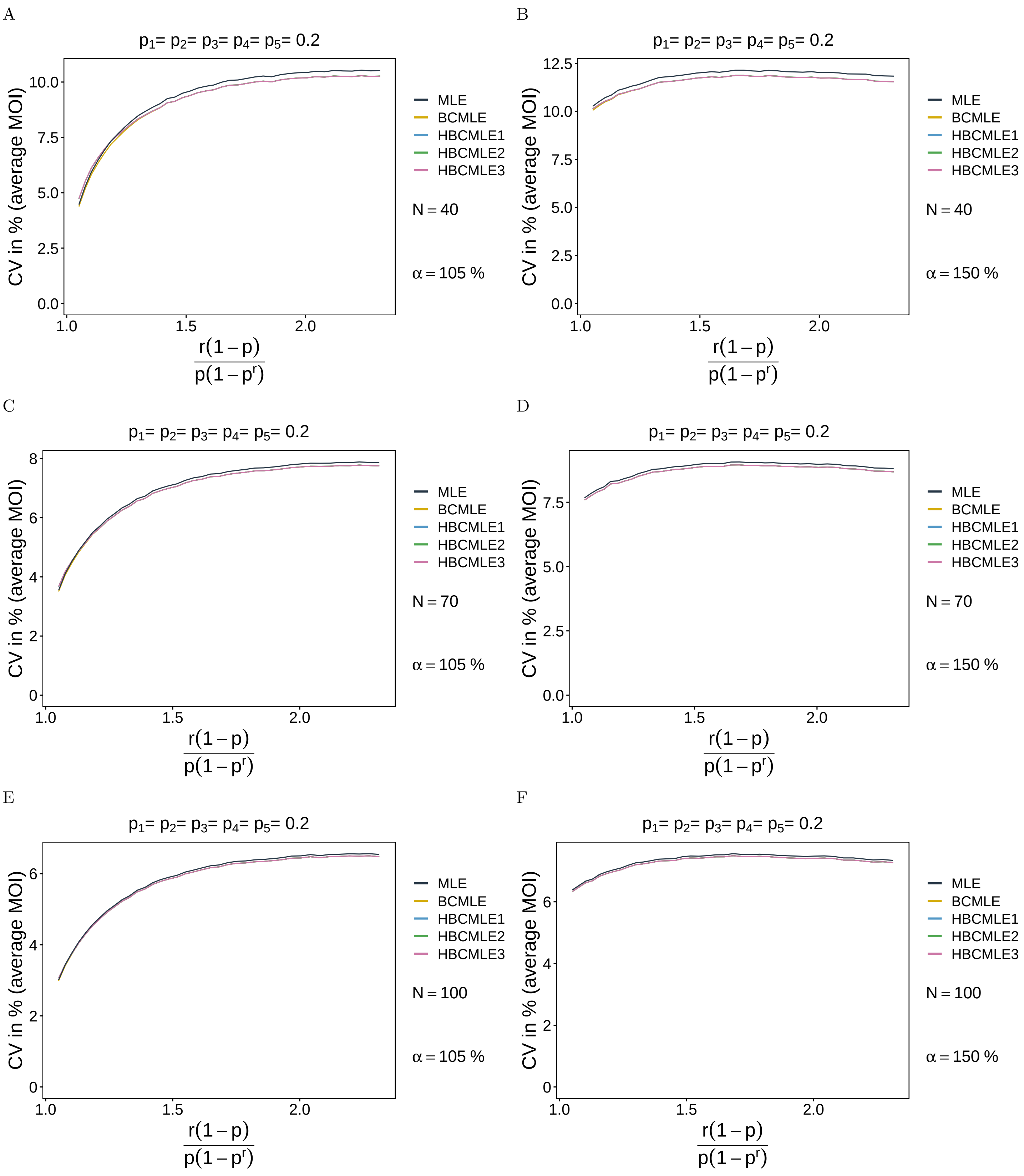

Figure 107: Similar to Figure 102 but for a different lineage-frequency distribution.

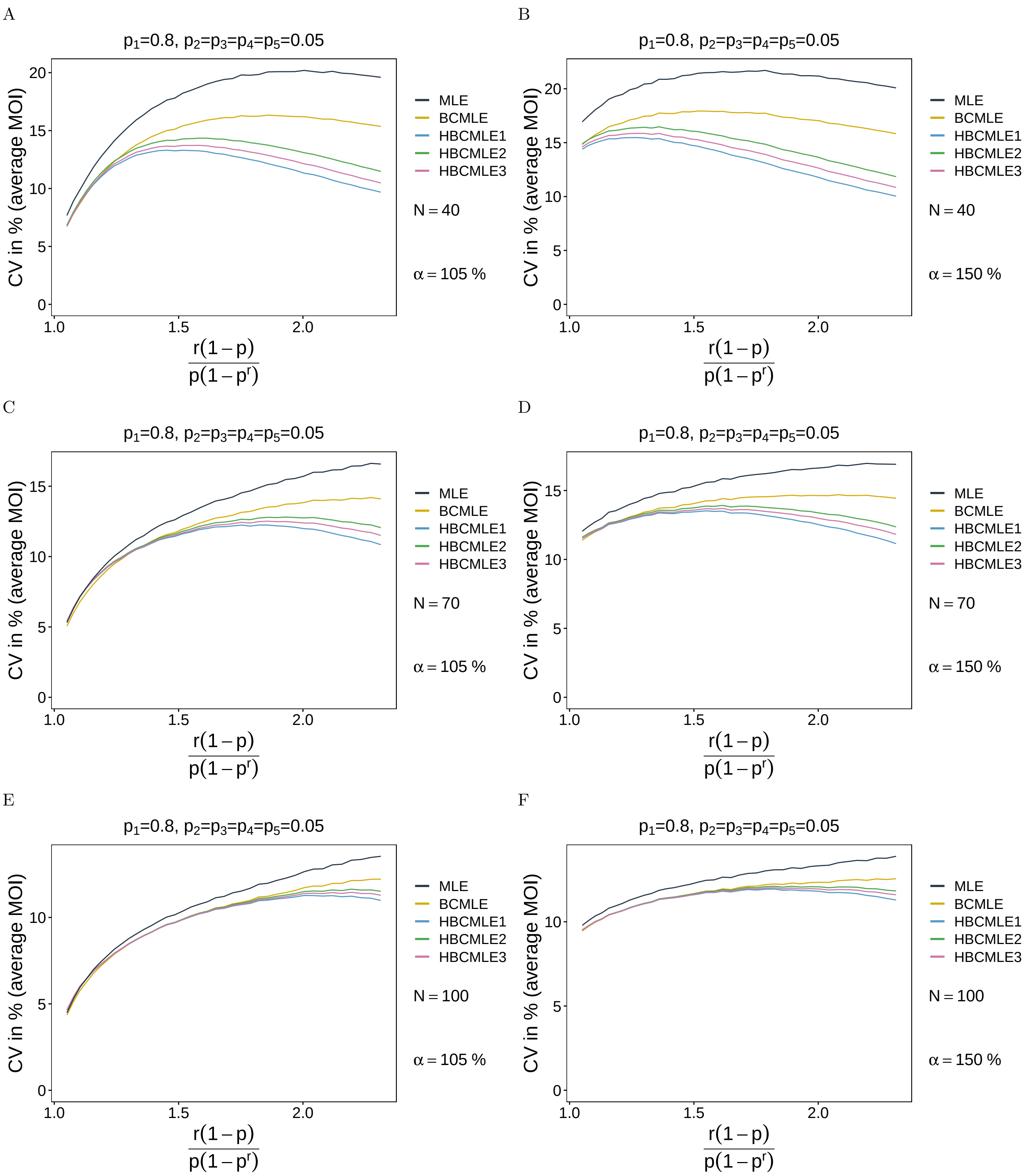

Figure 108: Similar to Figure 102 but for a different lineage-frequency distribution.

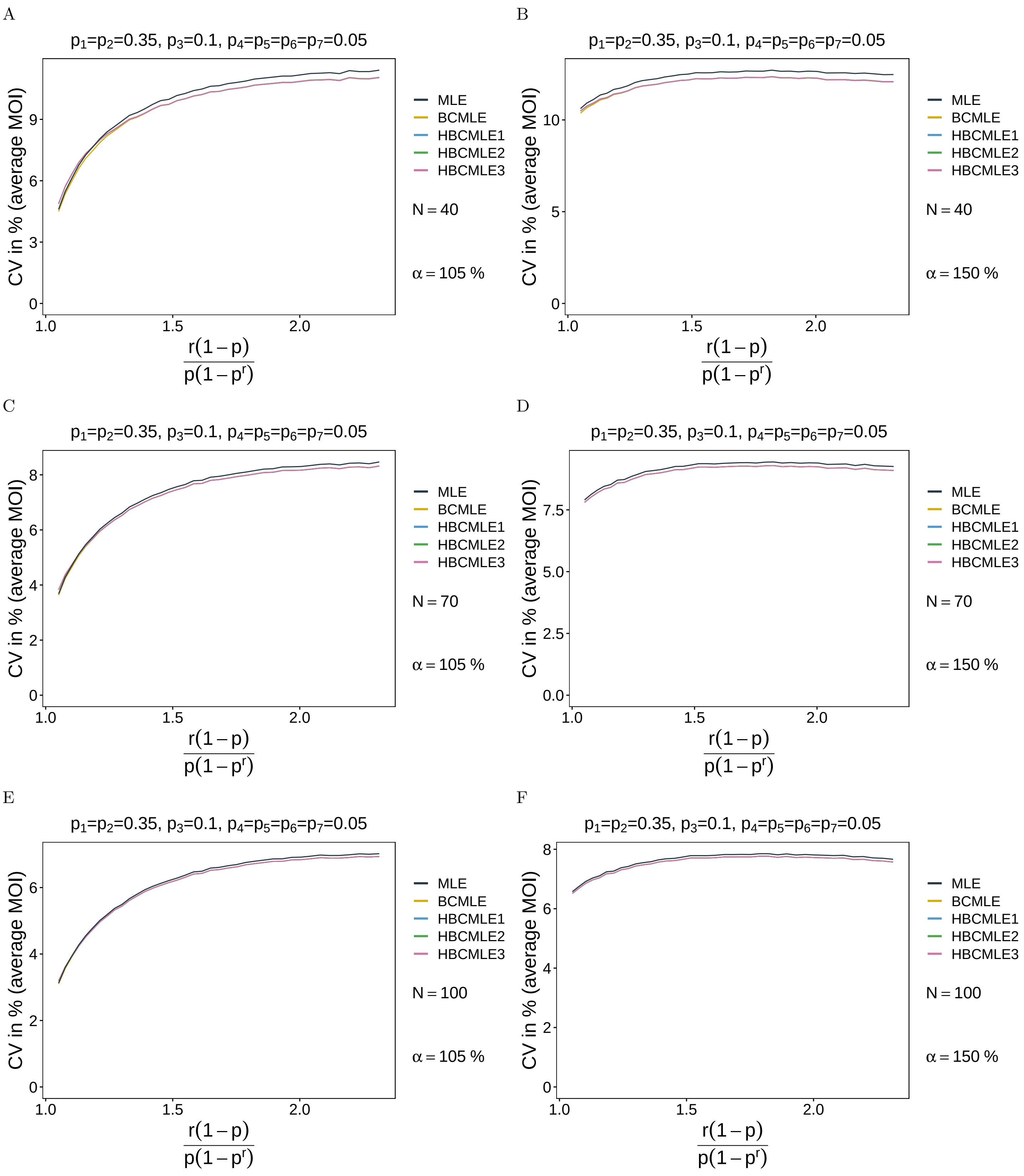

Figure 109: Similar to Figure 102 but for a different lineage-frequency distribution.

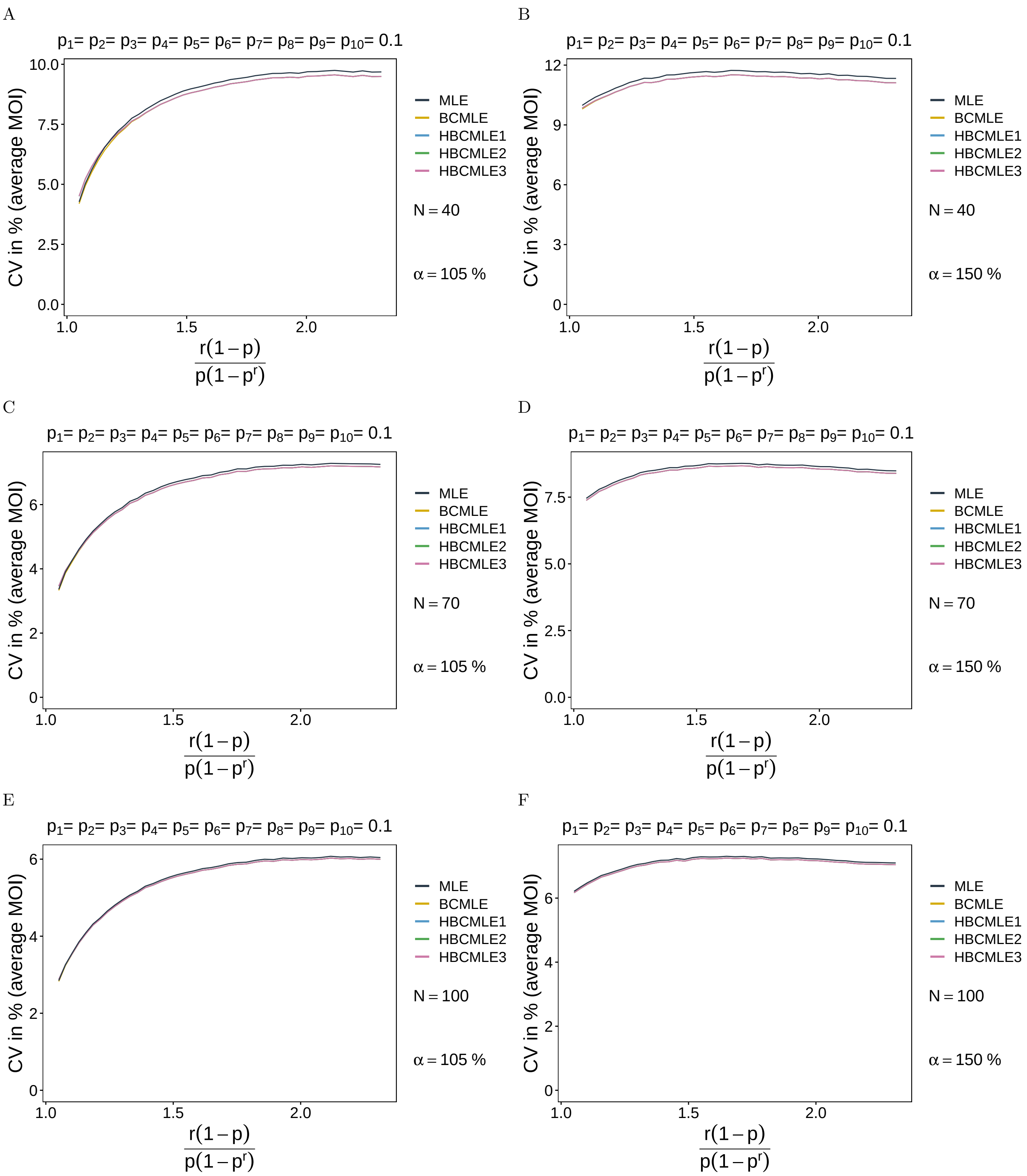

Figure 110: Similar to Figure 102 but for a different lineage-frequency distribution.
