# Supplementary material for: Bias-corrected maximum-likelihood estimation of multiplicity of infection and lineage frequencies
Source: PLoS One. 2021 Dec 29;16(12):e0261889. doi: 10.1371/journal.pone.0261889 (PMC8716058; doi:10.1371/journal.pone.0261889)
Supplement: S1 Table — (ZIP) [file pone.0261889.s003.zip › S1_Table.pdf]

Summary of Model Parameters for the Simulation Study		
par.	description	values
S	number of generated datasets	10 000
λ	Poisson parameter	$0.1, \ldots, 2$ in steps 0.05
ψ_{CP}	the average MOI	$\frac{\lambda}{1-e^{-\lambda}}$ with λ as above
ψ_{CNB}	the average MOI	$\frac{r(1-p)}{p(1-p^r)}$ with r and p s.t. $\psi_{CP} = \psi_{CNB}$
α	level of overdispersion	$1.05, 1.10, 1.20, 1.30, 1.50, 1.75, 2.00$
N	sample size	$40, 50, 60, 70, 80, 100, 150, 200, 300, 400$
n	number of lineages	$2, 3, 4, 5, 7, 8, 10$
$\boldsymbol{p}$	lineage-frequency distributions:	
	$n = 2$	$(0.5, 0.5), (0.6, 0.4), (0.7, 0.3), (0.8, 0.2), (0.9, 0.1)$
	$n = 3$	$(1/3, 1/3, 1/3), (0.5, 0.45, 0.05), (0.9, 0.05, 0.05),$
		$(0.6, 0.3, 0.1), (0.7, 0.2, 0.1), (0.8, 0.15, 0.05)$
		$(0.45, 0.45, 0.1)$
	$n = 4$	$(0.25, 0.25, 0.25, 0.25), (0.5, 0.25, 0.2, 0.05),$
		$(0.4, 0.2, 0.2, 0.2), (0.4, 0.4, 0.1, 0.1)$
		$(0.6, 0.3, 0.05, 0.05)$
	$n = 5$	$(0.2, 0.2, 0.2, 0.2, 0.2), (0.25, 0.2, 0.2, 0.2, 0.15),$
		$(0.5, 0.2, 0.2, 0.05, 0.05), (0.6, 0.2, 0.1, 0.05, 0.05),$
		$(0.7, 0.1, 0.1, 0.05, 0.05), (0.8, 0.05, 0.05, 0.05, 0.05)$
		$(0.3, 0.2, 0.2, 0.15, 0.15)$
	$n = 7$	$(0.35, 0.35, 0.1, 0.05, 0.05, 0.05, 0.05)$
		$(0.7, 0.05, 0.05, 0.05, 0.05, 0.05, 0.05)$
	$n = 8$	$(1/8, 1/8, 1/8, 1/8, 1/8, 1/8, 1/8, 1/8)$
		$(0.5, 0.1, 0.1, 0.1, 0.05, 0.05, 0.05, 0.05)$
	$n = 10$	$(0.1, 0.1, 0.1, 0.1, 0.1, 0.1, 0.1, 0.1, 0.1, 0.1)$
		$(0.4, 0.4, 0.09, 0.05, 0.01, 0.01, 0.01, 0.01, 0.01, 0.01)$
		$(0.7, 0.13, 0.1, 0.01, 0.01, 0.01, 0.01, 0.01, 0.01, 0.01)$
		$(0.91, 0.01, 0.01, 0.01, 0.01, 0.01, 0.01, 0.01, 0.01, 0.01)$

S1 Table. Summary of Model Parameters. Displayed are the parameter values used to generate datasets for the simulation study. The parameters were chosen so that the same MOI parameters were obtained for the conditional Poisson and conditional negative binomial model. A total number of S datasets were generated for each choice of model parameters and sample size (N) combination.
