# Supplementary figures and images for: Bias-corrected maximum-likelihood estimation of multiplicity of infection and lineage frequencies
Source: PLoS One. 2021 Dec 29;16(12):e0261889. doi: 10.1371/journal.pone.0261889 (PMC8716058; doi:10.1371/journal.pone.0261889)

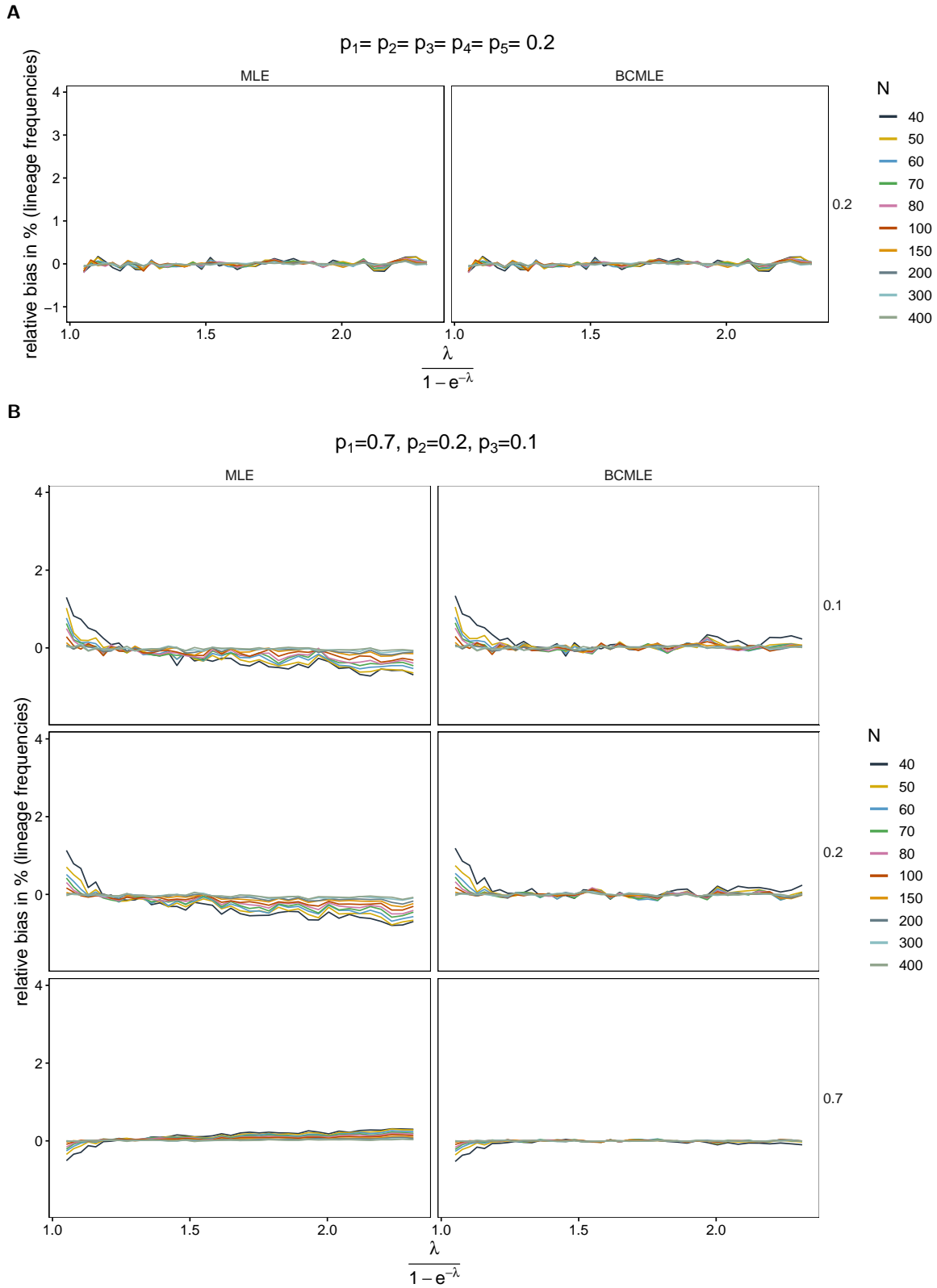

Supplement: S1 Fig — The figure shows the relative bias in % of the BCMLE (plots on the right in each panel) and the MLE (plots on the left in each panel) of lineage frequencies p as a function of the true parameter ψ based on simulated data created by the conditional Poisson model. Each panel assumes a different lineage-frequency distribution p shown at the top of each panel. In panel A, the relative bias in % of only one lineage frequency is illustrated, because all lineage frequencies are equal and their relative bias is almost identical. Different rows in panel B correspond to different lineage frequencies. Each colored line corresponds to a different sample size N. (ZIP) [file pone.0261889.s004.zip › S1_Fig.pdf]
