# Supplementary material for: Bias-corrected maximum-likelihood estimation of multiplicity of infection and lineage frequencies
Source: PLoS One. 2021 Dec 29;16(12):e0261889. doi: 10.1371/journal.pone.0261889 (PMC8716058; doi:10.1371/journal.pone.0261889)
Supplement: S2 Fig — As S1 Fig but for the BCMLE vs the HBCMLE2. (ZIP) [file pone.0261889.s005.zip › S2_Fig.pdf]

A

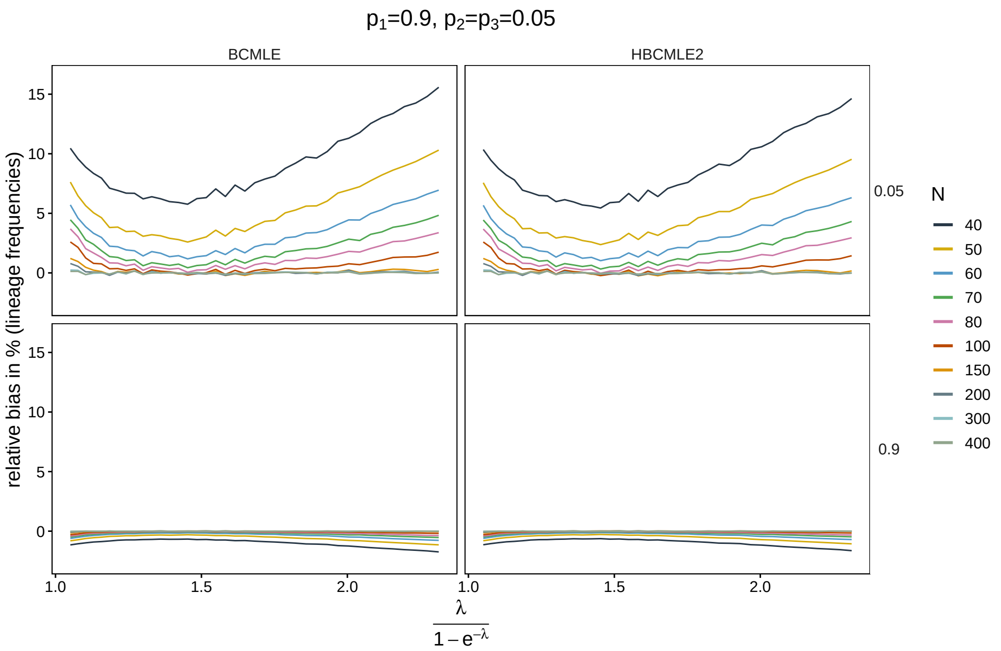

Fig 1. Bias of heuristically adjusted lineage-frequency estimates. As figure S1 Fig but for the BCMLE *vs* the HBCMLE2.
