# Supplementary material for: Bias-corrected maximum-likelihood estimation of multiplicity of infection and lineage frequencies
Source: PLoS One. 2021 Dec 29;16(12):e0261889. doi: 10.1371/journal.pone.0261889 (PMC8716058; doi:10.1371/journal.pone.0261889)
Supplement: S3 Fig — Similar to S1 Fig but for the coefficient of variation in %. The dotted lines are the respective predictions based on the Cramér-Rao lower bounds. (ZIP) [file pone.0261889.s006.zip › S3_Fig.pdf]

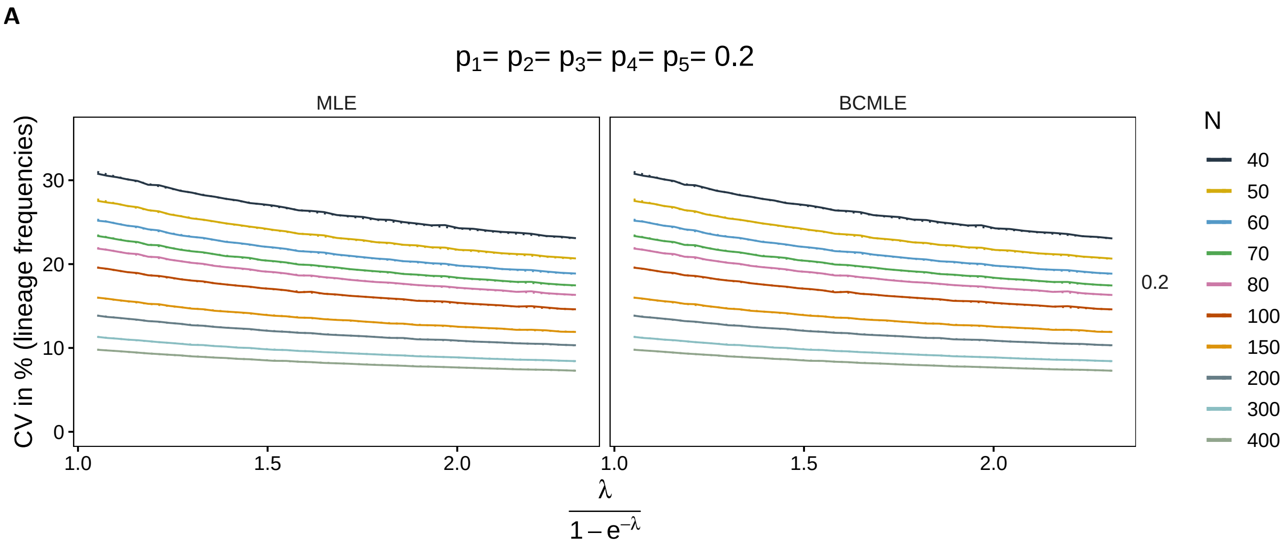

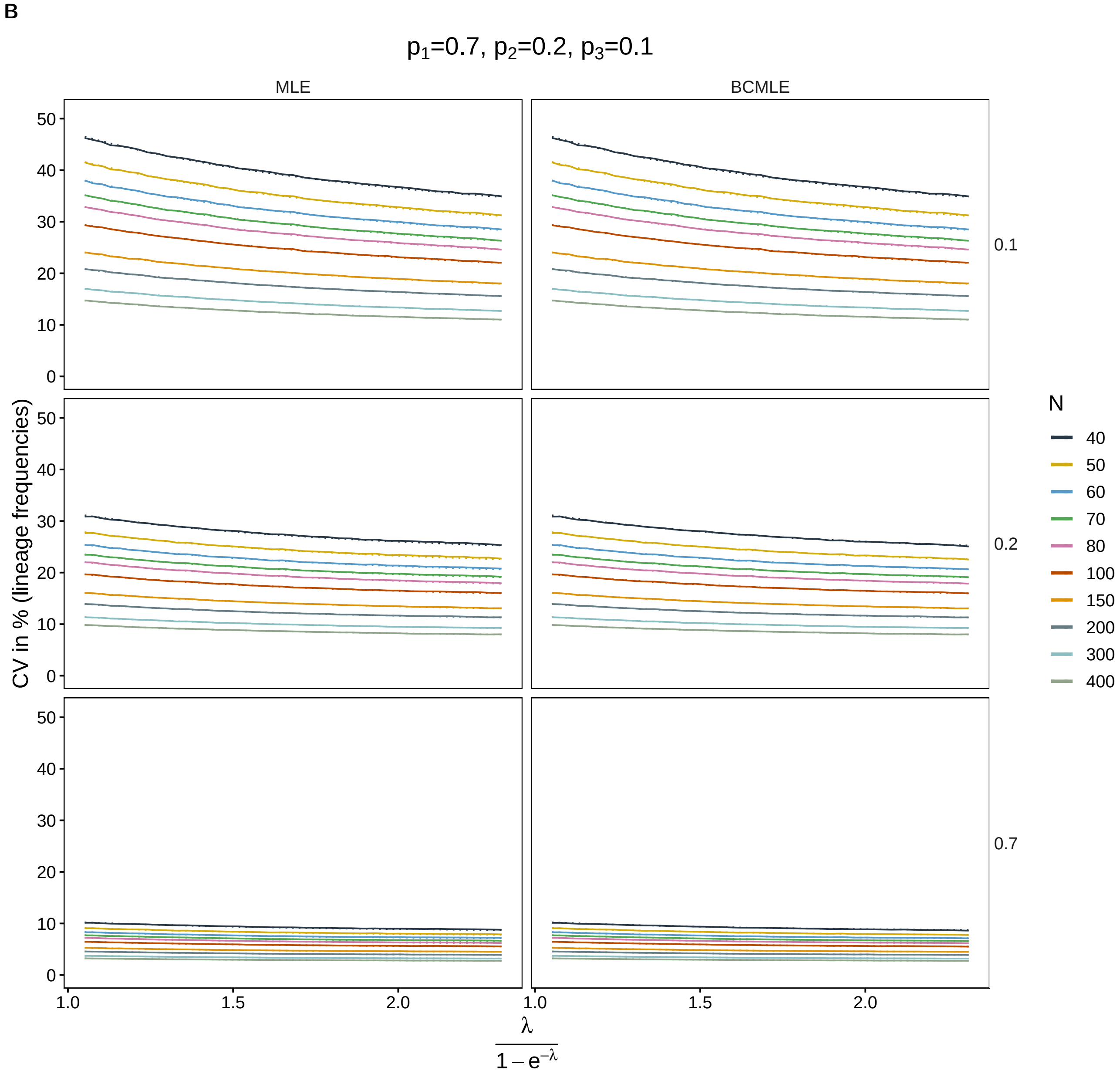

Fig 1. Variance of lineage frequency estimates. Similar to figure S1 Fig but for the coefficient of variation in %. The dotted lines are the respective predictions based on the Cramér-Rao lower bounds.
