# Supplementary material for: Bias-corrected maximum-likelihood estimation of multiplicity of infection and lineage frequencies
Source: PLoS One. 2021 Dec 29;16(12):e0261889. doi: 10.1371/journal.pone.0261889 (PMC8716058; doi:10.1371/journal.pone.0261889)
Supplement: S4 Fig — The figure shows the Euclidean distance (A) and Kullback-Leiber divergence (B) between the true frequencies and BCMLEs (solid lines) and the true frequencies and MLEs (dashed lines) for a lineage-frequency distribution as a function of the true parameter ψ based on simulated data created by the conditional Poisson model. The lineage-frequency distributions are shown at the top of each panel. Each colored line corresponds to a different sample size N. (ZIP) [file pone.0261889.s007.zip › S4_Fig.pdf]

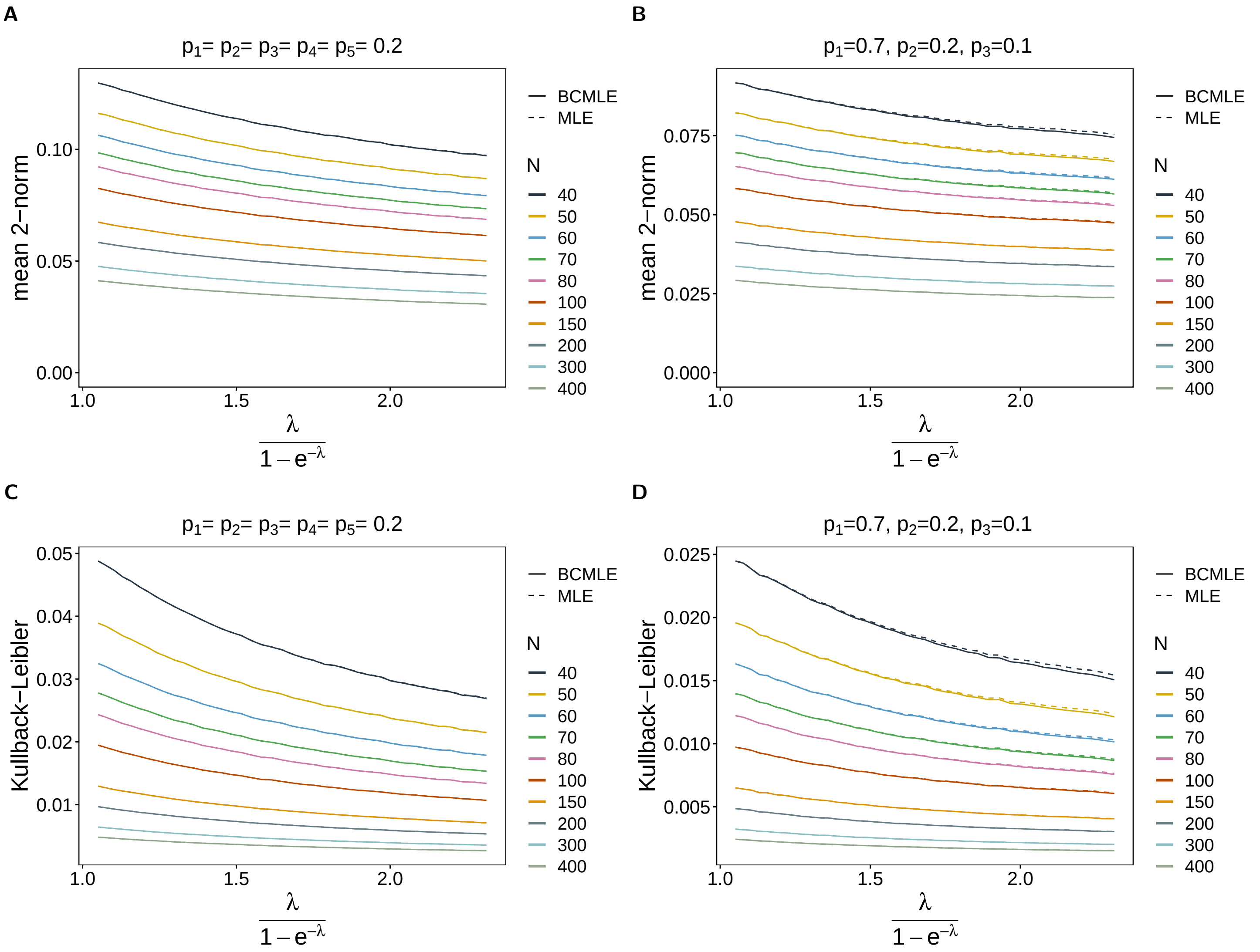

Fig 1. Euclidean distance and Kullback-Leiber divergence. The figure shows the Euclidean distance (**A**) and Kullback-Leiber divergence (**B**) between the true frequencies and BCMLEs (solid lines) and the true frequencies and MLEs (dashed lines) for a lineage-frequency distribution as a function of the true parameter ψ based on simulated data created by the conditional Poisson model. The lineage-frequency distributions are shown at the top of each panel. Each colored line corresponds to a different sample size N.
